# Evaluation of the genomic diversity of viruses infecting bacteria, archaea and eukaryotes using a common bioinformatic platform: steps towards a unified taxonomy

**DOI:** 10.1099/jgv.0.001110

**Published:** 2018-07-17

**Authors:** Pakorn Aiewsakun, Evelien M. Adriaenssens, Rob Lavigne, Andrew M. Kropinski, Peter Simmonds

**Affiliations:** ^1^​ Nuffield Department of Medicine, University of Oxford, Peter Medawar Building, South Parks, Oxford, OX1 3SY, UK; ^2^​ Department of Microbiology, Faculty of Science, Mahidol University, Bangkok, 10400, Thailand; ^3^​ Institute of Integrative Biology, University of Liverpool, Biosciences Building, Crown Street, L69 7ZB Liverpool, UK; ^4^​ Department of Biosystems, Laboratory of Gene Technology, KU Leuven. Kasteelpark Arenberg 21, Box 2462, 3001 Leuven, Belgium; ^5^​ Departments of Food Science, and Pathobiology, University of Guelph, 50 Stone Rd E, Guelph, ON, N1G 2W1, Canada

**Keywords:** virus, bacteriophage, prokaryote, eukaryote, metagenomic, taxonomy, virus classification, taxon, hidden Markov model, Baltimore classification

## Abstract

Genome Relationship Applied to Virus Taxonomy (GRAViTy) is a genetics-based tool that computes sequence relatedness between viruses. Composite generalized Jaccard (CGJ) distances combine measures of homology between encoded viral genes and similarities in genome organizational features (gene orders and orientations). This scoring framework effectively recapitulates the current, largely morphology and phenotypic-based, family-level classification of eukaryotic viruses. Eukaryotic virus families typically formed monophyletic groups with consistent CGJ distance cut-off dividing between and within family divergence ranges. In the current study, a parallel analysis of prokaryotic virus families revealed quite different sequence relationships, particularly those of tailed phage families (*Siphoviridae, Myoviridae* and *Podoviridae*), where members of the same family were generally far more divergent and often not detectably homologous to each other. Analysis of the 20 currently classified prokaryotic virus families indeed split them into 70 separate clusters of tailed phages genetically equivalent to family-level assignments of eukaryotic viruses. It further divided several bacterial (*Sphaerolipoviridae*, *Tectiviridae*) and archaeal (*Lipothrixviridae*) families. We also found that the subfamily-level groupings of tailed phages were generally more consistent with the family assignments of eukaryotic viruses, and this supports ongoing reclassifications, including *Spounavirinae* and *Vi1virus* taxa as new virus families. The current study applied a common benchmark with which to compare taxonomies of eukaryotic and prokaryotic viruses. The findings support the planned shift away from traditional morphology-based classifications of prokaryotic viruses towards a genome-based taxonomy. They demonstrate the feasibility of a unified taxonomy of viruses into which the vast body of metagenomic viral sequences may be consistently assigned.

## Introduction

The classification of viruses provides a catalogue of the vast diversity of viruses infecting eukaryotes, bacteria and archaea. The standard taxonomy is maintained by the International Committee on Taxonomy of Viruses (ICTV; https://talk.ictvonline.org/), and assigns viruses to a series of hierarchical taxa. Currently, 4404 species are assigned to 735 genera, which in turn are assigned to 122 families [1]. Members of certain families have more distant connections and may be further assigned currently to eight overarching orders. Despite their small size and simplicity relative to their hosts, viruses are extremely diverse in their gene complements, replication mechanisms and even their genetic material. While all other domains of life are based on double-stranded DNA genomes, virus genomes may comprise DNA or RNA, which can either be double- or single-stranded, linear or circular in topology and monocistronic or polycistronic. Genomes can be divided into multiple genome segments, which in some plant viruses may even be packaged into separate, independent virions. Another variable feature is genome size; the smallest known virus porcine circovirus has a genome of around 1780 bases and two genes while the largest known virus, pandoravirus, contains 2 473 870 base pairs with 1430 annotated genes [2]. Finally, and most problematic for a coherent classification scheme, viruses do not possess a universally present set of genes that are analogous to the ribosomal and replication-associated genes that have been widely used to create coherent taxonomies of prokaryotes and eukaryotes. Viruses are likely to have multiple, independent origins [3–6]. Reflecting this, the current virus taxonomy comprises a large number of groups that are unconnected at the levels of family and order.

Reflecting the diversity of genetic material and replication mechanisms, the Baltimore classification divides viruses into seven groups corresponding to those with double-stranded (ds)DNA genomes (group I), single-stranded (ss)DNA genomes (group II), dsRNA genomes (group III), ss(+)RNA genomes with a sense orientation of genes (group IV), ss(-)RNA genomes in antisense orientation (group V), ssRNA genomes with reverse transcription of a dsDNA replication intermediate (group VI) and dsDNA genomes with a ssRNA replication intermediate (group VII) [7]. While useful as a functional division of viruses, it maps imperfectly to virus taxonomy. For example, reverse-transcribing members of groups VI and VII are genetically similar despite their different genome compositions; some viruses with dsRNA genomes, such as *Hypoviridae,* are more akin to several ssRNA viruses in group IV. Conversely, most Baltimore groups additionally contain multiple evolutionarily unlinked virus groups.

The most diverse collections of viruses are those infecting bacteria. Many possess relatively large dsDNA genomes with extensive gene complements, encoding DNA replication enzymes and large, integrated sets of structural genes creating complex virion structures, required for cell entry, DNA packaging and virus exit [8–12]. Of these, the largest groups are classified in the order *Caudovirales*, the tailed phages [13, 14], within which three morphologically distinct families are recognized, namely the *Myoviridae*, *Podoviridae* and *Siphoviridae [15]*. The remainder, along with those infecting archaea, are classified into a further 17 separate families, 13 of which are specific to Archaea [16]. These assignments have historically been largely based upon morphology – the epithets myo-, sipho- and podo- describe the long contractile, long non-contractile and short tails of their respective families within the *Caudovirales*. Similarly, ampulla-, fusillo- and bicauda-, as examples, describe the bottle-, spindle- and two-tailed morphologies of archaeal virus families *Ampullaviridae*, *Fuselloviridae* and *Bicaudaviridae*.

However, the last decade has seen major shifts from this morphological classification towards a genome organization-based taxonomy (discussed in [17]), including the introduction of subfamilies and genera [18–20] and the use of genetics-based metrics of prokaryotic virus relatedness typically based upon nucleotide or more commonly protein sequence or proteome comparisons [5, 13, 21–24]. These methods can provide a relatively robust classification guide even for viruses with highly divergent genome sequences and organizations [5, 18–20, 25–27] despite high rates of horizontal gene transfer and mosaicism between some bacterial virus genomes [26, 28], particularly of temperate bacteriophages [29].

In a parallel approach for viruses infecting eukaryotes, we recently described the development and application of a genomics-based method (Genome Relationship Applied to Virus Taxonomy; GRAViTy) to quantify degrees of genetic relatedness within and between members of the 134 currently assigned eukaryotic virus families. This was based on the computation of composite generalized Jaccard (CGJ) distances between viruses, a metric that is based on homology detection between viral genes and similarities in genome organizational features (gene orders and orientations). On cross-validation with sampled datasets, GRAViTy showed high sensitivity and specificity in its assignment of known eukaryotic viruses to existing families and conversely, in assignment of unknown viruses as novel viruses. The method also predicted the existence of potentially more than 100 additional family-level groupings upon analysis of metagenomic datasets, providing a method by which the traditional morphological and phenotypic-based ICTV taxonomy could be extended to include viruses only known from their genome sequence.

The ability of GRAViTy to identify and assign families was assisted by a degree of consistency in relatedness between the genomes and genes of members of existing family-level taxa [30]. Typically, members of the same family possess genes that are largely homologous, particularly those encoding replication and structural proteins. These shared gene complements underlie common replication mechanisms and their morphologically similar, often distinctive, virions. The criteria used to group virus families into orders are, however, more variable, often based upon distant relatedness of replication enzymes. For example, members of *Mononegavirales* were grouped together based on sequence similarity in their RNA-dependent RNA polymerase (RdRp) proteins and their non-segmented genomes. The presence of a reverse transcriptase coding gene was used to classify group VI viruses into the *Ortervirales* order together with some of the group VII viruses. In contrast, the *Herpesvirales* order was formed on the basis of distinctive virion morphologies, while a combination of RdRp phylogeny and morphology was used to assign viruses into the *Picornavirales* order [31].

In this study, we have addressed the question of whether the same relationship between genetic similarity and family assignments existed among the currently described bacterial and archaeal virus families and orders. We envisaged this being problematic at the outset because their current taxonomy comprises only 20 families and two orders, compared to the 102 families and six orders of eukaryotic viruses. Furthermore, bacterial and archaeal virus taxa have been largely assigned on the basis of morphological criteria and may not reflect genome relatedness on which GRAViTy is based. To investigate this formally, we computed CGJ distances between currently classified bacterial and archaeal viruses and scored their relatedness to each other and to eukaryotic viruses in Baltimore groups I–IV (dsDNA, ssDNA, dsRNA and ss(+)RNA). This analysis revealed a major inconsistency in the way that some prokaryotic viruses are classified. This will need to be addressed if future genomics-based classification of metagenomically derived viruses are to be incorporated into the ICTV taxonomy [32].

## Results

### Sequence relatedness of viruses infecting bacteria, Archaea and Eucarya

**Fig. 1.**
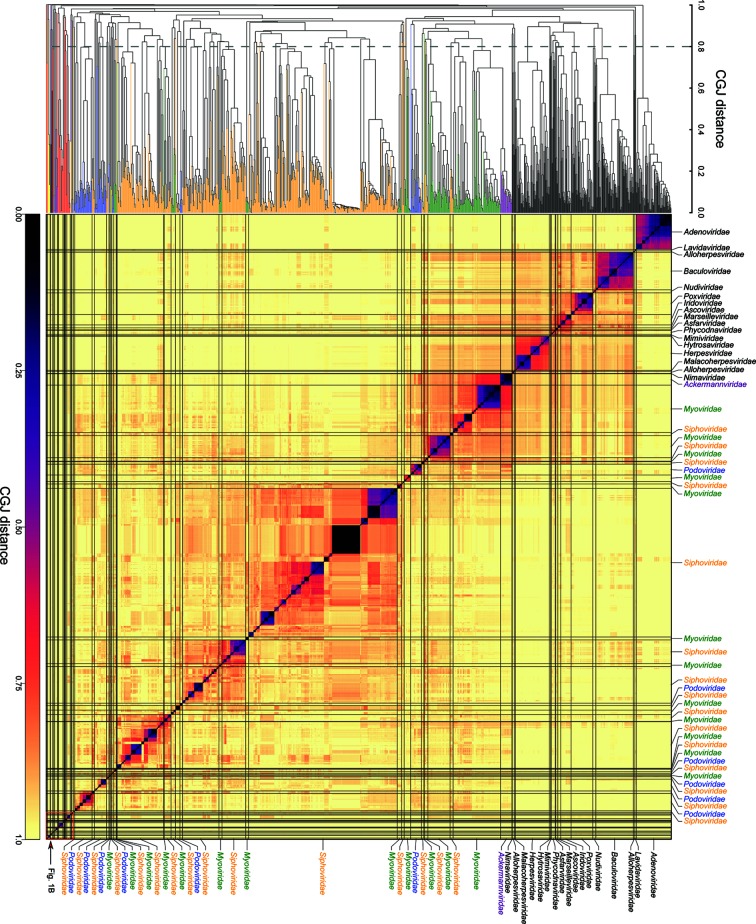

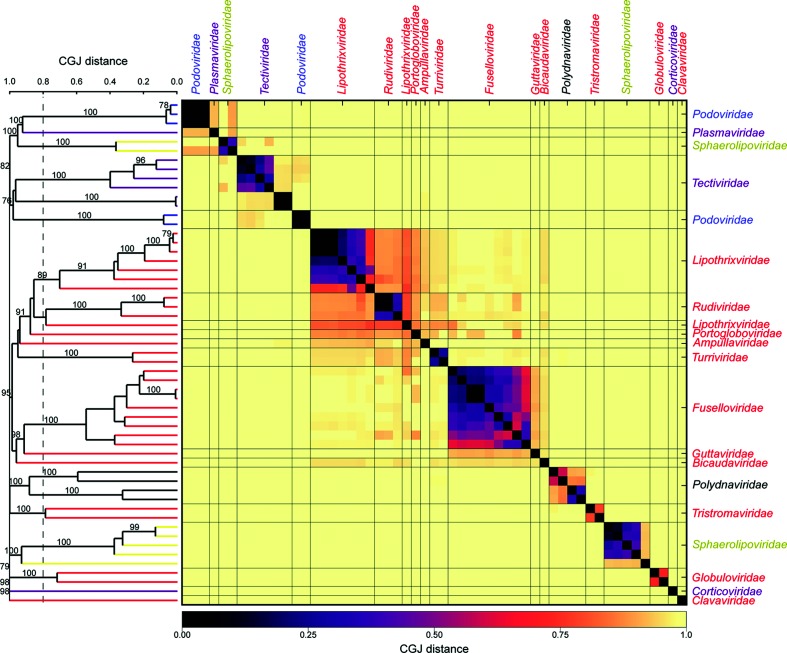
(a) Heat map and dendrogram of dsDNA viruses using pairwise composite generalized Jaccard (CGJ) distances. Branches and labels were colour coded by their hosts – *Siphoviridae*: orange; *Myoviridae*: blue; *Podoviridae*: green; other bacterial: purple; archaeal: red; dual host (archaea and bacteria): yellow; eukaryotic: black. The order of taxa in the heat map followed the phylogeny of the dendrogram and was not therefore constrained by ICTV family assignments. (b) Expanded view of the lower right-hand quadrant of the heat map and associated section of the dendrogram showing the genome relationships of archaeal viruses and other related viruses. Bootstrap support is shown above branches in the dendrogram (values of ≥70 % are shown). Taxa were colour coded as in Fig. 1(a).

**Fig. 2.**
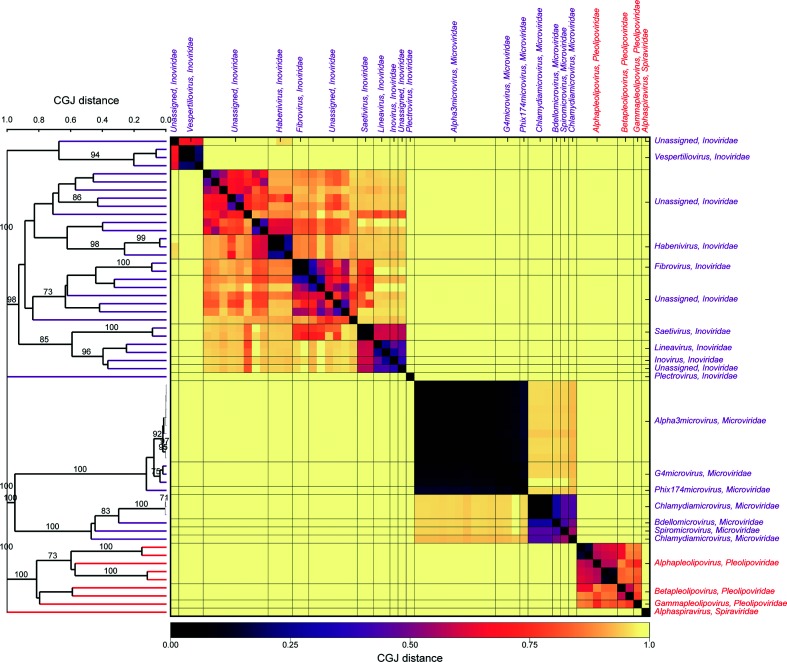
Genome relationships of bacterial and archaeal viruses in Baltimore group II. The heat map and dendrogram and associated bootstrap values are represented as in Fig. 1(b).

The genomes of 939 dsDNA viruses infecting Bacteria and Archaea were incorporated into the existing dataset of the classified eukaryotic virus in Baltimore group I and were analysed by GRAViTy (Figs 1, S1 and S2, available in the online version of this article). A similar re-analysis was performed on 59 ssDNA viruses (group II; Figs 2, S3 and S4), seven dsRNA and nine ss(+)RNA viruses (Baltimore groups III and IV, Figs S5, S6, S7 and S8, respectively) infecting bacteria and archaea in conjunction with the corresponding sets of eukaryotic viruses. See Table S1 for the datasets.

The inclusion of currently classified prokaryotic viruses vastly expanded the dataset of dsDNA viruses and created a large number of sequence clusters additional to those formed by eukaryotic viruses (Fig. 1). Sequence relationships between viruses were determined by their degrees of similarity to shared genes and genome organizations, and were plotted as a combined pairwise (dis)similarity heat map and dendrogram based on CGJ distances (Figs 1 and S1/S6). Their analysis by GRAViTy revealed an almost complete primary division of viruses into those infecting eukaryotes (black branches in the dendrogram, top left quadrant of the heat map) from different families of *Caudovirales* (green for *Myoviridae*, blue for *Podoviridae* and orange for *Siphoviridae*), other bacterial (purple) and archaeal viruses (red).

As reported previously [30], eukaryotic virus families fell into monophyletic family clades with a higher level grouping between families of several large dsDNA viruses. This was not the case for the three families within the *Caudovirales* order, *Siphoviridae*, *Myoviridae* and *Podoviridae*, each of which showed multiple, highly divergent lineages that were often highly interspersed with each other on the dendrogram. While the majority of prokaryotic viruses showed no identifiable homology with eukaryotic viruses, a number of separate groups of *Siphoviridae* and *Myoviridae,* and the newly designated bacterial virus family *Ackermannviridae,* formed a larger grouping with the previously identified group of large DNA viruses of eukaryotes [30]. This genetic linkage originated through their shared possession of conserved ribonucleotide reductase and DNA polymerase gene protein profile hidden Markov model (PPHMM) profiles (Tables S2 and S3).

**Fig. 3.**
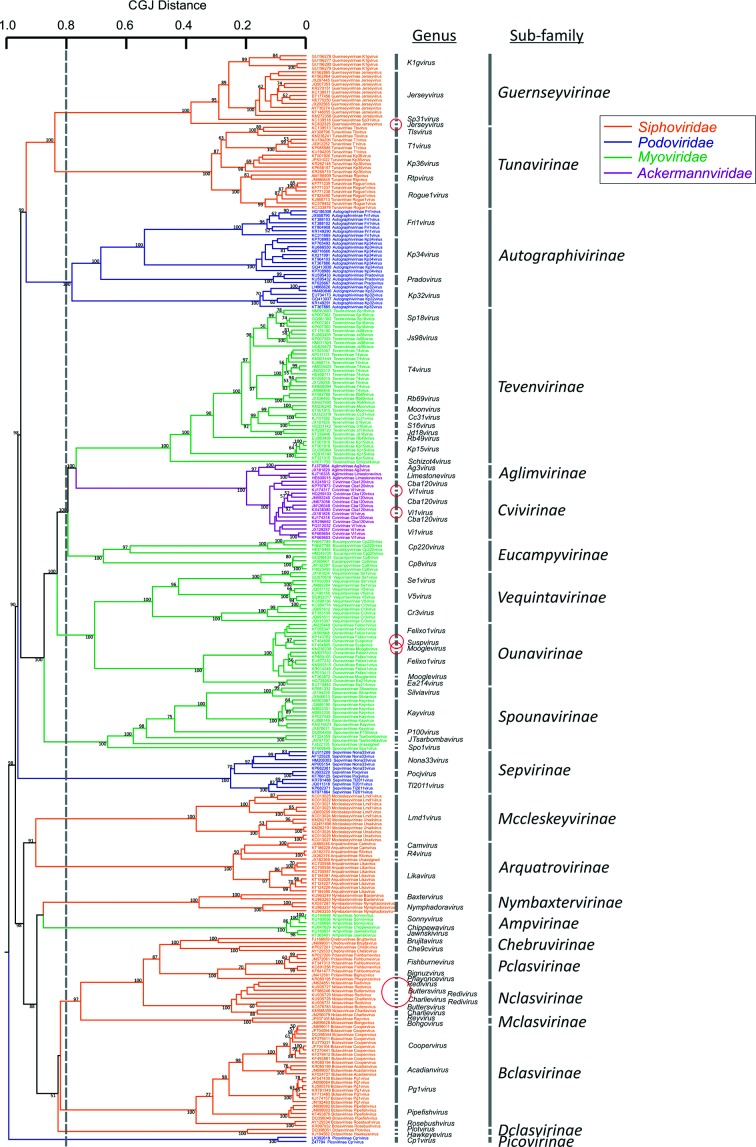
Dendrogram of members of *Caudovirales* that have subfamily assignments. Taxa were colour coded for family as in Fig. 1(a) (see the key). Minor discrepancies between genus assignments and phylogeny are shown in red circles. Bootstrap support is shown above branches in the dendrogram (values of ≥70 % are shown).

Many members of *Siphoviridae* formed a single bootstrap-supported cluster in the centre of the dendrogram/heat map, although this clade contained a large number (at least 11) of deep internal branches that were similarly divergent from each other as the eukaryotic virus families were from each other. Despite the polyphyly of *Siphoviridae*, *Myoviridae* and *Podoviridae* in *Caudovirales*, the recently established lower level taxonomy assignments of subfamily and genus of members of these families [33–35] were highly consistent with sequence relatedness estimated by GRAViTy (Fig. 3). Approximately one-third of *Caudovirales* has been assigned at the level of subfamily. Each of the 22 currently assigned subfamilies were clearly monophyletic in a dendrogram of CGJ distances, with deep branches between them. One cluster corresponds to the subfamily *Spounavirinae*, recently proposed as a new virus family *Herrelleviridae* [36], and the other example is the new family *Ackermannviridae,* whose members show a similar degree of inter-relatedness. The same analysis revealed that existing genus assignments within each subfamily (Fig. 3) or elsewhere (Figs S1 and S2) were supported, almost all forming genetically highly distinct clades of more closely related viruses. A small number of exceptions were identified (red rings in Fig. 3). There was a similar match between CGJ-distance-based groupings and genus assignments elsewhere in *Caudovirales*, the only identified exceptions being members of the genera *Phietavirus, Che8virus* and *L5virus* that contained members that were more divergent from each other than typically observed between members of other bacterial virus genera. Conversely, members of *Plotvirus* and *Pbi1virus* were almost identical to each other.

Each of the currently assigned archaeal virus families were highly divergent from each other and, when two or more sequences were available, monophyletic (Fig. 1b), consistent with previous genetic analyses [37]. The only exception was the separation of the *Gammalipothrixvirus* genus of *Lipothrixviridae*, which instead showed a closer, but non-bootstrap supported, affinity with *Rudiviridae*. Other taxa represented in this quadrant of the heat map were the bacterial virus families *Tectiviridae,* comprising two highly divergent clades corresponding to the *Alpha-* and *Betatectivirus* genera, *Plasmaviridae, Corticoviridae,* and two isolated groups of *Podoviridae,* corresponding to the genera *Cp1virus* and *Una961virus.* Members of the dual tropic family, *Sphaerolipoviridae* infecting bacteria (genus *Gammasphaerolipovirus*) grouped entirely separately from the *Alphasphaerolipoviridae* and *Betasphaeroliovirida*e genera that infect archaea. Amongst archaeal viruses, there was little evidence for any higher order relationships between different family-level groupings except for a distant linkage of the archaeal virus families *Rudi-, Lipothrix-, Turri-, Fusello-, Ampulla-, Portoglobo-* and *Guttaviridae* that formed a monophyletic group with 95 % bootstrap support. This higher level grouping incorporates the order *Ligamenvirales* that comprises the *Lipothrixviridae* and *Rudiviridae* families [38].

A small proportion of bacterial and archaeal viruses possessed alternative genome configurations, and were analysed in conjunction with eukaryotic viruses in Baltimore group II (*Inoviridae*, *Microviridae, Pleolipoviridae* and *Spiraviridae*; ssDNA, Figs 2, S3 and S4), group III (*Cystoviridae*; dsRNA; Figs S5 and S6) and group IV (*Leviviridae*; ss(+)RNA; Figs S7 and S8). The latter two RNA virus families were both monophyletic and showed no detectable relatedness to other RNA viruses in their Baltimore classes. In group II, bacterial and archaeal virus divided into six unrelated clades. Three of these corresponded to the families *Microviridae*, *Pleolipoviridae* and *Spiraviridae,* forming bootstrap-supported, although often highly divergent, monophyletic groups. However, members of the *Inoviridae* divided into three groups without detectable relatedness between them or other ssDNA viruses: the *Plectrovirus* genus, the *Vespertiliovirus* genus and the third diverse group comprising the genera *Saetivirus, Lineavirus* and *Inovirus* in one clade, deeply divided from the group formed from *Fibrovirus, Habenivirus,* and several other currently unassigned potential genera (Fig. 2).

### CGJ distances between bacterial and archaeal virus groups

The polyphyletic nature of several bacterial virus groups, including members of *Caudovirales, Sphaerolipoviridae*, *Tectiviridae* and *Inoviridae,* and of the archaeal virus family *Lipothrixviridae,* and their generally very deeply branched dendrograms indicate that the current assignment criteria for the prokaryotic virus family (and order) have created groups that are frequently quite different in genomic diversity than those of eukaryotic viruses.

**Fig. 4.**
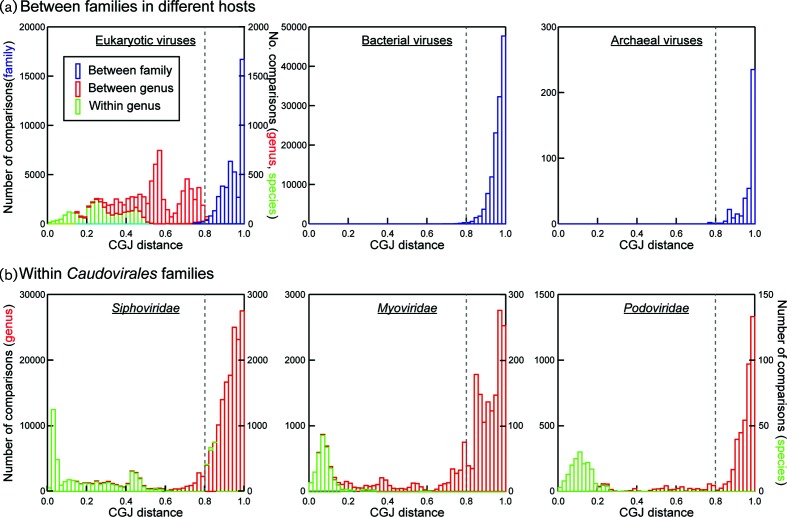
(a) Sets of pairwise composite generalized Jaccard (CGJ) distances between viral sequences of classified members of eukaryotic, bacterial and archaeal virus families. Blue bars represent the totals of pairwise comparisons (left-hand y-axis) over 0.02 distance intervals. For eukaryotic viruses, distances between genera (red bars) and within genera (green bars) were shown using the right-hand y-axis scale. (b) A separate analysis of sequences of members of the *Siphoviridae, Podoviridae* and *Myoviridae* families in *Caudovirales*, showing sets of pairwise distances between different genera (red) and within genera (green).

To investigate this systematically, the distributions of pairwise CGJ distances between members of different eukaryotic, bacterial and archaeal dsDNA virus families (Fig. 4a) were compared with those within members of individual bacterial virus families in *Caudovirales* (Fig. 4b). In general, almost all pairwise distances between eukaryotic virus families were constrained to a range between 0.8 and 1 (where 0=identical; 1=no detectable sequence relatedness), with only 1.1 % of pairwise comparisons (480/44367) falling below this informal threshold. Distributions of pairwise distances between bacterial virus families and between archaeal virus families were similarly constrained to distances above 0.8, although with an overall rightward shift to higher CGJ distances compared to eukaryotic viruses.

**Fig. 5.**
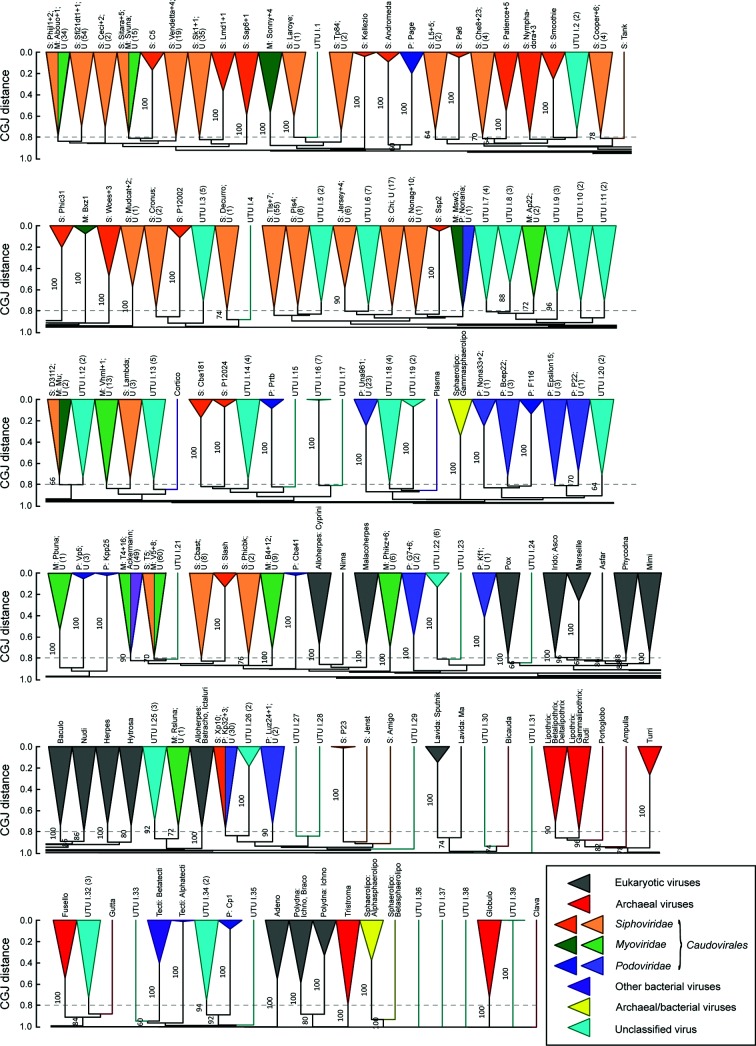
Dendrogram of classified and unclassified dsDNA viruses (Baltimore group I) based on composite generalised Jaccard (CGJ) distances, divided into six separate lines to represent the 139 clades present in the dataset. Tips are labelled with genus for members of *Caudovirales* (abbreviated as S: *Siphoviridae*; M: *Myoviridae*; P: *Podoviridae*), with family/genus for other bacterial, eukaryotic and archaeal viruses or with accession number codes for unclassified viruses. The scale bar for CGJ distance is shown at the left of each line and the 0.8 threshold that corresponds to eukaryotic family groupings is shown as a grey dotted line. Bootstrap re-sampling was performed with pruned signature tables as previously described [30]. Clades were coloured based on host origin according to the key; those containing both classified and unclassified sequences were shown in a lighter shade. The 39 new candidate unassigned taxonomic units (UTUs) arising from the inclusion of current unclassified viruses are shaded in light blue. Bootstrap support is shown above branches in the dendrogram (values of ≥70 % are shown).

Remarkably, distances within tailed phage families (*Podoviridae, Siphoviridae* and *Myovoridae*) (Fig. 4b) showed a comparable distribution to those between eukaryotic virus families (Fig. 4a, left), with over 92 % of pairwise distances being above the 0.8 distance threshold that divides (approximately) eukaryotic virus families from each other. Although not proposed as a classification threshold for prokaryotic viruses and more as a metric to evaluate comparative viral diversity, the same 0.8 CGJ distance threshold that divides eukaryotic virus families would split prokaryotic viruses into a total of 70 equivalent genetic groupings within *Caudovirales* (Figs 5, S9 and S10).

The genetic inter-relationships of eukaryotic viruses were in fact much more comparable to those of subfamilies currently assigned for some members of the three families within *Caudovirales.* The 0.8 threshold divides viruses remarkably consistently into the groups assigned subfamily status (Fig. 3), the main exception being the closer relatedness of the siphovirus subfamilies, *Chebruviriniae, Pclasvirinae* and *Nclasviriae.* A similar splitting of families using the 0.8 threshold occurred in other bacterial and archaeal dsDNA viruses – *Sphaerolipoviridae* would divide into three family-equivalent groups, and both *Tectiviridae* and *Lipothrixviridae* into two (Fig. 1b).

### Unclassified prokaryotic viruses

A total of 584 (near-) complete genomes of currently unclassified bacterial and archaeal viruses were obtained from the GenBank database (Table S4). These viral sequences were mostly generated from environmental sampling and therefore lacked information on morphology that would otherwise have allowed their provisional assignment into currently defined phage families. Of these, 580 sequences exhibited greatest similarity to members of Baltimore group I (Table S5). A dendrogram of the unclassified sequences and assigned bacterial, archaeal and eukaryotic viruses (Figs 5, S9 and S10) revealed the existence of 146 separate genetic clusters defined by the 0.8 cut-off value (grey dotted line in Fig. 5) and of these, 91 unclassified viruses grouped into 39 novel clades (shaded in light blue; see Table S6 for lists of sequences in each cluster).

Of the remaining four unclassified virus genome sequences, two (KX181651 and KY853667 – Xanthomonas phage Xf109 and Xf409, respectively) showed the greatest similarity to members of the family *Inoviridae*; KX344510 was a member of *Pleolipoviridae,* and MG065683 (Campylobacter phage B14) showed no similarity to any sequence in the classified dataset.

## Discussion

This study used a relatively simple metric of genetic relatedness between viral genomes, computed from a combination of similarity of gene complements and genome organization, based on gene synteny and gene orientation. We have previously shown that despite its simplicity, this method could be used to largely recapitulate the family relationships of eukaryotic viruses as well as to predict the existence of a large number of potential new families from analysis of metagenomic sequence data [30]. However, CGJ distances represent a single measure of genetic relatedness between viruses, and GRAViTy is necessarily limited in its ability to depict more complex virus relationships in which different genes with a viral genome are mosaic-containing genes with different evolutionary histories. Network and bipartite methods [26, 27, 39], in which virus relationships are depicted through their possession of shared genes, are potentially more effective in this regard, and are particularly relevant for certain groups of bacterial viruses among which horizontal gene transfer is widespread [40]. Nevertheless, for the purposes of the current study, condensing genetic relatedness to effectively a single number, the CGJ distance by GRAViTy, provides a straightforward and intuitive depiction of virus relatedness using dendrograms and heat maps. Furthermore, this relatively simpler monopartite technique provides a common methodology with which degrees of virus diversity can be directly compared within virus orders, families and genera regardless of their host. The use of a common benchmark would allow a unified approach to guide taxonomic assignments.

### Virus classification levels in eukaryotic, bacterial and archaeal viruses

The principle aim of the current study was to investigate the comparability of virus classification methods applied to different virus groups. Particularly relevant is the extent to which the primary division of eukaryotic viruses into families represents a common ranking that is shared in the classification of prokaryotic viruses. If criteria for taxonomic assignments are different between virus groups, then programs such as GRAViTy, trained on eukaryotic viruses, or vConTACT that classifies prokaryotic viruses [27], would be compromised in their abilities for the wider classification of sequence datasets of viruses where the nature of their hosts may be entirely unknown. For environmental sequence datasets, the existence of a common methodology and assignment criteria for taxonomic assignments is mandatory.

As depicted clearly in the current study, the application of a common genetics-based analysis of bacterial and archaeal viruses revealed a fundamental difference in taxonomic assignment levels to those used for eukaryotic viruses. Members of currently assigned prokaryotic virus families were typically far more divergent from each other than members of eukaryotic virus families at each level (Fig. 4). This is particularly evident among virus groups in the tailed page families, *Podoviridae, Siphoviridae* and *Myoviridae,* that formed polyphyletic groups (Figs 1, S1 and S2), as did several other bacterial and archaeal families with genera possessing no detectable genetic relatedness to each other (at least as determined by GRAViTy). These findings are consistent with the much earlier observation of the polyphyletic nature of these virus families in phage proteome trees [5]. This difference is similarly illustrated by distributions of pairwise CGJ distances (Fig. 4), where those between eukaryotic virus families matches those of distances between genera that are within individual families of the *Caudovirales.* The recent assignment of large numbers of *Caudovirales* to a series of subfamilies produces taxonomic groupings that better match typical inter-family distances observed between viruses infecting eukaryotes (Fig. 3).

### Morphology-based virus classification

The observed disparity in classification levels of viruses infecting different hosts is explained, at least in part, by the historical dependence on electron-microscopy and virion appearance as the primary classification criteria for making family-level assignments for bacterial and archaeal viruses [15]. In the pre-genomics age, morphology and some indication of host range were often the only information available with which to classify such viruses, in contrast to the relatively richer datasets of phenotypic and genetic information available for many human, animal and plant viruses. Nevertheless, virion morphology and the presence of conserved protein folds may represent stable metrics of evolutionary relatedness, particularly for viruses with complex sets of interdependent structural genes that produce the complex virion structures and DNA-packaging mechanisms of the tailed phages [41, 42]. These resemblances indeed point towards a shared evolutionary origin of many members of each family in *Caudovirales* that may not be recoverable from sequence comparisons alone.

Despite this, there remains considerable morphological variability within each of the tailed phage families. This is particularly evident among members of *Podoviridae* [43], in which their family membership is based on possession of a short ‘tail’, a feature that conceals the wide range of capsid shapes and sizes (icosahedral to tubular, 59–145 nm in length), as well as differences in genome lengths (16–77 Kbps) and organization (20–128 encoded genes or ORFs, colinear or ambisense) among members of this family. As indicated by CGJ distances (Figs 1, S1 and S2), genes of different members of *Podoviridae* often show very limited or no detectable sequence similarity, certainly none that would consistently group them into a higher level taxon that excludes other bacterial viruses. Morphological and genome variability is similarly apparent amongst siphoviruses and myoviruses, and between genera of other bacterial and archaeal viruses. *Inoviridae*, with its vastly different virion morphologies (>100×7 nm compared to 300×15 nm in the case of plectroviruses) and genome sizes (4.5 to more than 8.5 Kb, 4–11 genes), is a further striking example. Overall, the heterogeneity of prokaryotic viruses assigned to the same family is quite untypical of the variability found among members of eukaryotic virus families and is consistent with the much greater CGJ distances between members. It is therefore not surprising that sequence relationships as depicted by GRAViTy did not reproduce the close relationship between family membership and genetic divergence that we previously reported [30]. CGJ distance and grouping criteria that accurately reproduced eukaryotic family identifications appear to be quite different from those required to recapitulate existing family-level assignments of bacterial and archaeal viruses.

The morphological diversity of members of the proposed family, *Saltoviridae*, represents a complementary example of the disconnect between genetic relatedness and morphology [44]. Although representing a relatively closely related cluster of viruses with 55 % or greater overall amino acid sequences identity between members [45], they show considerable structural diversity – some members were previously classified as *Siphoviridae* and others as *Myoviridae.* This example of conflicts with genetic relatedness has contributed to the animated discussion of discrepancies between phylogeny and morphology for many years in the phage classification field [28].

An even more fundamental limitation of the current taxonomy of most bacterial and archaeal viruses is that morphology-based metrics, such as lengths and contractility that are used for assignments to the three families in *Caudovirales*, cannot be readily applied for classification of viruses that are only known from genome sequences within metagenomic datasets. Methods used for acquisition of sequences in environmental samples are currently unable to match assembled genome sequences to individually visualized virions (although conceivably this may not be a permanent technical limitation). While there has been some progress towards identification of structural gene modules in tailed phage genomes, their sequence relationships imperfectly reproduce their current assignments into three families [46] and, in general, bioinformatic and protein structure prediction methods cannot reliably predict family membership among *Caudovirales* as currently assigned. This limitation represents a fundamental barrier to the incorporation of metagenomically derived bacterial viruses into the ICTV classification. The development of genomics-based methods for incorporation of the vast number of metagenomically derived viruses [13, 21, 22] is therefore essential for the long-term integrity of a coherent and unified ICTV taxonomy [32].

### Steps towards a unified virus taxonomy

The ICTV is committed to the incorporation of viruses known only by their genome sequence into the current taxonomy in a manner that is as consistent as possible with the existing framework [32, 47]. However, as described, this will require a united system for taxonomy assignments of viruses infecting all three domains of life – one cannot maintain separate eukaryotic and bacterial/archaeal frameworks where hosts may often be entirely unknown. Secondly, to be of practical utility, taxonomic assignment criteria have to be reliably recoverable from genetic/bioinformatic information of virus genome sequences up to the family level – this represents the highest classification level mandated by the ICTV and represents an important functional and genetic division of viruses in the current taxonomy framework.

Deeper evolutionary relationships that might link viruses at higher taxonomic levels, such as order, class or phylum are conceptually valuable but not required by the ICTV. Indeed, virus relationships above the family level of eukaryotic viruses are often quite beyond the capabilities of current bioinformatic or evolutionary analysis methods to recover. However, future developments in protein structure prediction and greater understanding of protein evolution may ultimately allow these deeper relationships to be discovered and used for taxonomic purposes.

In the immediate future, discussion and reconciliation of differences in the criteria used for family assignment of viruses infecting different hosts will be important. While the identification of a CGJ distance threshold of 0.8 effectively divides eukaryotic virus families and genera, its appropriateness for bacterial and archaeal family definition requires considerable further investigation and parallel evaluation of other metrics produced by other methods, such as vConTACT [27] and future development of more informative bipartite methodologies [26, 39, 48]. Encouragingly, the subfamily taxonomic level of *Caudovirales* can be readily identified genetically and members shows CGJ distances in the range of inter-family distances of eukaryotic viruses. The ongoing re-evaluation of these subfamilies [36], the proposed introduction of new families such as ‘*Saltoviridae*’ [44], ‘*Lambdaviridae*’ [23] and ‘*Herrelleviridae*’ [36] and the current debate within the bacterial virus community to abolish the *Myoviridae, Podoviridae* and *Siphoviridae* families would indeed create a taxonomy far more compatible with that of eukaryotic viruses. This, in turn, should lead to a more appropriate context for taxonomic assignment of metagenomic sequences and allow a shift of focus to address the remaining unclassified bacterial viruses in the database.

The convergence of interest from virologists working with quite different virus groups and these recent methodological developments in quantifying virus sequence relationships potentially create the conditions for a step change in how viruses are classified in the future. Productive collaborative action to develop an all-encompassing, effective and pragmatic classification scheme for the global virome may not be too far in the future.

## Methods

### Reference datasets

Annotated prokaryotic viral genomes and their associated information were compiled from (i) the ICTV 2016 Master Species List 31V1.1 (MSL; https://talk.ictvonline.org/files/master-species-lists/), (ii) Virus Metadata Resource (https://talk.ictvonline.org/taxonomy/vmr/) and (iii) newly assigned viruses from the ICTV Executive Committee meeting, Singapore, 2017, now formally ratified. The associated information includes (i) Baltimore classification group, (ii) order assignment, (iii) family assignment, (iv) subfamily assignment, (v) genus assignment, (vi) virus name, (vii) GenBank accession number, (viii) RefSeq accession number, (ix) sequence description and (x) host group. In our previous work [30], we compiled eukaryotic virus genomes from various databases, and they were also included in this study as reference genomes. Together, the dataset comprised 4215 [prokaryotic virus (P): 1014; eukaryotic virus (E): 3201] whole genomic virus records, sampled across four Baltimore groups, six (P : 2; E : 4) orders, 99 (P : 25; E : 74) families and 634 (P : 265; E : 369) genera (Table S1). Taxonomic assignments followed those of the ICTV MSL, and for an extended list of viruses in the Virus Metadata Resource and RefSeq databases.

### Estimating sequence relatedness among reference viruses infecting Bacteria, Archaea and Eucarya using GRAViTy

We estimated genetic relatedness among viruses in each Baltimore group using GRAViTy (GitHub: PAiewsakun/GRAViTy) [30]. Briefly, protein sequences were extracted from virus genomes, and were clustered based on blastp pairwise sequence similarity scores [49]. Sequences in each cluster were then aligned and turned into a protein profile hidden Markov model (PPHMM). PPHMMs that show similarity to only one virus were excluded from the database if the family of that particular virus contains more than two viruses. Genomes were subsequently scanned against the PPHMM database to locate their genes, and these gene location profiles were used to build genomic organization models (GOMs), one for each virus family. These PPHMM and GOM databases formed the central part of the GRAViTy genome annotators.

To estimate sequence relatedness, each genome was scanned against the annotator, and was annotated with a PPHMM and a GOM signature. A PPHMM signature is a list of similarity scores to each of the PPHMMs, and a GOM signature is a list of distance correlations (GOM scores) between its gene location profile and each GOM specific to each of the virus families. Composite generalized Jaccard (CGJ) similarity scores between each sequence pair were then computed based on their PPHMM and GOM signatures. A CGJ score is a geometric mean of the two generalized Jaccard scores computed for a pair of PPHMM signatures and a pair of GOM signatures. Pairwise CGJ distances, which are equal to 1−CGJ, were subsequently computed, and these were used in the construction of the heat map and dendrogram depicting how viruses relate to one another. The dendrogram was estimated using the unweighted pair group method with arithmetic mean (UPGMA). To assess the uncertainty of the observed sequence relatedness, we use the bootstrapping technique with 50–100 pseudoreplicate samples. Four heat maps and UPGMA dendrograms were estimated in total, one for each Baltimore virus group (Figs 1a and S1–S8).

Moreover, sequence relatedness within several virus subgroups were further investigated using the GRAViTy framework. These include (i) group I archaeal viruses and several other viruses that show detectable similarity with them (Fig. 1b), (ii) group II prokaryotic viruses (Fig. 2) and (iii) virus members of the *Caudovirales* order with subfamily assignments (Fig. 3). In these analyses, the viruses’ PPHMM signatures were subsampled to exclude gene features of which the PPHMM scores were zero for all viruses. Their GOM signatures were also subsampled such that the GOM scores that did not pertain to the investigated virus families were excluded. The bootstrapping technique with 100 pseudoreplicate samples was used to assess the uncertainty of the observed sequence relatedness.

In addition, the distributions of pairwise inter-family CGJ distances of group I eukaryotic, bacterial and archaeal viruses were examined to assess if there was a common distance threshold that could delimit virus families. Furthermore, we also inspected inter- and intra-genus CGJ distances of eukaryotic viruses and of the members of the *Siphoviridae*, *Podoviridae* and *Myoviridae* families in the *Caudovirales* order. The results are shown in Fig. 4.

### Shared genes between group I bacteriophages and dsDNA eukaryotic viruses

Our results show that eukaryotic dsDNA viruses exhibit some detectable similarity with specific groups of group I bacteriophages, including 48 genera of *Myoviridae*, eight genera of *Podoviridae,* five genera of *Siphoviridae*, and the entire newly established family *Ackermannviridae* (Figs 1, S1 and S2, Table S2). To determine the genes that are shared among these groups of viruses, they were grouped together to the exclusion of the rest of group I dsDNA viruses, and mutual information (MI) scores were computed for each gene feature, measuring the dependence between this binary virus grouping and their PPHMM scores. PPHMMs that did not exhibit any similarity to any of these viruses were excluded from this analysis. We noted that the MI calculation is stochastic, and the sample size per group can affect the calculation. Thus, the mean values computed from 100 estimates were used in the result interpretation, and within each of the 100 instances, only two viruses were sampled from each of the two taxonomic groups. The results are shown in Table S3.

### Estimating sequence relatedness among unclassified bacteriophages and archaeal viruses and reference viruses using GRAViTy

The list of unclassified bacteriophages with complete genomes was compiled from the NCBI database using the search terms [*‘unclassified bacterial viruses’ [Organism] NOT (‘Bacteria’ [Organism] OR ‘Bacteria Latreille et al. 1825’ [Organism]) AND ‘complete genome’ [All Fields]*]. Similarly, the list of unclassified archaeal viruses with complete genomes was retrieved using the search terms (*‘unclassified archaeal viruses’* [*Organism*] *NOT ‘Archaea’ [Organism] AND ‘complete genome’* [*All Fields]*). Only those from the GenBank database were collected. In total, the list comprised 576 whole genomes of unclassified bacterial viruses, and eight unclassified archaeal viruses (Table S4). The search was performed in February 2018. The genome of unclassified bacteriophages and archaeal viruses were run through the GRAViTy pipeline to identify which virus groups they might belong to. Our results (Table S5) show that 580 of the unclassified bacteriophages (573 viruses) and archaeal viruses (seven viruses) exhibit some similarity to group I dsDNA. A bootstrapped dendrogram depicting relationship among these unclassified viruses and the classified dsDNA viruses is shown in Figs 5, S9 and S10. In Fig. 5, the dendrogram was collapsed at the height equal to 0.8. The list of sequences in each clade can be found in Table S6.

## Supplementary Data

Supplementary File 1

Supplementary File 2
