# Supplementary material for: Evaluation of the genomic diversity of viruses infecting bacteria, archaea and eukaryotes using a common bioinformatic platform: steps towards a unified taxonomy
Source: J Gen Virol. 2018 Jul 17;99(9):1331–43. doi: 10.1099/jgv.0.001110 (PMC6230767; doi:10.1099/jgv.0.001110)
Supplement: Supplementary File 1 [file jgv-99-1331-s001.pdf]

Figure S1 UPGMA dendrogram of Group I dsDNA viruses (NEWICK format)

((((((((((((((((((((KC693023_Adenoviridae_Mastadenovirus:0.019628,KC
693022_Adenoviridae_Mastadenovirus:0.019628):0.004722,KP329565_Adenovi
ridae_Mastadenovirus:0.02435)96:0.014091,AY771780_Adenoviridae_Mastade
novirus:0.038442)85:0.01828,L19443_Adenoviridae_Mastadenovirus:0.05672
2)88:0.02477,(((KC693021_Adenoviridae_Mastadenovirus:0.013048,HQ241819
_Adenoviridae_Mastadenovirus:0.013048)99:0.017847,KP329561_Adenovirida
e_Mastadenovirus:0.030894)95:0.018988,KP329564_Adenoviridae_Mastadenov
irus:0.049882)94:0.03161):0.015892,X73487_Adenoviridae_Mastadenovirus:
0.097384):0.008741,(FJ025931_Adenoviridae_Mastadenovirus:0.078951,J019
17_Adenoviridae_Mastadenovirus:0.078951):0.027174):0.013323,(((HQ60591
2_Adenoviridae_Mastadenovirus:0.027068,AY598782_Adenoviridae_Mastadeno
virus:0.027068)100:0.055638,KX505867_Adenoviridae_Mastadenovirus:0.082
706)77:0.030815,(KM190146_Adenoviridae_Mastadenovirus:0.026132,KP32956
3_Adenoviridae_Mastadenovirus:0.026132)100:0.087389):0.005928):0.01792
,(((AY163756_Adenoviridae_Mastadenovirus:0.034525,DQ086466_Adenovirida
e_Mastadenovirus:0.034525)99:0.034484,(AY487947_Adenoviridae_Mastadeno
virus:0.021777,JN254802_Adenoviridae_Mastadenovirus:0.021777)100:0.047
232)74:0.023368,(AJ854486_Adenoviridae_Mastadenovirus:0.022803,AB33380
1_Adenoviridae_Mastadenovirus:0.022803)100:0.069574)89:0.044991):0.013
78,HQ913600_Adenoviridae_Mastadenovirus:0.151148)99:0.090939,(((((((K
T698852_Adenoviridae_Mastadenovirus:0.034715,KT698856_Adenoviridae_Mas
tadenovirus:0.034715)100:0.111598,KJ563221_Adenoviridae_Mastadenovirus
:0.146313):0.011694,KT160425_Adenoviridae_Mastadenovirus:0.158006):0.0
13377,AF030154_Adenoviridae_Mastadenovirus:0.171384):0.017017,((AF2892
62_Adenoviridae_Mastadenovirus:0.128599,AC_000191_Adenoviridae_Mastade
novirus:0.128599):0.01277,(AC_000001_Adenoviridae_Mastadenovirus:0.0,A
F252854_Adenoviridae_Mastadenovirus:0.0)100:0.141369):0.047032):0.0120
04,AC_000190_Adenoviridae_Mastadenovirus:0.200405):0.006701,AC_000189_
Adenoviridae_Mastadenovirus:0.207106):0.020484,(((((GU226970_Adenoviri
dae_Mastadenovirus:0.034847,KX871230_Adenoviridae_Mastadenovirus:0.034
847)82:0.013163,JN252129_Adenoviridae_Mastadenovirus:0.048009):0.00171
3,KP238322_Adenoviridae_Mastadenovirus:0.049722)75:0.015492,Y07760_Ade
noviridae_Mastadenovirus:0.065214)99:0.050478,JN418926_Adenoviridae_Ma
stadenovirus:0.115692)100:0.111898):0.014497):0.039642,((KT698855_Aden
oviridae_Mastadenovirus:0.008762,KT698854_Adenoviridae_Mastadenovirus:
0.008762)85:0.006986,KT698853_Adenoviridae_Mastadenovirus:0.015747)100
:0.265981)84:0.047717,(EU835513_Adenoviridae_Mastadenovirus:0.241964,H
M049560_Adenoviridae_Mastadenovirus:0.241964)98:0.087481)100:0.15471,(
(((((AF036092_Adenoviridae_Atadenovirus:0.047463,JQ345700_Adenoviridae
_Atadenovirus:0.047463)89:0.015669,U40839_Adenoviridae_Atadenovirus:0.
063132)100:0.159691,Y09598_Adenoviridae_Atadenovirus:0.222823):0.01328
8,(DQ106414_Adenoviridae_Atadenovirus:0.134337,KJ156523_Adenoviridae_A
tadenovirus:0.134337)96:0.101774):0.035444,KJ675568_Adenoviridae_Atade
novirus:0.271555)100:0.14802,((((HM585353_Adenoviridae_Siadenovirus:0.
034297,EU715130_Adenoviridae_Siadenovirus:0.034297)80:0.029026,KP14432
9_Adenoviridae_Siadenovirus:0.063323):0.007833,(AC_000016_Adenoviridae
_Siadenovirus:0.0,AF074946_Adenoviridae_Siadenovirus:0.0)100:0.071156)
90:0.061186,AF224336_Adenoviridae_Siadenovirus:0.132342)100:0.287233)9
3:0.06458)98:0.06589,((((((GU734104_Adenoviridae_Aviadenovirus:0.10028
1,AF083975_Adenoviridae_Aviadenovirus:0.100281)100:0.093222,GU936707_A

denoviridae_Aviadenovirus:0.193502):0.018967,((KF477312_Adenoviridae_A
viadenovirus:0.042412,KC493646_Adenoviridae_Aviadenovirus:0.042412)100
:0.119907,GU188428_Adenoviridae_Aviadenovirus:0.162318):0.050151):0.01
3073,(KF477313_Adenoviridae_Aviadenovirus:0.121578,U46933_Adenoviridae
_Aviadenovirus:0.121578)100:0.103964):0.032616,(FN824512_Adenoviridae_
Aviadenovirus:0.14316,KX121164_Adenoviridae_Aviadenovirus:0.14316)93:0
.114998):0.030392,(JF510462_Adenoviridae_Aviadenovirus:0.095786,KJ4696
53_Adenoviridae_Aviadenovirus:0.095786)100:0.192765)100:0.261494)100:0
.445662,((((HG531932_Lavidaviridae_Sputnikvirus:0.147222,EU606015_Lavi
daviridae_Sputnikvirus:0.147222)100:0.704516,HQ712116_Lavidaviridae_Ma
virus:0.851738)85:0.135709,((((DQ665652_Alloherpesviridae_Batrachoviru
s:0.58748,DQ665917_Alloherpesviridae_Batrachovirus:0.58748)100:0.16758
8,M75136_Alloherpesviridae_Ictalurivirus:0.755068)100:0.217691,(((((((
((((((((((KM102981_Baculoviridae_Alphabaculovirus:0.092119,EU839994_B
aculoviridae_Alphabaculovirus:0.092119)100:0.029194,DQ123841_Baculovir
idae_Alphabaculovirus:0.121313)100:0.049175,KM009991_Baculoviridae_Alp
habaculovirus:0.170488):0.017873,((EU780426_Baculoviridae_Alphabaculov
irus:0.098327,AF169823_Baculoviridae_Alphabaculovirus:0.098327)100:0.0
53924,EF035042_Baculoviridae_Alphabaculovirus:0.152251)87:0.036109):0.
026082,((AY126275_Baculoviridae_Alphabaculovirus:0.044723,JQ798165_Bac
uloviridae_Alphabaculovirus:0.044723)95:0.016454,U59461_Baculoviridae_
Alphabaculovirus:0.061176)100:0.153266)100:0.057898,((DQ017380_Baculov
iridae_Alphabaculovirus:0.098884,KJ631622_Baculoviridae_Alphabaculovir
us:0.098884)86:0.018347,AY864330_Baculoviridae_Alphabaculovirus:0.1172
31)100:0.15511)98:0.044386,(((((KF158713_Baculoviridae_Alphabaculoviru
s:0.189526,FJ227128_Baculoviridae_Alphabaculovirus:0.189526)76:0.01514
4,EU309041_Baculoviridae_Alphabaculovirus:0.20467)76:0.022678,DQ837165
_Baculoviridae_Alphabaculovirus:0.227348):0.007261,KM596836_Baculoviri
dae_Unassigned:0.234609)98:0.038991,(((KJ676450_Baculoviridae_Alphabac
ulovirus:0.175763,KF611977_Baculoviridae_Alphabaculovirus:0.175763)81:
0.031926,FJ914221_Baculoviridae_Alphabaculovirus:0.207689)98:0.036498,
DQ504428_Baculoviridae_Alphabaculovirus:0.244187)96:0.029412)93:0.0431
27):0.037026,KP752043_Baculoviridae_Alphabaculovirus:0.353752):0.01114
9,((((EU591746_Baculoviridae_Alphabaculovirus:0.027863,AP006270_Baculo
viridae_Alphabaculovirus:0.027863)100:0.291333,(AF303045_Baculoviridae
_Alphabaculovirus:0.014713,AF271059_Baculoviridae_Alphabaculovirus:0.0
14713)100:0.304483):0.01618,KR011717_Baculoviridae_Unassigned:0.335376
):0.008514,AY394490_Baculoviridae_Alphabaculovirus:0.34389):0.021011):
0.028831,(GQ202541_Baculoviridae_Alphabaculovirus:0.128165,AF081810_Ba
culoviridae_Alphabaculovirus:0.128165)100:0.265567):0.005858,(AF325155
_Baculoviridae_Alphabaculovirus:0.071221,JX454574_Baculoviridae_Alphab
aculovirus:0.071221)100:0.328369)100:0.04749,((((((((KC961304_Baculovi
ridae_Alphabaculovirus:0.072522,AF512031_Baculoviridae_Alphabaculoviru
s:0.072522)75:0.021725,KF894742_Baculoviridae_Alphabaculovirus:0.09424
6)87:0.028256,AY043265_Baculoviridae_Alphabaculovirus:0.122502):0.0106
45,U75930_Baculoviridae_Alphabaculovirus:0.133148):0.004427,AP009046_B
aculoviridae_Alphabaculovirus:0.137574):0.019111,DQ486030_Baculovirida
e_Alphabaculovirus:0.156686):0.031182,(((KR815466_Baculoviridae_Alphab
aculovirus:0.020208,DQ813662_Baculoviridae_Alphabaculovirus:0.020208)1
00:0.070785,AY327402_Baculoviridae_Alphabaculovirus:0.090993):0.016385
,KJ631623_Baculoviridae_Alphabaculovirus:0.107378)88:0.08049)100:0.058
493,(((JX467702_Baculoviridae_Alphabaculovirus:0.080259,L22858_Baculov

iridae_Alphabaculovirus:0.080259)99:0.03297,L33180_Baculoviridae_Alpha
baculovirus:0.113229)99:0.06976,(EF125867_Baculoviridae_Alphabaculovir
us:0.145976,KU565883_Baculoviridae_Alphabaculovirus:0.145976):0.037014
)100:0.063372)100:0.200718)100:0.120505,((((((((((KJ406702_Baculovir
idae_Betabaculovirus:0.090676,KR091910_Baculoviridae_Betabaculovirus:0
.090676)100:0.08398,DQ333351_Baculoviridae_Betabaculovirus:0.174656):0
.016413,GQ884143_Baculoviridae_Betabaculovirus:0.191069):0.032501,(U53
466_Baculoviridae_Betabaculovirus:0.166656,AY229987_Baculoviridae_Beta
baculovirus:0.166656)97:0.056914):0.014006,(KC179784_Baculoviridae_Bet
abaculovirus:0.02341,HQ116624_Baculoviridae_Betabaculovirus:0.02341)10
0:0.214166):0.016748,((KX151395_Baculoviridae_Betabaculovirus:0.212217
,KP296186_Baculoviridae_Betabaculovirus:0.212217):0.013493,AF499596_Ba
culoviridae_Betabaculovirus:0.22571):0.028614):0.002003,AF547984_Bacul
oviridae_Betabaculovirus:0.256327):0.031143,JN408834_Baculoviridae_Bet
abaculovirus:0.28747):0.026332,AF270937_Baculoviridae_Betabaculovirus:
0.313802):0.01382,KU593505_Baculoviridae_Betabaculovirus:0.327622)80:0
.032156,(KR584663_Baculoviridae_Betabaculovirus:0.002399,AY522332_Bacu
loviridae_Betabaculovirus:0.002399)100:0.35738)99:0.074814,(((AF16222
1_Baculoviridae_Betabaculovirus:0.073969,EU255577_Baculoviridae_Betaba
culovirus:0.073969)100:0.048718,EU678671_Baculoviridae_Betabaculovirus
:0.122687)100:0.126621,(KM371112_Baculoviridae_Betabaculovirus:0.21793
9,KR011718_Baculoviridae_Betabaculovirus:0.217939)90:0.03137)100:0.095
657,DQ288858_Baculoviridae_Betabaculovirus:0.344965)99:0.089628)100:0.
132992)100:0.172048,(((AY349019_Baculoviridae_Gammabaculovirus:0.07320
1,DQ317692_Baculoviridae_Gammabaculovirus:0.073201)100:0.164441,AY4308
10_Baculoviridae_Gammabaculovirus:0.237641)100:0.470611,AF403738_Bacul
oviridae_Deltabaculovirus:0.708252)81:0.03138)100:0.146268,(((KM610234
_Nudiviridae_Unassigned:0.606902,KJ184318_Nudiviridae_Unassigned:0.606
902)100:0.113063,(EU747721_Nudiviridae_Alphanudivirus:0.52934,EF203088
_Nudiviridae_Alphanudivirus:0.52934)100:0.190625)100:0.069632,(AF45189
8_Nudiviridae_Betanudivirus:0.027459,JN418988_Nudiviridae_Betanudiviru
s:0.027459)100:0.762138)94:0.096304)90:0.022583,((((((((((((AY077832
_Poxviridae_Capripoxvirus:0.01467,AY077835_Poxviridae_Capripoxvirus:0.
01467)100:0.014537,AF325528_Poxviridae_Capripoxvirus:0.029207)100:0.11
5983,AF410153_Poxviridae_Suipoxvirus:0.14519):0.008031,(AY689437_Poxvi
ridae_Cervidpoxvirus:0.014892,AY689436_Poxviridae_Cervidpoxvirus:0.014
892)100:0.138329):0.008165,(AY386371_Poxviridae_Yatapoxvirus:0.073329,
AJ293568_Poxviridae_Yatapoxvirus:0.073329)100:0.088057)98:0.042478,(AF
170722_Poxviridae_Leporipoxvirus:0.049928,AF170726_Poxviridae_Leporipo
xvirus:0.049928)100:0.153936)100:0.076715,(HQ647181_Poxviridae_Unassig
ned:0.112433,KY094066_Poxviridae_Unassigned:0.112433)100:0.168146)100:
0.073595,((((((DQ437594_Poxviridae_Orthopoxvirus:0.072546,AF438165_Po
xviridae_Orthopoxvirus:0.072546)85:0.011597,AF012825_Poxviridae_Orthop
oxvirus:0.084143):0.007479,AF482758_Poxviridae_Orthopoxvirus:0.091622)
:0.009427,(X69198_Poxviridae_Orthopoxvirus:0.087319,AF380138_Poxvirida
e_Orthopoxvirus:0.087319):0.01373):0.007871,AY243312_Poxviridae_Orthop
oxvirus:0.10892):0.009752,((KU749311_Poxviridae_Orthopoxvirus:0.029153
,KU749310_Poxviridae_Orthopoxvirus:0.029153)100:0.041373,KP143769_Poxv
iridae_Orthopoxvirus:0.070526)99:0.048146)100:0.123358,HQ849551_Poxvir
idae_Orthopoxvirus:0.24203)100:0.112143)100:0.083248,((((KM502564_Poxv
iridae_Parapoxvirus:0.061786,AY386265_Poxviridae_Parapoxvirus:0.061786
)79:0.010888,AY386264_Poxviridae_Parapoxvirus:0.072674)97:0.022457,GQ3

29670_Poxviridae_Parapoxvirus:0.095131)100:0.248173,((HE601899_Poxviri
dae_Unassigned:0.268496,U60315_Poxviridae_Molluscipoxvirus:0.268496):0
.022251,KU980965_Poxviridae_Unassigned:0.290747)92:0.052558)100:0.0941
17)85:0.081312,DQ356948_Poxviridae_Crocodylidpoxvirus:0.518733)96:0.03
1165,((((KJ801920_Poxviridae_Avipoxvirus:0.086696,AF198100_Poxviridae_
Avipoxvirus:0.086696)95:0.02026,KJ859677_Poxviridae_Avipoxvirus:0.1069
56)100:0.077901,AY318871_Poxviridae_Avipoxvirus:0.184857)100:0.135831,
KP728110_Poxviridae_Avipoxvirus:0.320688)100:0.22921)100:0.134851,KT15
9937_Poxviridae_Unassigned:0.684749)100:0.085728,(((((HF679134_Poxviri
dae_Betaentomopoxvirus:0.231244,HF679131_Poxviridae_Betaentomopoxvirus
:0.231244)91:0.030438,AF250284_Poxviridae_Betaentomopoxvirus:0.261682)
75:0.020671,(HF679133_Poxviridae_Betaentomopoxvirus:0.094871,HF679132_
Poxviridae_Betaentomopoxvirus:0.094871)100:0.187482)100:0.124921,AP013
055_Poxviridae_Alphaentomopoxvirus:0.407274)88:0.064633,AF063866_Poxvi
ridae_Unassigned:0.471907)100:0.29857)100:0.096818,(((((((((((KT9898
84_Iridoviridae_Ranavirus:0.003613,JQ724856_Iridoviridae_Ranavirus:0.0
03613)100:0.040167,FJ433873_Iridoviridae_Ranavirus:0.04378)89:0.025317
,(KX574343_Iridoviridae_Unassigned:0.055416,KX353311_Iridoviridae_Rana
virus:0.055416)76:0.013682)95:0.033767,((AY548484_Iridoviridae_Ranavir
us:0.042972,KX185156_Iridoviridae_Ranavirus:0.042972)100:0.035509,AY15
0217_Iridoviridae_Ranavirus:0.078481):0.024383)100:0.217109,AY521625_I
ridoviridae_Ranavirus:0.319973)100:0.171347,(L63545_Iridoviridae_Lymph
ocystivirus:0.25867,AY380826_Iridoviridae_Lymphocystivirus:0.25867)100
:0.232649)85:0.060507,AF371960_Iridoviridae_Megalocytivirus:0.551827)1
00:0.114585,(((((HF920636_Iridoviridae_Iridovirus:0.046091,HF920634_I
ridoviridae_Iridovirus:0.046091)80:0.012331,HF920633_Iridoviridae_Irid
ovirus:0.058422)100:0.041139,(GQ918152_Iridoviridae_Iridovirus:0.07641
9,HF920635_Iridoviridae_Iridovirus:0.076419)98:0.023141)100:0.200261,(
KF938901_Iridoviridae_Unassigned:0.263133,DQ643392_Iridoviridae_Chlori
ridovirus:0.263133)72:0.036688)100:0.144797,(AF303741_Iridoviridae_Iri
dovirus:0.247604,HF920637_Iridoviridae_Iridovirus:0.247604)100:0.19701
4)100:0.153271,CU469068_Ascoviridae_Toursvirus:0.597889)100:0.068522)1
00:0.087498,((((JX491653_Ascoviridae_Ascovirus:0.068619,EF133465_Ascov
iridae_Ascovirus:0.068619):0.023333,KJ755191_Ascoviridae_Ascovirus:0.0
91952)100:0.269324,AM398843_Ascoviridae_Ascovirus:0.361275)100:0.14517
5,DQ517337_Ascoviridae_Ascovirus:0.506451)100:0.247459)97:0.04096,(((K
F483846_Marseilleviridae_Unassigned:0.139258,HQ113105_Marseilleviridae
_Unassigned:0.139258)100:0.060194,GU071086_Marseilleviridae_Marseillev
irus:0.199452)100:0.063612,KT835053_Marseilleviridae_Unassigned:0.2630
64)100:0.531805)92:0.021869,U18466_Asfarviridae_Asfivirus:0.816739):0.
009619,((((EU304328_Phycodnaviridae_Prasinovirus:0.640382,AJ890364_Phy
codnaviridae_Coccolithovirus:0.640382)72:0.06221,KX008963_Phycodnaviri
dae_Raphidovirus:0.702591):0.027732,(EU916176_Phycodnaviridae_Phaeovir
us:0.457379,AF204951_Phycodnaviridae_Phaeovirus:0.457379)100:0.272944)
83:0.038538,((DQ491002_Phycodnaviridae_Chlorovirus:0.237924,JF411744_P
hycodnaviridae_Chlorovirus:0.237924)100:0.164497,EF101928_Phycodnaviri
dae_Chlorovirus:0.402422)100:0.36644)88:0.057496)87:0.024815,(HQ336222
_Mimiviridae_Mimivirus:0.751075,GU244497_Mimiviridae_Cafeteriavirus:0.
751075)100:0.100097)90:0.016122)87:0.04119):0.00653,((EU522111_Hytrosa
viridae_Muscavirus:0.739328,EF568108_Hytrosaviridae_Glossinavirus:0.73
9328)88:0.154049,(((((((((KR351281_Herpesviridae_Cytomegalovirus:0.08
7677,FJ483968_Herpesviridae_Cytomegalovirus:0.087677)100:0.035797,AY18

6194_Herpesviridae_Cytomegalovirus:0.123475)100:0.133571,(AF480884_Her
pesviridae_Cytomegalovirus:0.094132,AY446894_Herpesviridae_Cytomegalov
irus:0.094132)100:0.162914)100:0.056838,(FJ483967_Herpesviridae_Cytome
galovirus:0.156423,FJ483970_Herpesviridae_Cytomegalovirus:0.156423)100
:0.157461)98:0.07774,(AF281817_Herpesviridae_Unassigned:0.319615,KC503
762_Herpesviridae_Unassigned:0.319615)100:0.07201)100:0.072718,((JX867
617_Herpesviridae_Muromegalovirus:0.237579,AF232689_Herpesviridae_Muro
megalovirus:0.237579)100:0.058689,U68299_Herpesviridae_Muromegalovirus
:0.296268)100:0.168075)100:0.061407,(((KU351741_Herpesviridae_Roseolov
irus:0.077169,AF037218_Herpesviridae_Roseolovirus:0.077169)100:0.10520
4,(AF157706_Herpesviridae_Roseolovirus:0.039843,X83413_Herpesviridae_R
oseolovirus:0.039843)100:0.14253)100:0.148152,KF017583_Herpesviridae_U
nassigned:0.330525)100:0.195225)100:0.102916,((KC462165_Herpesviridae_
Proboscivirus:0.055754,KF921519_Herpesviridae_Proboscivirus:0.055754)1
00:0.198706,KT832477_Herpesviridae_Proboscivirus:0.25446)100:0.374205)
93:0.054941,((((((((X64346_Herpesviridae_Rhadinovirus:0.066954,AF08342
4_Herpesviridae_Rhadinovirus:0.066954)100:0.122419,AF318573_Herpesviri
dae_Rhadinovirus:0.189372):0.012948,KT595939_Herpesviridae_Unassigned:
0.20232)85:0.031066,(AF083501_Herpesviridae_Rhadinovirus:0.150399,AF14
8805_Herpesviridae_Rhadinovirus:0.150399)100:0.082987)89:0.055336,(KM9
24295_Herpesviridae_Percavirus:0.084238,U20824_Herpesviridae_Percaviru
s:0.084238)100:0.204484)90:0.034572,(((KF274499_Herpesviridae_Macaviru
s:0.06434,AF005370_Herpesviridae_Macavirus:0.06434)99:0.03971,AY839756
_Herpesviridae_Macavirus:0.10405)83:0.032841,KJ705001_Herpesviridae_Ma
cavirus:0.136891)100:0.186403)93:0.042375,(U97553_Herpesviridae_Rhadin
ovirus:0.204639,HQ221963_Herpesviridae_Rhadinovirus:0.204639)100:0.161
03)96:0.045687,(((DQ279927_Herpesviridae_Lymphocryptovirus:0.025337,AJ
507799_Herpesviridae_Lymphocryptovirus:0.025337)100:0.120189,AY037858_
Herpesviridae_Lymphocryptovirus:0.145527)100:0.080868,AF319782_Herpesv
iridae_Lymphocryptovirus:0.226395)100:0.184961)100:0.27225)86:0.040264
,((((((((((((AP010838_Herpesviridae_Varicellovirus:0.021268,AY665713_H
erpesviridae_Varicellovirus:0.021268)84:0.007962,JQ343919_Herpesvirida
e_Varicellovirus:0.02923)100:0.015937,AF030027_Herpesviridae_Varicello
virus:0.045167)100:0.033941,KM051845_Herpesviridae_Varicellovirus:0.07
9108)100:0.067981,(FJ478159_Herpesviridae_Varicellovirus:0.103239,KT81
9633_Herpesviridae_Varicellovirus:0.103239)98:0.04385)100:0.077256,(AY
261359_Herpesviridae_Varicellovirus:0.050209,AJ004801_Herpesviridae_Va
ricellovirus:0.050209)100:0.174137):0.013537,(X04370_Herpesviridae_Var
icellovirus:0.068809,AF275348_Herpesviridae_Varicellovirus:0.068809)10
0:0.169074)74:0.032401,BK001744_Herpesviridae_Varicellovirus:0.270284)
:0.008987,EU082088_Herpesviridae_Mardivirus:0.27927)74:0.04745,(((((((
JQ360576_Herpesviridae_Simplexvirus:0.024434,JN561323_Herpesviridae_Si
mplexvirus:0.024434)100:0.030716,JN555585_Herpesviridae_Simplexvirus:0
.05515)100:0.027198,AB825953_Herpesviridae_Simplexvirus:0.082348):0.00
9921,((DQ149153_Herpesviridae_Simplexvirus:0.043079,AF533768_Herpesvir
idae_Simplexvirus:0.043079):0.000295,AY714813_Herpesviridae_Simplexvir
us:0.043374)100:0.048895)100:0.050461,HM625781_Herpesviridae_Simplexvi
rus:0.142729)83:0.019568,KT594769_Herpesviridae_Simplexvirus:0.162298)
93:0.034365,JQ596859_Herpesviridae_Simplexvirus:0.196663)100:0.130057)
:0.03103,(((AB049735_Herpesviridae_Mardivirus:0.117737,AF243438_Herpes
viridae_Mardivirus:0.117737):0.015056,AF291866_Herpesviridae_Mardiviru
s:0.132793)100:0.184357,(KJ668231_Herpesviridae_Mardivirus:0.004157,KX

589235_Herpesviridae_Mardivirus:0.004157)100:0.312993)73:0.0406)100:0.
184878,((AY372243_Herpesviridae_Iltovirus:0.305228,NC_006623_Herpesvir
idae_Iltovirus:0.305228)100:0.198345,KM924292_Herpesviridae_Scutavirus
:0.503573)88:0.039055)100:0.181242)100:0.169508):0.021636):0.027159,((
(((AY509253_Malacoherpesviridae_Ostreavirus:0.676174,JX453331_Malacohe
rpesviridae_Aurivirus:0.676174)100:0.209792,((((DQ657948_Alloherpesvir
idae_Cyprinivirus:0.277397,JQ815364_Alloherpesviridae_Cyprinivirus:0.2
77397)93:0.049277,JQ815363_Alloherpesviridae_Cyprinivirus:0.326673)100
:0.344811,FJ940765_Alloherpesviridae_Cyprinivirus:0.671484)100:0.18728
5,KT995472_Nimaviridae_Whispovirus:0.858769):0.027197):0.020059,((((((
(((((((JN126049_Ackermannviridae_Cba120virus:0.045975,KX438380_Ackerm
annviridae_Cba120virus:0.045975)93:0.018294,HQ259103_Ackermannviridae_
Cba120virus:0.064268):0.016566,KR296692_Ackermannviridae_Cba120virus:0
.080834):0.007714,(JN673056_Ackermannviridae_Cba120virus:0.053638,JN59
3240_Ackermannviridae_Cba120virus:0.053638)76:0.03491):0.010075,(KX245
012_Ackermannviridae_Cba120virus:0.081158,KP797973_Ackermannviridae_Cb
a120virus:0.081158):0.017464):0.00699,((KF669654_Ackermannviridae_Vi1v
irus:0.024569,KF669653_Ackermannviridae_Vi1virus:0.024569)100:0.053479
,FQ312032_Ackermannviridae_Vi1virus:0.078048)94:0.027564):0.006625,((J
X128257_Ackermannviridae_Vi1virus:0.091545,KJ174318_Ackermannviridae_C
ba120virus:0.091545):0.010314,(JX181828_Ackermannviridae_Vi1virus:0.09
1153,KJ174317_Ackermannviridae_Vi1virus:0.091153):0.010706):0.010378)1
00:0.087248,(((KX147096_Ackermannviridae_:0.128457,AB797215_Ackermannv
iridae_:0.128457)94:0.033823,(FJ373894_Ackermannviridae_Ag3virus:0.112
077,JX181829_Ackermannviridae_Ag3virus:0.112077)95:0.050204):0.018506,
(KJ716335_Ackermannviridae_Limestonevirus:0.057133,HE600015_Ackermannv
iridae_Limestonevirus:0.057133)100:0.123654)84:0.018699)100:0.108432,(
JX878496_Ackermannviridae_:0.191108,KP037007_Ackermannviridae_:0.19110
8)100:0.116809)100:0.387418,(((((((((((((KM501444_Myoviridae_T4virus:
0.039213,AP011113_Myoviridae_T4virus:0.039213):0.003169,KF925357_Myovi
ridae_T4virus:0.042382):0.012888,KJ668714_Myoviridae_T4virus:0.055269)
:0.008645,((HM035025_Myoviridae_T4virus:0.026679,JN202312_Myoviridae_T
4virus:0.026679)93:0.023763,HE956711_Myoviridae_T4virus:0.050441):0.01
3473):0.014504,(((KF208315_Myoviridae_T4virus:0.036015,JX128259_Myovir
idae_T4virus:0.036015)100:0.016831,JN986846_Myoviridae_T4virus:0.05284
7):0.002197,KM606994_Myoviridae_T4virus:0.055044)90:0.023374)100:0.082
202,((KM407600_Myoviridae_Rb69virus:0.054142,KF582788_Myoviridae_Rb69v
irus:0.054142)70:0.021304,JX536493_Myoviridae_Rb69virus:0.075446)100:0
.085174)99:0.024476,(((((KP007359_Myoviridae_Js98virus:0.047234,EU8634
09_Myoviridae_Js98virus:0.047234):0.008617,KT176190_Myoviridae_Js98vir
us:0.055851):0.00697,HM071924_Myoviridae_Js98virus:0.062822)100:0.0626
9,HQ829472_Myoviridae_Js98virus:0.125511)70:0.016337,(((GQ981382_Myovi
ridae_Sp18virus:0.056946,KP007361_Myoviridae_Sp18virus:0.056946):0.013
845,KP007360_Myoviridae_Sp18virus:0.070791)71:0.028606,(HM563683_Myovi
ridae_Sp18virus:0.076141,KP007362_Myoviridae_Sp18virus:0.076141)70:0.0
23256)98:0.04245)100:0.043249)87:0.025616,((((KM236240_Myoviridae_Moon
virus:0.046846,KT001915_Myoviridae_Moonvirus:0.046846)100:0.07736,(GU3
23318_Myoviridae_Cc31virus:0.033948,KJ101592_Myoviridae_Cc31virus:0.03
3948)100:0.090258)98:0.033077,(JX181825_Myoviridae_S16virus:0.066199,H
Q331142_Myoviridae_S16virus:0.066199)100:0.091084)96:0.028321,(KR26972
0_Myoviridae_Jd18virus:0.095683,KT239446_Myoviridae_Jd18virus:0.095683
)100:0.089921)89:0.025109)100:0.046018,(KP202158_Myoviridae_Tg1virus:0

.078033,HE956709_Myoviridae_Tg1virus:0.078033)100:0.178698)99:0.034585
,EU863408_Myoviridae_Rb49virus:0.291316)100:0.07619,((((KT001919_Myovi
ridae_Kp15virus:0.011493,KT001918_Myoviridae_Kp15virus:0.011493)100:0.
008184,HQ918180_Myoviridae_Kp15virus:0.019676)72:0.003669,GU295964_Myo
viridae_Kp15virus:0.023346)73:0.012969,KT321315_Myoviridae_Kp15virus:0
.036315)100:0.331191)79:0.02016,(((((JN377895_Myoviridae_Secunda5virus
:0.06208,DQ529280_Myoviridae_Secunda5virus:0.06208):0.005381,JX306041_
Myoviridae_Secunda5virus:0.067461):0.004398,HM452125_Myoviridae_Secund
a5virus:0.07186)96:0.022086,JN377894_Myoviridae_Secunda5virus:0.093945
)100:0.042832,AY375531_Myoviridae_Biquartavirus:0.136777)100:0.250889)
100:0.061975,KP671755_Myoviridae_Schizot4virus:0.449642)100:0.227295,(
(KR052480_Myoviridae_M12virus:0.0636,KF381361_Myoviridae_M12virus:0.06
36)98:0.017576,KR052482_Myoviridae_M12virus:0.081175)100:0.595761)87:0
.018399)100:0.083692,((((FN667789_Myoviridae_Cp220virus:0.069646,FN667
788_Myoviridae_Cp220virus:0.069646)100:0.100865,HE815464_Myoviridae_Cp
220virus:0.170512)100:0.41669,HM246720_Myoviridae_Cp220virus:0.587201)
98:0.093006,((GU296433_Myoviridae_Cp8virus:0.032578,JX569801_Myovirida
e_Cp8virus:0.032578)100:0.031104,(JN132397_Myoviridae_Cp8virus:0.01337
5,FR823450_Myoviridae_Cp8virus:0.013375)100:0.050307)100:0.616525)98:0
.098819)98:0.057127,((((((((((EU877232_Myoviridae_Felixo1virus:0.0353
74,KP869100_Myoviridae_Felixo1virus:0.035374):0.009166,KR014248_Myovir
idae_Felixo1virus:0.04454):0.004662,KM092515_Myoviridae_Felixo1virus:0
.049202)73:0.006948,KM657822_Myoviridae_Felixo1virus:0.05615)80:0.0109
88,KP010413_Myoviridae_Felixo1virus:0.067138):0.022995,(((JN225449_Myo
viridae_Felixo1virus:0.06109,KF055347_Myoviridae_Felixo1virus:0.06109)
:0.004617,KP143762_Myoviridae_Felixo1virus:0.065707):0.00649,JX560968_
Myoviridae_Felixo1virus:0.072198):0.017935)74:0.017117,((KT363872_Myov
iridae_Mooglevirus:0.077883,KM236239_Myoviridae_Mooglevirus:0.077883):
0.013283,(KT454806_Myoviridae_Suspvirus:0.064965,KT454805_Myoviridae_S
uspvirus:0.064965)76:0.026201)75:0.016084)100:0.22805,(HQ728263_Myovir
idae_Ea214virus:0.044862,EU710883_Myoviridae_Ea214virus:0.044862)100:0
.290438)100:0.388162,((((JX181824_Myoviridae_Se1virus:0.041904,GU07061
6_Myoviridae_Se1virus:0.041904)100:0.105937,(KF550303_Myoviridae_Se1vi
rus:0.041103,JN882284_Myoviridae_Se1virus:0.041103)100:0.106737)100:0.
274739,((DQ832317_Myoviridae_V5virus:0.041954,KC690136_Myoviridae_V5vi
rus:0.041954)98:0.018973,(JQ031132_Myoviridae_V5virus:0.037845,KJ19015
8_Myoviridae_V5virus:0.037845)96:0.023083)100:0.361653)99:0.086575,(((
(KC954774_Myoviridae_Cr3virus:0.018996,JQ691612_Myoviridae_Cr3virus:0.
018996)100:0.086219,KT353109_Myoviridae_Cr3virus:0.105215)96:0.022832,
JQ691611_Myoviridae_Cr3virus:0.128047)100:0.150615,JQ015307_Myoviridae
_Cr3virus:0.278661)100:0.230494)100:0.214307):0.026649,(((((HE983845_M
yoviridae_Pakpunavirus:0.019823,LN610572_Myoviridae_Pakpunavirus:0.019
823)100:0.016477,HQ832595_Myoviridae_Pakpunavirus:0.0363)100:0.026,GU9
88610_Myoviridae_Pakpunavirus:0.062301)75:0.026405,(KC862300_Myovirida
e_Pakpunavirus:0.079645,KC862297_Myoviridae_Pakpunavirus:0.079645):0.0
09061)100:0.393537,((KC862299_Myoviridae_Kpp10virus:0.023881,AB472900_
Myoviridae_Kpp10virus:0.023881)100:0.042436,LN610573_Myoviridae_Kpp10v
irus:0.066317)100:0.415926)100:0.267868)89:0.06573,((((KM236244_Siphov
iridae_T5virus:0.071186,KX017521_Siphoviridae_T5virus:0.071186)100:0.0
98174,KM979354_Siphoviridae_T5virus:0.16936):0.002869,(LN887948_Siphov
iridae_T5virus:0.114644,KJ190157_Siphoviridae_T5virus:0.114644)92:0.05
7585)98:0.036247,KP143763_Siphoviridae_T5virus:0.208476)100:0.607365)8

9:0.020312)94:0.034589,((((((((((KF208639_Myoviridae_B4virus:0.051199
,KF669647_Myoviridae_B4virus:0.051199)96:0.025429,(KJ489402_Myoviridae
_B4virus:0.021039,KT307976_Myoviridae_B4virus:0.021039)100:0.05559)90:
0.031159,(KF669662_Myoviridae_B4virus:0.080419,JN790865_Myoviridae_B4v
irus:0.080419)91:0.027369)100:0.126313,(KJ489397_Myoviridae_Bastillevi
rus:0.049409,JF966203_Myoviridae_Bastillevirus:0.049409)100:0.184692)1
00:0.065636,((((KT995480_Myoviridae_Bc431virus:0.083816,KJ451625_Myov
iridae_Bc431virus:0.083816):0.015108,JX094431_Myoviridae_Bc431virus:0.
098925):0.014614,KU577463_Myoviridae_Bc431virus:0.113539)91:0.052024,(
KJ676859_Myoviridae_Bc431virus:0.128038,KJ081346_Myoviridae_Bc431virus
:0.128038)72:0.037524)100:0.062766,(KT224359_Myoviridae_Tsarbombavirus
:0.067227,JN797797_Myoviridae_Tsarbombavirus:0.067227)100:0.161101)100
:0.071409)100:0.071057,(((KJ489401_Myoviridae_Wphvirus:0.027258,KJ4893
99_Myoviridae_Wphvirus:0.027258)100:0.049719,JN654439_Myoviridae_Wphvi
rus:0.076977)72:0.012699,HM144387_Myoviridae_Wphvirus:0.089676)100:0.2
81118):0.012664,(((AP013029_Myoviridae_Nit1virus:0.10478,KF669652_Myov
iridae_Nit1virus:0.10478)89:0.041537,AB930182_Myoviridae_Nit1virus:0.1
46317)100:0.14743,((KJ010547_Myoviridae_Agatevirus:0.097183,JX238501_M
yoviridae_Agatevirus:0.097183)71:0.013176,KM051843_Myoviridae_Agatevir
us:0.110359)100:0.183388)100:0.089712)100:0.148588,((((((KM216423_Myo
viridae_Kayvirus:0.052338,JQ686190_Myoviridae_Kayvirus:0.052338)95:0.0
29283,JX878671_Myoviridae_Kayvirus:0.081622):0.008245,(KP027446_Myovir
idae_Kayvirus:0.062386,KJ888149_Myoviridae_Kayvirus:0.062386)93:0.0274
8):0.009844,((AB853331_Myoviridae_Kayvirus:0.045445,AB853330_Myovirida
e_Kayvirus:0.045445)92:0.02925,AB903967_Myoviridae_Kayvirus:0.074696)8
4:0.025014)100:0.220523,(KF021268_Myoviridae_Sep1virus:0.15281,KP02744
7_Myoviridae_Sep1virus:0.15281)100:0.167423):0.012546,((JX194239_Myovi
ridae_Silviavirus:0.103159,JX846613_Myoviridae_Silviavirus:0.103159)79
:0.029136,KP881332_Myoviridae_Silviavirus:0.132295)100:0.200484)100:0.
131672,DQ004855_Myoviridae_P100virus:0.46445)74:0.067597)95:0.050417,F
J822135_Myoviridae_Unassigned:0.582464)100:0.131114,(((KC595513_Myovir
idae_Cp51virus:0.009946,KC595512_Myoviridae_Cp51virus:0.009946)100:0.0
31564,KF554508_Myoviridae_Cp51virus:0.04151)100:0.386767,KF669649_Myov
iridae_Spo1virus:0.428277)100:0.285301)100:0.119293,((((KP696447_Siph
oviridae_Slashvirus:0.05588,KF669661_Siphoviridae_Slashvirus:0.05588)7
5:0.020579,KF669663_Siphoviridae_Slashvirus:0.076459)87:0.055211,KP696
448_Siphoviridae_Slashvirus:0.13167)100:0.62628,(KJ804259_Siphoviridae
_Sextaecvirus:0.184707,KF929199_Siphoviridae_Sextaecvirus:0.184707)100
:0.573243)98:0.063205,HQ634192_Siphoviridae_Cbastvirus:0.821155):0.011
715):0.037873):0.014087,(AB366653_Myoviridae_Rslunavirus:0.860359,((((
JX100812_Siphoviridae_Phicbkvirus:0.017991,JX100811_Siphoviridae_Phicb
kvirus:0.017991)94:0.008323,JX100813_Siphoviridae_Phicbkvirus:0.026314
)99:0.028146,JX100809_Siphoviridae_Phicbkvirus:0.05446)100:0.087595,JX
100814_Siphoviridae_Phicbkvirus:0.142055)100:0.718304)81:0.02447):0.02
1196):0.015707,(((((((((JX415535_Podoviridae_G7cvirus:0.061302,KC2062
76_Podoviridae_G7cvirus:0.061302):0.001518,KJ135004_Podoviridae_G7cvir
us:0.06282)73:0.013087,(KF562340_Podoviridae_G7cvirus:0.050652,KF19207
5_Podoviridae_G7cvirus:0.050652)89:0.025254)98:0.042089,HQ259105_Podov
iridae_G7cvirus:0.117996):0.002095,KF620435_Podoviridae_G7cvirus:0.120
091)100:0.048774,JX880034_Podoviridae_G7cvirus:0.168864)100:0.21332,((
KF787095_Podoviridae_Jwalphavirus:0.144674,KT321317_Podoviridae_Jwalph
avirus:0.144674)100:0.135412,(KX098389_Podoviridae_Ea92virus:0.006271,

KF806588_Podoviridae_Ea92virus:0.006271)100:0.273814)100:0.102099)98:0
.133924,((KC139520_Podoviridae_Sp58virus:0.083552,KC139517_Podoviridae
_Sp58virus:0.083552)100:0.075556,KM236242_Podoviridae_Sp58virus:0.1591
08)100:0.357)90:0.076714,((((FN422399_Podoviridae_Lit1virus:0.021898,H
G962375_Podoviridae_Lit1virus:0.021898)77:0.008452,JX194238_Podovirida
e_Lit1virus:0.030349)100:0.177388,(FN422398_Podoviridae_Luz7virus:0.07
2733,LC064302_Podoviridae_Luz7virus:0.072733)100:0.135004)100:0.361174
,KJ621082_Podoviridae_Dfl12virus:0.568911):0.023911)100:0.311652,AB757
800_Podoviridae_Kf1virus:0.904474):0.017258):0.011331,((((((KU886223_
Myoviridae_Agrican357virus:0.009405,KU886224_Myoviridae_Agrican357viru
s:0.009405)80:0.004377,KU886222_Myoviridae_Agrican357virus:0.013782):0
.002329,KF806589_Myoviridae_Agrican357virus:0.016111)90:0.004909,KU886
225_Myoviridae_Agrican357virus:0.021019)100:0.628844,((AP014693_Myovir
idae_Rsl2virus:0.071791,AP014927_Myoviridae_Rsl2virus:0.071791)100:0.4
86197,JX233784_Myoviridae_Phikzvirus:0.557987)100:0.091876)98:0.040546
,AJ697969_Myoviridae_Elvirus:0.69041)100:0.040473,(((JX316028_Myovirid
ae_Eah2virus:0.036582,KX397364_Myoviridae_Eah2virus:0.036582)100:0.075
125,JN641803_Myoviridae_Ntreusvirus:0.111707)100:0.048112,KX397370_Myo
viridae_Machinavirus:0.159819)100:0.571064)100:0.202181):0.009109):0.0
30586):0.007736,(((((KT355471_Siphoviridae_Decurrovirus:0.912875,((KU
647626_Siphoviridae_Kelleziovirus:0.040152,KU647627_Siphoviridae_Kelle
ziovirus:0.040152)100:0.863192,((KU160638_Siphoviridae_Amigovirus:0.81
756,KU160654_Siphoviridae_Laroyevirus:0.81756):0.04927,((KM879463_Myov
iridae_Arvunavirus:0.119451,(KU160660_Myoviridae_Arv1virus:0.015037,KX
522650_Myoviridae_Arvunavirus:0.015037)100:0.104414)100:0.447486,((KT3
65401_Myoviridae_Jawnskivirus:0.028071,KU160651_Myoviridae_Jawnskiviru
s:0.028071)100:0.037225,(KU647629_Myoviridae_Chippewavirus:0.058577,(K
U160665_Myoviridae_Sonnyvirus:0.020276,(KU160656_Myoviridae_Sonnyvirus
:0.003574,KU160668_Myoviridae_Sonnyvirus:0.003574)100:0.016702)100:0.0
38301):0.006719)100:0.501641)100:0.299892):0.036514):0.009531):0.00759
3,((((((((((((((GQ303263_Siphoviridae_L5virus:0.036391,JF704093_Sip
hoviridae_L5virus:0.036391):0.013377,JX307702_Siphoviridae_L5virus:0.0
49768)77:0.015778,JN699015_Siphoviridae_L5virus:0.065546)100:0.07635,(
(JX411619_Siphoviridae_L5virus:0.018962,JX307704_Siphoviridae_L5virus:
0.018962)75:0.009045,JQ512844_Siphoviridae_L5virus:0.028007)100:0.1138
89)100:0.074744,((((JF704101_Siphoviridae_L5virus:0.01591,JF704098_Sip
hoviridae_L5virus:0.01591)99:0.021293,HM755814_Siphoviridae_L5virus:0.
037203)84:0.027439,AY129332_Siphoviridae_L5virus:0.064642)81:0.028416,
(JF704111_Siphoviridae_L5virus:0.032083,JF957058_Siphoviridae_L5virus:
0.032083)100:0.060975)100:0.123582)96:0.053696,JF957060_Siphoviridae_L
5virus:0.270336)86:0.028149,((((((JN408461_Siphoviridae_L5virus:0.1428
03,GU339467_Siphoviridae_L5virus:0.142803)85:0.034966,JN408460_Siphovi
ridae_L5virus:0.177769):0.026455,(JF946695_Siphoviridae_L5virus:0.1313
06,DQ398043_Siphoviridae_L5virus:0.131306)96:0.072919):0.019099,EU7442
50_Siphoviridae_L5virus:0.223324):0.011813,(JF704110_Siphoviridae_L5vi
rus:0.063923,JN699005_Siphoviridae_L5virus:0.063923)100:0.171214)79:0.
047956,((JF937094_Siphoviridae_L5virus:0.045622,JF704097_Siphoviridae_
L5virus:0.045622):0.017111,(JN699019_Siphoviridae_L5virus:0.038435,JN0
49605_Siphoviridae_L5virus:0.038435)91:0.024297)100:0.220361):0.015391
)82:0.038777,((((JF704107_Siphoviridae_L5virus:0.015616,JN408459_Sipho
viridae_L5virus:0.015616)100:0.02549,JQ684677_Siphoviridae_L5virus:0.0
41106)99:0.043073,JN083852_Siphoviridae_L5virus:0.084179)100:0.140274,

(JN831654_Siphoviridae_L5virus:0.072525,JX015524_Siphoviridae_L5virus:
0.072525)100:0.151929)100:0.112809):0.012473,KU998249_Siphoviridae_Sou
psvirus:0.349735)83:0.056179,(JN116826_Siphoviridae_Unassigned:0.08452
,JN116827_Siphoviridae_Rer2virus:0.08452)100:0.321395)93:0.041658,((((
((((((((AY500152_Siphoviridae_L5virus:0.038984,JF937108_Siphoviridae_L5
virus:0.038984)95:0.034108,JF937110_Siphoviridae_L5virus:0.073092):0.0
07394,JN020140_Siphoviridae_L5virus:0.080486):0.011772,((AY500153_Siph
oviridae_L5virus:0.063466,JF957057_Siphoviridae_L5virus:0.063466):0.00
8471,JN153086_Siphoviridae_L5virus:0.071937):0.020321):0.000811,((JX30
7705_Siphoviridae_L5virus:0.06257,EU744249_Siphoviridae_L5virus:0.0625
7):0.017949,JF937100_Siphoviridae_L5virus:0.080519):0.01255):0.003961,
(EU744251_Siphoviridae_L5virus:0.085646,JN698998_Siphoviridae_L5virus:
0.085646):0.011384):0.010959,((EU826470_Siphoviridae_L5virus:0.095404,
GU247132_Siphoviridae_L5virus:0.095404):0.007031,(JN398369_Siphovirida
e_L5virus:0.092296,JF937099_Siphoviridae_L5virus:0.092296):0.010139):0
.005554):0.007062,(JN699016_Siphoviridae_L5virus:0.088448,AF271693_Sip
hoviridae_L5virus:0.088448):0.026603):0.017338,(((JN687951_Siphovirida
e_L5virus:0.090427,JF937103_Siphoviridae_L5virus:0.090427):0.007444,(J
N572689_Siphoviridae_L5virus:0.046977,JQ698665_Siphoviridae_L5virus:0.
046977)100:0.050894):0.010356,JN153085_Siphoviridae_L5virus:0.108228)7
7:0.024162):0.006965,EU744248_Siphoviridae_L5virus:0.139355):0.010667,
JN699000_Siphoviridae_L5virus:0.150022)100:0.297551)100:0.287515,(((((
KC700557_Siphoviridae_Likavirus:0.031224,KC700556_Siphoviridae_Likavir
us:0.031224):0.008349,KC700558_Siphoviridae_Likavirus:0.039573)100:0.0
42603,KT184391_Siphoviridae_Likavirus:0.082176)78:0.04081,(((KT152029_
Siphoviridae_Likavirus:0.017547,KT124227_Siphoviridae_Likavirus:0.0175
47)90:0.021567,KT184390_Siphoviridae_Likavirus:0.039114):0.011743,KT12
4229_Siphoviridae_Likavirus:0.050857)100:0.072129)100:0.118873,(((JX18
2370_Siphoviridae_R4virus:0.007247,JX262376_Siphoviridae_R4virus:0.007
247)100:0.188199,JX182369_Siphoviridae_Unassigned:0.195446):0.014645,(
JX889246_Siphoviridae_Camvirus:0.056805,KT186228_Siphoviridae_Camvirus
:0.056805)100:0.153286):0.031768)100:0.493228)78:0.09903,(JX182372_Sip
hoviridae_Phic31virus:0.202441,AJ550940_Siphoviridae_Phic31virus:0.202
441)100:0.631676):0.00589,((((((((((((((((((JX570710_Siphoviridae_P
a6virus:0.01697,JX262223_Siphoviridae_Pa6virus:0.01697):0.000607,KJ578
792_Siphoviridae_Pa6virus:0.017577):0.001798,JX262220_Siphoviridae_Pa6
virus:0.019375):0.000601,((KJ578759_Siphoviridae_Pa6virus:0.016198,KJ5
78758_Siphoviridae_Pa6virus:0.016198):0.002232,JX262217_Siphoviridae_P
a6virus:0.01843):0.001546):0.002419,(KR337644_Siphoviridae_Pa6virus:0.
018132,KJ578774_Siphoviridae_Pa6virus:0.018132):0.004263):0.001482,((J
X570706_Siphoviridae_Pa6virus:0.02018,KR337650_Siphoviridae_Pa6virus:0
.02018):0.002895,JX262216_Siphoviridae_Pa6virus:0.023075):0.000802):0.
000744,(KR337653_Siphoviridae_Pa6virus:0.009249,KR337649_Siphoviridae_
Pa6virus:0.009249)100:0.015372):0.000273,(((KR337647_Siphoviridae_Pa6v
irus:0.019065,KJ578781_Siphoviridae_Pa6virus:0.019065):0.002979,(KJ578
788_Siphoviridae_Pa6virus:0.016735,KJ578779_Siphoviridae_Pa6virus:0.01
6735):0.00531):0.002261,(KJ578776_Siphoviridae_Pa6virus:0.023762,KJ578
765_Siphoviridae_Pa6virus:0.023762):0.000544):0.000587):0.002331,JX262
221_Siphoviridae_Pa6virus:0.027224):0.000437,FJ706172_Siphoviridae_Pa6
virus:0.027661):0.000411,((((KJ578787_Siphoviridae_Pa6virus:0.0212,KR3
37654_Siphoviridae_Pa6virus:0.0212):0.004053,(KJ578784_Siphoviridae_Pa
6virus:6.2e-05,KJ578783_Siphoviridae_Pa6virus:6.2e-

05)100:0.025191):0.00039,KJ578790_Siphoviridae_Pa6virus:0.025643):0.00
0483,(KJ578791_Siphoviridae_Pa6virus:0.021775,JX570712_Siphoviridae_Pa
6virus:0.021775):0.004351):0.001946):0.001352,(((JX570714_Siphoviridae
_Pa6virus:0.02081,KJ578767_Siphoviridae_Pa6virus:0.02081):0.001759,(JX
262224_Siphoviridae_Pa6virus:0.010346,JX262225_Siphoviridae_Pa6virus:0
.010346)100:0.012223):0.00484,((JX262215_Siphoviridae_Pa6virus:0.02040
9,JX262222_Siphoviridae_Pa6virus:0.020409):0.004441,DQ431235_Siphoviri
dae_Pa6virus:0.02485):0.002558):0.002015):0.002092,KJ578785_Siphovirid
ae_Pa6virus:0.031516):0.003913,(JX570705_Siphoviridae_Pa6virus:0.02334
5,JX262218_Siphoviridae_Pa6virus:0.023345)85:0.012083):0.000811,KR3376
45_Siphoviridae_Pa6virus:0.036239):0.002061,KJ578761_Siphoviridae_Pa6v
irus:0.0383):0.00133,((((((KR337648_Siphoviridae_Pa6virus:0.017639,KJ
578770_Siphoviridae_Pa6virus:0.017639):0.006,KR337643_Siphoviridae_Pa6
virus:0.023639):0.001872,(JX570713_Siphoviridae_Pa6virus:0.024614,JX57
0702_Siphoviridae_Pa6virus:0.024614):0.000897):0.001573,JX262219_Sipho
viridae_Pa6virus:0.027084):0.002811,KJ578780_Siphoviridae_Pa6virus:0.0
29895):0.001614,KJ722067_Siphoviridae_Pa6virus:0.031509):0.001067,((((
(KJ578789_Siphoviridae_Pa6virus:5.3e-
05,KJ578778_Siphoviridae_Pa6virus:5.3e-
05)93:0.000441,KJ578786_Siphoviridae_Pa6virus:0.000494)100:0.019359,KJ
578773_Siphoviridae_Pa6virus:0.019853):0.005972,KR337651_Siphoviridae_
Pa6virus:0.025825):0.002798,KR337652_Siphoviridae_Pa6virus:0.028623):0
.003952):0.007055):0.001378,FJ706171_Siphoviridae_Pa6virus:0.041008):0
.007123,KR337646_Siphoviridae_Pa6virus:0.048131)100:0.739199,(((((KX62
0753_Siphoviridae_Doucettevirus:0.16565,KX620751_Siphoviridae_Doucette
virus:0.16565):0.029524,KX620750_Siphoviridae_Doucettevirus:0.195174):
0.021449,KX620754_Siphoviridae_Doucettevirus:0.216623)100:0.3257,(KX62
0749_Siphoviridae_Anatolevirus:0.085152,KX620748_Siphoviridae_Anatolev
irus:0.085152)100:0.457171)91:0.087529,KU984979_Siphoviridae_Pfr1virus
:0.629852)92:0.157478):0.029852,((((KU160643_Siphoviridae_Korravirus:
0.022795,KU160640_Siphoviridae_Korravirus:0.022795):0.002502,KU160659_
Siphoviridae_Korravirus:0.025297)100:0.033958,(KU160661_Siphoviridae_K
orravirus:0.036199,KU160648_Siphoviridae_Korravirus:0.036199)88:0.0230
56)97:0.05041,(((KU160653_Siphoviridae_Korravirus:0.014377,KU160645_Si
phoviridae_Korravirus:0.014377)100:0.024538,KU160672_Siphoviridae_Korr
avirus:0.038915)100:0.02088,KU160652_Siphoviridae_Korravirus:0.059795)
99:0.049869)100:0.606668,KU998237_Siphoviridae_Vendettavirus:0.716333)
:0.100849):0.022825):0.0339,((((((((((((((JF937098_Siphoviridae_Che
8virus:0.123072,GU060500_Siphoviridae_Che8virus:0.123072):0.037607,FJ1
74690_Siphoviridae_Che8virus:0.160679):0.015198,(JN398368_Siphoviridae
_Che8virus:0.140345,JN859129_Siphoviridae_Che8virus:0.140345):0.035533
):0.016602,(((HQ728524_Siphoviridae_Che8virus:0.067566,JX121091_Siphov
iridae_Che8virus:0.067566)100:0.0684,JN698996_Siphoviridae_Che8virus:0
.135966)77:0.049624,EF536069_Siphoviridae_Che8virus:0.185589):0.00689)
:0.030208,((JN020141_Siphoviridae_Che8virus:0.159983,JF937093_Siphovir
idae_Che8virus:0.159983):0.045812,JN699012_Siphoviridae_Che8virus:0.20
5795):0.016892):0.019213,((JQ300538_Siphoviridae_Che8virus:0.160443,JN
020142_Siphoviridae_Che8virus:0.160443)83:0.054422,AY129330_Siphovirid
ae_Che8virus:0.214865):0.027035):0.013662,((JN020143_Siphoviridae_Che8
virus:0.16976,JX411620_Siphoviridae_Che8virus:0.16976)87:0.057186,FJ17
4692_Siphoviridae_Che8virus:0.226945):0.028616):0.013148,((((DQ398050
_Siphoviridae_Che8virus:0.13743,EU816590_Siphoviridae_Che8virus:0.1374

3):0.03759,JF704117_Siphoviridae_Che8virus:0.175019):0.027655,(DQ39804
5_Siphoviridae_Che8virus:0.148132,JN542517_Siphoviridae_Che8virus:0.14
8132):0.054542):0.030429,JF937102_Siphoviridae_Che8virus:0.233103):0.0
10038,FJ174693_Siphoviridae_Che8virus:0.243141):0.02557)100:0.294327,(
(AY129336_Siphoviridae_Che8virus:0.200185,JQ809702_Siphoviridae_Che8vi
rus:0.200185)98:0.052176,JF704115_Siphoviridae_Che8virus:0.252361)100:
0.310676)99:0.086537,(JN698993_Siphoviridae_Corndogvirus:0.037101,AY12
9335_Siphoviridae_Corndogvirus:0.037101)100:0.612473):0.007613,(((((EU
816588_Siphoviridae_Cjw1virus:0.07562,AY129331_Siphoviridae_Cjw1virus:
0.07562):0.009299,JF937106_Siphoviridae_Cjw1virus:0.084919):0.010261,(
JN006061_Siphoviridae_Cjw1virus:0.061124,JN412590_Siphoviridae_Cjw1vir
us:0.061124)89:0.034055):0.011807,(((JF937091_Siphoviridae_Cjw1virus:0
.047889,DQ398041_Siphoviridae_Cjw1virus:0.047889)88:0.035868,GQ303265_
Siphoviridae_Cjw1virus:0.083758):0.007054,EU816591_Siphoviridae_Cjw1vi
rus:0.090811):0.016175)100:0.488614,(((((AY129338_Siphoviridae_Omegavi
rus:0.143603,JF937101_Siphoviridae_Omegavirus:0.143603)100:0.072759,(J
F957059_Siphoviridae_Omegavirus:0.074968,JF937090_Siphoviridae_Omegavi
rus:0.074968)100:0.141394)100:0.054928,JN698997_Siphoviridae_Omegaviru
s:0.27129)80:0.022711,JN201525_Siphoviridae_Omegavirus:0.294002)100:0.
283245,KJ567043_Siphoviridae_Gaiavirus:0.577247):0.018354)93:0.061587)
83:0.038202,((((((KP027200_Siphoviridae_Fishburnevirus:0.03187,JN57206
1_Siphoviridae_Fishburnevirus:0.03187)99:0.028098,KT347313_Siphovirida
e_Fishburnevirus:0.059967):0.012088,(KC691256_Siphoviridae_Fishburnevi
rus:0.026894,KF841477_Siphoviridae_Fishburnevirus:0.026894)100:0.04516
1)100:0.175819,(KR080195_Siphoviridae_Phayoncevirus:0.199659,JN412591_
Siphoviridae_Bignuzvirus:0.199659):0.048215)100:0.240112,((FJ168659_Si
phoviridae_Brujitavirus:0.291822,JN699001_Siphoviridae_Brujitavirus:0.
291822)100:0.128114,(KP027201_Siphoviridae_Che9cvirus:0.211901,AY12933
3_Siphoviridae_Che9cvirus:0.211901)100:0.208036)91:0.068049)87:0.05425
4,((((((JN624851_Siphoviridae_Redivirus:0.158671,KU935727_Siphoviridae
_Redivirus:0.158671):0.013248,KF986246_Siphoviridae_Buttersvirus:0.171
919):0.020385,KU935729_Siphoviridae_Redivirus:0.192304):0.012754,KU935
728_Siphoviridae_Charlievirus:0.205058):0.020363,(KU935731_Siphovirida
e_Redivirus:0.162361,KC576783_Siphoviridae_Buttersvirus:0.162361):0.06
3061):0.013205,(KM588359_Siphoviridae_Charlievirus:0.156319,JN256079_S
iphoviridae_Charlievirus:0.156319)89:0.082308)100:0.303613)100:0.15315
)72:0.077777,((((JN680858_Siphoviridae_Bronvirus:0.063358,JF744988_Sip
hoviridae_Bronvirus:0.063358)100:0.117226,(JF704108_Siphoviridae_Bronv
irus:0.056492,HM152763_Siphoviridae_Bronvirus:0.056492)100:0.124092)10
0:0.381038,((JF937105_Siphoviridae_Reyvirus:0.184652,JN699628_Siphovir
idae_Bongovirus:0.184652)100:0.28727,DQ398052_Siphoviridae_Wildcatviru
s:0.471922)100:0.0897)100:0.148414,((KJ538721_Siphoviridae_Marvinvirus
:0.060057,JF704100_Siphoviridae_Marvinvirus:0.060057)100:0.627239,KJ51
0413_Siphoviridae_Bernal13virus:0.687296):0.02274)87:0.06313):0.014288
,(((((((((JN185608_Siphoviridae_Tm4virus:0.027043,HM152764_Siphovirida
e_Tm4virus:0.027043):0.00206,HM152767_Siphoviridae_Tm4virus:0.029103):
0.010865,JF704106_Siphoviridae_Tm4virus:0.039968)100:0.224409,((JF9371
04_Siphoviridae_Tm4virus:0.0575,JX042579_Siphoviridae_Tm4virus:0.0575)
100:0.179451,JN831653_Siphoviridae_Tm4virus:0.236951):0.027426):0.0307
58,AF068845_Siphoviridae_Tm4virus:0.295135)98:0.050561,JN243855_Siphov
iridae_Tm4virus:0.345695)100:0.181476,(JN412593_Siphoviridae_Liefievir
us:0.030601,DQ398042_Siphoviridae_Liefievirus:0.030601)100:0.49657)100

:0.139697,(KF024728_Siphoviridae_Ff47virus:0.024157,JX901189_Siphoviri
dae_Ff47virus:0.024157)100:0.64271)91:0.038869,(KJ028219_Siphoviridae_
Trigintaduovirus:0.460862,EU203571_Siphoviridae_Gilesvirus:0.460862)10
0:0.244874)71:0.081718)74:0.02498,(((JN412589_Siphoviridae_Patiencevi
rus:0.261392,AY129339_Siphoviridae_Barnyardvirus:0.261392)98:0.059995,
(EU770222_Siphoviridae_Barnyardvirus:0.188861,FJ174691_Siphoviridae_Ba
rnyardvirus:0.188861)100:0.132526)100:0.145004,((DQ398047_Siphoviridae
_Pbi1virus:0.007548,DQ398051_Siphoviridae_Plotvirus:0.007548)100:0.185
2,KJ194582_Siphoviridae_Hawkeyevirus:0.192748)100:0.273643)99:0.086029
,(JF704112_Siphoviridae_Send513virus:0.037143,KF416342_Siphoviridae_Se
nd513virus:0.037143)100:0.515277)100:0.260014):0.04683,((((((((((JN6
99011_Siphoviridae_Coopervirus:0.028678,JF704094_Siphoviridae_Coopervi
rus:0.028678):0.003366,DQ398044_Siphoviridae_Coopervirus:0.032044):0.0
07513,KF279411_Siphoviridae_Coopervirus:0.039557):0.006306,EU770221_Si
phoviridae_Coopervirus:0.045863):0.011584,(((JF704104_Siphoviridae_Coo
pervirus:0.009121,KT270441_Siphoviridae_Coopervirus:0.009121)100:0.015
925,KF279412_Siphoviridae_Coopervirus:0.025046)100:0.02282,KF493881_Si
phoviridae_Coopervirus:0.047866):0.009581)99:0.042764,KR080194_Siphovi
ridae_Coopervirus:0.100211)100:0.101419,((KR080199_Siphoviridae_Acadia
nvirus:0.053444,JN699007_Siphoviridae_Acadianvirus:0.053444)75:0.01473
,KF024727_Siphoviridae_Acadianvirus:0.068173)100:0.133457)100:0.064224
,((((KF713485_Siphoviridae_Pg1virus:0.024955,KJ174157_Siphoviridae_Pg1
virus:0.024955):0.004178,(AF547430_Siphoviridae_Pg1virus:0.013898,JN00
6064_Siphoviridae_Pg1virus:0.013898)91:0.015235):0.003339,(KJ595576_Si
phoviridae_Pg1virus:0.014545,KR781349_Siphoviridae_Pg1virus:0.014545)9
2:0.017927)76:0.016687,JN192463_Siphoviridae_Pg1virus:0.049159)100:0.2
16695)98:0.047763,(((JN698992_Siphoviridae_Pipefishvirus:0.044484,JN69
9003_Siphoviridae_Pipefishvirus:0.044484):0.008263,KF493879_Siphovirid
ae_Pipefishvirus:0.052747):0.014406,DQ398049_Siphoviridae_Pipefishviru
s:0.067153)100:0.246465):0.045593,((KF024722_Siphoviridae_L5virus:0.00
6845,AY129334_Siphoviridae_Rosebushvirus:0.006845)100:0.031485,KR99793
2_Siphoviridae_Rosebushvirus:0.03833)100:0.320881)100:0.413806,(KP8764
65_Siphoviridae_Ydn12virus:0.031281,KP876466_Siphoviridae_Ydn12virus:0
.031281)100:0.741735)80:0.04462,KU160669_Siphoviridae_Tankvirus:0.8176
37):0.041626):0.010979,((((((KX557287_Siphoviridae_Nymphadoravirus:0.0
51733,KU963257_Siphoviridae_Nymphadoravirus:0.051733)100:0.086433,KU96
3255_Siphoviridae_Nymphadoravirus:0.138166)100:0.205476,(KU963249_Siph
oviridae_Baxtervirus:0.181616,KU963263_Siphoviridae_Baxtervirus:0.1816
16)100:0.162026)99:0.183218,KU963247_Siphoviridae_Attisvirus:0.52686)1
00:0.239094,(KU998234_Siphoviridae_Wizardvirus:0.095926,KX557286_Sipho
viridae_Wizardvirus:0.095926)100:0.670029):0.052772,(((KU998244_Siphov
iridae_Smoothievirus:0.048668,KU998247_Siphoviridae_Smoothievirus:0.04
8668)81:0.015454,KU998246_Siphoviridae_Smoothievirus:0.064121)100:0.18
9814,KU998245_Siphoviridae_Smoothievirus:0.253936)100:0.564791):0.0515
16):0.003665):0.04019,(((((KU998241_Siphoviridae_Woesvirus:0.036624,KU
963245_Siphoviridae_Woesvirus:0.036624)100:0.159203,KU998240_Siphoviri
dae_Woesvirus:0.195826):0.026894,((KR011063_Siphoviridae_Tin2virus:0.0
01288,KR011064_Siphoviridae_Tin2virus:0.001288)100:0.05718,KR011062_Si
phoviridae_Tin2virus:0.058468)100:0.164252):0.054542,((KU998243_Siphov
iridae_Demosthenesvirus:0.003937,KU963258_Siphoviridae_Demosthenesviru
s:0.003937)100:0.01826,KU998242_Siphoviridae_Demosthenesvirus:0.022197
)100:0.255065)100:0.193788,KP790008_Siphoviridae_Gordtnkvirus:0.47105)

100:0.443048):0.00328,((((JN412588_Myoviridae_Bxz1virus:0.043134,AY129
337_Myoviridae_Bxz1virus:0.043134):0.006609,JN204348_Myoviridae_Bxz1vi
rus:0.049743)72:0.00605,JN699627_Myoviridae_Bxz1virus:0.055793)79:0.01
8683,(KT281790_Myoviridae_Bxz1virus:0.055492,JF704092_Myoviridae_Bxz1v
irus:0.055492):0.018984)100:0.842901):0.003091):0.011779,((((((((((((
((AP001553_Siphoviridae_Phietavirus:0.180556,AY954963_Siphoviridae_Phi
etavirus:0.180556):0.04049,AY954964_Siphoviridae_Phietavirus:0.221046)
:0.011869,AY954962_Siphoviridae_Phietavirus:0.232915):0.038152,((DQ908
929_Siphoviridae_Phietavirus:0.105205,AY954965_Siphoviridae_Phietaviru
s:0.105205)100:0.134343,AB370268_Siphoviridae_Phietavirus:0.239548):0.
031518)72:0.039674,((AY954967_Siphoviridae_Phietavirus:0.148828,AY9549
66_Siphoviridae_Phietavirus:0.148828)100:0.094911,AY954968_Siphovirida
e_Phietavirus:0.243739)97:0.067002):0.027141,((DQ530362_Siphoviridae_P
hietavirus:0.219617,AP008954_Siphoviridae_Phietavirus:0.219617):0.0193
76,AY954960_Siphoviridae_Phietavirus:0.238993)86:0.098889)100:0.093608
,((((((DQ530360_Siphoviridae_Phietavirus:0.148139,DQ530359_Siphovirid
ae_Phietavirus:0.148139):0.024249,(DQ517338_Siphoviridae_Phietavirus:0
.066878,AY954952_Siphoviridae_Phietavirus:0.066878)100:0.10551):0.0365
19,((AY954951_Siphoviridae_Phietavirus:0.147559,AF424781_Siphoviridae_
Phietavirus:0.147559):0.04379,AP008953_Siphoviridae_Phietavirus:0.1913
49):0.017558):0.019905,(AB370205_Siphoviridae_Phietavirus:0.199654,AY9
54953_Siphoviridae_Phietavirus:0.199654):0.029158):0.015784,GU477322_S
iphoviridae_Phietavirus:0.244596)83:0.047274,EU861004_Siphoviridae_Phi
etavirus:0.29187)99:0.125632,AY954950_Siphoviridae_Phietavirus:0.41750
2):0.013989):0.070819,(((((DQ834250_Siphoviridae_Phietavirus:0.188092,
JN192401_Siphoviridae_Phietavirus:0.188092)82:0.031456,DQ831957_Siphov
iridae_Phietavirus:0.219547):0.047284,JN192400_Siphoviridae_Phietaviru
s:0.266831)96:0.091404,AY954959_Siphoviridae_Phietavirus:0.358235):0.0
3197,AY954958_Siphoviridae_Phietavirus:0.390206)99:0.112104)100:0.2812
72,(((((((KP296793_Siphoviridae_Sitaravirus:0.054274,KP202972_Siphovi
ridae_Sitaravirus:0.054274)89:0.054825,KP296796_Siphoviridae_Sitaravir
us:0.109099)76:0.054289,KT361650_Siphoviridae_Sitaravirus:0.163388):0.
012712,(KP296795_Siphoviridae_Sitaravirus:0.096788,KP296791_Siphovirid
ae_Sitaravirus:0.096788)96:0.079311)100:0.312553,(KT361654_Siphovirida
e_Vegasvirus:0.404726,KT361651_Siphoviridae_Harrisonvirus:0.404726)83:
0.083926)100:0.148457,(((((AF424782_Siphoviridae_Triavirus:0.11106,AY9
54956_Siphoviridae_Triavirus:0.11106):0.014529,EU861005_Siphoviridae_T
riavirus:0.125589)71:0.033801,AY954957_Siphoviridae_Triavirus:0.15939)
100:0.114607,AB045978_Siphoviridae_Triavirus:0.273997)91:0.045166,AY95
4955_Siphoviridae_Triavirus:0.319164)100:0.317946):0.050186,((AF424783
_Siphoviridae_Biseptimavirus:0.187765,AB243556_Siphoviridae_Biseptimav
irus:0.187765)100:0.204,AY508486_Siphoviridae_Biseptimavirus:0.391765)
100:0.29553)86:0.063754,(DQ340064_Myoviridae_Svunavirus:0.114459,DQ840
344_Myoviridae_Svunavirus:0.114459)100:0.63659)78:0.032533):0.037306,(
(KC595518_Myoviridae_Abouovirus:0.053887,KC595517_Myoviridae_Abouoviru
s:0.053887)100:0.244778,(KT151956_Myoviridae_Jimmervirus:0.043048,KC59
5514_Myoviridae_Jimmervirus:0.043048)100:0.255617)100:0.522223):0.0012
85,(((EU887664_Siphoviridae_Tp21virus:0.065842,JX887877_Siphoviridae_T
p21virus:0.065842)100:0.592845,DQ289555_Siphoviridae_Wbetavirus:0.6586
88)72:0.04145,(EU874396_Siphoviridae_Cecivirus:0.137939,GU229986_Sipho
viridae_Cecivirus:0.137939)100:0.562199)86:0.122035):0.035324,(((((HE9
62497_Siphoviridae_Sap6virus:0.071224,KF192053_Siphoviridae_Sap6virus:

0.071224):0.020709,JF731128_Siphoviridae_Sap6virus:0.091933)89:0.03204
,(KJ094032_Siphoviridae_Sap6virus:0.087568,AB712291_Siphoviridae_Sap6v
irus:0.087568)91:0.036405)100:0.466601,(((JX442241_Siphoviridae_P70vir
us:0.060827,KJ094021_Siphoviridae_P70virus:0.060827):0.029335,(JX12692
0_Siphoviridae_P70virus:0.037531,JX126919_Siphoviridae_P70virus:0.0375
31)97:0.052631):0.015741,KJ094020_Siphoviridae_P70virus:0.105903)100:0
.484671)100:0.229021,(((GQ478084_Siphoviridae_Phifelvirus:0.08576,GQ47
8081_Siphoviridae_Phifelvirus:0.08576)100:0.356097,GQ478086_Siphovirid
ae_Phifelvirus:0.441857)100:0.256658,((JN051154_Siphoviridae_Phijl1vir
us:0.052204,JX486087_Siphoviridae_Phijl1virus:0.052204)100:0.257215,AY
236756_Siphoviridae_Phijl1virus:0.309419)100:0.389096)98:0.12108):0.03
7902):0.005618,(((((AF115102_Siphoviridae_Sfi21dt1virus:0.134153,AF085
222_Siphoviridae_Sfi21dt1virus:0.134153):0.03153,AF115103_Siphoviridae
_Sfi21dt1virus:0.165683)94:0.09833,(FJ236310_Siphoviridae_Sfi21dt1viru
s:0.17028,AF145054_Siphoviridae_Sfi21dt1virus:0.17028)92:0.093733)100:
0.286171,(((EF529515_Siphoviridae_Sfi11virus:0.063534,AY699705_Siphovi
ridae_Sfi11virus:0.063534)98:0.041725,FJ226752_Siphoviridae_Sfi11virus
:0.105259)99:0.103216,(AF158600_Siphoviridae_Sfi11virus:0.141267,U8897
4_Siphoviridae_Sfi11virus:0.141267)72:0.067208)100:0.341709)100:0.2281
69,(AJ312240_Siphoviridae_Psavirus:0.063171,JX120799_Siphoviridae_Psav
irus:0.063171)100:0.715182)74:0.084761):0.021731,((((((((FJ848885_Siph
oviridae_Sk1virus:0.00262,FJ848884_Siphoviridae_Sk1virus:0.00262)100:0
.092759,FJ848883_Siphoviridae_Sk1virus:0.095379):0.043387,AF009630_Sip
hoviridae_Sk1virus:0.138766):0.026514,((JQ740804_Siphoviridae_Sk1virus
:0.047235,JQ740813_Siphoviridae_Sk1virus:0.047235):0.005887,JQ740789_S
iphoviridae_Sk1virus:0.053122)100:0.112158):0.012225,((JQ740787_Siphov
iridae_Sk1virus:0.099073,JQ740788_Siphoviridae_Sk1virus:0.099073):0.03
1766,FJ848882_Siphoviridae_Sk1virus:0.130839):0.046667):0.031603,((((A
F011378_Siphoviridae_Sk1virus:0.018606,DQ227764_Siphoviridae_Sk1virus:
0.018606)100:0.075702,GQ979703_Siphoviridae_Sk1virus:0.094308)85:0.052
546,EU221285_Siphoviridae_Sk1virus:0.146854)81:0.030997,((DQ054536_Sip
hoviridae_Sk1virus:0.113218,DQ227763_Siphoviridae_Sk1virus:0.113218)71
:0.028806,FJ848881_Siphoviridae_Sk1virus:0.142024):0.035828):0.031257)
100:0.586205,(((((KC013023_Siphoviridae_Lmd1virus:0.033692,JQ659259_Si
phoviridae_Lmd1virus:0.033692)82:0.023073,KC013024_Siphoviridae_Lmd1vi
rus:0.056765)100:0.107311,(KC013025_Siphoviridae_Lmd1virus:0.094257,KC
013022_Siphoviridae_Lmd1virus:0.094257)99:0.069819)91:0.058956,KC01302
1_Siphoviridae_Lmd1virus:0.223032)100:0.143558,(((KM262192_Siphovirida
e_Una4virus:0.047358,GQ451696_Siphoviridae_Una4virus:0.047358)97:0.084
45,(KM262191_Siphoviridae_Una4virus:0.009904,KC013026_Siphoviridae_Una
4virus:0.009904)100:0.121904):0.02944,(KC013029_Siphoviridae_Una4virus
:0.010021,KC013027_Siphoviridae_Una4virus:0.010021)100:0.151228)100:0.
205341)100:0.428724)73:0.036913,((((KF188410_Siphoviridae_C5virus:0.08
2497,EU340421_Siphoviridae_C5virus:0.082497)96:0.039096,KJ564037_Sipho
viridae_C5virus:0.121592):0.015724,(AY739900_Siphoviridae_C5virus:0.12
7909,KJ564038_Siphoviridae_C5virus:0.127909):0.009407)94:0.030654,KJ56
4036_Siphoviridae_C5virus:0.16797)100:0.664256):0.052619):0.035489,(((
KU647628_Siphoviridae_Mudcatvirus:0.120983,KU160642_Siphoviridae_Mudca
tvirus:0.120983)100:0.276106,(KU160646_Siphoviridae_Gordonvirus:0.0384
63,KU160641_Siphoviridae_Gordonvirus:0.038463)100:0.358626)100:0.09243
9,(GU580942_Siphoviridae_Pepy6virus:0.12339,GU580941_Siphoviridae_Pepy
6virus:0.12339)100:0.366139)100:0.430806):0.011913):0.016945,(((((KM23

6248_Podoviridae_Pagevirus:0.045355,KP411017_Podoviridae_Pagevirus:0.0
45355):0.017879,(KF669660_Podoviridae_Pagevirus:0.032854,KF669655_Podo
viridae_Pagevirus:0.032854)82:0.030381)100:0.136347,KM236247_Podovirid
ae_Pagevirus:0.199581)100:0.670482,((((KF669651_Siphoviridae_Andromeda
virus:0.012757,KC330684_Siphoviridae_Andromedavirus:0.012757)78:0.0060
01,KC330679_Siphoviridae_Andromedavirus:0.018758)99:0.024964,(KC330682
_Siphoviridae_Andromedavirus:0.005024,KC330680_Siphoviridae_Andromedav
irus:0.005024)100:0.038698)83:0.046939,((KF669659_Siphoviridae_Androme
davirus:0.026289,KC330683_Siphoviridae_Andromedavirus:0.026289)91:0.02
2448,KF669648_Siphoviridae_Andromedavirus:0.048737)83:0.041924)100:0.7
79402):0.055327,((KY565347_Siphoviridae_Tp84virus:0.836483,(((JX976549
_Myoviridae_Ap22virus:0.185483,HM368260_Myoviridae_Ap22virus:0.185483)
82:0.092878,KJ817802_Myoviridae_Ap22virus:0.27836)79:0.029375,HE806280
_Myoviridae_Ap22virus:0.307736)100:0.528748):0.051572,(((GU815091_Myov
iridae_Pbunavirus:0.024985,KR054033_Myoviridae_Pbunavirus:0.024985)83:
0.011302,AB560486_Myoviridae_Pbunavirus:0.036287)100:0.032684,(KR05403
0_Myoviridae_Pbunavirus:0.045906,LN610589_Myoviridae_Pbunavirus:0.0459
06)95:0.023065)100:0.819084):0.037335):0.023803):0.017004,((((((((((
(((KP719134_Siphoviridae_Nonagvirus:0.076286,KJ419279_Siphoviridae_Non
agvirus:0.076286)95:0.07067,(KP719133_Siphoviridae_Nonagvirus:0.056936
,KP719132_Siphoviridae_Nonagvirus:0.056936)100:0.090019)100:0.219509,(
KM236243_Siphoviridae_Seuratvirus:0.109761,KP064094_Siphoviridae_Seura
tvirus:0.109761)100:0.256703)100:0.182642,(JQ067084_Siphoviridae_Np1vi
rus:0.03151,KX129925_Siphoviridae_Np1virus:0.03151)100:0.517596)78:0.1
03815,((((JQ307387_Siphoviridae_Septima3virus:0.021314,HG962376_Siphov
iridae_Septima3virus:0.021314):0.005578,NC_007806_Siphoviridae_Septima
3virus:0.026892)100:0.111035,JF939047_Siphoviridae_Septima3virus:0.137
927)100:0.207378,(KR935213_Siphoviridae_Titanvirus:0.020829,KR935215_S
iphoviridae_Titanvirus:0.020829)100:0.324477)100:0.307616)75:0.03994,J
X483876_Myoviridae_Rheph4virus:0.692861)83:0.105723,(((((LN610584_Siph
oviridae_Ab18virus:0.057693,LN610577_Siphoviridae_Ab18virus:0.057693)1
00:0.072284,JQ067087_Siphoviridae_Ab18virus:0.129977)100:0.339814,((JX
131330_Siphoviridae_Yuavirus:0.047934,KC758116_Siphoviridae_Yuavirus:0
.047934)93:0.023866,KT734862_Siphoviridae_Yuavirus:0.0718)100:0.397991
)100:0.121455,(JQ067093_Siphoviridae_Pamx74virus:0.199394,JQ067089_Sip
hoviridae_Pamx74virus:0.199394)100:0.391852):0.138526,((KP202969_Sipho
viridae_Jwxvirus:0.093135,KP202970_Siphoviridae_Jwxvirus:0.093135)100:
0.550655,(KT266805_Siphoviridae_Rdjlvirus:0.02389,HM151342_Siphovirida
e_Rdjlvirus:0.02389)100:0.6199):0.085982)91:0.068811):0.032751,(JQ6921
07_Siphoviridae_Ssp2virus:0.049624,JX556418_Siphoviridae_Ssp2virus:0.0
49624)100:0.781711):0.018564,(((((((((((JQ007353_Siphoviridae_Jersey
virus:0.061725,KR270151_Siphoviridae_Jerseyvirus:0.061725):0.014519,(E
F177456_Siphoviridae_Jerseyvirus:0.055258,HE775250_Siphoviridae_Jersey
virus:0.055258):0.020986):0.009428,KC139511_Siphoviridae_Jerseyvirus:0
.085672):0.016522,AY730274_Siphoviridae_Jerseyvirus:0.102195):0.003946
,JX297445_Siphoviridae_Jerseyvirus:0.10614):0.02056,((JX202565_Siphovi
ridae_Jerseyvirus:0.090836,KF562865_Siphoviridae_Jerseyvirus:0.090836)
:0.023576,KF562864_Siphoviridae_Jerseyvirus:0.114412):0.012289):0.0220
37,KF148055_Siphoviridae_Jerseyvirus:0.148738):0.014824,KM272358_Sipho
viridae_Jerseyvirus:0.163562)99:0.08516,((GU196278_Siphoviridae_K1gvir
us:0.101062,GU196277_Siphoviridae_K1gvirus:0.101062)88:0.056447,(GU196
280_Siphoviridae_K1gvirus:0.029697,GU196279_Siphoviridae_K1gvirus:0.02

9697)100:0.127812)100:0.091213)85:0.036117,KC139518_Siphoviridae_Sp31v
irus:0.284839)92:0.091854,KC832325_Siphoviridae_Jerseyvirus:0.376692)9
9:0.197177,((((((FJ750948_Siphoviridae_Hk578virus:0.060663,JX865427_Si
phoviridae_Hk578virus:0.060663):0.006101,KM233151_Siphoviridae_Hk578vi
rus:0.066764):0.013023,JQ086375_Siphoviridae_Hk578virus:0.079786):0.02
7171,(GQ502199_Siphoviridae_Hk578virus:0.060875,JN984867_Siphoviridae_
Hk578virus:0.060875)98:0.046083):0.014034,KM896878_Siphoviridae_Hk578v
irus:0.120991)100:0.192277,KF772233_Siphoviridae_Eiauvirus:0.313268)10
0:0.260602)99:0.246624,((((JN871591_Siphoviridae_Chivirus:0.026472,KC6
77662_Siphoviridae_Chivirus:0.026472)77:0.012965,KC139512_Siphoviridae
_Chivirus:0.039436)70:0.026957,JX094499_Siphoviridae_Chivirus:0.066393
):0.013318,KC139519_Siphoviridae_Chivirus:0.079712)100:0.740782):0.029
405):0.033567,((((((((JF501022_Siphoviridae_Kp36virus:0.132249,KP65815
7_Siphoviridae_Kp36virus:0.132249):0.027928,KR262148_Siphoviridae_Kp36
virus:0.160177):0.016386,(KT001920_Siphoviridae_Kp36virus:0.150183,KR2
69719_Siphoviridae_Kp36virus:0.150183):0.02638)100:0.104318,(((KP08558
6_Siphoviridae_T1virus:0.056662,KU194206_Siphoviridae_T1virus:0.056662
):0.009953,JX912252_Siphoviridae_T1virus:0.066614)100:0.064093,KU19420
5_Siphoviridae_T1virus:0.130707)100:0.150174)71:0.009575,((AY308796_Si
phoviridae_Tlsvirus:0.140842,KM236241_Siphoviridae_Tlsvirus:0.140842):
0.018129,KC139513_Siphoviridae_Tlsvirus:0.158972)100:0.131484)87:0.030
295,((((((KF771239_Siphoviridae_Rogue1virus:0.026051,KF771237_Siphovir
idae_Rogue1virus:0.026051)97:0.016009,KF771238_Siphoviridae_Rogue1viru
s:0.04206)100:0.028056,KT825490_Siphoviridae_Rogue1virus:0.070115)85:0
.02432,KJ668713_Siphoviridae_Rogue1virus:0.094436)80:0.023047,(KC33387
9_Siphoviridae_Rogue1virus:0.084499,KC579452_Siphoviridae_Rogue1virus:
0.084499)82:0.032983)100:0.116995,(AM156909_Siphoviridae_Rtpvirus:0.18
6819,JN986845_Siphoviridae_Rtpvirus:0.186819)81:0.047659)99:0.086274)1
00:0.398326,JF974294_Siphoviridae_Pis4avirus:0.719077):0.053217,((((((
((((JQ182732_Siphoviridae_Hk97virus:0.121761,JQ086374_Siphoviridae_Hk9
7virus:0.121761):0.022677,JQ086372_Siphoviridae_Hk97virus:0.144438):0.
037753,JQ086369_Siphoviridae_Hk97virus:0.182191):0.01058,AF069529_Siph
oviridae_Hk97virus:0.192771):0.023766,((HM173637_Siphoviridae_Hk97viru
s:0.157956,JQ086373_Siphoviridae_Hk97virus:0.157956):0.022406,JQ086377
_Siphoviridae_Hk97virus:0.180362):0.036176)74:0.030685,JQ182727_Siphov
iridae_Hk97virus:0.247223)94:0.079303,(JQ182726_Siphoviridae_Hk97virus
:0.220515,AF069308_Siphoviridae_Hk97virus:0.220515)95:0.106011)100:0.3
2292,((AF447491_Siphoviridae_E125virus:0.166812,CP000625_Siphoviridae_
E125virus:0.166812):0.032134,AY453853_Siphoviridae_E125virus:0.198946)
100:0.4505)78:0.054582,((JQ086376_Siphoviridae_Lambdavirus:0.171011,JQ
182735_Siphoviridae_Lambdavirus:0.171011)83:0.090173,EU078592_Siphovir
idae_Lambdavirus:0.261184)100:0.442844):0.039611,(HQ711985_Siphovirida
e_D3virus:0.146743,AF165214_Siphoviridae_D3virus:0.146743)100:0.596895
):0.028656):0.111171):0.029289,(((((DQ873690_Siphoviridae_D3112virus:0
.029689,JN808773_Siphoviridae_D3112virus:0.029689)96:0.02718,EU272037_
Siphoviridae_D3112virus:0.056869)93:0.06866,DQ631426_Siphoviridae_D311
2virus:0.125529)100:0.120832,(((EU272036_Siphoviridae_D3112virus:0.050
27,AY394005_Siphoviridae_D3112virus:0.05027)74:0.023958,HE584812_Sipho
viridae_D3112virus:0.074228)82:0.032405,HM624080_Siphoviridae_D3112vir
us:0.106632)100:0.139728)100:0.474064,KP010268_Myoviridae_Muvirus:0.72
0425)90:0.192331):0.029266,(((KR935217_Siphoviridae_Cronusvirus:0.8321
45,(((FN297812_Myoviridae_Vhmlvirus:0.09287,JX556417_Myoviridae_Vhmlvi

rus:0.09287)85:0.040481,AY133112_Myoviridae_Vhmlvirus:0.133351)100:0.5
97184,(KM360178_Myoviridae_Cvm10virus:0.14301,GU903191_Myoviridae_Cvm1
0virus:0.14301)100:0.587525)77:0.10161):0.077299,((KC821609_Podovirida
e_Cba41virus:0.020668,KC821632_Podoviridae_Cba41virus:0.020668)100:0.8
71919,((JN939332_Podoviridae_Prtbvirus:0.084353,JN939331_Podoviridae_P
rtbvirus:0.084353)100:0.755365,(KR136259_Siphoviridae_P12002virus:0.11
2211,KR136260_Siphoviridae_P12002virus:0.112211)100:0.727507)78:0.0528
7):0.016856):0.002508,(((AP013057_Myoviridae_Msw3virus:0.129519,AB7672
44_Myoviridae_Msw3virus:0.129519)100:0.670676,(KC139649_Podoviridae_No
nanavirus:0.233179,KJ802832_Podoviridae_Nonanavirus:0.233179)100:0.567
015):0.094271,(((KC821619_Siphoviridae_Cba181virus:0.146079,KC821617_S
iphoviridae_Cba181virus:0.146079):0.024421,KC821613_Siphoviridae_Cba18
1virus:0.1705)100:0.648928,(JQ823122_Siphoviridae_P12024virus:0.071126
,JQ823123_Siphoviridae_P12024virus:0.071126)100:0.748302)86:0.075038):
0.017486):0.03007):0.009464,(((((((EU311208_Podoviridae_Nona33virus:0
.061031,AF125520_Podoviridae_Nona33virus:0.061031)74:0.044181,HM208303
_Podoviridae_Nona33virus:0.105212):0.022321,AP005154_Podoviridae_Nona3
3virus:0.127533)98:0.059157,KP682381_Podoviridae_Nona33virus:0.18669)9
3:0.055309,(KJ603229_Podoviridae_Pocjvirus:0.045107,KF766125_Podovirid
ae_Pocjvirus:0.045107)100:0.196892):0.006149,(((KR781488_Podoviridae_T
l2011virus:0.074192,JQ011318_Podoviridae_Tl2011virus:0.074192)84:0.022
393,KF971864_Podoviridae_Tl2011virus:0.096585):0.018207,KP682371_Podov
iridae_Tl2011virus:0.114791)100:0.133357)100:0.594594,(((JX104231_Podo
viridae_Bcep22virus:0.166307,AY349011_Podoviridae_Bcep22virus:0.166307
):0.041908,(JN662425_Podoviridae_Bcep22virus:0.069274,FJ937737_Podovir
idae_Bcep22virus:0.069274)100:0.138941)100:0.606941,(KC262634_Podoviri
dae_F116virus:0.13597,AY625898_Podoviridae_F116virus:0.13597)100:0.679
186)70:0.027587)78:0.072343,(LT714109_Podoviridae_P22virus:0.703411,JN
391180_Podoviridae_Epsilon15virus:0.703411)84:0.211675):0.036399):0.00
3285,(((AY510084_Podoviridae_Vp5virus:0.019705,AY505112_Podoviridae_Vp
5virus:0.019705)100:0.022407,JF712866_Podoviridae_Vp5virus:0.042113)10
0:0.886991,(LC102729_Podoviridae_Kpp25virus:0.022426,AB910393_Podoviri
dae_Kpp25virus:0.022426)100:0.906677):0.025667):0.004902,(((((((AY299
121_Siphoviridae_Xp10virus:0.069217,DQ777876_Siphoviridae_Xp10virus:0.
069217)100:0.039732,AP008979_Siphoviridae_Xp10virus:0.108949)100:0.151
159,(EU717894_Siphoviridae_Xp10virus:0.166377,AB720063_Siphoviridae_Xp
10virus:0.166377)99:0.093731)100:0.388372,((KF626667_Podoviridae_Prado
virus:0.111959,KU595433_Podoviridae_Pradovirus:0.111959)82:0.038665,KU
595432_Podoviridae_Pradovirus:0.150624)100:0.497856)81:0.090398,((((((
KX211991_Podoviridae_Kp34virus:0.055239,KT964103_Podoviridae_Kp34virus
:0.055239)71:0.042839,KT367886_Podoviridae_Kp34virus:0.098078):0.02371
5,(KU666550_Podoviridae_Kp34virus:0.059632,AB716666_Podoviridae_Kp34vi
rus:0.059632)98:0.06216):0.010818,((KP708985_Podoviridae_Kp34virus:0.0
91171,KF765493_Podoviridae_Kp34virus:0.091171):0.014025,GQ413938_Podov
iridae_Kp34virus:0.105195):0.027415):0.014586,KP708986_Podoviridae_Kp3
4virus:0.147196)100:0.391223,((((KT388103_Podoviridae_Fri1virus:0.0746
75,KT388102_Podoviridae_Fri1virus:0.074675):0.01533,(HQ186308_Podoviri
dae_Fri1virus:0.029055,JX658790_Podoviridae_Fri1virus:0.029055)98:0.06
095):0.0235,(KT804908_Podoviridae_Fri1virus:0.097478,KR149290_Podoviri
dae_Fri1virus:0.097478):0.016027)100:0.130016,KC311669_Podoviridae_Fri
1virus:0.243521)100:0.294898)100:0.20046)78:0.056035,((((LN866626_Podo
viridae_Kp32virus:0.023563,HM480846_Podoviridae_Kp32virus:0.023563)97:

0.092569,EU734173_Podoviridae_Kp32virus:0.116132):0.025246,KT367885_Po
doviridae_Kp32virus:0.141378):0.012588,(GQ413937_Podoviridae_Kp32virus
:0.106261,KR149291_Podoviridae_Kp32virus:0.106261)82:0.047705)100:0.64
0947)88:0.117959,(((((JX867715_Podoviridae_Phieco32virus:0.018843,KP30
8307_Podoviridae_Phieco32virus:0.018843)100:0.057119,KM044272_Podoviri
dae_Phieco32virus:0.075962)80:0.02109,JX415536_Podoviridae_Phieco32vir
us:0.097052):0.00925,KF981730_Podoviridae_Phieco32virus:0.106302)100:0
.613458,(((KC294142_Podoviridae_Luz24virus:0.051393,KF856712_Podovirid
ae_Luz24virus:0.051393)73:0.031627,KP233880_Podoviridae_Luz24virus:0.0
83021):0.019002,(HG518155_Podoviridae_Luz24virus:0.045306,LN610578_Pod
oviridae_Luz24virus:0.045306)98:0.056717)100:0.617737)100:0.193112)70:
0.027759,((EU100884_Siphoviridae_P23virus:0.012291,EU100883_Siphovirid
ae_P23virus:0.012291)100:0.877936,KT151955_Siphoviridae_Jenstvirus:0.8
90227):0.050404):0.019041):0.006525):0.012343,((((AB731695_Podoviridae
_Una961virus:0.042729,AB647160_Podoviridae_Una961virus:0.042729)70:0.0
22182,JQ617284_Podoviridae_Una961virus:0.064911)100:0.856511,L13696_Pl
asmaviridae_Plasmavirus:0.921421):0.028784,(GQ403789_Sphaerolipovirida
e_Gammasphaerolipovirus:0.343553,AB063393_Sphaerolipoviridae_Gammaspha
erolipovirus:0.343553)100:0.606652):0.028335):0.001955):0.006952):0.00
6572,((((((((((AM087121_Lipothrixviridae_Betalipothrixvirus:0.026687,
AM087120_Lipothrixviridae_Betalipothrixvirus:0.026687)76:0.019806,AM08
7123_Lipothrixviridae_Betalipothrixvirus:0.046493)100:0.144111,EU54565
0_Lipothrixviridae_Betalipothrixvirus:0.190605)99:0.157312,AM087122_Li
pothrixviridae_Betalipothrixvirus:0.347916):0.028737,AF440571_Lipothri
xviridae_Betalipothrixvirus:0.376653)93:0.32858,AJ854042_Lipothrixviri
dae_Deltalipothrixvirus:0.705234)90:0.148649,(((AJ344259_Rudiviridae_R
udivirus:0.085873,AJ414696_Rudiviridae_Rudivirus:0.085873)100:0.248543
,AJ875026_Rudiviridae_Rudivirus:0.334416)100:0.442716,AJ567472_Lipothr
ixviridae_Gammalipothrixvirus:0.777132):0.076751):0.020496,KY780159_Po
rtogloboviridae_Alphaportoglobovirus:0.874379)93:0.062204,EF432053_Amp
ullaviridae_Ampullavirus:0.936583)70:0.010371,(GU080336_Turriviridae_A
lphaturrivirus:0.263182,AY569307_Turriviridae_Alphaturrivirus:0.263182
)100:0.683772)70:0.035331,((((((AY388628_Fuselloviridae_Alphafusellov
irus:0.203844,AY370762_Fuselloviridae_Alphafusellovirus:0.203844):0.02
0621,(EU030939_Fuselloviridae_Alphafusellovirus:0.01084,EU030938_Fusel
loviridae_Alphafusellovirus:0.01084)100:0.213625):0.079354,X07234_Fuse
lloviridae_Alphafusellovirus:0.303818):0.067242,(AY423772_Fusellovirid
ae_Alphafusellovirus:0.314772,FJ870916_Fuselloviridae_Alphafusellovir u
s:0.314772):0.056288):0.171223,(FJ870915_Fuselloviridae_Betafusellovir
us:0.373791,FJ870917_Fuselloviridae_Betafusellovirus:0.373791):0.16849
2)100:0.368901,HE580237_Guttaviridae_Betaguttavirus:0.911185)100:0.046
056,AJ888457_Bicaudaviridae_Bicaudavirus:0.95724):0.025044):0.011734):
0.001688):0.002015,(((((AY701338_Tectiviridae_Betatectivirus:0.125514,
AY257527_Tectiviridae_Betatectivirus:0.125514)94:0.137046,EU408779_Tec
tiviridae_Betatectivirus:0.26256):0.137881,KF188458_Tectiviridae_Betat
ectivirus:0.400441)100:0.560832,(AY848689_Tectiviridae_Alphatectivirus
:0.011393,AY848686_Tectiviridae_Alphatectivirus:0.011393)100:0.94988):
0.015282,(LK392619_Podoviridae_Cp1virus:0.081936,Z47794_Podoviridae_Cp
1virus:0.081936)100:0.89462)73:0.021166):0.00021,((X14855_Tristromavir
idae_Alphatristromavirus:0.786397,KU307456_Tristromaviridae_Alphatrist
romavirus:0.786397)100:0.210695,((AB290007/AB290006/AB290005/..._Polyd
naviridae_Ichnovirus:0.585324,AJ632330/AJ632320/AJ632317/..._Polydnavi

```
ridae_Bracovirus:0.585324)99:0.293818,(AB291209/AB291208/AB291207/..._
Polydnaviridae_Ichnovirus:0.327954,AY029400/AF004378/U41655/..._Polydn
aviridae_Ichnovirus:0.327954)100:0.551188)100:0.117951):0.00084):0.001
075,AF155037_Corticoviridae_Corticovirus:0.999007):6.6e-
05,((((AY950802_Sphaerolipoviridae_Alphasphaerolipovirus:0.135759,KC25
2997_Sphaerolipoviridae_Alphasphaerolipovirus:0.135759)95:0.192837,KT8
09302_Sphaerolipoviridae_Alphasphaerolipovirus:0.328596):0.047369,JN96
8479_Sphaerolipoviridae_Alphasphaerolipovirus:0.375964)100:0.552479,AY
048850_Sphaerolipoviridae_Betasphaerolipovirus:0.928444)100:0.07063)92
:0.00085,(AY722806_Globuloviridae_Globulovirus:0.719259,AJ635161_Globu
loviridae_Globulovirus:0.719259)100:0.280664)81:7.7e-
05,AB537968_Clavaviridae_Clavavirus:1.0);
```

Figure S2 UPGMA dendrogram of Group I dsDNA viruses

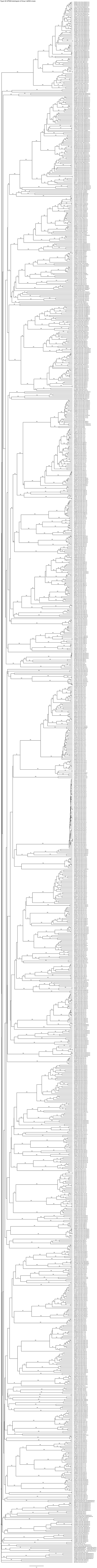

Figure S3 UPGMA dendrogram of Group II ssDNA viruses (NEWICK format)

(((((((FJ410797_Parvoviridae_Hepandensovirus:0.039353,DQ002873_Parvov
iridae_Hepandensovirus:0.039353)82:0.046006,GU371276_Parvoviridae_Hepa
ndensovirus:0.085358)100:0.807221,(((EU233812_Parvoviridae_Brevidenso
virus:0.006911,FJ360744_Parvoviridae_Brevidensovirus:0.006911)74:0.006
409,GU452720_Parvoviridae_Brevidensovirus:0.01332)100:0.050569,X74945_
Parvoviridae_Brevidensovirus:0.063889)99:0.80302,((((((((((((JF50469
9_Parvoviridae_Tetraparvovirus:0.016869,KT225725_Parvoviridae_Tetrapar
vovirus:0.016869)84:0.004515,(KC992732_Parvoviridae_Unassigned:0.00882
5,EU200677_Parvoviridae_Tetraparvovirus:0.008825)100:0.012559)95:0.010
895,AY622943_Parvoviridae_Tetraparvovirus:0.032279)84:0.018442,JQ03775
3_Parvoviridae_Tetraparvovirus:0.05072)99:0.12828,(GU938300_Parvovirid
ae_Tetraparvovirus:0.008445,KM926355_Parvoviridae_Protoparvovirus:0.00
8445)100:0.170555):0.03128,KP120516_Parvoviridae_Unassigned:0.21028):0
.118337,(((((AY386330_Parvoviridae_Erythroparvovirus:0.002588,AF16227
3_Parvoviridae_Erythroparvovirus:0.002588)100:0.017782,AJ249437_Parvov
iridae_Erythroparvovirus:0.020369)100:0.0402,U26342_Parvoviridae_Eryth
roparvovirus:0.060569)93:0.076678,GQ200736_Parvoviridae_Erythroparvovi
rus:0.137247)75:0.102014,JQ037754_Parvoviridae_Unassigned:0.239261):0.
018994,AF406967_Parvoviridae_Erythroparvovirus:0.258255):0.070362):0.0
23414,(((((AY388617_Parvoviridae_Dependoparvovirus:0.023871,AF085716_
Parvoviridae_Dependoparvovirus:0.023871)89:0.015893,JN420372_Parvoviri
dae_Dependoparvovirus:0.039764):0.009181,(U25749_Parvoviridae_Dependop
arvovirus:0.011486,U22967_Parvoviridae_Dependoparvovirus:0.011486)100:
0.037459):0.004762,((AY186198_Parvoviridae_Dependoparvovirus:0.029876,
AY629583_Parvoviridae_Dependoparvovirus:0.029876)71:0.011632,GU226971_
Parvoviridae_Dependoparvovirus:0.041508):0.012199):0.014303,((((AF513
851_Parvoviridae_Dependoparvovirus:0.007334,AF063497_Parvoviridae_Depe
ndoparvovirus:0.007334)95:0.006283,AF513852_Parvoviridae_Dependoparvov
irus:0.013618):0.002708,U48704_Parvoviridae_Dependoparvovirus:0.016325
)86:0.010053,AF043303_Parvoviridae_Dependoparvovirus:0.026378)80:0.010
378,U89790_Parvoviridae_Dependoparvovirus:0.036756)76:0.031254)98:0.04
1505,(AY349010_Parvoviridae_Dependoparvovirus:0.032201,KP733794_Parvov
iridae_Dependoparvovirus:0.032201)100:0.077315)99:0.242515):0.027,((((
JX896321_Parvoviridae_Protoparvovirus:0.106938,GQ387499_Parvoviridae_C
opiparvovirus:0.106938)80:0.071861,AF406966_Parvoviridae_Copiparvoviru
s:0.178799):0.05046,KM035804_Parvoviridae_Unassigned:0.229259):0.04806
8,KF999685_Parvoviridae_Protoparvovirus:0.277327)82:0.101703):0.028497
,((((((((((KF792837_Parvoviridae_Bocaparvovirus:0.025448,JN648103_Par
voviridae_Bocaparvovirus:0.025448):0.000869,JQ692585_Parvoviridae_Boca
parvovirus:0.026317):0.004961,((JN420365_Parvoviridae_Bocaparvovirus:0
.018184,JN420361_Parvoviridae_Bocaparvovirus:0.018184)89:0.006349,AF49
5467_Parvoviridae_Bocaparvovirus:0.024534):0.006744):0.00505,KU950356_
Parvoviridae_Bocaparvovirus:0.036328):0.001299,(((DQ000496_Parvovirida
e_Bocaparvovirus:0.009397,EU918736_Parvoviridae_Bocaparvovirus:0.00939
7)96:0.004523,(FJ170278_Parvoviridae_Bocaparvovirus:0.009005,FJ973561_
Parvoviridae_Bocaparvovirus:0.009005)99:0.004915)94:0.005468,HM145750_
Parvoviridae_Bocaparvovirus:0.019388)98:0.01824):0.004148,(KU321655_Pa
rvoviridae_Bocaparvovirus:0.037266,KT592508_Parvoviridae_Bocaparvoviru
s:0.037266):0.004509):0.007485,HM053693_Parvoviridae_Bocaparvovirus:0.
04926):0.01085,((KU172421_Parvoviridae_Bocaparvovirus:0.038477,M14363_

Parvoviridae_Bocaparvovirus:0.038477)79:0.017371,KT454512_Parvoviridae
_Bocaparvovirus:0.055849):0.004262):0.017109,(((JN831651_Parvoviridae_
Bocaparvovirus:0.017726,JF429835_Parvoviridae_Bocaparvovirus:0.017726)
:0.002987,(JF429834_Parvoviridae_Bocaparvovirus:0.016778,KF360033_Parv
oviridae_Bocaparvovirus:0.016778)80:0.003935)100:0.050795,KP729195_Par
voviridae_Bocaparvovirus:0.071508):0.005712)75:0.038204,HQ223038_Parvo
viridae_Bocaparvovirus:0.115424)100:0.263713,((GU214705_Parvoviridae_P
rotoparvovirus:0.024008,GU214706_Parvoviridae_Aveparvovirus:0.024008)7
3:0.013556,GU214704_Parvoviridae_Aveparvovirus:0.037564)100:0.341573):
0.028389):0.085546,(((((KU561552_Parvoviridae_Unassigned:0.042221,LC08
5675_Parvoviridae_Protoparvovirus:0.042221):0.002399,AB937988_Parvovir
idae_Protoparvovirus:0.04462)88:0.035562,(AB847987_Parvoviridae_Unassi
gned:0.017342,JX027296_Parvoviridae_Protoparvovirus:0.017342)100:0.062
84)79:0.035205,(((J02275_Parvoviridae_Protoparvovirus:0.037986,M19296_
Parvoviridae_Protoparvovirus:0.037986):0.012775,L23427_Parvoviridae_Pr
otoparvovirus:0.050761):0.011684,(AF036710_Parvoviridae_Protoparvoviru
s:0.031121,X01457_Parvoviridae_Protoparvovirus:0.031121)81:0.031324)98
:0.052942)100:0.270545,((KJ396347_Parvoviridae_Amdoparvovirus:0.032584
,JN202450_Parvoviridae_Amdoparvovirus:0.032584):0.010825,M20036_Parvov
iridae_Amdoparvovirus:0.043409)100:0.342522):0.107141)100:0.272359,(KX
907333_Parvoviridae_Unassigned:0.669348,AF218266_Parvoviridae_Penstyld
ensovirus:0.669348):0.096084):0.023485,((((((((((AY461507_Parvovirid
ae_Ambidensovirus:0.008457,S47266_Parvoviridae_Ambidensovirus:0.008457
)95:0.003597,L32896_Parvoviridae_Ambidensovirus:0.012054)96:0.012714,J
X645046_Parvoviridae_Ambidensovirus:0.024768):0.004038,AF036333_Parvov
iridae_Ambidensovirus:0.028806)100:0.168177,FJ810126_Parvoviridae_Ambi
densovirus:0.196983)95:0.216686,(((AX344110_Parvoviridae_Ambidensoviru
s:0.1882,KC991097_Parvoviridae_Ambidensovirus:0.1882):0.040822,AY03288
2_Parvoviridae_Ambidensovirus:0.229022)88:0.129225,AF192260_Parvovirid
ae_Ambidensovirus:0.358247):0.055422):0.071046,(KP410261_Parvoviridae_
Ambidensovirus:0.143393,KM052275_Parvoviridae_Ambidensovirus:0.143393)
99:0.341322):0.010787,(KU727766_Parvoviridae_Unassigned:0.248404,AY189
948_Parvoviridae_Ambidensovirus:0.248404)99:0.247099):0.004005,(AY1481
87_Parvoviridae_Ambidensovirus:0.288195,FJ040397_Parvoviridae_Ambidens
ovirus:0.288195)91:0.211313):0.060506,KX165268_Parvoviridae_Ambidensov
irus:0.560014):0.033412,KF275669_Parvoviridae_Unassigned:0.593426)86:0
.0722,((((AF375296_Parvoviridae_Iteradensovirus:0.014137,KF963252_Par
voviridae_Iteradensovirus:0.014137)73:0.007546,JX020762_Parvoviridae_I
teradensovirus:0.021684)97:0.02045,(JX110122_Parvoviridae_Iteradensovi
rus:0.033761,AB042597_Parvoviridae_Iteradensovirus:0.033761):0.008373)
100:0.068649,AY665654_Parvoviridae_Iteradensovirus:0.110783)97:0.16312
2,HQ613271_Parvoviridae_Iteradensovirus:0.273905)100:0.39172)92:0.1232
91):0.039349,'EU623082/EU623083 Bidnaviridae
Bidensovirus':0.828266):0.038643):0.02567)100:0.102305,(((((((((((((
((((((((((((((((((((((KT322141_Geminiviridae_Begomovirus:0.018261,A
F414287_Geminiviridae_Begomovirus:0.018261):0.003635,HQ162270_Geminivi
ridae_Begomovirus:0.021896):0.000654,DQ641698_Geminiviridae_Begomoviru
s:0.02255):0.002659,(((FN256261_Geminiviridae_Begomovirus:0.003296,FN4
34083_Geminiviridae_Begomovirus:0.003296)100:0.012286,AJ558117_Geminiv
iridae_Begomovirus:0.015582)79:0.005857,GQ373254_Geminiviridae_Begomov
irus:0.02144):0.003769):0.005309,AM183224_Geminiviridae_Begomovirus:0.
030518):0.003388,(((AY602166_Geminiviridae_Begomovirus:0.024963,AY6021

65_Geminiviridae_Begomovirus:0.024963):0.000476,DQ641695_Geminiviridae
_Begomovirus:0.025439):0.003223,AF264063_Geminiviridae_Begomovirus:0.0
28663):0.005243):0.005289,(FN297834_Geminiviridae_Begomovirus:0.034365
,AJ558124_Geminiviridae_Begomovirus:0.034365):0.00483):0.003447,(((U88
692_Geminiviridae_Begomovirus:0.014106,GU723730_Geminiviridae_Begomovi
rus:0.014106):0.001556,JQ867093_Geminiviridae_Begomovirus:0.015662)80:
0.011447,DQ866128_Geminiviridae_Begomovirus:0.027108)71:0.015534):0.00
4208,(((((JQ804985_Geminiviridae_Begomovirus:0.004009,JX972141_Geminiv
iridae_Begomovirus:0.004009)100:0.026418,AJ851005_Geminiviridae_Begomo
virus:0.030427):0.001627,AJ558125_Geminiviridae_Begomovirus:0.032055):
0.004489,EU862323_Geminiviridae_Begomovirus:0.036543):0.00808,(((DQ641
696_Geminiviridae_Begomovirus:0.029533,AM048837_Geminiviridae_Begomovi
rus:0.029533):0.002965,KC686705_Geminiviridae_Begomovirus:0.032497):0.
006878,DQ641697_Geminiviridae_Begomovirus:0.039375):0.005249):0.002227
):0.005736,(((((AM980509_Geminiviridae_Begomovirus:0.020897,AJ319675_G
eminiviridae_Begomovirus:0.020897)92:0.012981,AJ457985_Geminiviridae_B
egomovirus:0.033878):0.002538,EF011559_Geminiviridae_Begomovirus:0.036
417):0.002934,AJ319674_Geminiviridae_Begomovirus:0.039351):0.00856,(DQ
256460_Geminiviridae_Begomovirus:0.031258,AF311734_Geminiviridae_Begom
ovirus:0.031258)81:0.016652):0.004676):0.005658,(((((((GU076454_Gemin
iviridae_Begomovirus:0.017649,EU635776_Geminiviridae_Begomovirus:0.017
649):0.003846,GU076442_Geminiviridae_Begomovirus:0.021495):0.006864,(L
M651400_Geminiviridae_Begomovirus:0.01113,AY502934_Geminiviridae_Begom
ovirus:0.01113)100:0.017229):0.004586,DQ358913_Geminiviridae_Begomovir
us:0.032945):0.007186,(((AJ865340_Geminiviridae_Begomovirus:0.018104,A
J865341_Geminiviridae_Begomovirus:0.018104)94:0.010468,AM701763_Gemini
viridae_Begomovirus:0.028572):0.005151,AM701764_Geminiviridae_Begomovi
rus:0.033723):0.006408):0.007011,((AJ489258_Geminiviridae_Begomovirus:
0.028698,AJ132711_Geminiviridae_Begomovirus:0.028698)72:0.015545,X7631
9_Geminiviridae_Begomovirus:0.044243):0.002898):0.00571,(((AY502936_G
eminiviridae_Begomovirus:0.039319,AY502935_Geminiviridae_Begomovirus:0
.039319):0.001171,AF261885_Geminiviridae_Begomovirus:0.040489):0.00363
5,(AM701768_Geminiviridae_Begomovirus:0.03434,EF194760_Geminiviridae_B
egomovirus:0.03434):0.009785):0.005352,KU522485_Geminiviridae_Begomovi
rus:0.049476):0.003375):0.001973,((((KC763630_Geminiviridae_Begomovir
us:0.019434,AY044137_Geminiviridae_Begomovirus:0.019434):0.005815,AY04
4139_Geminiviridae_Begomovirus:0.02525):0.006814,EF110891_Geminivirida
e_Begomovirus:0.032064):0.003878,KF040453_Geminiviridae_Begomovirus:0.
035942):0.013657,((S53251_Geminiviridae_Begomovirus:0.017125,AF084006_
Geminiviridae_Begomovirus:0.017125)100:0.028463,JX183732_Geminiviridae
_Begomovirus:0.045587):0.004012):0.005225):0.00342):0.002543,((((((((
((((X74516_Geminiviridae_Begomovirus:0.008687,AB306314_Geminiviridae_B
egomovirus:0.008687)100:0.012307,EU487045_Geminiviridae_Begomovirus:0.
020994):0.003909,AB100305_Geminiviridae_Begomovirus:0.024903):0.002362
,KC577540_Geminiviridae_Begomovirus:0.027265):0.004471,((EU487046_Gemi
niviridae_Begomovirus:0.021268,EU487047_Geminiviridae_Begomovirus:0.02
1268):0.007072,KJ744212_Geminiviridae_Begomovirus:0.02834):0.003397):0
.003398,(AJ495814_Geminiviridae_Begomovirus:0.02767,JN809816_Geminivir
idae_Begomovirus:0.02767):0.007465):0.005265,((KC172826_Geminiviridae_
Begomovirus:0.026617,AJ495813_Geminiviridae_Begomovirus:0.026617):0.00
6516,DQ866124_Geminiviridae_Begomovirus:0.033133):0.007266):0.003251,K
P188831_Geminiviridae_Begomovirus:0.04365):0.000533,(AF327436_Geminivi

ridae_Begomovirus:0.035976,AF195782_Geminiviridae_Begomovirus:0.035976
):0.008208):0.003398,DQ866132_Geminiviridae_Begomovirus:0.047582):0.00
5637,AB050781_Geminiviridae_Begomovirus:0.053219):0.002406,(AB162141_G
eminiviridae_Begomovirus:0.030551,AB100304_Geminiviridae_Begomovirus:0
.030551)79:0.025073):0.002585,(JQ793786_Geminiviridae_Begomovirus:0.05
2237,EF602306_Geminiviridae_Begomovirus:0.052237):0.005973):0.002577):
0.004109,(((JN809819_Geminiviridae_Begomovirus:0.037164,DQ641701_Gemin
iviridae_Begomovirus:0.037164):0.014883,DQ641699_Geminiviridae_Begomov
irus:0.052047):0.008193,(AJ876550_Geminiviridae_Begomovirus:0.0558,AJ9
71263_Geminiviridae_Begomovirus:0.0558):0.00444):0.004655):0.003048,((
((((((FN554540_Geminiviridae_Begomovirus:0.009179,EU432373_Geminiviri
dae_Begomovirus:0.009179)100:0.015024,EU024120_Geminiviridae_Begomovir
us:0.024203)90:0.00998,AF155064_Geminiviridae_Begomovirus:0.034183):0.
004659,AM701757_Geminiviridae_Begomovirus:0.038842):0.003925,((AF01488
1_Geminiviridae_Begomovirus:0.010105,AY036009_Geminiviridae_Begomoviru
s:0.010105)100:0.01731,AJ542539_Geminiviridae_Begomovirus:0.027415)70:
0.015352):0.006424,FM210276_Geminiviridae_Begomovirus:0.049191):0.0063
21,FJ868828_Geminiviridae_Begomovirus:0.055513):0.005582,(((EU585781_G
eminiviridae_Begomovirus:0.044053,FN396966_Geminiviridae_Begomovirus:0
.044053):0.009972,HE862273_Geminiviridae_Begomovirus:0.054025):0.00195
6,AM701765_Geminiviridae_Begomovirus:0.055981):0.005114):0.005253,((KU
852742_Geminiviridae_Begomovirus:0.026745,JQ866297_Geminiviridae_Begom
ovirus:0.026745)99:0.016602,(KC223600_Geminiviridae_Begomovirus:0.0082
39,KC196077_Geminiviridae_Begomovirus:0.008239)100:0.035108)86:0.02300
1):0.001595):0.001267,((((((DQ852623_Geminiviridae_Begomovirus:0.02193
5,AF165098_Geminiviridae_Begomovirus:0.021935):0.00515,AY754814_Gemini
viridae_Begomovirus:0.027085):0.00318,KF663698_Geminiviridae_Begomovir
us:0.030265):0.001985,(Z48182_Geminiviridae_Begomovirus:0.023716,AF295
401_Geminiviridae_Begomovirus:0.023716):0.008535):0.012016,KM880103_Ge
miniviridae_Begomovirus:0.044266):0.019875,(AM182232_Geminiviridae_Beg
omovirus:0.052293,AF274349_Geminiviridae_Begomovirus:0.052293):0.01184
9):0.005069):0.001462,((((((((JX972142_Geminiviridae_Begomovirus:0.013
574,AJ704603_Geminiviridae_Begomovirus:0.013574)99:0.017002,AM411424_G
eminiviridae_Begomovirus:0.030576):0.002777,(JF681158_Geminiviridae_Be
gomovirus:0.001504,AJ558118_Geminiviridae_Begomovirus:0.001504)100:0.0
31849):0.007694,DQ871221_Geminiviridae_Begomovirus:0.041047):0.002912,
(KF150142_Geminiviridae_Begomovirus:0.037785,AM236784_Geminiviridae_Be
gomovirus:0.037785):0.006174):0.00872,((AY297924_Geminiviridae_Begomov
irus:0.033318,KU933258_Geminiviridae_Begomovirus:0.033318):0.009316,FJ
589571_Geminiviridae_Begomovirus:0.042635):0.010044):0.00384,((((AJ49
5812_Geminiviridae_Begomovirus:0.021521,AF206674_Geminiviridae_Begomov
irus:0.021521):0.006419,AJ786711_Geminiviridae_Begomovirus:0.02794):0.
007631,(DQ629101_Geminiviridae_Begomovirus:0.029079,EF165536_Geminivir
idae_Begomovirus:0.029079):0.006492):0.008468,(AY514632_Geminiviridae_
Begomovirus:0.025888,AY514630_Geminiviridae_Begomovirus:0.025888)91:0.
018152):0.010106,(AJ512761_Geminiviridae_Begomovirus:0.049294,LC091538
_Geminiviridae_Begomovirus:0.049294):0.004852):0.002373):0.006114,((((
FR715681_Geminiviridae_Begomovirus:0.026379,FR772081_Geminiviridae_Beg
omovirus:0.026379)84:0.013821,KX698087_Geminiviridae_Begomovirus:0.040
2):0.006835,GU111998_Geminiviridae_Begomovirus:0.047036):0.004558,EF37
3060_Geminiviridae_Begomovirus:0.051594):0.011039):0.008039):0.001599,
((((JF694486_Geminiviridae_Begomovirus:0.042849,KF414123_Geminiviridae

_Begomovirus:0.042849):0.009582,EU377539_Geminiviridae_Begomovirus:0.0
52431):0.008002,DQ641694_Geminiviridae_Begomovirus:0.060433):0.005914,
AF189018_Geminiviridae_Begomovirus:0.066348):0.005923):0.003636,((((((
(((((((DQ116884_Geminiviridae_Begomovirus:0.022711,AM948961_Geminivir
idae_Begomovirus:0.022711):0.003801,AM712436_Geminiviridae_Begomovirus
:0.026512):0.003673,(EF175733_Geminiviridae_Begomovirus:0.027615,FN543
099_Geminiviridae_Begomovirus:0.027615):0.002571):0.004821,LK028571_Ge
miniviridae_Begomovirus:0.035006):0.001671,(((AY754812_Geminiviridae_B
egomovirus:0.01167,U38239_Geminiviridae_Begomovirus:0.01167)97:0.01102
4,FJ514798_Geminiviridae_Begomovirus:0.022694):0.008183,(KC952005_Gemi
niviridae_Begomovirus:0.024311,KP195260_Geminiviridae_Begomovirus:0.02
4311):0.006566):0.0058):0.002651,(KT962229_Geminiviridae_Begomovirus:0
.030861,AJ437618_Geminiviridae_Begomovirus:0.030861):0.008468):0.00315
4,((((AJ875159_Geminiviridae_Begomovirus:0.017863,DQ673859_Geminivirid
ae_Begomovirus:0.017863)81:0.009136,AF188481_Geminiviridae_Begomovirus
:0.026998):0.00423,(FJ345402_Geminiviridae_Begomovirus:0.025705,DQ1168
78_Geminiviridae_Begomovirus:0.025705):0.005524):0.002015,(GQ994095_Ge
miniviridae_Begomovirus:0.023619,AJ457986_Geminiviridae_Begomovirus:0.
023619):0.009625):0.009239):0.006255,((FN552001_Geminiviridae_Begomovi
rus:0.023144,AJ002449_Geminiviridae_Begomovirus:0.023144):0.012826,(HF
549182_Geminiviridae_Begomovirus:0.018899,AM421522_Geminiviridae_Begom
ovirus:0.018899)87:0.017071):0.012767):0.004149,((GU136803_Geminivirid
ae_Begomovirus:0.016184,AF336806_Geminiviridae_Begomovirus:0.016184)10
0:0.023447,HM149260_Geminiviridae_Begomovirus:0.039631):0.013255):0.00
265,GU732204_Geminiviridae_Begomovirus:0.055537):0.002805,DQ339117_Gem
iniviridae_Begomovirus:0.058342):0.002018,(((((FN806779_Geminiviridae_
Begomovirus:0.025577,FN386459_Geminiviridae_Begomovirus:0.025577):0.00
6695,JQ897969_Geminiviridae_Begomovirus:0.032272)72:0.017866,FN552749_
Geminiviridae_Begomovirus:0.050138):0.003869,(AJ558122_Geminiviridae_B
egomovirus:0.022917,AM236779_Geminiviridae_Begomovirus:0.022917)99:0.0
31089):0.001412,(EU910141_Geminiviridae_Begomovirus:0.04463,AJ558121_G
eminiviridae_Begomovirus:0.04463):0.010789):0.004941):0.004285,((((FR7
72082_Geminiviridae_Begomovirus:0.026663,GQ478343_Geminiviridae_Begomo
virus:0.026663)72:0.009065,AY705380_Geminiviridae_Begomovirus:0.035728
):0.002931,AM236755_Geminiviridae_Begomovirus:0.03866):0.007948,((AJ45
7824_Geminiviridae_Begomovirus:0.034351,EU366903_Geminiviridae_Begomov
irus:0.034351):0.00483,AF314144_Geminiviridae_Begomovirus:0.039181):0.
007426):0.018038):0.005165,(((HM140367_Geminiviridae_Begomovirus:0.050
638,FJ177030_Geminiviridae_Begomovirus:0.050638):0.002401,(JF496657_Ge
miniviridae_Begomovirus:0.025812,AJ575819_Geminiviridae_Begomovirus:0.
025812)96:0.027228):0.009347,(HM230683_Geminiviridae_Begomovirus:0.047
905,KM023146_Geminiviridae_Begomovirus:0.047905):0.014482):0.007424):0
.006097):0.006084,((((EU847739_Geminiviridae_Begomovirus:0.030922,EU35
0585_Geminiviridae_Begomovirus:0.030922)77:0.010622,FJ685621_Geminivir
idae_Begomovirus:0.041545):0.010067,((FR873228_Geminiviridae_Begomovir
us:0.010205,FR717144_Geminiviridae_Begomovirus:0.010205)100:0.026266,F
M210278_Geminiviridae_Begomovirus:0.03647)82:0.015141):0.01169,(((AJ86
5339_Geminiviridae_Begomovirus:0.024443,AJ865338_Geminiviridae_Begomov
irus:0.024443)92:0.027856,AF271234_Geminiviridae_Begomovirus:0.052299)
:0.004492,AM701760_Geminiviridae_Begomovirus:0.056791):0.006511):0.018
69):0.002169,KF429251_Geminiviridae_Begomovirus:0.08416):0.001078,(AM7
01761_Geminiviridae_Begomovirus:0.06215,HQ180391_Geminiviridae_Begomov

irus:0.06215):0.023088):0.004849,((((((((((((((((FN543112_Geminivirida
e_Begomovirus:0.015081,FN678906_Geminiviridae_Begomovirus:0.015081)74:
0.006186,AJ436992_Geminiviridae_Begomovirus:0.021267):0.001849,(JN1352
33_Geminiviridae_Begomovirus:0.018887,HM143914_Geminiviridae_Begomovir
us:0.018887):0.004229):0.00275,FM955601_Geminiviridae_Begomovirus:0.02
5866):0.003336,(DQ116881_Geminiviridae_Begomovirus:0.020761,HM007106_G
eminiviridae_Begomovirus:0.020761)78:0.008441):0.000305,FM877858_Gemin
iviridae_Begomovirus:0.029507):0.003555,Y15934_Geminiviridae_Begomovir
us:0.033062):0.004388,(LK028570_Geminiviridae_Begomovirus:0.024238,HE5
80234_Geminiviridae_Begomovirus:0.024238)80:0.013212):0.002759,((AM404
179_Geminiviridae_Begomovirus:0.021859,HM007096_Geminiviridae_Begomovi
rus:0.021859):0.010343,JN135234_Geminiviridae_Begomovirus:0.032203):0.
008006):0.004915,AF314531_Geminiviridae_Begomovirus:0.045123):0.011259
,(HM007121_Geminiviridae_Begomovirus:0.053316,KC202818_Geminiviridae_B
egomovirus:0.053316):0.003067):0.001087,(DQ629102_Geminiviridae_Begomo
virus:0.047957,JQ954859_Geminiviridae_Begomovirus:0.047957):0.009513):
0.005074,((AJ579307_Geminiviridae_Begomovirus:0.049529,LK054801_Gemini
viridae_Begomovirus:0.049529):0.0061,KJ739692_Geminiviridae_Begomoviru
s:0.055629):0.006915):0.002088,((HF912280_Geminiviridae_Begomovirus:0.
047585,JN807765_Geminiviridae_Begomovirus:0.047585):0.005925,(EF408037
_Geminiviridae_Begomovirus:0.034769,KM359406_Geminiviridae_Begomovirus
:0.034769)84:0.018742):0.011121):0.005365,(((KF307208_Geminiviridae_Be
gomovirus:0.008168,FJ593629_Geminiviridae_Begomovirus:0.008168)94:0.00
9162,AJ507777_Geminiviridae_Begomovirus:0.01733)99:0.039753,(AJ420319_
Geminiviridae_Begomovirus:0.047937,KC149938_Geminiviridae_Begomovirus:
0.047937):0.009147):0.012913):0.011549,((((((FJ956700_Geminiviridae_Be
gomovirus:0.030191,KF260965_Geminiviridae_Begomovirus:0.030191):0.0066
96,AM491778_Geminiviridae_Begomovirus:0.036887):0.008678,AM701758_Gemi
niviridae_Begomovirus:0.045565):0.006628,(JN809820_Geminiviridae_Begom
ovirus:0.031721,AF307861_Geminiviridae_Begomovirus:0.031721)94:0.02047
2):0.014313,DQ127170_Geminiviridae_Begomovirus:0.066506):0.012983,KC89
8543_Geminiviridae_Begomovirus:0.079489):0.002056):0.008542):0.004137,
(((((((((AJ002451_Geminiviridae_Begomovirus:0.017823,FJ179372_Geminivi
ridae_Begomovirus:0.017823):0.002705,AF241479_Geminiviridae_Begomoviru
s:0.020528):0.00976,FJ176235_Geminiviridae_Begomovirus:0.030289):0.005
487,GU112023_Geminiviridae_Begomovirus:0.035776):0.005212,((EU365616_G
eminiviridae_Begomovirus:0.030973,AJ132430_Geminiviridae_Begomovirus:0
.030973):0.008171,EU360303_Geminiviridae_Begomovirus:0.039144):0.00184
3):0.009721,(EU365613_Geminiviridae_Begomovirus:0.035276,AJ002447_Gemi
niviridae_Begomovirus:0.035276):0.015432):0.010522,((((GU112081_Gemini
viridae_Begomovirus:0.009631,AJ002455_Geminiviridae_Begomovirus:0.0096
31)100:0.017902,EU384575_Geminiviridae_Begomovirus:0.027532)86:0.02432
6,GU112004_Geminiviridae_Begomovirus:0.051859):0.001899,(EU589392_Gemi
niviridae_Begomovirus:0.044007,FN645923_Geminiviridae_Begomovirus:0.04
4007):0.009751):0.007472):0.009698,(KX831888_Geminiviridae_Begomovirus
:0.035173,AF363011_Geminiviridae_Begomovirus:0.035173)78:0.035755):0.0
16258,((GU112010_Geminiviridae_Begomovirus:0.055859,JN807763_Geminivir
idae_Begomovirus:0.055859):0.016704,FJ515747_Geminiviridae_Begomovirus
:0.072563):0.014623):0.007037):0.003512,(((EU543562_Geminiviridae_Bego
movirus:0.04003,EU487031_Geminiviridae_Begomovirus:0.04003):0.009005,A
M286794_Geminiviridae_Begomovirus:0.049035):0.0146,EF197941_Geminiviri
dae_Begomovirus:0.063635):0.034102):0.002792,(((((((((((((((((HM164545

_Geminiviridae_Begomovirus:0.019127,FJ434943_Geminiviridae_Begomovirus
:0.019127):0.004085,HM164547_Geminiviridae_Begomovirus:0.023212):0.000
528,AB236325_Geminiviridae_Begomovirus:0.02374):0.005677,AB287439_Gemi
niviridae_Begomovirus:0.029417):0.004608,(((AB079689_Geminiviridae_Beg
omovirus:0.0145,AB028604_Geminiviridae_Begomovirus:0.0145)92:0.010505,
AB178946_Geminiviridae_Begomovirus:0.025005):0.005041,KT992046_Geminiv
iridae_Begomovirus:0.030046):0.003979):0.005801,(JX416174_Geminivirida
e_Begomovirus:0.023897,AJ542540_Geminiviridae_Begomovirus:0.023897):0.
015929):0.004543,(AB055009_Geminiviridae_Begomovirus:0.036742,AB178949
_Geminiviridae_Begomovirus:0.036742):0.007627):0.002446,(((AB433979_Ge
miniviridae_Begomovirus:0.020229,AB079766_Geminiviridae_Begomovirus:0.
020229)75:0.006277,AB300463_Geminiviridae_Begomovirus:0.026506):0.0063
12,AJ438936_Geminiviridae_Begomovirus:0.032819):0.013996):0.001124,(((
AB287441_Geminiviridae_Begomovirus:0.014842,AB287440_Geminiviridae_Beg
omovirus:0.014842)94:0.01268,AB182261_Geminiviridae_Begomovirus:0.0275
22):0.01067,AB079765_Geminiviridae_Begomovirus:0.038192):0.009746):0.0
06716,AJ438937_Geminiviridae_Begomovirus:0.054655):0.00366,AM238692_Ge
miniviridae_Begomovirus:0.058315):0.001969,((AB178945_Geminiviridae_Be
gomovirus:0.036706,AB020781_Geminiviridae_Begomovirus:0.036706):0.0077
23,AB007990_Geminiviridae_Begomovirus:0.04443):0.015855):0.00865,AF134
484_Geminiviridae_Begomovirus:0.068935):0.002207,((((KM215610_Geminivi
ridae_Begomovirus:0.028718,EU734831_Geminiviridae_Begomovirus:0.028718
):0.010134,X61153_Geminiviridae_Begomovirus:0.038852):0.005294,AF35033
0_Geminiviridae_Begomovirus:0.044146):0.016498,((HE793424_Geminivirida
e_Begomovirus:0.02723,DQ875879_Geminiviridae_Begomovirus:0.02723)79:0.
009648,FR717137_Geminiviridae_Begomovirus:0.036878)92:0.023766):0.0104
98):0.013899,AJ223191_Geminiviridae_Begomovirus:0.085041):0.000573,(((
(((AB377113_Geminiviridae_Begomovirus:0.00758,AB307731_Geminiviridae_B
egomovirus:0.00758)100:0.014852,AB050597_Geminiviridae_Begomovirus:0.0
22432)76:0.005101,EU487042_Geminiviridae_Begomovirus:0.027533)100:0.03
5895,AJ558119_Geminiviridae_Begomovirus:0.063428):0.012125,AM050730_Ge
miniviridae_Begomovirus:0.075553):0.007517,JX025358_Geminiviridae_Bego
movirus:0.08307):0.002544):0.011535,(((EF544602_Geminiviridae_Begomovi
rus:0.040606,AJ965540_Geminiviridae_Begomovirus:0.040606)72:0.017605,A
J965539_Geminiviridae_Begomovirus:0.058211):0.010044,EF544604_Geminivi
ridae_Begomovirus:0.068255):0.028893):0.00338):0.007708,((FJ455449_Gem
iniviridae_Begomovirus:0.071424,FJ159269_Geminiviridae_Begomovirus:0.0
71424):0.021202,HM991146_Geminiviridae_Begomovirus:0.092626):0.01561):
0.000365,KR612272_Geminiviridae_Begomovirus:0.108601):0.022908,(((JX47
3582_Geminiviridae_Begomovirus:0.092796,JQ407224_Geminiviridae_Begomov
irus:0.092796):0.017247,JN817517_Geminiviridae_Begomovirus:0.110042):0
.013624,FJ210467_Geminiviridae_Begomovirus:0.123667):0.007842):0.02157
8,((((((KX831132_Geminiviridae_Begomovirus:0.076839,GQ472984_Geminivir
idae_Begomovirus:0.076839):0.009721,(FJ237617_Geminiviridae_Begomoviru
s:0.015663,FJ237614_Geminiviridae_Begomovirus:0.015663)100:0.070897):0
.014926,(GU451249_Geminiviridae_Begomovirus:0.053448,EU798996_Geminivi
ridae_Begomovirus:0.053448)97:0.048039):0.00477,AF029217_Geminiviridae
_Begomovirus:0.106257):0.03204,AM259382_Geminiviridae_Begomovirus:0.13
8297):0.008917,(KF551592_Geminiviridae_Begomovirus:0.137797,FM210275_G
eminiviridae_Begomovirus:0.137797):0.009417):0.005872):0.02838,((((((
((((((((((((EU710749_Geminiviridae_Begomovirus:0.026502,JX871385_Gem
iniviridae_Begomovirus:0.026502):0.004854,KU562963_Geminiviridae_Begom

ovirus:0.031356):0.005691,JX162595_Geminiviridae_Begomovirus:0.037047)
:0.00427,(JX415187_Geminiviridae_Begomovirus:0.033454,HM585439_Geminiv
iridae_Begomovirus:0.033454):0.007863):0.003311,(EU518935_Geminivirida
e_Begomovirus:0.035581,AY965897_Geminiviridae_Begomovirus:0.035581):0.
009046):0.002761,((GQ334472_Geminiviridae_Begomovirus:0.037471,EF12175
5_Geminiviridae_Begomovirus:0.037471):0.006068,AJ344452_Geminiviridae_
Begomovirus:0.043539):0.003849):0.004877,((FM160943_Geminiviridae_Bego
movirus:0.037458,JX871376_Geminiviridae_Begomovirus:0.037458):0.004305
,EU710751_Geminiviridae_Begomovirus:0.041763):0.010502):0.002067,((((J
X871380_Geminiviridae_Begomovirus:0.042901,AY751753_Geminiviridae_Bego
movirus:0.042901):0.002767,JX871383_Geminiviridae_Begomovirus:0.045668
):0.002497,((U77963_Geminiviridae_Begomovirus:0.03282,FJ600483_Geminiv
iridae_Begomovirus:0.03282):0.004546,KM887907_Geminiviridae_Begomoviru
s:0.037366):0.010798):0.00186,AJ549960_Geminiviridae_Begomovirus:0.050
025):0.004307):0.001111,JN419002_Geminiviridae_Begomovirus:0.055443):0
.003374,((AJ488768_Geminiviridae_Begomovirus:0.03772,HM585435_Geminivi
ridae_Begomovirus:0.03772):0.010918,FJ944023_Geminiviridae_Begomovirus
:0.048637):0.01018):0.004145,(((FN436001_Geminiviridae_Begomovirus:0.0
37724,JN419021_Geminiviridae_Begomovirus:0.037724):0.0109,JN419013_Gem
iniviridae_Begomovirus:0.048624):0.012223,KX691400_Geminiviridae_Begom
ovirus:0.060847):0.002115):0.009674,(AY090555_Geminiviridae_Begomoviru
s:0.041621,JQ429791_Geminiviridae_Begomovirus:0.041621)95:0.031016):0.
004218,(((JN676150_Geminiviridae_Begomovirus:0.02214,AY339619_Geminivi
ridae_Begomovirus:0.02214):0.009712,AY339618_Geminiviridae_Begomovirus
:0.031852)91:0.028772,JX857691_Geminiviridae_Begomovirus:0.060625):0.0
16229):0.005738,AY090558_Geminiviridae_Begomovirus:0.082592):0.003347,
((((KC706561_Geminiviridae_Begomovirus:0.031619,AY090557_Geminiviridae
_Begomovirus:0.031619)89:0.019203,JF803252_Geminiviridae_Begomovirus:0
.050822):0.018126,EF016486_Geminiviridae_Begomovirus:0.068948):0.01090
7,AF408199_Geminiviridae_Begomovirus:0.079855):0.006084):0.005692,HM35
7461_Geminiviridae_Begomovirus:0.091631):0.006332,HM357456_Geminivirid
ae_Begomovirus:0.097963):0.006457,KC791690_Geminiviridae_Begomovirus:0
.10442):0.010445,KT948787_Geminiviridae_Begomovirus:0.114866):0.009694
,((((((AY083351_Geminiviridae_Begomovirus:0.041499,KC430935_Geminivir
idae_Begomovirus:0.041499):0.002767,EU710750_Geminiviridae_Begomovirus
:0.044266):0.007334,KC430936_Geminiviridae_Begomovirus:0.0516):0.00531
9,(AF130415_Geminiviridae_Begomovirus:0.048695,HM626515_Geminiviridae_
Begomovirus:0.048695):0.008225):0.002871,KX691411_Geminiviridae_Begomo
virus:0.05979):0.013444,(JQ743486_Geminiviridae_Begomovirus:0.020645,A
F149227_Geminiviridae_Begomovirus:0.020645)100:0.052589):0.010952,((((
JN680352_Geminiviridae_Begomovirus:0.026953,AJ508784_Geminiviridae_Beg
omovirus:0.026953)96:0.022782,KF358470_Geminiviridae_Begomovirus:0.049
735):0.01044,JX857693_Geminiviridae_Begomovirus:0.060175):0.004072,KC1
53490_Geminiviridae_Begomovirus:0.064247):0.019939)81:0.040373):0.0371
3,HM357458_Geminiviridae_Begomovirus:0.16169):0.019777):0.026311,(('EF
417916/EF417915 Geminiviridae Begomovirus':0.128912,'AF511528/AF511529
Geminiviridae Begomovirus':0.128912):0.024836,'AJ012082/AJ012081
Geminiviridae
Begomovirus':0.153747):0.05403):0.000758,GU385879_Geminiviridae_Begomo
virus:0.208536):0.019987,HQ396465_Geminiviridae_Begomovirus:0.228523):
0.007465,(((((((((((((((((('Y15034/Y15033 Geminiviridae
Begomovirus':0.022911,'JN848773/JN848774 Geminiviridae

Begomovirus':0.022911)71:0.006171,'HM236370/HM236371 Geminiviridae
Begomovirus':0.029082):0.003846,('FJ174698/FJ999999 Geminiviridae
Begomovirus':0.029973,'DQ022611/GU972604 Geminiviridae
Begomovirus':0.029973):0.002955):0.002591,(('GU997691/GU997692
Geminiviridae Begomovirus':0.025004,'DQ875868/DQ875869 Geminiviridae
Begomovirus':0.025004):0.008997,'KT899302/KT899303 Geminiviridae
Begomovirus':0.034001):0.001518):0.002958,((('KF723258/KF723261
Geminiviridae Begomovirus':0.030557,'JN848772/JQ283246 Geminiviridae
Begomovirus':0.030557):0.002173,('FJ213931/HQ896204 Geminiviridae
Begomovirus':0.027443,'AF101476/AF101478 Geminiviridae
Begomovirus':0.027443):0.005286):0.002694,((('AJ608286/AJ508783
Geminiviridae Begomovirus':0.024513,'HQ008338/HQ009518 Geminiviridae
Begomovirus':0.024513):0.004304,'DQ520944/DQ356428 Geminiviridae
Begomovirus':0.028817):0.003235,'L14460/L14461 Geminiviridae
Begomovirus':0.032052):0.003372):0.003053):0.004474,'JF803246/JF803264
Geminiviridae Begomovirus':0.042951):0.000566,((('JF694471/JF694473
Geminiviridae Begomovirus':0.028563,'X99550/X99551 Geminiviridae
Begomovirus':0.028563):0.004002,'AF039031/AF039032 Geminiviridae
Begomovirus':0.032566):0.006333,'HM585443/HM585444 Geminiviridae
Begomovirus':0.038898):0.004618):0.00316,(((('JF803254/JF803265
Geminiviridae Begomovirus':0.028768,'AF068636/AY965899 Geminiviridae
Begomovirus':0.028768):0.004246,'KT201151/KT201152 Geminiviridae
Begomovirus':0.033014):0.002065,'JN564749/JN564750 Geminiviridae
Begomovirus':0.035079):0.007165,('AY044135/AY044136 Geminiviridae
Begomovirus':0.039652,'JN848775/JN848776 Geminiviridae
Begomovirus':0.039652):0.002592):0.000637,'Y14874/Y14875 Geminiviridae
Begomovirus':0.042881):0.003795):0.002565,(((((((((('JN411687/JN4116
88 Geminiviridae Begomovirus':0.017825,'DQ875872/DQ875873
Geminiviridae Begomovirus':0.017825):0.003162,'DQ395343/EU158095
Geminiviridae Begomovirus':0.020988):0.004359,('AF049336/AF039841
Geminiviridae Begomovirus':0.021434,'X15983/X15984 Geminiviridae
Begomovirus':0.021434):0.003913):0.004282,'D00940/D00941 Geminiviridae
Begomovirus':0.029629):0.002215,'EU914817/EU914818 Geminiviridae
Begomovirus':0.031844):0.001751,'JN848770/JN848771 Geminiviridae
Begomovirus':0.033595):0.004064,'DQ520943/DQ406674 Geminiviridae
Begomovirus':0.03766):0.000836,(('AF012300/AF012301 Geminiviridae
Begomovirus':0.028853,'EU710752/EU710753 Geminiviridae
Begomovirus':0.028853):0.007754,'KX691402/KX691413 Geminiviridae
Begomovirus':0.036607):0.001889):0.00143,'HQ201952/HQ201953
Geminiviridae Begomovirus':0.039926):0.00406,('DQ207749/EF534708
Geminiviridae Begomovirus':0.039166,'AY508991/AY508992 Geminiviridae
Begomovirus':0.039166):0.004819):0.00111,'HM585441/HM585442
Geminiviridae Begomovirus':0.045096):0.002536,'EU158096/EU158097
Geminiviridae
Begomovirus':0.047631):0.00161):0.005779,'AY508993/AY508994
Geminiviridae Begomovirus':0.05502):0.000221,((((((('Y11097/Y11098
Geminiviridae Begomovirus':0.030523,'M88179/M88180 Geminiviridae
Begomovirus':0.030523):0.001887,'Y11099/Y11100 Geminiviridae
Begomovirus':0.032409):0.00797,'DQ336350/DQ336351 Geminiviridae
Begomovirus':0.040379):0.003745,'AF139168/AF170101 Geminiviridae
Begomovirus':0.044124):0.004149,(('JQ714137/JQ714138 Geminiviridae
Begomovirus':0.034622,'KU058856/KU058860 Geminiviridae

Begomovirus':0.034622):0.006753,'DQ178608/DQ178609 Geminiviridae
Begomovirus':0.041374):0.006898):0.002007,((('HM003779/HM003778
Geminiviridae Begomovirus':0.037487,'KX691407/KX691415 Geminiviridae
Begomovirus':0.037487):0.000803,'HQ009519/HQ009520 Geminiviridae
Begomovirus':0.03829):0.0031,'KC176780/KC176781 Geminiviridae
Begomovirus':0.04139):0.008889):0.001125,(('L01635/L01636
Geminiviridae Begomovirus':0.020096,'D00201/D00200 Geminiviridae
Begomovirus':0.020096)92:0.023113,'EF585290/EF585291 Geminiviridae
Begomovirus':0.043209):0.008195):0.003836):0.005194,'FJ972767/FJ972768
Geminiviridae Begomovirus':0.060435):0.000964,'KT201153/KT201154
Geminiviridae Begomovirus':0.061399):0.002487,(((('AF490004/AF491306
Geminiviridae Begomovirus':0.034428,'JN418998/JN418999 Geminiviridae
Begomovirus':0.034428):0.006409,'JX122965/JX122966 Geminiviridae
Begomovirus':0.040837):0.005366,'AF291705/AF291706 Geminiviridae
Begomovirus':0.046203):0.009152,'EU710756/EU710757 Geminiviridae
Begomovirus':0.055355):0.008531):0.002599,(('K02029/K02030
Geminiviridae Begomovirus':0.055059,'FJ601917/FJ600484 Geminiviridae
Begomovirus':0.055059):0.007378,('AJ557451/AJ557453 Geminiviridae
Begomovirus':0.053445,'HM585445/HM585446 Geminiviridae
Begomovirus':0.053445):0.008992):0.004048):0.001295,('JF694461/JF69446
2 Geminiviridae Begomovirus':0.047046,'JX827487/JX827488 Geminiviridae
Begomovirus':0.047046):0.020733):0.000584,(((('HM236368/HM236369
Geminiviridae Begomovirus':0.028148,'AY044133/AY044134 Geminiviridae
Begomovirus':0.028148)75:0.014815,'FJ944021/FJ944022 Geminiviridae
Begomovirus':0.042963):0.002609,(('DQ347950/EU339937 Geminiviridae
Begomovirus':0.028031,'EU339936/DQ356429 Geminiviridae
Begomovirus':0.028031):0.008236,'FJ944019/FJ944020 Geminiviridae
Begomovirus':0.036267):0.009306):0.015475,'X70418/X70419 Geminiviridae
Begomovirus':0.061048):0.007316):0.00328,(('M88686/M88687
Geminiviridae Begomovirus':0.025876,'JN419006/JN419008 Geminiviridae
Begomovirus':0.025876)97:0.01803,'KX691396/KX691412 Geminiviridae
Begomovirus':0.043906)71:0.027738)79:0.041032,(((((((('JF756671/JF7566
77 Geminiviridae Begomovirus':0.010283,'FJ619507/FJ619508
Geminiviridae Begomovirus':0.010283)100:0.023669,'AF224760/AF224761
Geminiviridae Begomovirus':0.033952)73:0.007658,('AY927277/EF547938
Geminiviridae Begomovirus':0.030942,'DQ318937/DQ318938 Geminiviridae
Begomovirus':0.030942):0.010669):0.01021,(('DQ875870/DQ875871
Geminiviridae Begomovirus':0.040503,'AF480940/AF480941 Geminiviridae
Begomovirus':0.040503):0.003421,('EU710754/EU710755 Geminiviridae
Begomovirus':0.030141,'FN434438/FN434439 Geminiviridae
Begomovirus':0.030141):0.013783):0.007896):0.010326,'JN381819/JN381820
Geminiviridae Begomovirus':0.062147):0.010113,(('M38183/M38182
Geminiviridae Begomovirus':0.040294,'AF325497/AF325498 Geminiviridae
Begomovirus':0.040294):0.020728,'AY064391/AF440790 Geminiviridae
Begomovirus':0.061022):0.011238):0.003125,(((('AF110189/AF110190
Geminiviridae Begomovirus':0.039546,'JF694480/JF694479 Geminiviridae
Begomovirus':0.039546):0.003223,('DQ406672/DQ406673 Geminiviridae
Begomovirus':0.029495,'U65529/U65530 Geminiviridae
Begomovirus':0.029495):0.013274):0.003167,'AF421552/AF421553
Geminiviridae Begomovirus':0.045936):0.011039,((('EU021216/FJ792608
Geminiviridae Begomovirus':0.040023,'KC426927/KC426928 Geminiviridae
Begomovirus':0.040023):0.004916,'U57457/AF499442 Geminiviridae

Begomovirus':0.044939):0.003125,'JQ821386/JQ821387 Geminiviridae
Begomovirus':0.048063):0.008912):0.01841):0.007182,'HM163576/HM163577
Geminiviridae
Begomovirus':0.082567)79:0.030108):0.051306,(((((((((((('AF422174/AF42
2175 Geminiviridae Begomovirus':0.031104,'AJ717572/AJ704971
Geminiviridae Begomovirus':0.031104):0.008899,'AF155806/AF155807
Geminiviridae Begomovirus':0.040003):0.006217,('AJ717542/AJ704949
Geminiviridae Begomovirus':0.034122,'AF126806/AF126807 Geminiviridae
Begomovirus':0.034122):0.012099):0.011199,(('AY795983/AY795989
Geminiviridae Begomovirus':0.039588,'AF112354/AF112355 Geminiviridae
Begomovirus':0.039588):0.016119,'JX658694/JX658684 Geminiviridae
Begomovirus':0.055706):0.001713):0.003573,('KT878829/KT878828
Geminiviridae Begomovirus':0.060451,'HE617299/HE617300 Geminiviridae
Begomovirus':0.060451):0.000541):0.009739,((('FN396962/FN396963
Geminiviridae Begomovirus':0.022685,'FJ011668/FJ011669 Geminiviridae
Begomovirus':0.022685)100:0.020675,'DQ641692/DQ641693 Geminiviridae
Begomovirus':0.04336)76:0.015002,'EU596959/EU596960 Geminiviridae
Begomovirus':0.058363):0.012368):0.012039,(('AJ314737/AJ314738
Geminiviridae Begomovirus':0.057982,'Z24758/Z24759 Geminiviridae
Begomovirus':0.057982):0.002565,'GQ924760/GQ924761 Geminiviridae
Begomovirus':0.060548)76:0.022222):0.002591,(((((('U15015/U15017
Geminiviridae Begomovirus':0.03519,'AF509743/AF509742 Geminiviridae
Begomovirus':0.03519):0.001876,'AM884015/AM992534 Geminiviridae
Begomovirus':0.037066):0.004387,'AB085793/AB085794 Geminiviridae
Begomovirus':0.041453):0.006074,'KJ862841/KJ862842 Geminiviridae
Begomovirus':0.047527):0.005046,'AF509739/AF509740 Geminiviridae
Begomovirus':0.052573):0.020644,'KM244719/KM244718 Geminiviridae
Begomovirus':0.073218):0.012143):0.004681,(('AF141922/AF141897
Geminiviridae Begomovirus':0.065768,'AY190290/AY190291 Geminiviridae
Begomovirus':0.065768):0.022975,('KT454832/KT454833 Geminiviridae
Begomovirus':0.0392,'KT454834/KT454835 Geminiviridae
Begomovirus':0.0392)99:0.049542):0.001298):0.008855,'J02057/J02058
Geminiviridae Begomovirus':0.098896):0.007618,'EU365617/EU384576
Geminiviridae
Begomovirus':0.106515):0.025147,(((((((('FN543425/FN543426
Geminiviridae Begomovirus':0.04512,'D14703/D14704 Geminiviridae
Begomovirus':0.04512):0.008224,'AJ627904/AJ627905 Geminiviridae
Begomovirus':0.053343):0.004239,'DQ641690/DQ641691 Geminiviridae
Begomovirus':0.057582):0.009416,('HM777508/HM777510 Geminiviridae
Begomovirus':0.054059,'AB267834/AB267835 Geminiviridae
Begomovirus':0.054059):0.012939):0.004362,'AM999981/AM999982
Geminiviridae Begomovirus':0.07136):0.020314,(('JF694484/JF694485
Geminiviridae Begomovirus':0.053034,'KP663485/KP663484 Geminiviridae
Begomovirus':0.053034)77:0.029786,'GQ472985/GQ472986 Geminiviridae
Begomovirus':0.08282):0.008854):0.008158,'AF126406/AF142440
Geminiviridae Begomovirus':0.099832):0.012107,((('FJ463902/FJ463901
Geminiviridae Begomovirus':0.026711,'DQ641688/DQ641689 Geminiviridae
Begomovirus':0.026711)90:0.017487,'AY727903/AY727904 Geminiviridae
Begomovirus':0.044198)100:0.053857,'KJ481204/KJ481205 Geminiviridae
Begomovirus':0.098055):0.013884):0.019723):0.03232):0.072006):0.04249,
KF660223_Geminiviridae_Begomovirus:0.278477):0.003093,(KC626021_Gemini
viridae_Begomovirus:0.144318,KC791691_Geminiviridae_Begomovirus:0.1443

18)80:0.137252):0.011465,(((((((((((FJ515898_Geminiviridae_Begomovir
us:0.022936,AJ586885_Geminiviridae_Begomovirus:0.022936):0.002926,EU83
9579_Geminiviridae_Begomovirus:0.025862):0.006661,(EF456744_Geminiviri
dae_Begomovirus:0.030194,AB433786_Geminiviridae_Begomovirus:0.030194):
0.002329):0.002412,HQ393473_Geminiviridae_Begomovirus:0.034935):0.0057
11,(((HQ333138_Geminiviridae_Begomovirus:0.02434,DQ644562_Geminivirida
e_Begomovirus:0.02434):0.007008,HQ333144_Geminiviridae_Begomovirus:0.0
31349):0.002164,(EU253456_Geminiviridae_Begomovirus:0.025643,AF104036_
Geminiviridae_Begomovirus:0.025643):0.00787):0.007133):0.003678,((EF45
6741_Geminiviridae_Begomovirus:0.034115,FJ969830_Geminiviridae_Begomov
irus:0.034115):0.008515,FR751068_Geminiviridae_Begomovirus:0.04263):0.
001694):0.00635,(HQ393456_Geminiviridae_Begomovirus:0.035909,HQ393447_
Geminiviridae_Begomovirus:0.035909):0.014764):0.00383,KF156759_Geminiv
iridae_Begomovirus:0.054504):0.004849,((((KF040467_Geminiviridae_Bego
movirus:0.025933,FJ529203_Geminiviridae_Begomovirus:0.025933):0.005004
,(KU992909_Geminiviridae_Begomovirus:0.019296,HQ333143_Geminiviridae_B
egomovirus:0.019296):0.01164):0.007603,(KJ476507_Geminiviridae_Begomov
irus:0.023342,KC907406_Geminiviridae_Begomovirus:0.023342)86:0.015197)
:0.003607,(AF326775_Geminiviridae_Begomovirus:0.036087,FJ969829_Gemini
viridae_Begomovirus:0.036087):0.006059):0.005775,((KC488316_Geminiviri
dae_Begomovirus:0.032373,KJ476510_Geminiviridae_Begomovirus:0.032373):
0.005246,EU839576_Geminiviridae_Begomovirus:0.037619):0.010302):0.0114
32):0.008528,(FJ969831_Geminiviridae_Begomovirus:0.043597,HQ393477_Gem
iniviridae_Begomovirus:0.043597):0.024285)74:0.019394,FN432356_Geminiv
iridae_Begomovirus:0.087276):0.012928,DQ512731_Geminiviridae_Begomovir
us:0.100204):0.022865,AJ132548_Geminiviridae_Begomovirus:0.123069)100:
0.169966):0.151075,((((((((EU921828_Geminiviridae_Curtovirus:0.02705
8,EU193175_Geminiviridae_Curtovirus:0.027058):0.006921,U56975_Geminivi
ridae_Curtovirus:0.033978):0.015605,EF501977_Geminiviridae_Curtovirus:
0.049584):0.002508,AY548948_Geminiviridae_Curtovirus:0.052092):0.01763
4,(U02311_Geminiviridae_Curtovirus:0.035289,FJ545686_Geminiviridae_Cur
tovirus:0.035289)88:0.034437):0.002677,JN817383_Geminiviridae_Curtovir
us:0.072403)97:0.039046,M24597_Geminiviridae_Curtovirus:0.111449)74:0.
047358,(((KC108896_Geminiviridae_Turncurtovirus:0.008633,GU456685_Gemi
niviridae_Turncurtovirus:0.008633):0.003411,(KC108905_Geminiviridae_Tu
rncurtovirus:0.00739,KC108902_Geminiviridae_Turncurtovirus:0.00739)79:
0.004654)100:0.022962,KT388076_Geminiviridae_Turncurtovirus:0.035006)1
00:0.1238):0.080783,(GU734126_Geminiviridae_Curtovirus:0.09935,U49907_
Geminiviridae_Curtovirus:0.09935)100:0.14024):0.062366,(((KX618694_Gem
iniviridae_Unassigned:0.086637,KM386645_Geminiviridae_Unassigned:0.086
637)99:0.109284,X84735_Geminiviridae_Topocuvirus:0.195921):0.060433,FM
164726_Geminiviridae_Begomovirus:0.256354):0.045602):0.142154):0.21431
7,(((KC896623_Geminiviridae_Unassigned:0.002638,JQ901105_Geminiviridae
_Grablovirus:0.002638)100:0.448311,(((((JX945572_Geminiviridae_Becurto
virus:0.007444,JX131633_Geminiviridae_Becurtovirus:0.007444):0.00175,J
X458087_Geminiviridae_Becurtovirus:0.009195)95:0.024325,EU273818_Gemin
iviridae_Becurtovirus:0.033519)79:0.02089,HQ443515_Geminiviridae_Becur
tovirus:0.05441)100:0.373413,(((((((((((((((((((EU628639_Geminiviri
dae_Mastrevirus:0.009181,EU628631_Geminiviridae_Mastrevirus:0.009181):
0.002192,(EU628641_Geminiviridae_Mastrevirus:0.008621,EU628629_Geminiv
iridae_Mastrevirus:0.008621):0.002752):0.000234,EU628597_Geminiviridae
_Mastrevirus:0.011607):0.000937,EU628638_Geminiviridae_Mastrevirus:0.0

12545):0.004622,((EU628643_Geminiviridae_Mastrevirus:0.010455,AF329889
_Geminiviridae_Mastrevirus:0.010455):0.002058,EU628626_Geminiviridae_M
astrevirus:0.012513):0.004654):0.001168,AF329878_Geminiviridae_Mastrev
irus:0.018335):0.001754,AF007881_Geminiviridae_Mastrevirus:0.02009)79:
0.011705,EU244915_Geminiviridae_Mastrevirus:0.031795):0.006049,(M82918
_Geminiviridae_Mastrevirus:0.030142,AF037752_Geminiviridae_Mastrevirus
:0.030142):0.007701):0.000675,(EU244916_Geminiviridae_Mastrevirus:0.03
1253,AF072672_Geminiviridae_Mastrevirus:0.031253):0.007265):0.007608,M
23022_Geminiviridae_Mastrevirus:0.046126):0.005349,(KX787914_Geminivir
idae_Mastrevirus:0.039424,GQ273988_Geminiviridae_Mastrevirus:0.039424)
:0.012051):0.001414,(EU445699_Geminiviridae_Mastrevirus:0.038306,EU244
914_Geminiviridae_Mastrevirus:0.038306):0.014583):0.00502,(((((GQ4153
95_Geminiviridae_Mastrevirus:0.011083,GQ415392_Geminiviridae_Mastrevir
us:0.011083):0.002557,EU224265_Geminiviridae_Mastrevirus:0.01364):0.00
071,(GQ415391_Geminiviridae_Mastrevirus:0.009494,EU224264_Geminivirida
e_Mastrevirus:0.009494):0.004856):0.002074,GQ415397_Geminiviridae_Mast
revirus:0.016424):0.010577,X60168_Geminiviridae_Mastrevirus:0.027001):
0.002851,(L39638_Geminiviridae_Mastrevirus:0.017593,GQ415399_Geminivir
idae_Mastrevirus:0.017593):0.012259)80:0.028057)72:0.055588,((((((KF8
06702_Geminiviridae_Mastrevirus:0.016738,KF806701_Geminiviridae_Mastre
virus:0.016738)100:0.043852,JX458741_Geminiviridae_Mastrevirus:0.06059
):0.011077,JQ948052_Geminiviridae_Mastrevirus:0.071667):0.006393,((JQ3
61910_Geminiviridae_Mastrevirus:0.05232,KJ437671_Geminiviridae_Mastrev
irus:0.05232):0.012808,JQ624879_Geminiviridae_Mastrevirus:0.065128):0.
012932):0.004377,(KJ210622_Geminiviridae_Mastrevirus:0.025193,KJ187746
_Geminiviridae_Mastrevirus:0.025193)99:0.057243):0.010353,((((JQ64745
5_Geminiviridae_Mastrevirus:0.002453,AJ311031_Geminiviridae_Mastreviru
s:0.002453)86:0.00929,FJ620684_Geminiviridae_Mastrevirus:0.011744):0.0
04647,AM040732_Geminiviridae_Mastrevirus:0.016391):0.006163,JN791096_G
eminiviridae_Mastrevirus:0.022554)91:0.020802,AM296025_Geminiviridae_M
astrevirus:0.043356)86:0.049434):0.011589,JQ948051_Geminiviridae_Mastr
evirus:0.104379):0.009118):0.034297,(D01030_Geminiviridae_Mastrevirus:
0.07365,JF508490_Geminiviridae_Mastrevirus:0.07365)75:0.074144):0.0090
03,((((((((AM850136_Geminiviridae_Mastrevirus:0.010555,AM849097_Gemin
iviridae_Mastrevirus:0.010555)100:0.00974,FR687960_Geminiviridae_Mastr
evirus:0.020295):0.012253,(AM933135_Geminiviridae_Mastrevirus:0.010537
,Y11023_Geminiviridae_Mastrevirus:0.010537)85:0.022012):0.015609,FR687
959_Geminiviridae_Mastrevirus:0.048157):0.007303,GU256532_Geminivirida
e_Mastrevirus:0.05546):0.005427,(((JN989426_Geminiviridae_Mastrevirus:
0.017456,GU256530_Geminiviridae_Mastrevirus:0.017456)81:0.01697,JN9894
16_Geminiviridae_Mastrevirus:0.034426):0.018413,(M81103_Geminiviridae_
Mastrevirus:0.042796,JN989422_Geminiviridae_Mastrevirus:0.042796):0.01
0042):0.008049):0.008295,KM377674_Geminiviridae_Mastrevirus:0.069182):
0.011669,JN989439_Geminiviridae_Mastrevirus:0.080851):0.014588,GU25653
1_Geminiviridae_Mastrevirus:0.095439):0.061358):0.00654,(((((JQ948069
_Geminiviridae_Mastrevirus:0.025272,JF905486_Geminiviridae_Mastrevirus
:0.025272):0.008224,JQ948061_Geminiviridae_Mastrevirus:0.033496):0.002
682,HQ113104_Geminiviridae_Mastrevirus:0.036178):0.018346,HM122238_Gem
iniviridae_Mastrevirus:0.054525):0.006129,(JQ948091_Geminiviridae_Mast
revirus:0.023772,JQ948088_Geminiviridae_Mastrevirus:0.023772)90:0.0368
82)79:0.049979,M20021_Geminiviridae_Mastrevirus:0.110633)75:0.052704):
0.066444,(FJ665631_Geminiviridae_Eragrovirus:0.021674,FJ665630_Geminiv

iridae_Eragrovirus:0.021674)100:0.208107)87:0.115222,((((KT214376_Gemi
niviridae_Capulavirus:0.00779,HF921459_Geminiviridae_Capulavirus:0.007
79)100:0.042513,KP732474_Geminiviridae_Capulavirus:0.050303)91:0.02977
8,JX094280_Geminiviridae_Capulavirus:0.08008)87:0.056317,KT214389_Gemi
niviridae_Capulavirus:0.136398)100:0.208605):0.054616,(KP303687_Gemini
viridae_Unassigned:0.238685,JQ920490_Geminiviridae_Unassigned:0.238685
)87:0.160935):0.007033,(JQ898331_Geminiviridae_Unassigned:0.178471,KF3
71630_Genomoviridae_Gemytondvirus:0.178471)93:0.228182):0.02117):0.023
126):0.089432,((((((((((((((((KJ547636_Genomoviridae_Gemycircularvir
us:0.052052,KT862243_Genomoviridae_Gemycircularvirus:0.052052):0.00475
8,(KJ547641_Genomoviridae_Gemycircularvirus:0.049439,GQ365709_Genomovi
ridae_Gemycircularvirus:0.049439):0.00737):0.008615,KT862245_Genomovir
idae_Gemycircularvirus:0.065425):0.005675,JX185429_Genomoviridae_Gemyc
ircularvirus:0.071099):0.005091,((KT732806_Genomoviridae_Gemycircularv
irus:0.02652,KT732807_Genomoviridae_Gemycircularvirus:0.02652)94:0.023
704,KT732814_Genomoviridae_Gemycircularvirus:0.050223):0.025967):0.010
878,(((KJ547640_Genomoviridae_Gemycircularvirus:0.055089,KM510192_Geno
moviridae_Gemycircularvirus:0.055089):0.00446,(KJ547639_Genomoviridae_
Gemycircularvirus:0.03751,KJ547638_Genomoviridae_Gemycircularvirus:0.0
3751)82:0.022039):0.012835,((JQ412056_Genomoviridae_Gemycircularvirus:
0.046285,KT862253_Genomoviridae_Gemycircularvirus:0.046285):0.00943,KT
732792_Genomoviridae_Gemycircularvirus:0.055715):0.016669):0.014684):0
.033105,((((((KT732794_Genomoviridae_Gemycircularvirus:0.045903,KF3716
38_Genomoviridae_Gemycircularvirus:0.045903):0.012868,KT862250_Genomov
iridae_Gemycircularvirus:0.058771):0.010635,(KF371640_Genomoviridae_Ge
mycircularvirus:0.042008,KF371639_Genomoviridae_Gemycircularvirus:0.04
2008):0.027398):0.007843,KT598248_Genomoviridae_Gemycircularvirus:0.07
7249):0.004012,((KT732795_Genomoviridae_Gemycircularvirus:0.063529,KT8
62248_Genomoviridae_Gemycircularvirus:0.063529):0.016664,KT253577_Geno
moviridae_Gemycircularvirus:0.080193):0.001068):0.024549,KJ641719_Geno
moviridae_Gemycircularvirus:0.10581):0.014364):0.00598,(((((KF371632_G
enomoviridae_Gemygorvirus:0.062206,KT732804_Genomoviridae_Gemycircular
virus:0.062206):0.021868,(KM598387_Genomoviridae_Gemycircularvirus:0.0
56118,KT309029_Genomoviridae_Gemycircularvirus:0.056118):0.027956):0.0
24909,(KT862249_Genomoviridae_Gemycircularvirus:0.085827,KF371636_Geno
moviridae_Gemycircularvirus:0.085827):0.023155):0.005001,KP263547_Geno
moviridae_Gemycircularvirus:0.113983):0.0056,((KT732803_Genomoviridae_
Gemycircularvirus:0.043157,KT732801_Genomoviridae_Gemycircularvirus:0.
043157)98:0.047438,KM598385_Genomoviridae_Gemycircularvirus:0.090595):
0.028987):0.00657):0.005684,((((((((KJ641726_Genomoviridae_Gemykibivir
us:0.046864,JX185430_Genomoviridae_Gemykibivirus:0.046864):0.004121,(K
J547642_Genomoviridae_Gemykibivirus:0.040151,KF371634_Genomoviridae_Ge
mykibivirus:0.040151):0.010833):0.017211,KF371633_Genomoviridae_Gemyki
bivirus:0.068196):0.004306,(KJ547643_Genomoviridae_Gemykibivirus:0.064
899,KU343137_Genomoviridae_Gemykibivirus:0.064899):0.007603):0.009077,
(((KP133075_Genomoviridae_Gemykibivirus:0.04016,KP263543_Genomoviridae
_Gemykibivirus:0.04016)88:0.011206,KJ641737_Genomoviridae_Gemykibiviru
s:0.051366):0.01909,LK931483_Genomoviridae_Gemykibivirus:0.070456):0.0
11123):0.003643,(((KJ547635_Genomoviridae_Gemygorvirus:0.023011,KT7327
90_Genomoviridae_Gemygorvirus:0.023011)76:0.009137,KT862254_Genomoviri
dae_Gemygorvirus:0.032149):0.00546,KT862238_Genomoviridae_Gemygorvirus
:0.037609)96:0.047612):0.0191,KP987887_Genomoviridae_Gemykibivirus:0.1

04321):0.012568,((KP263545_Genomoviridae_Gemykibivirus:0.077769,JX1854
28_Genomoviridae_Gemyduguivirus:0.077769):0.020186,KF371635_Genomoviri
dae_Gemycircularvirus:0.097955):0.018934):0.014949):0.037707,(KT732813
_Genomoviridae_Gemykibivirus:0.0917,LK931485_Genomoviridae_Gemykibivir
us:0.0917)91:0.077845):0.004604,((KT732797_Genomoviridae_Gemycircularv
irus:0.071547,KF413620_Genomoviridae_Gemycircularvirus:0.071547)97:0.0
87245,KR912221_Genomoviridae_Gemycircularvirus:0.158792):0.015357):0.0
1193,(KF371637_Genomoviridae_Gemycircularvirus:0.161424,KF371641_Genom
oviridae_Gemycircularvirus:0.161424):0.024656):0.022296,(KT732800_Geno
moviridae_Gemykolovirus:0.093466,KT732798_Genomoviridae_Gemykolovirus:
0.093466)83:0.114909):0.02192,KT363839_Genomoviridae_Gemykibivirus:0.2
30295):0.009561,HQ335086_Genomoviridae_Gemycircularvirus:0.239856):0.0
44271,KT862242_Genomoviridae_Gemycircularvirus:0.284127):0.020452,(((K
J938717_Genomoviridae_Gemykrogvirus:0.055446,LK931484_Genomoviridae_Ge
mykrogvirus:0.055446)73:0.017485,KJ547634_Genomoviridae_Gemykrogvirus:
0.072932)95:0.165798,KP974693_Genomoviridae_Gemyvongvirus:0.238729):0.
065849):0.069204,KF371631_Genomoviridae_Gemykroznavirus:0.373783):0.16
6597):0.118046)98:0.327879,(((((KY405007_Bacilladnaviridae_Protobacill
adnavirus:0.262036,KY405006_Bacilladnaviridae_Protobacilladnavirus:0.2
62036):0.046749,(KF133809_Bacilladnaviridae_Protobacilladnavirus:0.168
709,KY405008_Bacilladnaviridae_Kieseladnavirus:0.168709)78:0.140076):0
.110676,AB781089_Bacilladnaviridae_Diatodnavirus:0.419461):0.06271,(((
AB553581_Bacilladnaviridae_Protobacilladnavirus:0.145847,AB193315_Baci
lladnaviridae_Protobacilladnavirus:0.145847):0.060174,AB844272_Bacilla
dnaviridae_Protobacilladnavirus:0.206021):0.103944,AB597949_Bacilladna
viridae_Protobacilladnavirus:0.309966)84:0.172206)97:0.457667,(((((((
((((((((((KJ831064_Circoviridae_Cyclovirus:0.031834,JX185426_Circovir
idae_Cyclovirus:0.031834):0.017175,GQ404846_Circoviridae_Cyclovirus:0.
049008):0.007549,(GQ404854_Circoviridae_Cyclovirus:0.038671,KC512918_C
ircoviridae_Cyclovirus:0.038671):0.017887):0.001233,(GQ404847_Circovir
idae_Cyclovirus:0.041753,JX185422_Circoviridae_Cyclovirus:0.041753):0.
016037):0.005705,(GQ404844_Circoviridae_Cyclovirus:0.055302,HQ738637_C
ircoviridae_Cyclovirus:0.055302):0.008193):0.018687,(((KC512920_Circov
iridae_Cyclovirus:0.045652,JX185419_Circoviridae_Cyclovirus:0.045652):
0.008371,(GQ404855_Circoviridae_Cyclovirus:0.035811,JF938081_Circoviri
dae_Cyclovirus:0.035811):0.018212):0.004545,KJ641715_Circoviridae_Cycl
ovirus:0.058568):0.023615):0.013545,((((((HQ738636_Circoviridae_Cyclo
virus:0.034786,GQ404849_Circoviridae_Cyclovirus:0.034786):0.018967,KM3
82269_Circoviridae_Cyclovirus:0.053754):0.004249,KC512919_Circoviridae
_Cyclovirus:0.058003):0.004684,KC512916_Circoviridae_Cyclovirus:0.0626
87):0.013823,LC018134_Circoviridae_Cyclovirus:0.07651):0.008531,HQ7386
43_Circoviridae_Cyclovirus:0.085041):0.010422,((JF938082_Circoviridae_
Cyclovirus:0.064723,KJ641728_Circoviridae_Cyclovirus:0.064723):0.01173
1,KJ641717_Circoviridae_Cyclovirus:0.076454):0.019009):0.000265):0.018
763,KF726984_Circoviridae_Cyclovirus:0.114491):0.005554,(GQ404845_Circ
oviridae_Cyclovirus:0.070514,KJ641712_Circoviridae_Cyclovirus:0.070514
):0.049531):0.006507,(KJ641720_Circoviridae_Cyclovirus:0.079325,HM2288
74_Circoviridae_Cyclovirus:0.079325):0.047228):0.010677,(JX569794_Circ
oviridae_Cyclovirus:0.083295,JF938079_Circoviridae_Cyclovirus:0.083295
):0.053935):0.033895,KM382270_Circoviridae_Cyclovirus:0.171125):0.0610
92,JX185424_Circoviridae_Cyclovirus:0.232217):0.040018,((((KC771281_Ci
rcoviridae_Cyclovirus:0.058211,HQ738634_Circoviridae_Cyclovirus:0.0582

11):0.01768,KT732787_Circoviridae_Cyclovirus:0.075891):0.019597,GQ4048
57_Circoviridae_Cyclovirus:0.095488):0.015687,KF031466_Circoviridae_Cy
clovirus:0.111175)82:0.16106):0.133175,KM017740_Circoviridae_Cycloviru
s:0.405409):0.051422,(((((((KJ641723_Circoviridae_Circovirus:0.10253,
KJ641724_Circoviridae_Circovirus:0.10253):0.018246,(KJ641711_Circoviri
dae_Circovirus:0.069404,KJ641727_Circoviridae_Circovirus:0.069404):0.0
51373):0.116983,((JQ011377_Circoviridae_Circovirus:0.117789,GU799606_C
ircoviridae_Circovirus:0.117789):0.037026,GQ404856_Circoviridae_Circov
irus:0.154815):0.082945):0.053227,(((AF071879_Circoviridae_Circovirus
:0.033802,KC339249_Circoviridae_Circovirus:0.033802)84:0.032318,AY6518
50_Circoviridae_Circovirus:0.06612)71:0.051129,(KJ020099_Circoviridae_
Circovirus:0.058543,JX863737_Circoviridae_Circovirus:0.058543)87:0.058
706):0.043281,(KT783484_Circoviridae_Circovirus:0.069021,JQ814849_Circ
oviridae_Circovirus:0.069021)83:0.091509):0.130456):0.043095,KJ641740_
Circoviridae_Cyclovirus:0.334081):0.039561,((EU056309_Circoviridae_Cir
covirus:0.084562,AY228555_Circoviridae_Circovirus:0.084562):0.04606,AJ
304456_Circoviridae_Circovirus:0.130623)87:0.243018):0.018254,(((((((
DQ146997_Circoviridae_Circovirus:0.069708,GQ404851_Circoviridae_Circov
irus:0.069708):0.015312,AJ301633_Circoviridae_Circovirus:0.085021):0.0
4902,AF252610_Circoviridae_Circovirus:0.13404):0.016465,DQ172906_Circo
viridae_Circovirus:0.150506):0.057354,DQ845075_Circoviridae_Circovirus
:0.20786):0.023288,DQ845074_Circoviridae_Circovirus:0.231148):0.029878
,AF071878_Circoviridae_Circovirus:0.261026):0.04313,KP793918_Circoviri
dae_Circovirus:0.304156):0.08774):0.038204,KC241982_Circoviridae_Circo
virus:0.430099):0.026732):0.010627,KR902499_Circoviridae_Cyclovirus:0.
467459):0.001768,(KT732786_Circoviridae_Cyclovirus:0.071779,KT732785_C
ircoviridae_Cyclovirus:0.071779)90:0.397448)97:0.433594,((((('AB02751
1/AB009046/AB255373/... Nanoviridae
Nanovirus':0.009946,'GQ150778/GQ150780/GQ150779/... Nanoviridae
Nanovirus':0.009946)81:0.012365,'HE654123/HE654124/HE654127/...
Nanoviridae
Nanovirus':0.022311)76:0.044148,('KC979054/KC979061/KC979058/...
Nanoviridae Nanovirus':0.028265,'GU553134/JN133282/JN133280/...
Nanoviridae
Nanovirus':0.028265)89:0.038195):0.025597,(('KC978962/KC978959/KC97896
0/... Nanoviridae Nanovirus':0.020252,'KC978950/KC978953/KC978954/...
Nanoviridae
Nanovirus':0.020252)78:0.012429,'AJ132184/AJ132183/AJ132180/...
Nanoviridae
Nanovirus':0.032681)70:0.059375)100:0.373826,(('S56276/L41577/L41574/.
.. Nanoviridae Babuvirus':0.01345,'EF546813/EF546808/EF546810/...
Nanoviridae
Babuvirus':0.01345)100:0.058995,'KF435147/JX867550/JX867546/...
Nanoviridae
Babuvirus':0.072445)100:0.393436)98:0.346865,(((((((((((KT862221_Smac
oviridae_Porprismacovirus:0.063738,KT862218_Smacoviridae_Porprismacovi
rus:0.063738):0.029708,KT862225_Smacoviridae_Porprismacovirus:0.093446
):0.004523,KM573772_Smacoviridae_Porprismacovirus:0.097969):0.043149,(
(KT862219_Smacoviridae_Porprismacovirus:0.087424,KT862222_Smacoviridae
_Bovismacovirus:0.087424):0.024626,KM598409_Smacoviridae_Bovismacoviru
s:0.11205):0.029067):0.008583,((((KF880727_Smacoviridae_Porprismacovi
rus:0.040852,KJ577817_Smacoviridae_Porprismacovirus:0.040852):0.021655

,KJ577819_Smacoviridae_Porprismacovirus:0.062507):0.012823,KT600068_Sm
acoviridae_Porprismacovirus:0.07533):0.001065,KJ577810_Smacoviridae_Po
rprismacovirus:0.076395):0.011826,KP233194_Smacoviridae_Porprismacovir
us:0.088221):0.061479):0.039853,KT862228_Smacoviridae_Cosmacovirus:0.1
89554):0.069332,(((KT862223_Smacoviridae_Huchismacovirus:0.096671,KT86
2224_Smacoviridae_Drosmacovirus:0.096671):0.046117,KM573769_Smacovirid
ae_Drosmacovirus:0.142788):0.015905,KM573774_Smacoviridae_Drosmacoviru
s:0.158692):0.100194):0.04508,JN634851_Smacoviridae_Bovismacovirus:0.3
03966):0.040201,(GQ351275_Smacoviridae_Porprismacovirus:0.188227,GQ351
272_Smacoviridae_Porprismacovirus:0.188227)87:0.155941):0.035071,(((((
(((KJ577811_Smacoviridae_Porprismacovirus:0.03886,KM573775_Smacovirida
e_Porprismacovirus:0.03886):0.022403,KP233190_Smacoviridae_Porprismaco
virus:0.061263):0.012456,(JX274036_Smacoviridae_Porprismacovirus:0.054
648,KP233189_Smacoviridae_Porprismacovirus:0.054648):0.01907):0.016529
,(KT862220_Smacoviridae_Porprismacovirus:0.081263,KM573771_Smacovirida
e_Porprismacovirus:0.081263):0.008985):0.003475,KJ577816_Smacoviridae_
Porprismacovirus:0.093722):0.016244,KP860906_Smacoviridae_Porprismacov
irus:0.109966):0.003578,KJ577812_Smacoviridae_Porprismacovirus:0.11354
4):0.087945,(KT862229_Smacoviridae_Huchismacovirus:0.067835,KM598410_S
macoviridae_Dragsmacovirus:0.067835)81:0.133654):0.17775):0.073731,(((
(KP233174_Smacoviridae_Huchismacovirus:0.019219,KY086300_Smacoviridae_
Huchismacovirus:0.019219):0.007466,(KP233178_Smacoviridae_Huchismacovi
rus:0.016763,KY086301_Smacoviridae_Huchismacovirus:0.016763):0.009922)
:0.035849,KP233180_Smacoviridae_Huchismacovirus:0.062534)95:0.170661,(
((KP233191_Smacoviridae_Porprismacovirus:0.062133,KM573770_Smacovirida
e_Porprismacovirus:0.062133):0.013611,KC545226_Smacoviridae_Porprismac
ovirus:0.075744):0.010302,KC545227_Smacoviridae_Porprismacovirus:0.086
046)74:0.147149):0.219775):0.08957,GQ351273_Smacoviridae_Porprismacovi
rus:0.54254)100:0.270207)72:0.090073):0.037019):0.046467)86:0.008579):
0.005071,((((((((((((((((((((AB064605_Anelloviridae_Alphatorquevir
us:0.04379,AB064607_Anelloviridae_Alphatorquevirus:0.04379)82:0.022497
,AB060594_Anelloviridae_Alphatorquevirus:0.066286):0.010408,AB054647_A
nelloviridae_Alphatorquevirus:0.076694):0.012129,(AF435014_Anellovirid
ae_Alphatorquevirus:0.069069,AB041007_Anelloviridae_Alphatorquevirus:0
.069069):0.019754):0.006248,((AB041957_Anelloviridae_Alphatorquevirus:
0.076875,AF345524_Anelloviridae_Alphatorquevirus:0.076875):0.011871,AF
261761_Anelloviridae_Alphatorquevirus:0.088746):0.006325):0.010411,DQ1
87006_Anelloviridae_Alphatorquevirus:0.105482):0.00717,AB060597_Anello
viridae_Alphatorquevirus:0.112651):0.014761,((((AB017613_Anelloviridae
_Alphatorquevirus:0.085215,AB028668_Anelloviridae_Alphatorquevirus:0.0
85215):0.009021,AF348409_Anelloviridae_Alphatorquevirus:0.094236):0.01
7322,(AB037926_Anelloviridae_Alphatorquevirus:0.07526,AF345526_Anellov
iridae_Alphatorquevirus:0.07526):0.036297):0.00261,(AB025946_Anellovir
idae_Alphatorquevirus:0.090441,AX025830_Anelloviridae_Alphatorquevirus
:0.090441):0.023727):0.013245):0.007175,(AB049607_Anelloviridae_Alphat
orquevirus:0.114162,AB049608_Anelloviridae_Alphatorquevirus:0.114162):
0.020426):0.003396,AX025718_Anelloviridae_Alphatorquevirus:0.137983):0
.010295,AF345523_Anelloviridae_Alphatorquevirus:0.148279):0.017815,AX1
74942_Anelloviridae_Alphatorquevirus:0.166094):0.006088,((FR751463_Ane
lloviridae_Unassigned:0.083058,AB038621_Anelloviridae_Alphatorquevirus
:0.083058)76:0.03712,(AB064598_Anelloviridae_Alphatorquevirus:0.081921
,AB064595_Anelloviridae_Alphatorquevirus:0.081921)81:0.038258)73:0.052

003):0.00582,AY666122_Anelloviridae_Alphatorquevirus:0.178002):0.07093
3,(((AB041958_Anelloviridae_Alphatorquevirus:0.131073,AB041959_Anellov
iridae_Alphatorquevirus:0.131073)75:0.071325,KP296856_Anelloviridae_Al
phatorquevirus:0.202398):0.031286,KP296857_Anelloviridae_Alphatorquevi
rus:0.233684):0.015251):0.035818,((KP296855_Anelloviridae_Alphatorquev
irus:0.19693,KP296854_Anelloviridae_Alphatorquevirus:0.19693):0.022833
,KP296853_Anelloviridae_Alphatorquevirus:0.219762)81:0.06499):0.108964
,((((((((((((AB303558_Anelloviridae_Gammatorquevirus:0.043296,AB30356
1_Anelloviridae_Gammatorquevirus:0.043296):0.006863,AB303553_Anellovir
idae_Gammatorquevirus:0.050159):0.008935,AB303559_Anelloviridae_Gammat
orquevirus:0.059094):0.005633,((AB303554_Anelloviridae_Gammatorqueviru
s:0.048459,AB303552_Anelloviridae_Gammatorquevirus:0.048459):0.012209,
AB290917_Anelloviridae_Gammatorquevirus:0.060668):0.004059):0.001004,(
(AB303564_Anelloviridae_Gammatorquevirus:0.056428,AB303560_Anellovirid
ae_Gammatorquevirus:0.056428):0.001805,AB303562_Anelloviridae_Gammator
quevirus:0.058233):0.007498):0.014164,AB303566_Anelloviridae_Gammatorq
uevirus:0.079895):0.006782,AB290919_Anelloviridae_Gammatorquevirus:0.0
86677):0.018235,AB449062_Anelloviridae_Gammatorquevirus:0.104912):0.02
0668,(AY622908_Anelloviridae_Unassigned:0.100149,EF538876_Anelloviridа
e_Gammatorquevirus:0.100149):0.025431):0.021907,AY622909_Anelloviridae
_Unassigned:0.147487)89:0.073487,EF538875_Anelloviridae_Gammatorquevir
us:0.220973):0.0058,KT027939_Anelloviridae_Unassigned:0.226774)77:0.12
8859,((((((((((KM259873_Anelloviridae_Betatorquevirus:0.075641,AF2910
73_Anelloviridae_Betatorquevirus:0.075641):0.013232,AB026929_Anellovir
idae_Betatorquevirus:0.088873):0.001563,AB038627_Anelloviridae_Betator
quevirus:0.090436):0.006945,(AB038630_Anelloviridae_Betatorquevirus:0.
083871,EF538882_Anelloviridae_Betatorquevirus:0.083871):0.01351):0.011
177,AB038625_Anelloviridae_Betatorquevirus:0.108558):0.004336,AB038629
_Anelloviridae_Betatorquevirus:0.112894):0.001686,(JX134044_Anelloviri
dae_Unassigned:0.107927,KM259874_Anelloviridae_Betatorquevirus:0.10792
7):0.006653):0.018076,((KU041847_Anelloviridae_Betatorquevirus:0.09387
2,AB026931_Anelloviridae_Betatorquevirus:0.093872):0.013959,AB041963_A
nelloviridae_Betatorquevirus:0.107831):0.024825):0.004149,AB041962_Ane
lloviridae_Betatorquevirus:0.136805):0.025338,EF538880_Anelloviridae_B
etatorquevirus:0.162143):0.018884,EF538881_Anelloviridae_Betatorquevir
us:0.181027)93:0.174606):0.038084):0.02576,AB041960_Anelloviridae_Epsi
lontorquevirus:0.419477):0.066156,AB041961_Anelloviridae_Zetatorquevir
us:0.485633):0.097622,((((KM434181_Anelloviridae_Unassigned:0.496104,K
M262783_Anelloviridae_Unassigned:0.496104):0.01337,KM668486_Anelloviri
dae_Unassigned:0.509474):0.011581,(JQ406846_Anelloviridae_Kappatorquev
irus:0.116921,AY823991_Anelloviridae_Kappatorquevirus:0.116921)100:0.4
04133):0.043359,((AY823990_Anelloviridae_Iotatorquevirus:0.0837,AB0760
01_Anelloviridae_Iotatorquevirus:0.0837)97:0.451516,AB057358_Anellovir
idae_Deltatorquevirus:0.535216):0.029198):0.018842):0.060999,AB076002_
Anelloviridae_Thetatorquevirus:0.644254)100:0.204478,(((((HQ287751_Ane
lloviridae_Unassigned:0.146794,KF373760_Anelloviridae_Unassigned:0.146
794)78:0.130629,KF373758_Anelloviridae_Unassigned:0.277423):0.074705,F
J459582_Anelloviridae_Lambdatorquevirus:0.352128)73:0.089841,AB076003_
Anelloviridae_Etatorquevirus:0.441969):0.030469,EF538877_Anelloviridae
_Etatorquevirus:0.472438)99:0.376294)99:0.11039,((((((KM111536_Anellov
iridae_Gyrovirus:0.057058,HM590588_Anelloviridae_Gyrovirus:0.057058):0
.006489,JQ308210_Anelloviridae_Gyrovirus:0.063548):0.019099,KF294862_A

nelloviridae_Gyrovirus:0.082647):0.008129,M55918_Anelloviridae_Gyrovir
us:0.090776)96:0.27652,KR137527_Anelloviridae_Gyrovirus:0.367296)89:0.
229055,(KF294861_Anelloviridae_Gyrovirus:0.093137,JX310702_Anellovirid
ae_Gyrovirus:0.093137)99:0.503213)99:0.362772)80:0.03211,(((EF506570_I
noviridae_Vespertiliovirus:0.065666,U28974_Inoviridae_Vespertiliovirus
:0.065666):0.149778,X51344_Inoviridae_Vespertiliovirus:0.215444)97:0.4
60358,AF133242_Inoviridae_Unassigned:0.675802):0.31543):0.008724):4.1e
-
05,(((((((HG315669_Inoviridae_Unassigned:0.455637,EF489910_Inoviridae_
Unassigned:0.455637):0.119777,M57538_Inoviridae_Unassigned:0.575414):0
.097318,(AB259124_Inoviridae_Unassigned:0.422784,HM064452_Inoviridae_U
nassigned:0.422784)84:0.249948):0.037822,M11912_Inoviridae_Unassigned:
0.710554):0.114219,(((AB434711_Inoviridae_Habenivirus:0.045956,AB25912
3_Inoviridae_Habenivirus:0.045956)95:0.220546,AB937974_Inoviridae_Habe
nivirus:0.266502)98:0.356121,(AM040673_Inoviridae_Unassigned:0.40083,H
G007973_Inoviridae_Unassigned:0.40083)80:0.221793):0.20215):0.061461,(
((((AB043678_Inoviridae_Unassigned:0.32711,AB012573_Inoviridae_Unassig
ned:0.32711):0.116298,(AY242528_Inoviridae_Fibrovirus:0.090728,D89074_
Inoviridae_Fibrovirus:0.090728)99:0.35268):0.175,HQ224500_Inoviridae_U
nassigned:0.618408):0.012798,(JN848801_Inoviridae_Unassigned:0.418127,
AY714348_Inoviridae_Unassigned:0.418127):0.213079):0.205077,X52107_Ino
viridae_Unassigned:0.836283)74:0.049951):0.039145,(((U02303_Inoviridae
_Unassigned:0.371092,V00604_Inoviridae_Inovirus:0.371092):0.029356,(X0
2139_Inoviridae_Lineavirus:0.255846,X14336_Inoviridae_Lineavirus:0.255
846):0.144603)94:0.193711,(KC357596_Inoviridae_Saetivirus:0.089475,AB0
02632_Inoviridae_Saetivirus:0.089475)100:0.504685)85:0.33122)98:0.0746
17):3e-
06,((X58839_Inoviridae_Plectrovirus:1.0,(((((((((DQ079871_Microviridae
_Alpha3microvirus:0.002652,DQ079870_Microviridae_Alpha3microvirus:0.00
2652):0.000648,DQ079872_Microviridae_Alpha3microvirus:0.0033):0.000598
,DQ079876_Microviridae_Alpha3microvirus:0.003899):0.000739,DQ079879_Mi
croviridae_Alpha3microvirus:0.004637):0.000783,DQ079875_Microviridae_A
lpha3microvirus:0.00542):0.012518,DQ079874_Microviridae_Alpha3microvir
us:0.017938):0.005148,((GQ149088_Microviridae_Alpha3microvirus:0.00241
,X60323_Microviridae_Alpha3microvirus:0.00241)94:0.003732,X60322_Micro
viridae_Alpha3microvirus:0.006141)98:0.016944)91:0.068545,(((DQ079869_
Microviridae_G4microvirus:0.023034,DQ079877_Microviridae_G4microvirus:
0.023034):0.015829,V00657_Microviridae_G4microvirus:0.038863)72:0.0405
74,J02482_Microviridae_Phix174microvirus:0.079437):0.012193)100:0.8571
19,(((((U41758_Microviridae_Chlamydiamicrovirus:0.005132,AE002163_Micr
oviridae_Chlamydiamicrovirus:0.005132):0.004425,AJ270057_Microviridae_
Chlamydiamicrovirus:0.009557)100:0.290377,AF306496_Microviridae_Bdello
microvirus:0.299934):0.135277,M17988_Microviridae_Spiromicrovirus:0.43
5211):0.033303,D00624_Microviridae_Chlamydiamicrovirus:0.468514)100:0.
480235)97:0.051251):0.0,((((JN882266_Pleolipoviridae_Alphapleolipoviru
s:0.153777,JN882264_Pleolipoviridae_Alphapleolipovirus:0.153777)99:0.4
17619,FJ685651_Pleolipoviridae_Alphapleolipovirus:0.571397):0.02073,(K
F056323_Pleolipoviridae_Alphapleolipovirus:0.118791,GU321093_Pleolipov
iridae_Alphapleolipovirus:0.118791)100:0.473336)76:0.218924,((JN882265
_Pleolipoviridae_Betapleolipovirus:0.588563,JN882267_Pleolipoviridae_B
etapleolipovirus:0.588563):0.199149,AF191797_Pleolipoviridae_Gammapleo

lipovirus:0.787713):0.023338)100:0.188949):0.0)100:0.0,HE681887_Spirav
iridae_Alphaspiravirus:1.0);

Figure S4 UPGMA dendrogram of Group II ssDNA viruses

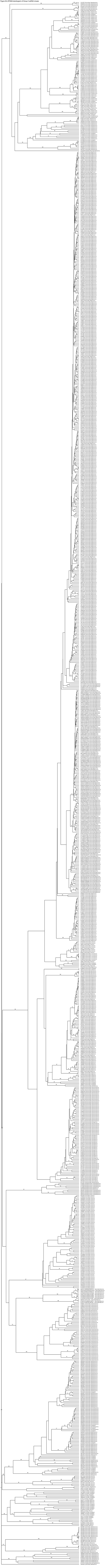

Figure S5 UPGMA dendrogram of Group III dsRNA viruses (NEWICK format)

((((((((((((((KT456287_Endornaviridae_Alphaendornavirus:0.001717,AB719397_Endornaviridae_Alphaendornavirus:0.001717)100:0.02049,KJ634409_Endornaviridae_Alphaendornavirus:0.022207):0.005907,AB844265_Endornaviridae_Alphaendornavirus:0.028114):0.01579,(D32136_Endornaviridae_Alphaendornavirus:0.019056,AB014344_Endornaviridae_Alphaendornavirus:0.019056)98:0.024848):0.002741,JN880414_Endornaviridae_Alphaendornavirus:0.046646):0.008168,(KF562072_Endornaviridae_Alphaendornavirus:0.008445,KT727022_Endornaviridae_Alphaendornavirus:0.008445)89:0.046368)73:0.496174,(((((KX977569_Endornaviridae_Alphaendornavirus:0.003417,JN019858_Endornaviridae_Alphaendornavirus:0.003417)91:0.009617,KR080326_Endornaviridae_Alphaendornavirus:0.013034):0.023529,(KT456288_Endornaviridae_Alphaendornavirus:0.001443,AB719398_Endornaviridae_Alphaendornavirus:0.001443)100:0.035119):0.035172,LC144945_Endornaviridae_Unassigned:0.071734)98:0.445694,KT721705_Endornaviridae_Alphaendornavirus:0.517428):0.03356):0.024383,AJ877914_Endornaviridae_Alphaendornavirus:0.57537):0.011439,KF311065_Endornaviridae_Alphaendornavirus:0.58681):0.021627,AJ000929_Endornaviridae_Alphaendornavirus:0.608436):0.010068,AB218287_Endornaviridae_Alphaendornavirus:0.618504):0.018986,(JX678977_Endornaviridae_Alphaendornavirus:0.01912,KT388110_Endornaviridae_Alphaendornavirus:0.01912)100:0.618371):0.114634,(((((KJ123645_Endornaviridae_Betaendornavirus:0.004417,KC852908_Endornaviridae_Betaendornavirus:0.004417)100:0.065291,DQ399289_Endornaviridae_Betaendornavirus:0.069708)98:0.519082,HQ380014_Endornaviridae_Betaendornavirus:0.58879):0.014042,KP239989_Endornaviridae_Betaendornavirus:0.602833):0.00398,LC076696_Endornaviridae_Endornavirus:0.606813)77:0.145311)100:0.247441,(((AB512282/AB512283_Megabirnaviridae_Megabirnavirus:0.870335,((((HM004067/HM004068/HM004069/..._Chrysoviridae_Chrysovirus:0.141708,GQ290645/GQ290649/HM013826/..._Chrysoviridae_Chrysovirus:0.141708)99:0.2284,(AF296439/AF296440/AF296441/..._Chrysoviridae_Chrysovirus:0.026816,FN178512/FN178513/FN178514/..._Chrysoviridae_Chrysovirus:0.026816)100:0.343291)82:0.108019,((AJ781397/AJ781398/AJ781399/..._Chrysoviridae_Chrysovirus:0.002228,AJ781166/AJ781165/AJ781164/..._Chrysoviridae_Chrysovirus:0.002228)100:0.348506,AF297176/AF297177/AF297178/..._Chrysoviridae_Chrysovirus:0.350734)82:0.127393)99:0.343965,(AB620061/AB620062/AB620063/..._Quadriviridae_Quadrivirus:0.818848,(JF716350/JF716351_Botybirnaviridae_Botybirnavirus:0.815403,(U01059_Totiviridae_Totivirus:0.790934,((KC610514_Totiviridae_Totivirus:0.499549,((J04692_Totiviridae_Totivirus:0.179257,HQ158596_Totiviridae_Totivirus:0.179257)99:0.267565,(U01060_Totiviridae_Totivirus:0.391062,(JN997472_Totiviridae_Totivirus:0.025774,JN997473_Totiviridae_Totivirus:0.025774)99:0.365288):0.055759):0.052727)88:0.205338,((U08999_Totiviridae_Trichomonasvirus:0.560311,(HQ607520_Totiviridae_Trichomonasvirus:0.22671,(AF127178_Totiviridae_Trichomonasvirus:0.118937,AF325840_Totiviridae_Trichomonasvirus:0.118937)79:0.107772)97:0.333602)84:0.083663,((M92355_Totiviridae_Leishmaniavirus:0.066623,U32108_Totiviridae_Leishmaniavirus:0.066623)96:0.291795,(AY561500_Totiviridae_Victorivirus:0.178027,((AF527633_Totiviridae_Victorivirus:0.055361,(AF039080_Totiviridae_Victorivirus:0.037962,(AB300379_Totiviridae_Victorivirus:0.034064,(AM261427_Totiviridae_Victorivirus:0.02421,AF337175_Totiviridae_Victorivirus:0.02421):0.009854):0.003898):0.017399):0.019769,((AB085814_Totiviridae_Victorivirus:0.052016,AB176964_Totiviridae_Victorivi

rus:0.052016):0.019734,((U41345_Totiviridae_Victorivirus:0.037827,AB74
2454_Totiviridae_Victorivirus:0.037827):0.009949,((HE588147_Totivirida
e_Victorivirus:0.024654,HE572591_Totiviridae_Victorivirus:0.024654):0.
022305,(AF038665_Totiviridae_Victorivirus:0.035902,FR750562_Totivirida
e_Victorivirus:0.035902):0.011058):0.000816):0.023974):0.00338)79:0.10
2897)88:0.180392)92:0.285556):0.060912):0.086047):0.024469):0.003445):
0.003244):0.048243)93:0.066487,L13218_Totiviridae_Giardiavirus:0.93682
1):0.061443,((((((((((((((KF484726/KF484727_Partitiviridae_Unassign
ed:0.007701,FJ550604/FJ550605_Partitiviridae_Alphapartitivirus:0.00770
1)74:0.003328,KF484724/KF484725_Partitiviridae_Unassigned:0.011029)90:
0.007515,AY705784/AY705785_Partitiviridae_Alphapartitivirus:0.018544)7
6:0.009656,EU489061/EU489062_Partitiviridae_Alphapartitivirus:0.0282):
0.005399,(((EF173394/EF173393_Partitiviridae_Alphapartitivirus:0.00011
6,EF173392/EF173391_Partitiviridae_Alphapartitivirus:0.000116)96:0.000
205,EF173396/EF173395_Partitiviridae_Alphapartitivirus:0.000322)100:0.
003165,(EU605883/EU605884_Partitiviridae_Alphapartitivirus:0.002566,AY
751737/AY751738_Partitiviridae_Alphapartitivirus:0.002566)75:0.00092)1
00:0.030112)96:0.055089,KF372436/KF372437_Partitiviridae_Unassigned:0.
088687)89:0.23379,AJ781168/AJ781167_Partitiviridae_Alphapartitivirus:0
.322476):0.041168,HQ541323/HQ541324_Partitiviridae_Alphapartitivirus:0
.363645):0.006451,(AB465308/AB465309_Partitiviridae_Alphapartitivirus:
0.363982,AM999771/AM999772_Partitiviridae_Alphapartitivirus:0.363982):
0.006114):0.00575,AY949985/DQ181926_Partitiviridae_Unassigned:0.375846
):0.005047,((((KF963179/KF963180_Partitiviridae_Alphapartitivirus:0.00
195,KF963177/KF963178_Partitiviridae_Alphapartitivirus:0.00195)100:0.1
03485,KU565806/KU565807_Partitiviridae_Alphapartitivirus:0.105436)75:0
.185228,KF963186/KF963187_Partitiviridae_Alphapartitivirus:0.290664):0
.009419,(FJ816271/FJ816272_Partitiviridae_Alphapartitivirus:0.013092,K
F963175/KF963176_Partitiviridae_Alphapartitivirus:0.013092)100:0.28699
1):0.080809):0.017325,(LC151461/LC151462_Partitiviridae_Unassigned:0.0
69849,AB569997/AB569998_Partitiviridae_Alphapartitivirus:0.069849)86:0
.328368)99:0.320779,((((((((JX971984/JX971985_Partitiviridae_Betapart
itivirus:0.010128,KX709964/KX709965_Partitiviridae_Betapartitivirus:0.
010128):0.002939,JX971980/JX971981_Partitiviridae_Betapartitivirus:0.0
13067):0.001416,(JX971976/JX971977_Partitiviridae_Betapartitivirus:0.0
07717,JX971978/JX971979_Partitiviridae_Betapartitivirus:0.007717)76:0.
006765)72:0.012559,(EU195326/EU195327_Partitiviridae_Betapartitivirus:
0.022329,JX971982/JX971983_Partitiviridae_Betapartitivirus:0.022329):0
.004713):0.002264,JN196536/JN196537_Partitiviridae_Betapartitivirus:0.
029306)87:0.057422,(AB113347/AB113348_Partitiviridae_Betapartitivirus:
0.046972,AF133290/AF133291_Partitiviridae_Betapartitivirus:0.046972)75
:0.039756):0.044579,LC010953/LC010952_Partitiviridae_Betapartitivirus:
0.131307)83:0.243228,((AY533038/AY533036_Partitiviridae_Betapartitivir
us:0.023951,JX625227/JX625228_Partitiviridae_Betapartitivirus:0.023951
):0.004778,(LC150606/LC150607_Partitiviridae_Betapartitivirus:0.007305
,AF047013/AF015924_Partitiviridae_Betapartitivirus:0.007305)93:0.02142
3)93:0.345807):0.052425,((((AY603051/AY603052_Partitiviridae_Betaparti
tivirus:0.013063,AY260757/AY260756_Partitiviridae_Betapartitivirus:0.0
13063)88:0.028082,AY247204/AY247205_Partitiviridae_Betapartitivirus:0.
041144)96:0.042449,L39125/L39126/L39127_Partitiviridae_Betapartitiviru
s:0.083593)90:0.303455,(JN606091/JN606090_Partitiviridae_Betapartitivi
rus:0.025639,HM565953/HM565954_Partitiviridae_Betapartitivirus:0.02563

9)96:0.361408):0.039912)95:0.292036)88:0.099885,((((((((AM040148/AM04
0149_Partitiviridae_Gammapartitivirus:0.001568,AY156521/AY156522_Parti
tiviridae_Gammapartitivirus:0.001568)98:0.009683,AY089993/AY089994_Par
titiviridae_Gammapartitivirus:0.01125):0.007511,(AY033436/AY033437_Par
titiviridae_Gammapartitivirus:0.014918,AF316992/AF316993_Partitivirida
e_Gammapartitivirus:0.014918):0.003843):0.018242,AM087202/AM087203_Par
titiviridae_Gammapartitivirus:0.037003):0.0451,D55668/D55669_Partitivi
ridae_Gammapartitivirus:0.082103):0.055422,(AY615211/AY615212/AY615213
_Partitiviridae_Gammapartitivirus:0.056347,EU118277/EU118278/EU118279_
Partitiviridae_Gammapartitivirus:0.056347):0.081178)78:0.194533,AY7383
36/AY738337_Partitiviridae_Gammapartitivirus:0.332057)74:0.376136,((((
(((LC195294/LC195295_Partitiviridae_Deltapartitivirus:0.002356,JN11727
8/JN117279_Partitiviridae_Deltapartitivirus:0.002356)98:0.018361,HE805
113/HE805114_Partitiviridae_Unassigned:0.020718):0.022023,FJ461349/FJ4
61350_Partitiviridae_Unassigned:0.042741):0.014199,(KT285020/KT285021_
Partitiviridae_Unassigned:0.023448,JN117276/JN117277_Partitiviridae_De
ltapartitivirus:0.023448):0.033493):0.114713,HM560702/HM560703/HM56070
4_Partitiviridae_Deltapartitivirus:0.171653)87:0.137537,FR687854/FR687
855_Partitiviridae_Deltapartitivirus:0.30919):0.189006,(DQ218036/DQ218
037/DQ218038_Partitiviridae_Unassigned:0.249951,DQ093961/DQ355440/DQ35
5439_Partitiviridae_Unassigned:0.249951)85:0.248245):0.209997):0.05913
7,(KY884720/KY884721_Partitiviridae_Cryspovirus:0.004162,U95995/U95996
_Partitiviridae_Cryspovirus:0.004162)100:0.763168)77:0.051551)95:0.127
002,(KF861768/KF861773_Picobirnaviridae_Picobirnavirus:0.459094,AB1868
97/AB186898_Picobirnaviridae_Picobirnavirus:0.459094)100:0.486788):0.0
11528,((HQ128706_Amalgaviridae_Amalgavirus:0.290481,HM029246_Amalgavir
idae_Amalgavirus:0.290481):0.011962,EF442780_Amalgaviridae_Amalgavirus
:0.302444)99:0.654967):0.017165,((((AJ304859/AJ303139/X79442/..._Reovi
ridae-
Rotavirus_Rotavirus:0.27744,DQ838640/DQ838635/DQ838645/..._Reoviridae-
Rotavirus_Rotavirus:0.27744):0.022073,GU733443/GU733444/GU733445/..._R
eoviridae-
Rotavirus_Rotavirus:0.299512):0.009065,JN596591/JQ919995/JQ919997/..._
Reoviridae-
Rotavirus_Rotavirus:0.308577)100:0.515103,(((JN596592/JQ920004/JQ92000
6/..._Reoviridae-
Rotavirus_Rotavirus:0.099672,EU490415/AY238390/EU490418/..._Reoviridae
-
Rotavirus_Rotavirus:0.099672)99:0.192404,KM369892/KM369893/KM369895/..
._Reoviridae-
Rotavirus_Rotavirus:0.292076):0.01277,DQ113897/DQ113898/DQ113899/..._R
eoviridae-
Rotavirus_Rotavirus:0.304847)100:0.518833)100:0.150896):0.023689):0.00
1301):0.000408,(((((((((KF234259/KF234260/KF234261/..._Reoviridae-
Orbivirus_Orbivirus:0.170298,JQ070376/JQ070377/JQ070378/..._Reoviridae
-
Orbivirus_Orbivirus:0.170298):0.029891,KF624614/KF624615/KF624616/..._
Reoviridae-
Orbivirus_Orbivirus:0.200189):0.004974,((AM744977/AM744978/AM744979/..
._Reoviridae-
Orbivirus_Orbivirus:0.169359,X12819/M11787/M22096/..._Reoviridae-
Orbivirus_Orbivirus:0.169359):0.004705,KR822286/KR822287/KR822288/..._

Reoviridae-
Orbivirus_Orbivirus:0.174064):0.031099):0.005649,KJ495755/KJ495756/KJ4
95757/..._Reoviridae-
Orbivirus_Orbivirus:0.210812):0.061752,(((JQ610675/JQ610676/JQ610677/.
.._Reoviridae-
Orbivirus_Orbivirus:0.171363,JQ610665/JQ610666/JQ610667/..._Reoviridae
-
Orbivirus_Orbivirus:0.171363):0.034774,U94887/AF021235/M94681/..._Reov
iridae-
Orbivirus_Orbivirus:0.206137):0.012901,AB018086/AB014725/AB014728/..._
Reoviridae-
Orbivirus_Orbivirus:0.219039):0.053525):0.003027,FJ183384/FJ183385/FJ1
83386/..._Reoviridae-
Orbivirus_Orbivirus:0.275591)81:0.090307,(((((KP268804/KP268805/KP2688
06/..._Reoviridae-
Orbivirus_Orbivirus:0.15672,KP268794/KP268795/KP268796/..._Reoviridae-
Orbivirus_Orbivirus:0.15672):0.010743,HM543465/HM543466/HM543467/..._R
eoviridae-
Orbivirus_Orbivirus:0.167464)74:0.069246,KP268784/KP268785/KP268786/..
._Reoviridae-
Orbivirus_Orbivirus:0.23671):0.052877,(HQ842619/HQ842620/HQ842621/..._
Reoviridae-
Orbivirus_Orbivirus:0.205092,KC853042/KC853043/KC853044/..._Reoviridae
-
Orbivirus_Orbivirus:0.205092)72:0.084495):0.005847,((AY701509/AY701510
/AY701511/..._Reoviridae-
Orbivirus_Orbivirus:0.023818,DQ248057/DQ248058/DQ248059/..._Reoviridae
-
Orbivirus_Orbivirus:0.023818):0.001643,KF296322/KF296324/KF296323/..._
Reoviridae-
Orbivirus_Orbivirus:0.025461)100:0.269973)73:0.070464)96:0.142988,AF13
3431/AF133432/AF145400/..._Reoviridae-
Orbivirus_Orbivirus:0.508886)100:0.479464,(((AY701339/AY701340/AY70134
1/..._Reoviridae-
Seadornavirus_Seadornavirus:0.291361,AF133429/AF134509/AF134510/..._Re
oviridae-
Seadornavirus_Seadornavirus:0.291361)93:0.133092,AF133430/AF134514/AF1
34515/..._Reoviridae-
Seadornavirus_Seadornavirus:0.424452)100:0.513869,KP638402/KP638403/KP
638404/..._Reoviridae-
Cardoreovirus_Cardoreovirus:0.938321)91:0.050028):0.00408,((U73201/U73
202/U72757/..._Reoviridae-
Phytoreovirus_Phytoreovirus:0.215178,FJ497789/FJ497790/FJ497791/..._Re
oviridae-
Phytoreovirus_Phytoreovirus:0.215178):0.030053,AB254451/D86439/D13774/
..._Reoviridae-
Phytoreovirus_Phytoreovirus:0.24523)100:0.7472):0.007543):2.7e-
05,(((((((AF389452/AF389453/AF389454/..._Reoviridae-
Cypovirus_Cypovirus:0.409509,AF389462/AF389463/AF389464/..._Reoviridae
-
Cypovirus_Cypovirus:0.409509)94:0.158871,(EF634325/EF634324/EF634323/.
.._Reoviridae-

Cypovirus_Cypovirus:0.307406,AF291683/AF291684/AF291685/..._Reoviridae
-
Cypovirus_Cypovirus:0.307406)98:0.260974)98:0.138008,KC588366/KC588367
/KC588368/..._Reoviridae-
Cypovirus_Cypovirus:0.706389)83:0.030761,(KM978415/KM978416/KM978417/.
.._Reoviridae-
Dinovernavirus_Dinovernavirus:0.015269,DQ087276/DQ087277/DQ087278/..._
Reoviridae-
Dinovernavirus_Dinovernavirus:0.015269)100:0.721881)98:0.050039,AF0203
34/U66714/AF020335/..._Reoviridae-
Oryzavirus_Oryzavirus:0.787188)100:0.114796,(((((AJ294757/AJ409145/AJ4
09146/..._Reoviridae-
Fijivirus_Fijivirus:0.008028,HQ637550/HQ637551/HQ637553/..._Reoviridae
-
Fijivirus_Fijivirus:0.008028)99:0.013173,FN563989/FN563990/FN563991/..
._Reoviridae-
Fijivirus_Fijivirus:0.021201)99:0.014979,AF499925/AF499928/AF499926/..
._Reoviridae-
Fijivirus_Fijivirus:0.03618)100:0.123128,AY029520/AF049704/AF359556/..
._Reoviridae-
Fijivirus_Fijivirus:0.159308)100:0.509978,D49693/D49695/D49694/..._Reo
viridae-
Fijivirus_Fijivirus:0.669286)100:0.232698)100:0.034661,((AB102674/AB09
8022/AB102675/..._Reoviridae-
Mycoreovirus_Mycoreovirus:0.556817,AY277888/AY277889/AY277890/..._Reov
iridae-
Mycoreovirus_Mycoreovirus:0.556817)100:0.167737,(AF282467/AF282468/AF2
82469/..._Reoviridae-
Coltivirus_Coltivirus:0.007572,AF133428/AF139758/AF139759/..._Reovirid
ae-
Coltivirus_Coltivirus:0.007572)100:0.716981)100:0.212091)100:0.063355,
((((((((KU198602/KU198603/KU198604/..._Reoviridae-
Orthoreovirus_Orthoreovirus:0.112872,GQ258977/GQ258978/GQ258979/..._Re
oviridae-
Orthoreovirus_Orthoreovirus:0.112872):0.002975,HQ847903/HQ847904/HQ847
905/..._Reoviridae-
Orthoreovirus_Orthoreovirus:0.115847):0.016822,KC852153/KC852155/KC852
154/..._Reoviridae-
Orthoreovirus_Orthoreovirus:0.13267):0.048528,(((JF342668/JF342666/JF3
42667/..._Reoviridae-
Orthoreovirus_Orthoreovirus:0.003245,JF342662/JF342660/JF342661/..._Re
oviridae-
Orthoreovirus_Orthoreovirus:0.003245):0.000703,KM382261/KM382259/KM382
260/..._Reoviridae-
Orthoreovirus_Orthoreovirus:0.003948)100:0.085065,FR694191/FR694193/FR
694192/..._Reoviridae-
Orthoreovirus_Orthoreovirus:0.089013)96:0.092184)83:0.06778,GQ468270/G
Q468267/GQ468266/..._Reoviridae-
Orthoreovirus_Orthoreovirus:0.248978)100:0.23031,(KR337474/KR337473/KR
337475/..._Reoviridae-
Orthoreovirus_Orthoreovirus:0.001187,KC795566/KC795567/KC795565/..._Re
oviridae-

Orthoreovirus_Orthoreovirus:0.001187)100:0.478101)82:0.076138,(((KU194
213/KU194214/KU194215/..._Reoviridae-
Aquareovirus_Aquareovirus:0.015201,HM989930/HM989931/HM989932/..._Reov
iridae-
Aquareovirus_Aquareovirus:0.015201)100:0.075109,AF418294/AF418295/AF41
8296/..._Reoviridae-
Aquareovirus_Aquareovirus:0.09031)100:0.179956,(EF589098/EF589099/EF58
9100/..._Reoviridae-
Aquareovirus_Aquareovirus:0.034277,AF403398/AF403399/AF403400/..._Reov
iridae-
Aquareovirus_Aquareovirus:0.034277)100:0.235989)100:0.28516)100:0.4445
74,((((((AB006783/AY129662_Birnaviridae_Aquabirnavirus:0.00475,KP26866
4/KP268679_Birnaviridae_Aquabirnavirus:0.00475)91:0.008069,M18049/M587
56_Birnaviridae_Aquabirnavirus:0.012819)73:0.00434,KP268650/KP268665_B
irnaviridae_Aquabirnavirus:0.01716)99:0.088155,(AJ459382/AJ459383_Birn
aviridae_Blosnavirus:0.089418,X92760/X92761_Birnaviridae_Avibirnavirus
:0.089418):0.015897)100:0.50633,(JN589003/JN589002_Birnaviridae_Unassi
gned:0.0491,U60650/AF196645_Birnaviridae_Entomobirnavirus:0.0491)100:0
.562544)100:0.388355,((((KJ957164/KJ957165/KJ957166_Cystoviridae_Cysto
virus:0.188707,M17461/M17462/M12921_Cystoviridae_Cystovirus:0.188707)1
00:0.301776,(KX074201/KX074202/KX074203_Cystoviridae_Cystovirus:0.1703
91,AF261668/AF261667/AF261666_Cystoviridae_Cystovirus:0.170391)100:0.3
20091)86:0.349522,(FJ608823/FJ608824/FJ608825_Cystoviridae_Cystovirus:
0.304628,AF408636/AY039807/AY034425_Cystoviridae_Cystovirus:0.304628)9
5:0.535377)83:0.066906,AF226851/AF226852/AF226853_Cystoviridae_Cystovi
rus:0.90691)100:0.09309)96:0.0):0.0):0.0)100;

Figure S6 UPGMA dendrogram of Group III dsRNA viruses

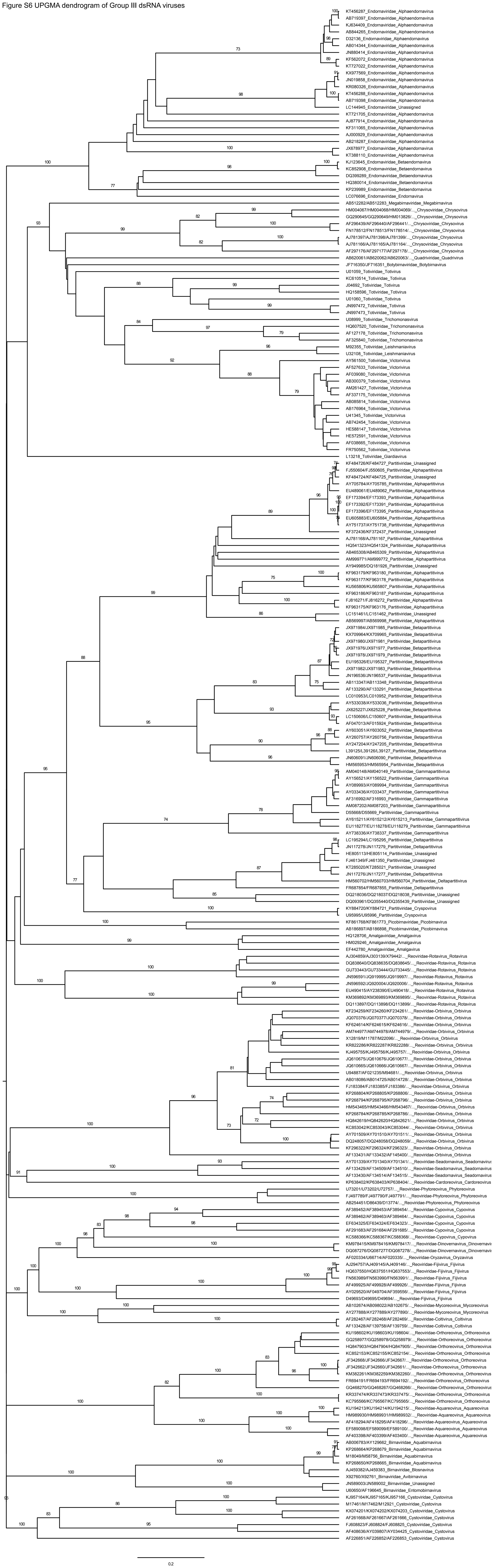

Figure S7 UPGMA dendrogram of Group IV ss(+)RNA viruses (NEWICK format)

((AF461742_Permutotetraviridae_Alphapermutotetravirus:0.999449,((((((G
U991616_Carmotetraviridae_Alphacarmotetravirus:0.757633,(KC601995_Tomb
usviridae_Umbravirus:0.642569,((((U57305_Tombusviridae_Umbravirus:0.03
3474,FJ188473_Tombusviridae_Umbravirus:0.033474)100:0.143284,(U03563_T
ombusviridae_Umbravirus:0.093437,(KJ918748_Tombusviridae_Umbravirus:0.
035622,(EU151723_Tombusviridae_Umbravirus:0.031637,(Z69910_Tombusvirid
ae_Umbravirus:0.027676,AF402620_Tombusviridae_Umbravirus:0.027676):0.0
03961):0.003986)93:0.057815)94:0.08332)100:0.275659,((((AJ557804_Tombu
sviridae_Aureusvirus:0.034066,EF589670_Tombusviridae_Aureusvirus:0.034
066)91:0.076206,((AJ243370_Tombusviridae_Aureusvirus:0.012395,X87115_T
ombusviridae_Aureusvirus:0.012395)89:0.01301,(KF482072_Tombusviridae_A
ureusvirus:0.017698,(DQ227315_Tombusviridae_Aureusvirus:0.013305,EU127
904_Tombusviridae_Aureusvirus:0.013305):0.004392)76:0.007707)97:0.0848
67)100:0.129502,(AF266518_Tombusviridae_Zeavirus:0.139404,(AY163842_To
mbusviridae_Tombusvirus:0.051498,(((X15511_Tombusviridae_Tombusvirus:0
.020444,AY830918_Tombusviridae_Tombusvirus:0.020444):0.006772,(AJ60740
2_Tombusviridae_Tombusvirus:0.021371,(X85215_Tombusviridae_Tombusvirus
:0.007118,KU187189_Tombusviridae_Tombusvirus:0.007118)94:0.014253):0.0
05844):0.003339,(M25270_Tombusviridae_Tombusvirus:0.025171,((AM711119_
Tombusviridae_Tombusvirus:0.011617,(DQ011234_Tombusviridae_Tombusvirus
:0.005109,(JQ864181_Tombusviridae_Tombusvirus:0.003143,AY100482_Tombus
viridae_Tombusvirus:0.003143)92:0.001966)71:0.006508)77:0.0097,(X62493
_Tombusviridae_Tombusvirus:0.016564,(M21958_Tombusviridae_Tombusvirus:
0.014049,(JX182425_Tombusviridae_Tombusvirus:0.008912,(JN700748_Tombus
viridae_Tombusvirus:0.002189,JX197071_Tombusviridae_Tombusvirus:0.0021
89)98:0.006723)71:0.005137):0.002514):0.004753):0.003854):0.005384)78:
0.020943)100:0.087906)85:0.10037)85:0.147716,((X14736_Tombusviridae_Ma
chlomovirus:0.199293,(EU081018_Tombusviridae_Panicovirus:0.14644,(KX75
8441_Tombusviridae_Panicovirus:0.13363,(U55002_Tombusviridae_Panicovir
us:0.02829,JX848617_Tombusviridae_Panicovirus:0.02829)100:0.10534):0.0
12809):0.052853)97:0.160822,((L18870/M88589_Tombusviridae_Dianthovirus
:0.068076,(J04357/X08021_Tombusviridae_Dianthovirus:0.018882,L07884/S4
6028_Tombusviridae_Dianthovirus:0.018882)100:0.049194)100:0.284472,(X8
3964_Tombusviridae_Avenavirus:0.335002,((X94560_Tombusviridae_Betanecr
ovirus:0.08553,(AF452884_Tombusviridae_Betanecrovirus:0.05058,U62546_T
ombusviridae_Betanecrovirus:0.05058)92:0.03495)100:0.210719,((U20976_T
ombusviridae_Gammacarmovirus:0.061212,FJ457015_Tombusviridae_Gammacarm
ovirus:0.061212)100:0.168816,(((Y13463_Tombusviridae_Gallantivirus:0.0
72414,FJ768020_Tombusviridae_Macanavirus:0.072414)100:0.084003,(X85989
_Tombusviridae_Alphanecrovirus:0.036555,(M33002_Tombusviridae_Alphanec
rovirus:0.028571,(AY616760_Tombusviridae_Alphanecrovirus:0.022867,KP90
1095_Tombusviridae_Unassigned:0.022867)93:0.005704)70:0.007984)100:0.1
19861):0.066743,((M29671_Tombusviridae_Gammacarmovirus:0.069957,AB0869
51_Tombusviridae_Gammacarmovirus:0.069957)99:0.114942,(D86123_Tombusvi
ridae_Betacarmovirus:0.162056,(((AY038066_Tombusviridae_Pelarspovirus:
0.039338,AY038068_Tombusviridae_Pelarspovirus:0.039338)91:0.038916,(AY
613852_Tombusviridae_Pelarspovirus:0.067078,(AY038069_Tombusviridae_Pe
larspovirus:0.054313,KC166238_Tombusviridae_Pelarspovirus:0.054313):0.
012764):0.011176)89:0.060535,((X86448_Tombusviridae_Betacarmovirus:0.0

89214,(L16015_Tombusviridae_Betacarmovirus:0.053872,M22445_Tombusvirid
ae_Betacarmovirus:0.053872)93:0.035341):0.015341,(GQ244431_Tombusvirid
ae_Alphacarmovirus:0.089831,((KX184304_Tombusviridae_Unassigned:0.0572
25,HM640935_Tombusviridae_Unassigned:0.057225):0.027356,(EF207438_Tomb
usviridae_Alphacarmovirus:0.078223,((HQ677625_Tombusviridae_Alphacarmo
virus:0.043273,AJ514833_Tombusviridae_Alphacarmovirus:0.043273)72:0.02
4266,(U72332_Tombusviridae_Alphacarmovirus:0.056575,(DQ219415_Tombusvi
ridae_Alphacarmovirus:0.0483,(AF192772_Tombusviridae_Alphacarmovirus:0
.015764,X02986_Tombusviridae_Alphacarmovirus:0.015764)100:0.032536):0.
008275):0.010964):0.010683):0.006359):0.00525):0.014723):0.034234):0.0
23267):0.022844):0.03826):0.006868):0.066221):0.038753):0.017546):0.00
7567):0.027375):0.064927)98:0.101477,(KP638562_Luteoviridae_Unassigned
:0.186882,((AF441393_Luteoviridae_Luteovirus:0.088617,AB038147_Luteovi
ridae_Luteovirus:0.088617):0.074375,((EU024678_Luteoviridae_Luteovirus
:0.091413,KX159470_Luteoviridae_Unassigned:0.091413):0.041351,(KC57199
9_Luteoviridae_Luteovirus:0.041327,(AF218798_Luteoviridae_Luteovirus:0
.024643,(D11028_Luteoviridae_Luteovirus:0.012782,(AY220739_Luteovirida
e_Luteovirus:0.011997,X07653_Luteoviridae_Luteovirus:0.011997):0.00078
4)99:0.011862)98:0.016684)99:0.091437):0.030227):0.02389)100:0.367012)
:0.088675):0.115064):0.16942,(((HF679486_Luteoviridae_Unassigned:0.249
716,(KU297983_Luteoviridae_Enamovirus:0.033107,(HM439775_Luteoviridae_
Enamovirus:0.00367,L04573_Luteoviridae_Enamovirus:0.00367)100:0.029437
)100:0.216609)96:0.165778,(((KF427701_Luteoviridae_Polerovirus:0.12884
1,KJ885302_Luteoviridae_Polerovirus:0.128841)83:0.090805,(AF157029_Lut
eoviridae_Polerovirus:0.208924,((D00530_Luteoviridae_Polerovirus:0.154
173,(AY695933_Luteoviridae_Polerovirus:0.136481,((L25299_Luteoviridae_
Polerovirus:0.039362,(AF235168_Luteoviridae_Polerovirus:0.031207,FM865
413_Luteoviridae_Polerovirus:0.031207):0.008156)99:0.085908,((HQ388348
_Luteoviridae_Polerovirus:0.053721,X13063_Luteoviridae_Polerovirus:0.0
53721)75:0.053803,(AF352024_Luteoviridae_Polerovirus:0.088827,(X83110_
Luteoviridae_Polerovirus:0.022833,AF473561_Luteoviridae_Polerovirus:0.
022833)99:0.065994):0.018697):0.017747):0.011211):0.017692):0.017853,(
JQ700308_Luteoviridae_Polerovirus:0.154449,((KM233705_Luteoviridae_Pol
erovirus:0.125845,(KT992824_Luteoviridae_Polerovirus:0.039977,(KT96208
9_Luteoviridae_Polerovirus:0.026721,KC921392_Luteoviridae_Polerovirus:
0.026721)90:0.013257)99:0.085868):0.021324,(((KU315178_Luteoviridae_Po
lerovirus:0.109574,KT962999_Luteoviridae_Polerovirus:0.109574):0.00733
5,(GU167940_Luteoviridae_Polerovirus:0.093584,(AY956384_Luteoviridae_P
olerovirus:0.023701,LC192169_Luteoviridae_Polerovirus:0.023701)99:0.06
9883):0.023324):0.012346,(KT868949_Luteoviridae_Unassigned:0.122285,((
X76931_Luteoviridae_Polerovirus:0.030283,EU000534_Luteoviridae_Polerov
irus:0.030283)95:0.071168,(AB594828_Luteoviridae_Polerovirus:0.069727,
EF529624_Luteoviridae_Polerovirus:0.069727):0.031725):0.020833):0.0069
7):0.017915):0.007279):0.017577):0.036898):0.010722)93:0.169336,AJ8674
90_Sobemoviridae_Polemovirus:0.388983):0.026512)81:0.240827,(((((((((
(((AF208001_Sobemoviridae_Sobemovirus:0.047882,LC019764_Sobemoviridae_
Sobemovirus:0.047882):0.000763,JQ782213_Sobemoviridae_Sobemovirus:0.04
8645):0.007361,AM940437_Sobemoviridae_Sobemovirus:0.056005):0.00435,JX
123318_Sobemoviridae_Sobemovirus:0.060355):0.029173,M23021_Sobemovirid
ae_Sobemovirus:0.089529):0.007699,KC577470_Sobemoviridae_Sobemovirus:0
.097227):0.016859,((DQ875594_Sobemoviridae_Sobemovirus:0.024761,AY0042
91_Sobemoviridae_Sobemovirus:0.024761)91:0.053901,JF495127_Sobemovirid

ae_Sobemovirus:0.078663)79:0.035424):0.010431,HM754263_Sobemoviridae_S
obemovirus:0.124518):0.015467,KC778720_Sobemoviridae_Sobemovirus:0.139
985):0.033915,LC081344_Sobemoviridae_Sobemovirus:0.1739):0.009075,((EF
091714_Sobemoviridae_Sobemovirus:0.065524,JN620802_Sobemoviridae_Sobem
ovirus:0.065524)74:0.04837,KC577469_Sobemoviridae_Sobemovirus:0.113894
)75:0.069081):0.079487,((AJ608206_Sobemoviridae_Sobemovirus:0.110838,A
M990928_Sobemoviridae_Sobemovirus:0.110838)76:0.082597,Z48630_Sobemovi
ridae_Sobemovirus:0.193436):0.069026)100:0.324224,U07551_Barnaviridae_
Barnavirus:0.586686)79:0.069635)71:0.270732):0.047772,((((((X77156/X1
5959_Nodaviridae_Alphanodavirus:0.004416,X02396/X00956_Nodaviridae_Alp
hanodavirus:0.004416)100:0.034167,AF329080/X15960_Nodaviridae_Alphanod
avirus:0.038583)98:0.094498,AF174533/AF174534_Nodaviridae_Alphanodavir
us:0.133081)80:0.165876,(HQ259079/EF137180_Nodaviridae_Unassigned:0.03
5644,AY222839/AY222840_Nodaviridae_Unassigned:0.035644)100:0.263313)76
:0.286947,AF171942/AF171943_Nodaviridae_Alphanodavirus:0.585904):0.073
012,((((EU826137/EU826138_Nodaviridae_Betanodavirus:0.00042,EU236146/E
U236147_Nodaviridae_Betanodavirus:0.00042)99:0.013567,AB056571/AB05657
2_Nodaviridae_Betanodavirus:0.013987):0.008984,(FJ803911/FJ803923_Noda
viridae_Betanodavirus:0.004882,AY324869/AY324870_Nodaviridae_Betanodav
irus:0.004882)88:0.018088):0.005481,EU236148/EU236149_Nodaviridae_Beta
nodavirus:0.028451)100:0.630464)82:0.091227,((HM030972/HM030973_Nodavi
ridae_Unassigned:0.070612,JQ943579/JQ943580_Nodaviridae_Unassigned:0.0
70612)77:0.06949,HM030970/HM030971_Nodaviridae_Unassigned:0.140102)100
:0.61004)99:0.224682):0.008322,(((((((((((((KJ701554_Caliciviridae_V
esivirus:0.033346,KM244552_Caliciviridae_Vesivirus:0.033346):0.001185,
JX847605_Caliciviridae_Vesivirus:0.034531)99:0.020297,AB070225_Caliciv
iridae_Vesivirus:0.054828)78:0.016941,(((EF195384_Caliciviridae_Vesivi
rus:0.022997,AJ866991_Caliciviridae_Vesivirus:0.022997):0.011584,U7687
4_Caliciviridae_Vesivirus:0.034581):0.007711,AF321298_Caliciviridae_Ve
sivirus:0.042291)97:0.029477)86:0.02865,L40021_Caliciviridae_Vesivirus
:0.100419)100:0.26517,((((AY694184_Caliciviridae_Sapovirus:0.051371,A
B775659_Caliciviridae_Sapovirus:0.051371)87:0.030963,AF182760_Calicivi
ridae_Sapovirus:0.082334):0.02293,(AY237420_Caliciviridae_Sapovirus:0.
019318,AY603425_Caliciviridae_Sapovirus:0.019318)100:0.085945):0.03945
3,JN899075_Caliciviridae_Sapovirus:0.144716)89:0.081953,KJ473715_Calic
iviridae_Unassigned:0.226669)72:0.13892):0.037714,((M67473_Caliciviri d
ae_Lagovirus:0.024065,Z69620_Caliciviridae_Lagovirus:0.024065):0.00715
3,EU871528_Caliciviridae_Lagovirus:0.031218)100:0.372085):0.017594,(((
AB117797_Caliciviridae_Unassigned:0.016632,AY082891_Caliciviridae_Nebo
virus:0.016632)84:0.012598,DQ013304_Caliciviridae_Nebovirus:0.02923)10
0:0.058097,KT119483_Caliciviridae_Nebovirus:0.087327)100:0.33357):0.09
3358,((((JQ613567_Caliciviridae_Norovirus:0.043351,AB044366_Calicivir
idae_Norovirus:0.043351)92:0.023305,JX145650_Caliciviridae_Norovirus:0
.066656):0.005326,(KX396056_Caliciviridae_Norovirus:0.039926,M87661_Ca
liciviridae_Norovirus:0.039926)92:0.032056)82:0.036383,DQ285629_Calici
viridae_Norovirus:0.108366)100:0.328861,FJ355928_Caliciviridae_Unassig
ned:0.437227):0.077028):0.117809,KJ577139_Caliciviridae_Unassigned:0.6
32064)85:0.059267,((KX024775_Solinviviridae_Nyfulvavirus:0.578599,FJ52
8584_Solinviviridae_Invictavirus:0.578599)80:0.107808,(AY337486_Marnav
iridae_Marnavirus:0.635748,((((((((((((((((AF414373_Picornaviridae_E
nterovirus:0.027336,AF326766_Picornaviridae_Enterovirus:0.027336):0.00
2728,KJ420749_Picornaviridae_Enterovirus:0.030065):0.011334,(AF326759_

Picornaviridae_Enterovirus:0.015711,AF201894_Picornaviridae_Enteroviru
s:0.015711)99:0.025687):0.002453,((AY421760_Picornaviridae_Enterovirus
:0.021673,U05876_Picornaviridae_Enterovirus:0.021673)93:0.012969,(M884
83_Picornaviridae_Enterovirus:0.016353,M16560_Picornaviridae_Enterovir
us:0.016353)98:0.018289):0.009209):0.003449,((((KU955844_Picornavirida
e_Enterovirus:0.01945,DQ092770_Picornaviridae_Enterovirus:0.01945):0.0
06717,KP345887_Picornaviridae_Enterovirus:0.026167):0.003748,D00214_Pi
cornaviridae_Enterovirus:0.029914):0.004798,AY462106_Picornaviridae_En
terovirus:0.034712):0.012588):0.004413,(D00820_Picornaviridae_Enterovi
rus:0.020417,AY426531_Picornaviridae_Enterovirus:0.020417)100:0.031296
):0.013181,(((((DQ473485_Picornaviridae_Enterovirus:0.030616,K02121_Pi
cornaviridae_Enterovirus:0.030616)87:0.019004,AF363453_Picornaviridae_
Enterovirus:0.049619):0.003421,(A10937_Picornaviridae_Enterovirus:0.03
9488,FJ445111_Picornaviridae_Enterovirus:0.039488):0.013553):0.002294,
(EF077279_Picornaviridae_Enterovirus:0.040628,EF582385_Picornaviridae_
Enterovirus:0.040628):0.014707):0.007693,V01149_Picornaviridae_Enterov
irus:0.063028):0.001866):0.002387,KU587555_Picornaviridae_Enterovirus:
0.067281):0.008216,((((HQ595342_Picornaviridae_Unassigned:0.02663,HQ59
5340_Picornaviridae_Unassigned:0.02663)95:0.014947,AY064708_Picornavir
idae_Sapelovirus:0.041577):0.009786,AY563023_Picornaviridae_Sapeloviru
s:0.051364):0.002899,((AF406813_Picornaviridae_Sapelovirus:0.045753,KP
233897_Picornaviridae_Rabovirus:0.045753):0.003503,(JN572115_Picornavi
ridae_Unassigned:0.037529,HQ595344_Picornaviridae_Unassigned:0.037529)
:0.011728):0.005006):0.021234):0.019039,(JN674502_Picornaviridae_Unass
igned:0.083695,FR727144_Picornaviridae_Unassigned:0.083695):0.010841)8
9:0.137421,((((((((((KJ194505_Picornaviridae_Cosavirus:0.016942,FJ438
908_Picornaviridae_Cosavirus:0.016942)100:0.017506,FJ438907_Picornavir
idae_Cosavirus:0.034448):0.005315,(KM516909_Picornaviridae_Cosavirus:0
.036659,FJ555055_Picornaviridae_Cosavirus:0.036659):0.003104):0.007047
,FJ438902_Picornaviridae_Cosavirus:0.04681):0.009116,JN867758_Picornav
iridae_Cosavirus:0.055926)81:0.011959,((X96870_Picornaviridae_Aphthovi
rus:0.004616,DQ272578_Picornaviridae_Aphthovirus:0.004616)100:0.060437
,JQ864242_Picornaviridae_Cardiovirus:0.065053):0.002831):0.005161,X968
71_Picornaviridae_Erbovirus:0.073045):0.001851,((((EF165067_Picornavir
idae_Cardiovirus:0.015733,EU376394_Picornaviridae_Cardiovirus:0.015733
)100:0.021361,X56019_Picornaviridae_Cardiovirus:0.037094)97:0.0293,M81
861_Picornaviridae_Cardiovirus:0.066394):0.003903,(KP100644_Picornavir
idae_Mischivirus:0.040958,JQ814851_Picornaviridae_Mischivirus:0.040958
)91:0.02934):0.004599):0.00994,((KF958461_Picornaviridae_Mosavirus:0.0
4242,JF973687_Picornaviridae_Mosavirus:0.04242)99:0.035917,KM873611_Pi
cornaviridae_Torchivirus:0.078337):0.006499):0.011884,(((EU236594_Pico
rnaviridae_Aphthovirus:0.056329,KP236128_Picornaviridae_Aphthovirus:0.
056329)75:0.00828,(AY593829_Picornaviridae_Aphthovirus:0.008734,AF3081
57_Picornaviridae_Aphthovirus:0.008734)100:0.055875)85:0.018957,(KM589
358_Picornaviridae_Unassigned:0.062762,DQ641257_Picornaviridae_Senecav
irus:0.062762):0.020804):0.013154)76:0.092875,((((KJ950971_Picornaviri
dae_Hunnivirus:0.027482,JQ941880_Picornaviridae_Hunnivirus:0.027482)10
0:0.038935,(AJ011380_Picornaviridae_Teschovirus:0.011331,AF231769_Pico
rnaviridae_Teschovirus:0.011331)100:0.055085):0.008001,KM396708_Picorn
aviridae_Unassigned:0.074417):0.032613,KM396707_Picornaviridae_Unassig
ned:0.107031)77:0.082564):0.042362):0.041456,(((((((AB040749_Picornav
iridae_Kobuvirus:0.00272,AB010145_Picornaviridae_Kobuvirus:0.00272)100

:0.017707,JF755427_Picornaviridae_Kobuvirus:0.020427)99:0.012324,AB084
788_Picornaviridae_Kobuvirus:0.032751):0.002458,(((JN630514_Picornavir
idae_Kobuvirus:0.010366,KP260507_Picornaviridae_Kobuvirus:0.010366):0.
003111,EU787450_Picornaviridae_Kobuvirus:0.013477)92:0.014288,KF793927
_Picornaviridae_Kobuvirus:0.027765)83:0.007444)100:0.025148,(LC055960_
Picornaviridae_Kobuvirus:0.048869,LC055961_Picornaviridae_Kobuvirus:0.
048869):0.011489):0.015799,((GQ179640_Picornaviridae_Salivirus:0.01442
4,GQ184145_Picornaviridae_Salivirus:0.014424)94:0.011923,KM023140_Pico
rnaviridae_Salivirus:0.026347)100:0.049809):0.009081,((((KF741227_Pic
ornaviridae_Sicinivirus:0.016437,KP779642_Picornaviridae_Sicinivirus:0
.016437)91:0.012252,KF979331_Picornaviridae_Unassigned:0.028689)99:0.0
37283,KT325852_Picornaviridae_Unassigned:0.065971):0.002097,((KT880666
_Picornaviridae_Gallivirus:0.008317,KF979337_Picornaviridae_Gallivirus
:0.008317)100:0.035329,JQ691613_Picornaviridae_Gallivirus:0.043646)91:
0.024423):0.002868,(KF387721_Picornaviridae_Sakobuvirus:0.053997,GU182
406_Picornaviridae_Passerivirus:0.053997):0.01694):0.0143):0.015673,((
GU182410_Picornaviridae_Oscivirus:0.048794,GU182408_Picornaviridae_Osc
ivirus:0.048794)96:0.037222,KJ415177_Picornaviridae_Unassigned:0.08601
6):0.014893)93:0.172503):0.040117,((((KX783423_Picornaviridae_Rosaviru
s:0.032108,JF973686_Picornaviridae_Rosavirus:0.032108):0.007046,KJ1581
69_Picornaviridae_Rosavirus:0.039154)70:0.005467,KX783425_Picornavirid
ae_Rosavirus:0.044621)100:0.219693,JN819202_Picornaviridae_Dicipivirus
:0.264314):0.049215):0.011344,((((KF979335_Picornaviridae_Unassigned:0
.012743,HM751199_Picornaviridae_Megrivirus:0.012743):0.008481,KF961188
_Picornaviridae_Megrivirus:0.021224)100:0.033909,(KF979336_Picornaviri
dae_Unassigned:0.011302,KF961187_Picornaviridae_Megrivirus:0.011302)10
0:0.043831):0.006258,KC663628_Picornaviridae_Megrivirus:0.061391)100:0
.263482):0.054705,(((((((KT877158_Picornaviridae_Hepatovirus:0.019974
,KT452714_Picornaviridae_Hepatovirus:0.019974)88:0.01207,KT452691_Pico
rnaviridae_Hepatovirus:0.032043):0.014443,(KT452730_Picornaviridae_Hep
atovirus:0.040613,M14707_Picornaviridae_Hepatovirus:0.040613):0.005873
):0.001148,(((KT452685_Picornaviridae_Hepatovirus:0.030795,KT452637_Pi
cornaviridae_Hepatovirus:0.030795)74:0.008646,KT452742_Picornaviridae_
Hepatovirus:0.03944):0.002608,KR703607_Picornaviridae_Hepatovirus:0.04
2048):0.005586):0.011513,KT452658_Picornaviridae_Hepatovirus:0.059147)
:0.006315,KT452735_Picornaviridae_Hepatovirus:0.065462)97:0.035509,AJ2
25173_Picornaviridae_Tremovirus:0.100971)100:0.239883,KP230449_Picorna
viridae_Harkavirus:0.340854):0.038725):0.035354,(((((((AJ005695_Picor
naviridae_Parechovirus:0.028472,S45208_Picornaviridae_Parechovirus:0.0
28472)100:0.026149,KF006989_Picornaviridae_Parechovirus:0.054621)91:0.
020622,(HF677705_Picornaviridae_Parechovirus:0.038526,AF327920_Picorna
viridae_Parechovirus:0.038526)100:0.036717):0.011295,(AB937989_Picorna
viridae_Unassigned:0.084219,KC843627_Picornaviridae_Potamipivirus:0.08
4219):0.00232)78:0.035173,JQ316470_Picornaviridae_Pasivirus:0.121712)8
7:0.124564,(((((KT880669_Picornaviridae_Unassigned:0.011165,KF979334_P
icornaviridae_Avisivirus:0.011165)100:0.055368,KF979333_Picornaviridae
_Avisivirus:0.066534):0.006041,KC614703_Picornaviridae_Avisivirus:0.07
2575):0.015426,(KJ000696_Picornaviridae_Unassigned:0.082625,DQ226541_P
icornaviridae_Avihepatovirus:0.082625):0.005376)84:0.042488,KM203656_P
icornaviridae_Unassigned:0.130489)83:0.115787):0.065855,(((KF183915_Pi
cornaviridae_Limnipivirus:0.006934,KC465953_Picornaviridae_Limnipiviru
s:0.006934)100:0.054296,KF306267_Picornaviridae_Limnipivirus:0.06123)7

0:0.011287,JX134222_Picornaviridae_Limnipivirus:0.072517)100:0.239613)
:0.026764,(KC935379_Picornaviridae_Kunsagivirus:0.122614,EU142040_Pico
rnaviridae_Aquamavirus:0.122614)97:0.21628):0.076039)83:0.120883,KP770
140_Picornaviridae_Ampivirus:0.535815):0.076438,((((AF323747_Iflavirid
ae_Iflavirus:0.02222,AY365064_Iflaviridae_Iflavirus:0.02222)100:0.0612
56,(KJ186788_Iflaviridae_Iflavirus:0.01238,JN870848_Iflaviridae_Iflavi
rus:0.01238)100:0.071095)100:0.501686,((((((((((KP217032_Iflaviridae_
Unassigned:0.027455,LC068762_Iflaviridae_Iflavirus:0.027455):0.001262,
(KJ629170_Iflaviridae_Iflavirus:0.023942,KF751885_Iflaviridae_Iflaviru
s:0.023942):0.004775):0.011456,KJ679438_Iflaviridae_Iflavirus:0.040172
)100:0.057034,(KX779454_Iflaviridae_Unassigned:0.088836,EF517277_Iflav
iridae_Iflavirus:0.088836):0.00837):0.008794,((EU035616_Iflaviridae_If
lavirus:0.071182,KU645789_Iflaviridae_Iflavirus:0.071182):0.021616,(KP
866792_Iflaviridae_Unassigned:0.064826,KF500002_Iflaviridae_Iflavirus:
0.064826):0.027971):0.013202)71:0.09385,(((AY251269_Iflaviridae_Iflav
irus:0.017353,AJ489744_Iflaviridae_Iflavirus:0.017353)100:0.061537,KX4
32289_Iflaviridae_Unassigned:0.07889):0.009378,AB826460_Iflaviridae_Un
assigned:0.088268)70:0.0375,AB826459_Iflaviridae_Unassigned:0.125768)7
0:0.074082):0.078568,(((KM272628_Iflaviridae_Unassigned:0.002801,KF934
491_Iflaviridae_Unassigned:0.002801)100:0.047251,AB766259_Iflaviridae_
Iflavirus:0.050052)99:0.142174,KF843822_Iflaviridae_Iflavirus:0.192227
)71:0.086191):0.079807,(((KP714074_Iflaviridae_Unassigned:0.071956,AF0
92924_Iflaviridae_Iflavirus:0.071956):0.010082,KF699344_Iflaviridae_Un
assigned:0.082039)79:0.030352,JF720348_Iflaviridae_Iflavirus:0.11239)9
3:0.245834):0.080557,((KR534892_Iflaviridae_Unassigned:0.06095,JN09170
7_Iflaviridae_Iflavirus:0.06095)97:0.040472,AB000906_Iflaviridae_Iflav
irus:0.101422)100:0.33736):0.038967,((((((((KF500001_Dicistroviridae_
Unassigned:0.019534,AY275710_Dicistroviridae_Aparavirus:0.019534):0.00
3918,EF219380_Dicistroviridae_Aparavirus:0.023453)89:0.01166,AF150629_
Dicistroviridae_Aparavirus:0.035112)100:0.052618,AY634314_Dicistroviri
dae_Aparavirus:0.08773)97:0.090474,(((AF014388_Dicistroviridae_Cripavi
rus:0.036472,AF218039_Dicistroviridae_Cripavirus:0.036472)88:0.010105,
KU169878_Dicistroviridae_Cripavirus:0.046577)91:0.022697,KT873140_Dici
stroviridae_Unassigned:0.069275)100:0.108929)70:0.092754,(AF022937_Dic
istroviridae_Cripavirus:0.070654,AF536531_Dicistroviridae_Cripavirus:0
.070654)100:0.200304):0.060376,((((AB006531_Dicistroviridae_Triatoviru
s:0.072822,AF183905_Dicistroviridae_Triatovirus:0.072822)80:0.036914,A
B017037_Dicistroviridae_Triatovirus:0.109737)90:0.159754,KJ802403_Dici
stroviridae_Cripavirus:0.26949):0.051117,DQ288865_Dicistroviridae_Tria
tovirus:0.320608):0.010726):0.038726,(KX657786_Dicistroviridae_Unassig
ned:0.015062,KX657784_Dicistroviridae_Unassigned:0.015062)100:0.354999
):0.013378,((HQ113110_Dicistroviridae_Unassigned:0.026429,AF277675_Dic
istroviridae_Aparavirus:0.026429)99:0.016047,HM777507_Dicistroviridae_
Aparavirus:0.042476)100:0.340963)76:0.09431):0.049411,(((((((((((X64
886/M14913_Secoviridae_Comovirus:0.033896,GU810903/GU810904_Secovirida
e_Comovirus:0.033896):0.011768,X00206/X00729_Secoviridae_Comovirus:0.0
45663):0.005201,(FJ712026/FJ712027_Secoviridae_Comovirus:0.041428,AB29
5643/AB295644_Secoviridae_Comovirus:0.041428):0.009436):0.005647,(U708
66/M62738_Secoviridae_Comovirus:0.028759,AF394606/AF394607_Secoviridae
_Comovirus:0.028759)89:0.027753):0.006997,(((EU421059/EU421060_Secovir
idae_Comovirus:0.015887,AB054688/AB054689_Secoviridae_Comovirus:0.0158
87)100:0.031121,KP404602/KP404603_Secoviridae_Comovirus:0.047008):0.01

3527,M83830/M83309_Secoviridae_Comovirus:0.060535):0.002974)95:0.10912
1,(((((EU158250/EU158249_Secoviridae_Fabavirus:0.011549,AB084452/AB084
453_Secoviridae_Fabavirus:0.011549)100:0.02267,AB084450/AB084451_Secov
iridae_Fabavirus:0.034219):0.010383,EU881936/EU881937_Secoviridae_Faba
virus:0.044602):0.006459,AF225953/AF225954_Secoviridae_Fabavirus:0.051
061):0.013737,KC595304/KC595305_Secoviridae_Fabavirus:0.064798)95:0.10
7831)93:0.219766,((((KM229700/KM229701_Secoviridae_Torradovirus:0.0464
92,KF533719/KF533720_Secoviridae_Torradovirus:0.046492)73:0.012379,KC8
55266/KC855267_Secoviridae_Torradovirus:0.05887)82:0.029281,((KC999058
/KC999059_Secoviridae_Torradovirus:0.017078,EF681764/EF681765_Secoviri
dae_Torradovirus:0.017078)100:0.038673,DQ388879/DQ388880_Secoviridae_T
orradovirus:0.055751)93:0.032401):0.016956,KU052530/KU052531_Secovirid
ae_Torradovirus:0.105107)100:0.287288):0.011572,(((M95497_Secoviridae_
Waikavirus:0.047008,KT238881_Secoviridae_Waikavirus:0.047008)87:0.0153
66,U67839_Secoviridae_Waikavirus:0.062374)100:0.312871,(D14066_Secovir
idae_Sequivirus:0.02208,EU980442_Secoviridae_Sequivirus:0.02208)100:0.
353165):0.028723):0.014449,((KU215538/KU215539_Secoviridae_Unassigned:
0.205346,JN052073/JN052074_Secoviridae_Unassigned:0.205346):0.086398,(
AJ311875/AJ311876_Secoviridae_Unassigned:0.123423,DQ344639/DQ344640_Se
coviridae_Unassigned:0.123423)91:0.168321):0.126673):0.005255,(((AJ621
357/AJ621358_Secoviridae_Cheravirus:0.033568,AB030940/AB030941_Secovir
idae_Cheravirus:0.033568)77:0.006707,KT692952/KT692953_Secoviridae_Che
ravirus:0.040276)100:0.1901,JQ437415/JQ581051_Secoviridae_Cheravirus:0
.230376)79:0.193295):0.046764,(((((((((KC832887/KC832892_Secoviridae_
Nepovirus:0.025401,JX304792/JQ670669_Secoviridae_Nepovirus:0.025401):0
.004684,KJ556849/KJ556850_Secoviridae_Nepovirus:0.030085):0.011952,U50
869/AY363727_Secoviridae_Nepovirus:0.042037)100:0.063572,AB518485/AB51
8486_Secoviridae_Nepovirus:0.105609)89:0.133007,AY303787/AY303788_Seco
viridae_Nepovirus:0.238616):0.016048,(((HE613269/AY291208_Secoviridae_
Nepovirus:0.028877,AY303786/AY017339_Secoviridae_Nepovirus:0.028877):0
.007871,D00915/X16907_Secoviridae_Nepovirus:0.036748)100:0.194717,KC90
4083/KC904084_Secoviridae_Nepovirus:0.231465):0.0232):0.024669,(FN6919
34/FN691935_Secoviridae_Nepovirus:0.224411,AF368272/AF020051_Secovirid
ae_Nepovirus:0.224411):0.054923):0.018247,(L19655/D12477_Secoviridae_N
epovirus:0.265904,FR851461/FR851462_Secoviridae_Nepovirus:0.265904):0.
031678):0.040782,((KY646466/KJ572573_Secoviridae_Nepovirus:0.035965,AB
649296/AB649297_Secoviridae_Nepovirus:0.035965)100:0.036562,KX424571/K
X424572_Secoviridae_Nepovirus:0.072527)100:0.265836):0.011211,(((X1534
6/X15163_Secoviridae_Nepovirus:0.041322,HE774604/AY291207_Secoviridae_
Nepovirus:0.041322):0.003488,(AY157993/AY157994_Secoviridae_Nepovirus:
0.025203,D00322/X04062_Secoviridae_Nepovirus:0.025203)93:0.019606)100:
0.039793,AB073147/AB073148_Secoviridae_Nepovirus:0.084602)100:0.264972
)89:0.120861):0.033655,AY860978/AY860979_Secoviridae_Unassigned:0.5040
9):0.002185,AB009958/AB009959_Secoviridae_Sadwavirus:0.506275):0.02088
5):0.058002):0.018817,((((((MF041808_Polycipiviridae_Sopolycivirus:0.0
34048,KX883910_Polycipiviridae_Sopolycivirus:0.034048)75:0.005242,LA86
6448_Polycipiviridae_Sopolycivirus:0.03929)100:0.081681,(MF041810_Poly
cipiviridae_Sopolycivirus:0.03291,MF041809_Polycipiviridae_Sopolycivir
us:0.03291)100:0.08806)100:0.170897,LH935078/LH935077_Polycipiviridae_
Sopolycivirus:0.291868):0.024943,(((MF041813_Polycipiviridae_Sopolyciv
irus:0.054867,LA858223_Polycipiviridae_Sopolycivirus:0.054867)96:0.027
711,LI719284_Polycipiviridae_Sopolycivirus:0.082578)91:0.044209,(MF041

812_Polycipiviridae_Sopolycivirus:0.03048,LI526777_Polycipiviridae_Sopolycivirus:0.03048)100:0.096307)96:0.190023)100:0.235972,KX883940_Polycipiviridae_Hupolycivirus:0.552782):0.051196):0.008275):0.023495):0.050659):0.004925):0.054466,(KX883688_Polycipiviridae_Spipolycivirus:0.549675,KA182589_Polycipiviridae_Spipolycivirus:0.549675)99:0.196122)100:0.123235,(((((((((((((((((((((EU250210_Potyviridae_Potyvirus:0.005578,AF538686_Potyviridae_Potyvirus:0.005578)98:0.014652,JX867236_Potyviridae_Potyvirus:0.020231):0.002342,(AJ243766_Potyviridae_Potyvirus:0.018116,AJ437280_Potyviridae_Potyvirus:0.018116):0.004456):0.000869,EU564817_Potyviridae_Potyvirus:0.023442):0.002782,((KT834407_Potyviridae_Unassigned:0.017128,AF501591_Potyviridae_Potyvirus:0.017128):0.005436,AB541985_Potyviridae_Potyvirus:0.022564):0.00366):0.002827,(((((X97895_Potyviridae_Potyvirus:0.004121,AJ889867_Potyviridae_Potyvirus:0.004121)91:0.005565,AY166866_Potyviridae_Potyvirus:0.009686)82:0.009197,X12456_Potyviridae_Potyvirus:0.018883):0.004387,U09509_Potyviridae_Potyvirus:0.02327):0.003306,KF649336_Potyviridae_Potyvirus:0.026576):0.002475):0.002724,M96425_Potyviridae_Potyvirus:0.031775):0.001827,((((JQ314463_Potyviridae_Potyvirus:0.021995,AJ516010_Potyviridae_Potyvirus:0.021995):0.001766,AB551370_Potyviridae_Potyvirus:0.023761):0.003431,AJ437279_Potyviridae_Potyvirus:0.027191):0.004171,KU685505_Potyviridae_Potyvirus:0.031362):0.002239):0.002231,AJ890348_Potyviridae_Potyvirus:0.035833):0.006368,(((((((KC691259_Potyviridae_Potyvirus:0.015074,JQ326210_Potyviridae_Potyvirus:0.015074)73:0.005492,LC159494_Potyviridae_Unassigned:0.020567):0.006309,AF169561_Potyviridae_Potyvirus:0.026876):0.001216,(AB016500_Potyviridae_Potyvirus:0.019312,AB027007_Potyviridae_Potyvirus:0.019312):0.00878):0.001788,AJ316084_Potyviridae_Potyvirus:0.029879):0.000855,(JX291161_Potyviridae_Potyvirus:0.01592,JQ609095_Potyviridae_Potyvirus:0.01592)84:0.014814):0.003047,((AJ564636_Potyviridae_Potyvirus:0.028315,KP769852_Potyviridae_Potyvirus:0.028315):0.001361,KX013584_Potyviridae_Potyvirus:0.029676):0.004105):0.003009,KT222674_Potyviridae_Potyvirus:0.036791):0.00541):0.002499,(((((X67673_Potyviridae_Potyvirus:0.004485,AY027810_Potyviridae_Potyvirus:0.004485)80:0.004573,AY010722_Potyviridae_Potyvirus:0.009058)100:0.017816,(KC345607_Potyviridae_Potyvirus:0.005168,KC345605_Potyviridae_Potyvirus:0.005168)100:0.021706):0.003461,((BD171712_Potyviridae_Potyvirus:5.7e-
05,AB088221_Potyviridae_Potyvirus:5.7e-
05)100:0.027097,EF579955_Potyviridae_Potyvirus:0.027154):0.003181):0.012057,FM206346_Potyviridae_Potyvirus:0.042392):0.002307):0.002723,(((AJ131403_Potyviridae_Potyvirus:0.016149,AF543212_Potyviridae_Potyvirus:0.016149):0.003657,AJ296311_Potyviridae_Potyvirus:0.019806)80:0.022944,X04083_Potyviridae_Potyvirus:0.04275):0.004672):0.000224,(KT633868_Potyviridae_Unassigned:0.038063,EU410442_Potyviridae_Potyvirus:0.038063):0.009583):0.002412,(((((((((((U19287_Potyviridae_Potyvirus:0.001617,AY138897_Potyviridae_Potyvirus:0.001617)100:0.012766,DQ851493_Potyviridae_Potyvirus:0.014383):0.003866,AF127929_Potyviridae_Potyvirus:0.018249):0.002176,(HQ122652_Potyviridae_Potyvirus:0.017335,HQ161081_Potyviridae_Potyvirus:0.017335):0.003089):0.000701,JQ807999_Potyviridae_Potyvirus:0.021126):0.002175,((((D00507_Potyviridae_Potyvirus:0.008399,EF105297_Potyviridae_Potyvirus:0.008399)75:0.003272,AJ507388_Potyviridae_Potyvirus:0.011671):0.003855,AY656816_Potyviridae_Potyvirus:0.015525):0.006018,AY437609_Potyviridae_Potyvirus:0.021543):0.001758):0.001747,AB246773_Potyviridae_Potyvirus:0.025048):0.000504,(((JN190431_Potyvirid

ae_Potyvirus:0.018898,AM039800_Potyviridae_Potyvirus:0.018898):0.00130
8,(AY112735_Potyviridae_Potyvirus:0.004819,AJ312437_Potyviridae_Potyvi
rus:0.004819)94:0.015387):0.002268,U34972_Potyviridae_Potyvirus:0.0224
73):0.003079):0.001335,(KX505964_Potyviridae_Potyvirus:0.012572,AJ2980
33_Potyviridae_Potyvirus:0.012572)86:0.014316):0.004345,((AJ307036_Pot
yviridae_Potyvirus:0.01897,KU981084_Potyviridae_Potyvirus:0.01897):0.0
06209,AF348210_Potyviridae_Potyvirus:0.025179):0.006054):0.000919,AY62
6825_Potyviridae_Potyvirus:0.032151):0.00912,(((DQ851494_Potyviridae_P
otyvirus:0.010151,DQ821938_Potyviridae_Potyvirus:0.010151)82:0.010314,
AY206394_Potyviridae_Potyvirus:0.020465):0.012379,AF023848_Potyviridae
_Potyvirus:0.032844):0.008427):0.005244,(((((AJ307057_Potyviridae_Poty
virus:0.029047,JF838187_Potyviridae_Potyvirus:0.029047):0.005889,(AJ31
0197_Potyviridae_Potyvirus:0.027622,AJ001691_Potyviridae_Potyvirus:0.0
27622):0.007314):0.004798,((M11458_Potyviridae_Potyvirus:0.027834,D109
30_Potyviridae_Potyvirus:0.027834):0.007232,KP343681_Potyviridae_Potyv
irus:0.035066):0.004668):0.001702,EF427894_Potyviridae_Potyvirus:0.041
435):0.001892,(((((U47033_Potyviridae_Potyvirus:0.011015,D83749_Potyvi
ridae_Potyvirus:0.011015)74:0.007983,DQ641248_Potyviridae_Potyvirus:0.
018999):0.001509,AB011819_Potyviridae_Potyvirus:0.020507)73:0.012604,(
KM523548_Potyviridae_Potyvirus:0.029385,JQ801448_Potyviridae_Potyvirus
:0.029385):0.003727):0.003951,(EU847625_Potyviridae_Potyvirus:0.031191
,AB219545_Potyviridae_Potyvirus:0.031191):0.005871):0.006264):0.003188
):0.003543):0.004184,(AM181350_Potyviridae_Potyvirus:0.037784,DQ925486
_Potyviridae_Potyvirus:0.037784):0.016458):0.002073,(((((((GU207957_Po
tyviridae_Potyvirus:0.010404,AB509453_Potyviridae_Potyvirus:0.010404)9
1:0.008791,D86371_Potyviridae_Potyvirus:0.019195):0.003994,JN613807_Po
tyviridae_Potyvirus:0.023189):0.008327,KC443039_Potyviridae_Potyvirus:
0.031516):0.005736,(JQ824374_Potyviridae_Potyvirus:0.003585,JN613805_P
otyviridae_Potyvirus:0.003585)100:0.033668):0.006366,(((((D13751_Potyv
iridae_Potyvirus:0.006827,AJ243957_Potyviridae_Potyvirus:0.006827):0.0
01555,X16415_Potyviridae_Potyvirus:0.008382):0.003329,AM157175_Potyvir
idae_Potyvirus:0.011711):0.002447,JQ794501_Potyviridae_Potyvirus:0.014
158):0.007697,(KJ787006_Potyviridae_Potyvirus:0.014589,AY912055_Potyvi
ridae_Potyvirus:0.014589):0.007266)94:0.021763):0.006024,((((((X97705_
Potyviridae_Potyvirus:0.003029,X97704_Potyviridae_Potyvirus:0.003029)1
00:0.023945,KF906523_Potyviridae_Potyvirus:0.026975):0.010885,(JQ35073
8_Potyviridae_Potyvirus:0.035052,JX156434_Potyviridae_Potyvirus:0.0350
52):0.002807):0.001944,(HQ676607_Potyviridae_Potyvirus:0.026877,HM3635
16_Potyviridae_Potyvirus:0.026877):0.012927):0.001384,(GU181199_Potyvi
ridae_Potyvirus:0.036823,AM158908_Potyviridae_Potyvirus:0.036823):0.00
4364):0.001788,(GQ916624_Potyviridae_Potyvirus:0.033555,KX355613_Potyv
iridae_Potyvirus:0.033555):0.00942):0.006667):0.006672):0.00661,((((((
DQ851495_Potyviridae_Potyvirus:0.030929,DQ645484_Potyviridae_Potyvirus
:0.030929):0.002458,AB818538_Potyviridae_Potyvirus:0.033387):0.003939,
JN008909_Potyviridae_Potyvirus:0.037326):0.00102,EF219408_Potyviridae_
Potyvirus:0.038346):0.009514,(JQ807997_Potyviridae_Potyvirus:0.006477,
JQ807995_Potyviridae_Potyvirus:0.006477)100:0.041383):0.005083,((JX470
965_Potyviridae_Potyvirus:0.033881,AJ237843_Potyviridae_Potyvirus:0.03
3881):0.011309,DQ299908_Potyviridae_Potyvirus:0.04519):0.007753):0.009
983):0.005544,(((((((JQ395040_Potyviridae_Potyvirus:0.035568,DQ851496_
Potyviridae_Potyvirus:0.035568):0.003466,(Z26920_Potyviridae_Potyvirus
:0.021787,GQ421689_Potyviridae_Potyvirus:0.021787)83:0.017247):0.00295

3,(AJ865076_Potyviridae_Potyvirus:0.029377,AJ510223_Potyviridae_Potyvi
rus:0.029377):0.01261):0.005984,KT692938_Potyviridae_Potyvirus:0.04797
1):0.002788,(AM182028_Potyviridae_Potyvirus:0.027013,JQ723475_Potyviri
dae_Potyvirus:0.027013)88:0.023746):0.007732,AF499738_Potyviridae_Poty
virus:0.058491):0.006568,((((JQ692088_Potyviridae_Potyvirus:0.020332,A
Y642590_Potyviridae_Potyvirus:0.020332)72:0.007759,AJ297628_Potyvirida
e_Potyvirus:0.028091):0.011269,JX156422_Potyviridae_Potyvirus:0.03936)
:0.013434,(AJ851866_Potyviridae_Potyvirus:0.046359,KJ830760_Potyvirida
e_Potyvirus:0.046359):0.006435):0.012265):0.00341):0.03401,((AY623627_
Potyviridae_Rymovirus:0.028082,AY623626_Potyviridae_Rymovirus:0.028082
)83:0.010829,Y09854_Potyviridae_Rymovirus:0.038911)93:0.063569)97:0.06
212,U42596_Potyviridae_Potyvirus:0.164599)97:0.089425,AY994084_Potyvir
idae_Brambyvirus:0.254024):0.145865,((((((((HM181930_Potyviridae_Ipom
ovirus:0.0,FJ185044_Potyviridae_Ipomovirus:0.0)100:0.012421,FJ039520_P
otyviridae_Ipomovirus:0.012421)87:0.009723,(GU563327_Potyviridae_Ipomo
virus:0.015644,GQ329864_Potyviridae_Ipomovirus:0.015644):0.0065)94:0.0
13345,(AY578085_Potyviridae_Ipomovirus:0.02353,KU935732_Potyviridae_Ip
omovirus:0.02353)93:0.011959):0.013805,EU259611_Potyviridae_Ipomovirus
:0.049294)99:0.047708,((HQ840786_Potyviridae_Ipomovirus:0.016833,HE600
072_Potyviridae_Ipomovirus:0.016833)100:0.056085,Z73124_Potyviridae_Ip
omovirus:0.072918)78:0.024084)74:0.029983,JF280796_Potyviridae_Unassig
ned:0.126985)85:0.178797,(((KF984546_Potyviridae_Tritimovirus:0.031116
,KF260962_Potyviridae_Tritimovirus:0.031116):0.00532,(AF057533_Potyvir
idae_Tritimovirus:0.028033,AY377938_Potyviridae_Tritimovirus:0.028033)
:0.008403)77:0.019514,(EF608612_Potyviridae_Tritimovirus:0.044423,Z485
06_Potyviridae_Tritimovirus:0.044423):0.011527)100:0.249831):0.02599,(
(FJ263671_Potyviridae_Poacevirus:0.046254,GQ388116_Potyviridae_Poacevi
rus:0.046254)74:0.011757,JX156425_Potyviridae_Poacevirus:0.058011)97:0
.273761):0.068117)85:0.122402,(((((KU053507_Potyviridae_Macluravirus:0
.059228,KP405232_Potyviridae_Macluravirus:0.059228):0.012828,(KT724961
_Potyviridae_Macluravirus:0.025863,AB710145_Potyviridae_Macluravirus:0
.025863)99:0.046192)100:0.143877,(LC055681/LC060925_Potyviridae_Bymovi
rus:0.035222,Y10973/X90904_Potyviridae_Bymovirus:0.035222)100:0.180712
):0.025788,((D86634/D86635_Potyviridae_Bymovirus:0.05265,AJ132268/AJ13
2269_Potyviridae_Bymovirus:0.05265)92:0.104781,AJ306718/AJ306719_Potyv
iridae_Bymovirus:0.157432):0.084289):0.09641,X73883_Potyviridae_Bymovi
rus:0.338131)96:0.184159)100:0.346742)79:0.047867,((((((((JX857868_As
troviridae_Unassigned:0.032982,FJ973620_Astroviridae_Mamastrovirus:0.0
32982):0.00557,(JX857869_Astroviridae_Unassigned:0.030443,KF859964_Ast
roviridae_Mamastrovirus:0.030443):0.008109):0.008621,GQ415660_Astrovir
idae_Mamastrovirus:0.047173):0.016078,(((KM035759_Astroviridae_Unassig
ned:0.026091,Y15937_Astroviridae_Mamastrovirus:0.026091):0.00973,AY179
509_Astroviridae_Mamastrovirus:0.035821):0.007252,(JX556691_Astrovirid
ae_Mamastrovirus:0.038461,KM017741_Astroviridae_Mamastrovirus:0.038461
):0.004611):0.020179)100:0.19314,((((((KF787112_Astroviridae_Mamastrov
irus:0.044369,KF499111_Astroviridae_Mamastrovirus:0.044369):0.009874,Z
25771_Astroviridae_Mamastrovirus:0.054243):0.010105,KP404149_Astroviri
dae_Mamastrovirus:0.064348):0.046833,AB308374_Astroviridae_Mamastrovir
us:0.111181):0.095576,((JX857870_Astroviridae_Unassigned:0.019737,JF74
2759_Astroviridae_Unassigned:0.019737)78:0.011592,FJ222451_Astrovirida
e_Mamastrovirus:0.03133)100:0.175427):0.016286,JF729316_Astroviridae_M
amastrovirus:0.223042):0.033349):0.00711,((((((HQ916317_Astroviridae_M

amastrovirus:0.010763,HQ916313_Astroviridae_Mamastrovirus:0.010763):0.
00103,KJ620979_Astroviridae_Mamastrovirus:0.011793)99:0.018811,(HQ9163
16_Astroviridae_Mamastrovirus:0.013763,HQ916314_Astroviridae_Mamastrov
irus:0.013763)98:0.01684):0.005775,KR868724_Astroviridae_Mamastrovirus
:0.036378):0.011477,JF713710_Astroviridae_Mamastrovirus:0.047855)100:0
.050846,(((JX544743_Astroviridae_Unassigned:0.051927,KP663426_Astrovir
idae_Unassigned:0.051927):0.005228,JQ340310_Astroviridae_Mamastrovirus
:0.057155):0.006852,JF713713_Astroviridae_Mamastrovirus:0.064007)98:0.
034695)95:0.1648):0.077271,JX556693_Astroviridae_Unassigned:0.340773):
0.014725,JF755422_Astroviridae_Unassigned:0.355497)87:0.104859,(((AF20
6663_Astroviridae_Avastrovirus:0.069661,Y15936_Astroviridae_Avastrovir
us:0.069661):0.013617,FJ434664_Astroviridae_Avastrovirus:0.083278)99:0
.163321,AB033998_Astroviridae_Avastrovirus:0.246599)95:0.213757)100:0.
456543)85:0.036313,(((((((((((((JX236040_Flaviviridae_Flavivirus:0.003
787,AY632545_Flaviviridae_Flavivirus:0.003787):0.002773,(DQ211652_Flav
iviridae_Flavivirus:0.001641,M12294_Flaviviridae_Flavivirus:0.001641):
0.004919):0.000128,(((AY453411_Flaviviridae_Flavivirus:0.003313,M18370
_Flaviviridae_Flavivirus:0.003313):0.000457,JF895923_Flaviviridae_Flav
ivirus:0.00377):0.00055,AF161266_Flaviviridae_Flavivirus:0.00432):0.00
2369):0.007093,AY632544_Flaviviridae_Flavivirus:0.013781):0.010708,(((
KC692068_Flaviviridae_Unassigned:0.006992,AF326825_Flaviviridae_Flaviv
irus:0.006992):0.001568,JQ086551_Flaviviridae_Flavivirus:0.00856):0.00
5532,U88536_Flaviviridae_Flavivirus:0.014092):0.010397):0.00659,DQ8376
42_Flaviviridae_Flavivirus:0.031078):0.00737,JQ308185_Flaviviridae_Fla
vivirus:0.038448):0.006724,(U87411_Flaviviridae_Flavivirus:0.002188,M1
9197_Flaviviridae_Flavivirus:0.002188)77:0.042984):0.002416,((((((DQ8
59066_Flaviviridae_Flavivirus:0.004332,DQ859056_Flaviviridae_Flaviviru
s:0.004332):0.004237,DQ859057_Flaviviridae_Flavivirus:0.008569):0.0011
38,(DQ859062_Flaviviridae_Flavivirus:0.000878,DQ859067_Flaviviridae_Fl
avivirus:0.000878)97:0.008829):0.013713,(KC692067_Flaviviridae_Unassig
ned:0.017121,AY632539_Flaviviridae_Flavivirus:0.017121):0.006299):0.00
0829,AY099336_Flaviviridae_Flavivirus:0.024249):0.003638,((DQ525916_Fl
aviviridae_Flavivirus:0.010626,KF917536_Flaviviridae_Flavivirus:0.0106
26):0.009324,AY632541_Flaviviridae_Flavivirus:0.01995):0.007937):0.007
709,(((EU707555_Flaviviridae_Flavivirus:0.018621,KC788512_Flaviviridae
_Flavivirus:0.018621):0.002668,DQ859064_Flaviviridae_Flavivirus:0.0212
89):0.005596,(AB114858_Flaviviridae_Flavivirus:0.017644,DQ859065_Flavi
viridae_Flavivirus:0.017644):0.009241):0.00871):0.011993):0.032626,(((
(((((DQ235149_Flaviviridae_Flavivirus:0.006202,AY863002_Flaviviridae_
Flavivirus:0.006202):0.002195,DQ235145_Flaviviridae_Flavivirus:0.00839
7):0.002053,((KF815939_Flaviviridae_Flavivirus:0.004397,DQ235144_Flavi
viridae_Flavivirus:0.004397):0.00052,KF815940_Flaviviridae_Flavivirus:
0.004917):0.005533):0.004941,(((AY323490_Flaviviridae_Flavivirus:0.001
735,AF331718_Flaviviridae_Flavivirus:0.001735)86:0.001494,Y07863_Flavi
viridae_Flavivirus:0.00323)86:0.006918,KP144332_Flaviviridae_Flaviviru
s:0.010147):0.005244):0.005229,DQ235146_Flaviviridae_Flavivirus:0.0206
2):0.008811,(((U27495_Flaviviridae_Flavivirus:0.003794,AY193805_Flaviv
iridae_Flavivirus:0.003794):0.001632,AF253419_Flaviviridae_Flavivirus:
0.005425):0.023557,(X03700_Flaviviridae_Flavivirus:0.019216,AY632540_F
laviviridae_Flavivirus:0.019216):0.009766):0.000449):0.005853,((((KF91
7541_Flaviviridae_Flavivirus:0.004717,DQ837641_Flaviviridae_Flavivirus
:0.004717)82:0.011089,KJ152564_Flaviviridae_Flavivirus:0.015806):0.006

166,KJ210048_Flaviviridae_Unassigned:0.021972):0.008734,DQ235150_Flavi
viridae_Flavivirus:0.030706):0.004578):0.004619,((AY632535_Flavivirida
e_Flavivirus:0.020811,AY632536_Flaviviridae_Flavivirus:0.020811):0.007
433,(L06436_Flaviviridae_Flavivirus:0.018557,DQ859060_Flaviviridae_Fla
vivirus:0.018557):0.009688):0.011659):0.029501,(((AJ299445_Flavivirida
e_Flavivirus:0.007477,AJ242984_Flaviviridae_Flavivirus:0.007477)84:0.0
07442,AF160193_Flaviviridae_Flavivirus:0.014919)74:0.03683,(AF144692_F
laviviridae_Flavivirus:0.009544,KJ469371_Flaviviridae_Flavivirus:0.009
544)87:0.042206):0.017655):0.010809)88:0.34655,((((((KT599442_Flavivir
idae_Unassigned:0.006656,KP688058_Flaviviridae_Flavivirus:0.006656)87:
0.012438,AB488408_Flaviviridae_Flavivirus:0.019095):0.00979,JQ957875_F
laviviridae_Unassigned:0.028885):0.008647,((AY149905_Flaviviridae_Flav
ivirus:0.01304,KJ741267_Flaviviridae_Flavivirus:0.01304):0.010505,(KT1
92549_Flaviviridae_Flavivirus:0.00421,JQ268258_Flaviviridae_Flavivirus
:0.00421)95:0.019335):0.013987):0.008774,((KC464457_Flaviviridae_Flavi
virus:0.012525,AB262759_Flaviviridae_Flavivirus:0.012525):0.008105,FJ6
44291_Flaviviridae_Flavivirus:0.02063):0.025675):0.009171,KX148546_Fla
viviridae_Unassigned:0.055476)88:0.371288)79:0.22616,AF285080_Flavivir
idae_Flavivirus:0.652924)95:0.23725,(((((((((AF179612_Flaviviridae_He
pacivirus:0.000318,U22304_Flaviviridae_Hepacivirus:0.000318)100:0.0427
57,KJ950938_Flaviviridae_Hepacivirus:0.043075):0.008243,KC815310_Flavi
viridae_Hepacivirus:0.051318):0.007097,(KC796077_Flaviviridae_Hepacivi
rus:0.047238,KJ950939_Flaviviridae_Hepacivirus:0.047238):0.011177):0.0
08323,(KP641127_Flaviviridae_Hepacivirus:0.003834,KP265943_Flavivirida
e_Hepacivirus:0.003834)100:0.062904):0.01247,((((KC796078_Flaviviridae
_Hepacivirus:0.000841,KC796090_Flaviviridae_Hepacivirus:0.000841)100:0
.057788,KC796074_Flaviviridae_Hepacivirus:0.058629):0.006437,((KP32540
1_Flaviviridae_Hepacivirus:0.01662,AB863589_Flaviviridae_Hepacivirus:0
.01662)89:0.029844,KC551802_Flaviviridae_Hepacivirus:0.046464):0.01860
2):0.007671,KC411784_Flaviviridae_Hepacivirus:0.072737):0.006472):0.02
4876,(KC411777_Flaviviridae_Hepacivirus:0.052925,KC411806_Flaviviridae
_Hepacivirus:0.052925):0.051159):0.015273,((((((D00944_Flaviviridae_He
pacivirus:0.013895,AF177036_Flaviviridae_Hepacivirus:0.013895):0.00633
8,EF108306_Flaviviridae_Hepacivirus:0.020233):0.010089,((M62321_Flaviv
iridae_Hepacivirus:0.002255,AF011751_Flaviviridae_Hepacivirus:0.002255
):0.001409,AF009606_Flaviviridae_Hepacivirus:0.003664)100:0.026658):0.
002696,((Y12083_Flaviviridae_Hepacivirus:0.010325,D84262_Flaviviridae_
Hepacivirus:0.010325)72:0.015284,GU814265_Flaviviridae_Hepacivirus:0.0
25609):0.007408):0.003637,Y13184_Flaviviridae_Hepacivirus:0.036655):0.
013537,(Y11604_Flaviviridae_Hepacivirus:0.036179,D17763_Flaviviridae_H
epacivirus:0.036179):0.014014):0.069165)84:0.34536,(((((KC796076_Flavi
viridae_Pegivirus:0.034708,KC796080_Flaviviridae_Pegivirus:0.034708):0
.008164,(KC815311_Flaviviridae_Pegivirus:0.024599,KJ950934_Flavivirida
e_Pegivirus:0.024599)86:0.018272):0.045675,(KT439329_Flaviviridae_Pegi
virus:0.011018,KT427414_Flaviviridae_Pegivirus:0.011018)98:0.07753):0.
013989,(((((KC796088_Flaviviridae_Pegivirus:0.024881,GU566734_Flavivir
idae_Pegivirus:0.024881):0.020679,KC410872_Flaviviridae_Pegivirus:0.04
556):0.008339,U94421_Flaviviridae_Pegivirus:0.053899):0.004691,KC14526
5_Flaviviridae_Pegivirus:0.05859):0.012188,KU351669_Flaviviridae_Pegiv
irus:0.070778):0.031758):0.106419,((((((((U63715_Flaviviridae_Pegiviru
s:0.008722,AY196904_Flaviviridae_Pegivirus:0.008722):0.001375,U45966_F
laviviridae_Pegivirus:0.010097):0.001204,(U44402_Flaviviridae_Pegiviru

s:0.008105,AB003289_Flaviviridae_Pegivirus:0.008105):0.003195):0.00112
2,AB003292_Flaviviridae_Pegivirus:0.012423):0.001502,AY949771_Flavivir
idae_Pegivirus:0.013925)85:0.013583,AB021287_Flaviviridae_Pegivirus:0.
027508)97:0.032278,D87713_Flaviviridae_Pegivirus:0.059786)96:0.050798,
(KF234501_Flaviviridae_Pegivirus:0.06463,AF070476_Flaviviridae_Pegivir
us:0.06463)70:0.045954):0.098372)84:0.255762)91:0.343636,((((((JX4289
45_Flaviviridae_Pestivirus:0.013774,AF326963_Flaviviridae_Pestivirus:0
.013774):0.005982,M31182_Flaviviridae_Pestivirus:0.019756):0.002052,AF
037405_Flaviviridae_Pestivirus:0.021808):0.017249,((AF144617_Flaviviri
dae_Pestivirus:0.01106,AF002227_Flaviviridae_Pestivirus:0.01106)72:0.0
10139,FJ040215_Flaviviridae_Pestivirus:0.0212):0.017857):0.031731,(AY7
81152_Flaviviridae_Pestivirus:0.048686,EF100713_Flaviviridae_Pestiviru
s:0.048686):0.022102):0.039229,KJ950914_Flaviviridae_Pestivirus:0.1100
16):0.077228,KU041639_Flaviviridae_Pestivirus:0.187244)100:0.621109)86
:0.081822):0.063038):0.029935):0.010885,((L29010_Hypoviridae_Hypovirus
:0.174788,M57938_Hypoviridae_Hypovirus:0.174788)98:0.728603,(AY307099_
Hypoviridae_Hypovirus:0.089493,AF188515_Hypoviridae_Hypovirus:0.089493
)95:0.813898)93:0.090641):0.00421,((((((((((((((((((((((KJ789130_Be
taflexiviridae_Carlavirus:0.025227,EU527979_Betaflexiviridae_Carlaviru
s:0.025227):0.001169,AB032469_Betaflexiviridae_Carlavirus:0.026396):0.
005439,(KX364696_Betaflexiviridae_Carlavirus:0.030396,KJ572564_Betafle
xiviridae_Carlavirus:0.030396):0.001439):0.003498,KX000914_Betaflexivi
ridae_Carlavirus:0.035333):0.003738,((((JN039374_Betaflexiviridae_Car
lavirus:0.02047,AJ516059_Betaflexiviridae_Carlavirus:0.02047):0.0063,F
J531634_Betaflexiviridae_Carlavirus:0.02677):0.004272,EU754720_Betafle
xiviridae_Carlavirus:0.031042):0.002027,EU074853_Betaflexiviridae_Carl
avirus:0.033069):0.001886,L25658_Betaflexiviridae_Carlavirus:0.034956)
:0.004115):0.003229,DQ455582_Betaflexiviridae_Carlavirus:0.042299):0.0
00896,(((AJ620300_Betaflexiviridae_Carlavirus:0.031013,AB051848_Betaf
lexiviridae_Carlavirus:0.031013):0.002289,AM158439_Betaflexiviridae_Ca
rlavirus:0.033302):0.000749,(EF492068_Betaflexiviridae_Carlavirus:0.02
5065,KJ415259_Betaflexiviridae_Carlavirus:0.025065):0.008986):0.003652
,AM493895_Betaflexiviridae_Carlavirus:0.037703):0.005493):0.001338,(KJ
572562_Betaflexiviridae_Carlavirus:0.040471,JQ245696_Betaflexiviridae_
Carlavirus:0.040471):0.004063):0.012782,AY505475_Betaflexiviridae_Carl
avirus:0.057315):0.002834,(((KJ572561_Betaflexiviridae_Carlavirus:0.0
18477,KJ572560_Betaflexiviridae_Carlavirus:0.018477)76:0.00631,KJ57256
3_Betaflexiviridae_Carlavirus:0.024787)97:0.026359,FJ196835_Betaflexiv
iridae_Carlavirus:0.051146):0.001736,(AJ292226_Betaflexiviridae_Carlav
irus:0.044702,AB517596_Betaflexiviridae_Carlavirus:0.044702):0.00818):
0.007268):0.01949,(((FJ685618_Betaflexiviridae_Carlavirus:0.045104,EF
527260_Betaflexiviridae_Carlavirus:0.045104):0.005583,AY461421_Betafle
xiviridae_Carlavirus:0.050687):0.014168,EU162589_Betaflexiviridae_Carl
avirus:0.064855):0.005209,(KP828803_Betaflexiviridae_Carlavirus:0.0204
3,KP784454_Betaflexiviridae_Carlavirus:0.02043)100:0.049633):0.009576)
:0.028445,((((((JQ904630_Betaflexiviridae_Carlavirus:0.007035,HM58481
9_Betaflexiviridae_Carlavirus:0.007035)100:0.029938,DQ098905_Betaflexi
viridae_Carlavirus:0.036973):0.000608,AJ863509_Betaflexiviridae_Carlav
irus:0.037581):0.006559,EU020009_Betaflexiviridae_Carlavirus:0.044139)
:0.000492,(EU433397_Betaflexiviridae_Carlavirus:0.039767,HQ184471_Beta
flexiviridae_Carlavirus:0.039767):0.004865):0.000919,D14449_Betaflexiv
iridae_Carlavirus:0.04555)88:0.032593,(((JQ395043_Betaflexiviridae_Car

lavirus:0.012136,AM182569_Betaflexiviridae_Carlavirus:0.012136)100:0.0
41509,JF320810_Betaflexiviridae_Carlavirus:0.053645):0.012679,JX212747
_Betaflexiviridae_Carlavirus:0.066325):0.011819)70:0.029942):0.023752,
((((((KF958838_Betaflexiviridae_Robigovirus:0.018411,AF237816_Betaflex
iviridae_Robigovirus:0.018411):0.000785,(KF030846_Betaflexiviridae_Rob
igovirus:0.015616,KC218926_Betaflexiviridae_Robigovirus:0.015616):0.00
358)86:0.013209,AF017780_Betaflexiviridae_Robigovirus:0.032404)100:0.0
58401,(JX277553_Betaflexiviridae_Foveavirus:0.052034,AF057136_Betaflex
iviridae_Foveavirus:0.052034)75:0.038772):0.026058,((KT893296_Betaflex
iviridae_Foveavirus:0.01225,FJ824737_Betaflexiviridae_Foveavirus:0.012
25):0.004778,KT893294_Betaflexiviridae_Foveavirus:0.017028)100:0.09983
6):0.012108,(((HQ339956_Betaflexiviridae_Foveavirus:0.018685,HE963831_
Betaflexiviridae_Foveavirus:0.018685):0.003061,D21829_Betaflexiviridae
_Foveavirus:0.021746)99:0.071991,EF693898_Betaflexiviridae_Foveavirus:
0.093736):0.035235):0.002865):0.03455,((AF314662_Betaflexiviridae_Unas
signed:0.060648,AY072921_Betaflexiviridae_Robigovirus:0.060648)77:0.03
2599,AF315308_Betaflexiviridae_Unassigned:0.093247)71:0.07314)100:0.18
2164,((((((AY713379_Betaflexiviridae_Trichovirus:0.032337,M58152_Beta
flexiviridae_Trichovirus:0.032337)93:0.026456,(DQ117579_Betaflexivirid
ae_Trichovirus:0.021004,AF170028_Betaflexiviridae_Trichovirus:0.021004
)97:0.037788)91:0.067303,(FR877530_Betaflexiviridae_Trichovirus:0.0325
18,D88448_Betaflexiviridae_Trichovirus:0.032518)100:0.093577)79:0.0357
65,(KF533710_Betaflexiviridae_Chordovirus:0.03306,KF533711_Betaflexivi
ridae_Chordovirus:0.03306)100:0.1288)76:0.05865,((KF700263_Betaflexivi
ridae_Tepovirus:0.025798,KF700262_Betaflexiviridae_Tepovirus:0.025798)
100:0.165103,EU835937_Betaflexiviridae_Tepovirus:0.190901):0.02961):0.
018572,((((X75433_Betaflexiviridae_Vitivirus:0.054776,JN427015_Betafle
xiviridae_Vitivirus:0.054776):0.017517,X75448_Betaflexiviridae_Vitivir
us:0.072293):0.01043,AB432910_Betaflexiviridae_Vitivirus:0.082723):0.0
08503,JX105428_Betaflexiviridae_Vitivirus:0.091226)100:0.147856):0.022
155,((KM507061_Betaflexiviridae_Prunevirus:0.140363,HG008921_Betaflexi
viridae_Prunevirus:0.140363)91:0.076333,AJ318061_Betaflexiviridae_Citr
ivirus:0.216696)72:0.044542):0.087314):0.0616,((HQ241409_Betaflexiviri
dae_Divavirus:0.017026,JX173276_Betaflexiviridae_Divavirus:0.017026):0
.002903,JX173277_Betaflexiviridae_Divavirus:0.019928)100:0.390223):0.0
10231,(KT763043_Betaflexiviridae_Capillovirus:0.091149,X82547_Betaflex
iviridae_Capillovirus:0.091149)99:0.329233):0.022161,((D14995_Betaflex
iviridae_Capillovirus:0.00555,AB004063_Betaflexiviridae_Capillovirus:0
.00555)100:0.038375,KU375548_Betaflexiviridae_Capillovirus:0.043925)10
0:0.398618):0.047437,((((((((((((((AJ633822_Alphaflexiviridae_Potex
virus:0.035828,AY707100_Alphaflexiviridae_Potexvirus:0.035828):0.00686
9,AY789138_Alphaflexiviridae_Potexvirus:0.042697):0.006978,AB353071_Al
phaflexiviridae_Potexvirus:0.049675):0.014056,D12517_Alphaflexiviridae
_Potexvirus:0.063732):0.003244,(((AB066288_Alphaflexiviridae_Potexviru
s:0.041662,U23414_Alphaflexiviridae_Potexvirus:0.041662):0.00418,AJ620
114_Alphaflexiviridae_Potexvirus:0.045842):0.01531,D00344_Alphaflexivi
ridae_Potexvirus:0.061152):0.005824):0.008269,Z21647_Alphaflexiviridae
_Potexvirus:0.075245):0.003465,((AJ316085_Alphaflexiviridae_Potexvirus
:0.035689,KR872420_Alphaflexiviridae_Potexvirus:0.035689):0.00865,D137
47_Alphaflexiviridae_Potexvirus:0.044338):0.034371):0.000467,((((S735
80_Alphaflexiviridae_Potexvirus:0.044807,U62963_Alphaflexiviridae_Pote
xvirus:0.044807):0.002064,X06728_Alphaflexiviridae_Potexvirus:0.046871

):0.006237,AF484251_Alphaflexiviridae_Potexvirus:0.053108):0.016229,(K
J711908_Alphaflexiviridae_Potexvirus:0.05649,KY288487_Alphaflexivirida
e_Potexvirus:0.05649):0.012848):0.00246,AB219105_Alphaflexiviridae_Pot
exvirus:0.071798):0.007379):0.019661,(JN389521_Alphaflexiviridae_Potex
virus:0.074383,FJ670570_Alphaflexiviridae_Potexvirus:0.074383):0.02445
5):0.029383,((((((AY366207_Alphaflexiviridae_Potexvirus:0.023718,JF93
0327_Alphaflexiviridae_Potexvirus:0.023718)81:0.007824,AY366208_Alphaf
lexiviridae_Potexvirus:0.031542)89:0.013665,AY366209_Alphaflexiviridae
_Potexvirus:0.045206):0.007181,AF308158_Alphaflexiviridae_Potexvirus:0
.052387)85:0.021422,((D13957_Alphaflexiviridae_Potexvirus:0.027497,AY8
63024_Alphaflexiviridae_Potexvirus:0.027497)75:0.011439,KX196173_Alpha
flexiviridae_Potexvirus:0.038936)97:0.034873):0.012408,(((AB304848_Alp
haflexiviridae_Potexvirus:0.027592,AB206396_Alphaflexiviridae_Potexvir
us:0.027592):0.006228,AM745758_Alphaflexiviridae_Potexvirus:0.03382)76
:0.015979,DQ660333_Alphaflexiviridae_Potexvirus:0.049799)94:0.036418):
0.028742,D29630_Alphaflexiviridae_Potexvirus:0.11496):0.013262):0.0579
59,((KP313240_Alphaflexiviridae_Mandarivirus:0.004555,JX040635_Alphafl
exiviridae_Mandarivirus:0.004555)100:0.015737,AF406744_Alphaflexivirid
ae_Mandarivirus:0.020293)100:0.165888):0.003535,EU489641_Alphaflexivir
idae_Lolavirus:0.189716):0.004634,((((AB010302_Alphaflexiviridae_Alle
xivirus:0.026994,KM379144_Alphaflexiviridae_Allexivirus:0.026994)83:0.
013999,U89243_Alphaflexiviridae_Allexivirus:0.040993):0.004069,((AJ292
230_Alphaflexiviridae_Allexivirus:0.035027,KF555653_Alphaflexiviridae_
Allexivirus:0.035027):0.005125,AB010300_Alphaflexiviridae_Allexivirus:
0.040152):0.00491)92:0.043899,(JN053266_Alphaflexiviridae_Unassigned:0
.030616,KX058345_Alphaflexiviridae_Unassigned:0.030616)98:0.058345)74:
0.034379,M97264_Alphaflexiviridae_Allexivirus:0.12334)75:0.07101):0.01
11,(M62730_Alphaflexiviridae_Potexvirus:0.05649,D26017_Alphaflexivirid
ae_Potexvirus:0.05649)100:0.14896):0.019043,AY055762_Alphaflexiviridae
_Botrexvirus:0.224493):0.039386,KC923234_Alphaflexiviridae_Platypuviru
s:0.263879)70:0.056286,AY147260_Alphaflexiviridae_Sclerodarnavirus:0.3
20165)70:0.169815):0.097933,AF238884_Gammaflexiviridae_Mycoflexivirus:
0.587914):0.039877,((((((((((J04375_Tymoviridae_Tymovirus:0.048015,AY
751780_Tymoviridae_Tymovirus:0.048015):0.006465,(AY751777_Tymoviridae_
Tymovirus:0.027179,AY751779_Tymoviridae_Tymovirus:0.027179)99:0.027301
)85:0.085784,(AY751778_Tymoviridae_Tymovirus:0.029816,EU684141_Tymovir
idae_Tymovirus:0.029816)100:0.110449):0.042568,(((KC840043_Tymovirida
e_Tymovirus:0.040556,JX508291_Tymoviridae_Tymovirus:0.040556)95:0.0310
88,J04374_Tymoviridae_Tymovirus:0.071645)77:0.084662,JX508290_Tymoviri
dae_Tymovirus:0.156306):0.019739,FN563123_Tymoviridae_Tymovirus:0.1760
45):0.006786):0.011576,HQ425778_Tymoviridae_Tymovirus:0.194408):0.0035
31,AY789137_Tymoviridae_Tymovirus:0.197939):0.000965,AF195000_Tymoviri
dae_Tymovirus:0.198904):0.011007,AF098523_Tymoviridae_Tymovirus:0.2099
11):0.001206,((X07441_Tymoviridae_Tymovirus:0.081737,EF554577_Tymoviri
dae_Tymovirus:0.081737)70:0.117463,D00637_Tymoviridae_Tymovirus:0.1992
):0.011917):0.01413,Y16104_Tymoviridae_Tymovirus:0.225247):0.063002,((
((((((AY884005_Tymoviridae_Marafivirus:0.073904,FJ915122_Tymoviridae_M
arafivirus:0.073904):0.002264,(U87832_Tymoviridae_Marafivirus:0.058074
,KT273411_Tymoviridae_Marafivirus:0.058074):0.018094)75:0.064216,FJ436
028_Tymoviridae_Marafivirus:0.140384):0.009229,AJ271595_Tymoviridae_Un
assigned:0.149613):0.009625,(JF727261_Tymoviridae_Marafivirus:0.09368,
AF265566_Tymoviridae_Marafivirus:0.09368)70:0.065558):0.016839,KX35420

2_Tymoviridae_Marafivirus:0.176077):0.015092,(((((KT162926_Tymoviridae
_Unassigned:0.040129,KT162924_Tymoviridae_Unassigned:0.040129)84:0.028
103,AB624361_Tymoviridae_Maculavirus:0.068233):0.03436,(KX171166_Tymov
iridae_Maculavirus:0.070481,AJ309022_Tymoviridae_Maculavirus:0.070481)
:0.032112):0.034061,JQ429443_Tymoviridae_Maculavirus:0.136653):0.01726
6,FJ444852_Tymoviridae_Marafivirus:0.15392):0.03725):0.051034,FM200426
_Tymoviridae_Maculavirus:0.242204):0.046045)78:0.339542)93:0.114367,(K
T581451_Deltaflexiviridae_Deltaflexivirus:0.269198,KX015962_Deltaflexi
viridae_Deltaflexivirus:0.269198)96:0.47296)100:0.143287,(((EU099844/E
U099845_Benyviridae_Benyvirus:0.255734,AB818898/AB818899_Benyviridae_B
enyvirus:0.255734):0.039685,(AF280539/AF061869/AF280540/..._Benyvirida
e_Benyvirus:0.10768,D84410/D84411/D84412/..._Benyviridae_Benyvirus:0.1
0768)95:0.187738)100:0.587122,((((((JQ001749_Hepeviridae_Orthohepeviru
s:0.078115,AY535004_Hepeviridae_Orthohepevirus:0.078115)88:0.057884,GU
345042_Hepeviridae_Orthohepevirus:0.135999)84:0.059704,L08816_Hepeviri
dae_Orthohepevirus:0.195703)93:0.156767,HQ731075_Hepeviridae_Piscihepe
virus:0.35247)100:0.420896,((U18246/L37299_Alphatetraviridae_Omegatetr
avirus:0.059238,AY594352/AY594353_Alphatetraviridae_Omegatetravirus:0.
059238)100:0.577367,AF102884_Alphatetraviridae_Betatetravirus:0.636605
)95:0.13676):0.053996,((((((((((((((((KF477193_Virgaviridae_Tobamovi
rus:0.009163,KT383474_Virgaviridae_Tobamovirus:0.009163):3.2e-
05,AF332868_Virgaviridae_Tobamovirus:0.009195)79:0.003923,EF375551_Vir
gaviridae_Tobamovirus:0.013118):0.004371,DQ355023_Virgaviridae_Tobamov
irus:0.017489):0.001999,V01408_Virgaviridae_Tobamovirus:0.019488):0.00
7766,(KU659022_Virgaviridae_Tobamovirus:0.020764,M81413_Virgaviridae_T
obamovirus:0.020764):0.006489):0.00459,(((AB089381_Virgaviridae_Tobamo
virus:0.015391,D13438_Virgaviridae_Tobamovirus:0.015391):0.005964,KF49
5564_Virgaviridae_Tobamovirus:0.021355):0.000124,AM398436_Virgaviridae
_Tobamovirus:0.021479)81:0.010365):0.004758,(((U03387_Virgaviridae_Tob
amovirus:0.006835,HQ667979_Virgaviridae_Tobamovirus:0.006835)100:0.009
566,AB017503_Virgaviridae_Tobamovirus:0.016401):0.004789,U30944_Virgav
iridae_Tobamovirus:0.021189)84:0.015412):0.005761,M34077_Virgaviridae_
Tobamovirus:0.042362):0.003808,AM040955_Virgaviridae_Tobamovirus:0.046
171)70:0.01489,X82130_Virgaviridae_Tobamovirus:0.06106):0.001313,((((A
J295949_Virgaviridae_Tobamovirus:0.012314,AJ295948_Virgaviridae_Tobamo
virus:0.012314)96:0.012462,AF321057_Virgaviridae_Tobamovirus:0.024776)
82:0.008934,(AB261167_Virgaviridae_Tobamovirus:0.03032,D12505_Virgavir
idae_Tobamovirus:0.03032):0.00339)82:0.011279,(KJ395757_Virgaviridae_T
obamovirus:0.028918,HM026454_Virgaviridae_Tobamovirus:0.028918)89:0.01
6071)82:0.017384):0.017896,(((HQ389540_Virgaviridae_Tobamovirus:0.0313
76,DQ356949_Virgaviridae_Tobamovirus:0.031376)99:0.02677,JN566124_Virg
aviridae_Tobamovirus:0.058147):0.004421,(AF395898_Virgaviridae_Tobamov
irus:0.028657,AB917427_Virgaviridae_Tobamovirus:0.028657)95:0.033911):
0.017701):0.013412,(JF729471_Virgaviridae_Tobamovirus:0.042191,EU04333
5_Virgaviridae_Tobamovirus:0.042191)95:0.051491)100:0.310775,((((((AJ
238607/AJ243719/AJ277556_Virgaviridae_Pomovirus:0.039214,KT225271/KT22
5272/KT225273_Virgaviridae_Pomovirus:0.039214)96:0.043789,Z97873/U6451
2/Z66493_Virgaviridae_Pomovirus:0.083003)72:0.020271,AJ223596/AJ223597
/AJ223598_Virgaviridae_Pomovirus:0.103274)89:0.047464,D86636/D86637/D8
6638_Virgaviridae_Pomovirus:0.150738)84:0.089585,((((L07937/L07938_Vir
gaviridae_Furovirus:0.017594,AJ012005/AJ012006_Virgaviridae_Furovirus:
0.017594)97:0.008218,AJ132576/AJ132577_Virgaviridae_Furovirus:0.025811

)89:0.013437,AJ132578/AJ132579_Virgaviridae_Furovirus:0.039248)93:0.02
6191,AB033691/AB033692_Virgaviridae_Furovirus:0.065439)100:0.174884):0
.037357,(((X78602/L07269_Virgaviridae_Pecluvirus:0.065748,X99149/AF447
397_Virgaviridae_Pecluvirus:0.065748)100:0.098141,AB976029/AB976030_Vi
rgaviridae_Goravirus:0.163889):0.052528,J04342/X03854/M16576_Virgaviri
dae_Hordeivirus:0.216417):0.061263):0.046843,((AF166084/Z36974_Virgavi
ridae_Tobravirus:0.036636,L23972/X03241_Virgaviridae_Tobravirus:0.0366
36):0.006612,X14006/X51828_Virgaviridae_Tobravirus:0.043248)100:0.2812
75)71:0.079933)90:0.070649,((((((((DQ318818/AY743591/AY363228_Bromovi
ridae_Ilarvirus:0.017276,DQ091193/DQ091194/DQ091195_Bromoviridae_Ilarv
irus:0.017276)82:0.013193,AY496068/AY496069/U35145_Bromoviridae_Ilarvi
rus:0.030469):0.003122,KT290039/KT290040/KT290041_Bromoviridae_Ilarvir
us:0.033592):0.009947,JX463340/JX463341/JX463342_Bromoviridae_Ilarviru
s:0.043539)89:0.030515,U80934/U75538/X00435_Bromoviridae_Ilarvirus:0.0
74054)99:0.099944,((((U57047/U34050/U85399_Bromoviridae_Ilarvirus:0.02
1407,EU919666/EU919667/X86352_Bromoviridae_Ilarvirus:0.021407)82:0.011
97,U93192/U93193/U93194_Bromoviridae_Ilarvirus:0.033377):0.006489,AF22
6160/AF226161/AF226162_Bromoviridae_Ilarvirus:0.039866)75:0.019086,(EF
584664/EF584665/U17389_Bromoviridae_Ilarvirus:0.032039,U23715/U17726/U
17390_Bromoviridae_Ilarvirus:0.032039)78:0.026913)99:0.115046):0.01749
3,(((((HE572565/FN669168/FN669169_Bromoviridae_Ilarvirus:0.032685,KF03
1037/KF031038/KF031039_Bromoviridae_Ilarvirus:0.032685):0.006579,(JN41
6771/JN416772/JN416773_Bromoviridae_Ilarvirus:0.003511,AF278534/AF2785
35/U57046_Bromoviridae_Ilarvirus:0.003511)100:0.035753)91:0.020424,AF1
74584/AF174585/U15608_Bromoviridae_Ilarvirus:0.059689):0.017626,((U576
48/AF277662/L28145_Bromoviridae_Ilarvirus:0.043668,AY682102/AY707771/A
Y707772_Bromoviridae_Ilarvirus:0.043668)73:0.024458,L00163/X01572/K027
03_Bromoviridae_Alfamovirus:0.068126):0.009188)87:0.081607,AY500236/AY
500237/AY500238_Bromoviridae_Ilarvirus:0.158922):0.032569):0.028217,AF
235033/AF235165/AF235166_Bromoviridae_Ilarvirus:0.219708)100:0.163355,
(((((((AB080598/AB080599/AB080600_Bromoviridae_Bromovirus:0.035521,M6
5139/M28817/M28818_Bromoviridae_Bromovirus:0.035521):0.002315,(X58456/
X58457/X58458_Bromoviridae_Bromovirus:0.005634,X02380/X01678/J02042_Br
omoviridae_Bromovirus:0.005634)100:0.032201):0.004795,AB444583/AB44458
4/AB444585_Bromoviridae_Bromovirus:0.042631):0.004325,AB194806/AB19480
7/AB194808_Bromoviridae_Bromovirus:0.046956)99:0.042647,M65138/M64713/
M60291_Bromoviridae_Bromovirus:0.089603)92:0.05028,(((D10044/D10663/AJ
277268_Bromoviridae_Cucumovirus:0.031857,D00356/D00355/D10538_Bromovir
idae_Cucumovirus:0.031857):0.007898,U15728/U15729/U15730_Bromoviridae_
Cucumovirus:0.039754)75:0.014999,FM881899/FM881900/FM881901_Bromovirid
ae_Cucumovirus:0.054754)100:0.085128)97:0.088289,((KF742519/KF742521/K
F742520_Bromoviridae_Anulavirus:0.037891,AB724113/AB724115/AB724114_Br
omoviridae_Anulavirus:0.037891)100:0.061475,AJ272327/AJ272329/AJ272328
_Bromoviridae_Anulavirus:0.099366)100:0.128805)93:0.104955,X94346/X943
47/X76993/..._Bromoviridae_Oleavirus:0.333127)80:0.049936)91:0.092043)
100:0.104154,(((((((((KR002686/KR002687_Closteroviridae_Crinivirus:0
.036427,AY242077/AY242078_Closteroviridae_Crinivirus:0.036427):0.00292
3,AB523788/AB523789_Closteroviridae_Crinivirus:0.03935):0.006655,EU191
904/EU191905_Closteroviridae_Crinivirus:0.046005):0.005884,FJ380118/FJ
380119_Closteroviridae_Crinivirus:0.051889)96:0.069354,((GQ376201/GQ22
5585_Closteroviridae_Crinivirus:0.045474,AY776335/AY776334_Closterovir
idae_Crinivirus:0.045474)99:0.028269,(AY488137/AY488138_Closterovirida

e_Crinivirus:0.030124,AY330918/AY330919_Closteroviridae_Crinivirus:0.0
30124)100:0.043618):0.0475):0.005546,AJ557128/AJ557129/AJ508757_Closte
roviridae_Crinivirus:0.126789):0.002864,(AY903447/AY903448_Closterovir
idae_Crinivirus:0.126842,AJ428554/AJ428555_Closteroviridae_Crinivirus:
0.126842):0.002811):0.047951,(FJ815440/FJ815441_Closteroviridae_Criniv
irus:0.160553,U15440/U15441_Closteroviridae_Crinivirus:0.160553):0.017
051)99:0.176445,(((HE588185_Closteroviridae_Velarivirus:0.189724,HM588
723_Closteroviridae_Velarivirus:0.189724)79:0.111018,Y10237_Closterovi
ridae_Velarivirus:0.300742):0.021064,KR349464_Closteroviridae_Velarivi
rus:0.321806):0.032243)98:0.142412,(((((FJ467504_Closteroviridae_Ampe
lovirus:0.027228,FJ467503_Closteroviridae_Ampelovirus:0.027228):0.0045
53,FR822696_Closteroviridae_Ampelovirus:0.031781):0.009433,AM182328_Cl
osteroviridae_Ampelovirus:0.041214)100:0.210866,AF414119_Closterovirid
ae_Ampelovirus:0.25208):0.047985,EF546442_Closteroviridae_Ampelovirus:
0.300065)76:0.105399,(((AF037268_Closteroviridae_Ampelovirus:0.103216
,KC904540_Closteroviridae_Ampelovirus:0.103216)100:0.193504,LC052212_C
losteroviridae_Ampelovirus:0.29672):0.015121,JQ023131_Closteroviridae_
Ampelovirus:0.31184):0.047806,AF531505_Closteroviridae_Ampelovirus:0.3
59646):0.045817)87:0.090998):0.016502,((((((KT203917_Closteroviridae_
Closterovirus:0.026896,AY792620_Closteroviridae_Closterovirus:0.026896
)100:0.05184,FJ869862_Closteroviridae_Closterovirus:0.078737)92:0.1527
62,(GU234167_Closteroviridae_Unassigned:0.035706,X73476_Closterovirida
e_Closterovirus:0.035706)100:0.195792):0.005174,(AF314061_Closteroviri
dae_Closterovirus:0.013271,AY881628_Closteroviridae_Closterovirus:0.01
3271)100:0.223401):0.019976,((DQ860839_Closteroviridae_Closterovirus:0
.081777,KJ748003_Closteroviridae_Closterovirus:0.081777):0.020368,DQ35
7218_Closteroviridae_Closterovirus:0.102146)81:0.154502):0.021426,U163
04_Closteroviridae_Closterovirus:0.278075)82:0.145704,(AB923924_Closte
roviridae_Unassigned:0.405949,AB733585_Closteroviridae_Unassigned:0.40
5949):0.01783)82:0.089184)97:0.066296)100:0.184708,(((((((L04653_Toga
viridae_Alphavirus:0.025791,AF075256_Togaviridae_Alphavirus:0.025791):
0.00682,(AF075254_Togaviridae_Alphavirus:0.010083,AF075253_Togaviridae
_Alphavirus:0.010083)95:0.022529):0.001838,((AF075251_Togaviridae_Alph
avirus:0.019685,AF075259_Togaviridae_Alphavirus:0.019685):0.008451,AF0
75258_Togaviridae_Alphavirus:0.028135):0.006314):0.020408,(((AF214040_
Togaviridae_Alphavirus:0.020779,FJ827631_Togaviridae_Alphavirus:0.0207
79):0.00964,GQ281603_Togaviridae_Alphavirus:0.030419):0.014305,(KJ4696
40_Togaviridae_Alphavirus:0.026203,X63135_Togaviridae_Alphavirus:0.026
203)87:0.018521):0.010133):0.007787,((((M20303_Togaviridae_Alphavirus:
0.02295,AF369024_Togaviridae_Alphavirus:0.02295)80:0.012007,(M20162_To
gaviridae_Alphavirus:0.021336,AY702913_Togaviridae_Alphavirus:0.021336
)79:0.013621):0.007079,((X04129_Togaviridae_Alphavirus:0.029847,KM1155
30_Togaviridae_Alphavirus:0.029847):0.004642,(AF237947_Togaviridae_Alp
havirus:0.029135,HM147985_Togaviridae_Alphavirus:0.029135):0.005355):0
.007547):0.006076,((HM147993_Togaviridae_Alphavirus:0.038236,J02363_To
gaviridae_Alphavirus:0.038236):0.007664,HM147989_Togaviridae_Alphaviru
s:0.045901):0.002211):0.014532):0.009029,((HM147990_Togaviridae_Alphav
irus:0.053494,U73745_Togaviridae_Alphavirus:0.053494):0.005833,AF12628
4_Togaviridae_Alphavirus:0.059327):0.012346):0.024938,(KY303625_Togavi
ridae_Alphavirus:0.046019,JX678730_Togaviridae_Alphavirus:0.046019)89:
0.050592)93:0.05314,AJ316244_Togaviridae_Alphavirus:0.14975)100:0.6142
17)89:0.063394):0.055179):0.002905):0.029131,M15240_Rubiviridae_Rubivi

rus:0.914577):0.069374,((((((AF180391_Arteriviridae_Simartevirus:0.05
9219,KP126831_Arteriviridae_Simartevirus:0.059219)82:0.048499,KR139839
_Arteriviridae_Simartevirus:0.107718):0.038095,((KM110946_Arterivirida
e_Unassigned:0.007877,KM110938_Arteriviridae_Simartevirus:0.007877)100
:0.064749,KT447550_Arteriviridae_Simartevirus:0.072625)87:0.073187):0.
046533,(KM677927_Arteriviridae_Simartevirus:0.024612,KR862307_Arterivi
ridae_Simartevirus:0.024612)100:0.167734)100:0.160391,((((U87392_Arter
iviridae_Porartevirus:0.005391,AF046869_Arteriviridae_Porartevirus:0.0
05391)100:0.091068,U15146_Arteriviridae_Porartevirus:0.096459)80:0.040
326,KP280006_Arteriviridae_Porartevirus:0.136784)85:0.065712,KP026921_
Arteriviridae_Nesartevirus:0.202496)99:0.150241)87:0.114474,X53459_Art
eriviridae_Equartevirus:0.46721)100:0.474865,(((((((((((EU420139_Cor
onaviridae_Alphacoronavirus:0.014585,EU420138_Coronaviridae_Alphacoron
avirus:0.014585):0.003761,AF304460_Coronaviridae_Alphacoronavirus:0.01
8347):0.002882,((KY073744_Coronaviridae_Alphacoronavirus:0.01185,KF430
219_Coronaviridae_Alphacoronavirus:0.01185):0.002064,KT368907_Coronavi
ridae_Alphacoronavirus:0.013914):0.007315):0.009731,(((AY567487_Corona
viridae_Alphacoronavirus:0.018654,KJ473806_Coronaviridae_Alphacoronavi
rus:0.018654):0.00367,(DQ648858_Coronaviridae_Alphacoronavirus:0.01368
8,AF353511_Coronaviridae_Alphacoronavirus:0.013688):0.008636):0.003033
,KJ473809_Coronaviridae_Alphacoronavirus:0.025357):0.005603):0.004699,
((JQ989271_Coronaviridae_Alphacoronavirus:0.001341,JQ989270_Coronaviri
dae_Alphacoronavirus:0.001341)100:0.014368,KJ473807_Coronaviridae_Alph
acoronavirus:0.015708):0.01995):0.012842,(((AY994055_Coronaviridae_Alp
hacoronavirus:0.015207,KP849472_Coronaviridae_Alphacoronavirus:0.01520
7):0.000655,(HM245925_Coronaviridae_Alphacoronavirus:0.009397,KM347965
_Coronaviridae_Alphacoronavirus:0.009397)78:0.006465):0.012133,KR06145
9_Coronaviridae_Alphacoronavirus:0.027995)91:0.020505):0.020313,(EF203
064_Coronaviridae_Alphacoronavirus:0.007336,KJ473808_Coronaviridae_Alp
hacoronavirus:0.007336)99:0.061477)95:0.157407,((EU095850_Coronavirida
e_Gammacoronavirus:0.023477,M95169_Coronaviridae_Gammacoronavirus:0.02
3477)100:0.080117,EU111742_Coronaviridae_Gammacoronavirus:0.103595)93:
0.122625):0.052242,(((((KC545386_Coronaviridae_Betacoronavirus:0.00467
1,KC545383_Coronaviridae_Betacoronavirus:0.004671)100:0.026218,(JX8690
59_Coronaviridae_Betacoronavirus:0.000516,KC164505_Coronaviridae_Betac
oronavirus:0.000516)100:0.030373):0.010367,(EF065505_Coronaviridae_Bet
acoronavirus:0.01483,EF065509_Coronaviridae_Betacoronavirus:0.01483)93
:0.026426)100:0.069245,(((KU762338_Coronaviridae_Unassigned:0.019907,E
F065513_Coronaviridae_Betacoronavirus:0.019907)97:0.031831,(GU190215_C
oronaviridae_Unassigned:0.015512,AY274119_Coronaviridae_Betacoronaviru
s:0.015512)96:0.036226):0.00829,KF636752_Coronaviridae_Betacoronavirus
:0.060028)87:0.050474):0.014334,((((JN874559_Coronaviridae_Betacoronav
irus:0.02204,AF391541_Coronaviridae_Betacoronavirus:0.02204):0.003729,
(FJ938068_Coronaviridae_Betacoronavirus:0.011432,AF029248_Coronavirida
e_Betacoronavirus:0.011432)87:0.014338):0.010093,KM349742_Coronavirida
e_Betacoronavirus:0.035862):0.009629,AY597011_Coronaviridae_Betacorona
virus:0.045491)100:0.079344)96:0.153627):0.055761,((((((JQ065043_Coron
aviridae_Deltacoronavirus:0.001045,JQ065042_Coronaviridae_Deltacoronav
irus:0.001045)100:0.020714,JQ065046_Coronaviridae_Deltacoronavirus:0.0
21759):0.001993,JQ065045_Coronaviridae_Deltacoronavirus:0.023752):0.00
6891,((JQ065044_Coronaviridae_Deltacoronavirus:0.016135,FJ376622_Coron
aviridae_Deltacoronavirus:0.016135):0.004609,(FJ376621_Coronaviridae_D

eltacoronavirus:0.012542,FJ376619_Coronaviridae_Deltacoronavirus:0.012
542)77:0.008202):0.0099):0.007374,JQ065049_Coronaviridae_Deltacoronavi
rus:0.038018):0.019958,(JQ065048_Coronaviridae_Deltacoronavirus:0.0249
68,JQ065047_Coronaviridae_Deltacoronavirus:0.024968)79:0.033008)100:0.
276247)100:0.515359,((((DQ898157_Coronaviridae_Bafinivirus:0.104607,GU
002364_Coronaviridae_Bafinivirus:0.104607)99:0.345262,KJ681496_Coronav
iridae_Unassigned:0.44987)98:0.238841,(JQ860350_Coronaviridae_Toroviru
s:0.017359,AY427798_Coronaviridae_Torovirus:0.017359)100:0.671352):0.0
14196,(KM589359_Coronaviridae_Unassigned:0.64758,KJ541759_Coronavirida
e_Unassigned:0.64758):0.055326)100:0.146676)93:0.050143,((((JQ957872_M
esoniviridae_Alphamesonivirus:0.028052,KJ125489_Mesoniviridae_Alphames
onivirus:0.028052):0.007314,(HM746600_Mesoniviridae_Alphamesonivirus:0
.021752,DQ458789_Mesoniviridae_Alphamesonivirus:0.021752)71:0.013614):
0.014581,(AB753015_Mesoniviridae_Alphamesonivirus:0.014445,KC807171_Me
soniviridae_Alphamesonivirus:0.014445)91:0.035502):0.019559,(JQ957873_
Mesoniviridae_Unassigned:0.042564,JQ957874_Mesoniviridae_Unassigned:0.
042564):0.026942)100:0.83022)74:0.021567,AF227196_Roniviridae_Okavirus
:0.921293)95:0.020782)99:0.041876)71:0.014292):0.001207)85:0.000551,((
(((((((((((LC150565_Narnaviridae_Mitovirus:0.018856,LC006129_Narnavi
ridae_Mitovirus:0.018856)93:0.024758,AJ132754_Narnaviridae_Mitovirus:0
.043614):0.004083,(AY563138_Narnaviridae_Mitovirus:0.030612,LC006128_N
arnaviridae_Mitovirus:0.030612)71:0.017084):0.006307,(AJ132755_Narnavi
ridae_Mitovirus:0.029993,LN827941_Narnaviridae_Mitovirus:0.029993)82:0
.024011):0.012878,((KC602001_Narnaviridae_Mitovirus:0.041619,LN827947_
Narnaviridae_Mitovirus:0.041619):0.013465,AJ132756_Narnaviridae_Mitovi
rus:0.055083):0.011799):0.015309,((AY615209_Narnaviridae_Mitovirus:0.0
56629,LC150564_Narnaviridae_Mitovirus:0.056629):0.019375,LC145036_Narn
aviridae_Mitovirus:0.076004):0.006187):0.024427,KJ463570_Narnaviridae_
Mitovirus:0.106619)81:0.246327,(KT921182_Narnaviridae_Mitovirus:0.0893
07,KT921181_Narnaviridae_Mitovirus:0.089307)81:0.263639):0.013123,(KT9
21183_Narnaviridae_Mitovirus:0.052591,KT921179_Narnaviridae_Mitovirus:
0.052591)93:0.313478):0.053577,(((((((LN827949_Narnaviridae_Mitovirus
:0.023146,AJ004930_Narnaviridae_Mitovirus:0.023146)100:0.031702,EF5801
00_Narnaviridae_Mitovirus:0.054848)70:0.016752,LC150567_Narnaviridae_M
itovirus:0.0716)74:0.26696,LN827943_Narnaviridae_Narnavirus:0.33856):0
.028588,L31849_Narnaviridae_Mitovirus:0.367148):0.010102,(KU057949_Nar
naviridae_Mitovirus:0.030997,KR005622_Narnaviridae_Mitovirus:0.030997)
98:0.346252):0.026161,MF196914_Narnaviridae_Mitovirus:0.40341):0.01063
7,(LC150566_Narnaviridae_Mitovirus:0.067877,LN827942_Narnaviridae_Mito
virus:0.067877):0.34617):0.005598):0.031927,HQ992989_Narnaviridae_Mito
virus:0.451572):0.029078,HE717021_Narnaviridae_Mitovirus:0.480651):0.0
05847,KT921180_Narnaviridae_Mitovirus:0.486498)100:0.493021,((U90136_N
arnaviridae_Narnavirus:0.734404,AF039063_Narnaviridae_Narnavirus:0.734
404)81:0.111074,LC150604_Narnaviridae_Narnavirus:0.845477)94:0.134042)
81:0.020481,((KU882057_Narnaviridae_Narnavirus:0.925086,LC150605_Narna
viridae_Narnavirus:0.925086)98:0.074914,(AF334111_Leviviridae_Leviviru
s:0.727787,((V00642_Leviviridae_Levivirus:0.273229,(X03869_Leviviridae
_Levivirus:0.190111,(JX045649_Leviviridae_Levivirus:0.044733,JX045650_
Leviviridae_Levivirus:0.044733)99:0.145378):0.083118)99:0.300012,(X801
91_Leviviridae_Levivirus:0.500727,(AF059242_Leviviridae_Allolevivirus:
0.056758,(X07489_Leviviridae_Allolevivirus:0.008671,EF068134_Levivirid

ae_Allolevivirus:0.008671)100:0.048086)100:0.443969):0.072513)83:0.154
547)99:0.272213)75:0.0)88:0.0)100;

Figure S8 UPGMA dendrogram of Group IV ss(+)RNA viruses

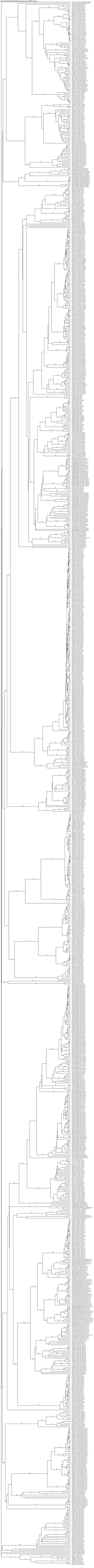

Figure S9 UPGMA dendrogram of Group I dsDNA viruses and unclassified prokaryotic viruses (NEWICK format)

((((((((((((((((((((((((((((((((((Query0472_KY065478:0.022277,Que
ry0058_KJ417497:0.022277)96:0.014846,Query0535_KY065480:0.037123)98:0.
035756,(Query0315_KY065450:0.057799,Query0035_KT337356:0.057799)74:0.0
15081):0.027635,Query0255_KY065472:0.100514):0.013985,Query0367_KY0654
97:0.114499):0.012484,((Query0452_KT337341:0.092364,Query0021_KY065451
:0.092364):0.017083,Query0482_KY065476:0.109448):0.017535):0.018062,((
Query0539_KY065488:0.059174,Query0272_KT337352:0.059174)98:0.041052,(Q
uery0256_KY065473:0.057114,Query0102_KY065452:0.057114)98:0.043112)78:
0.044819)94:0.131863,Query0366_KY065496:0.276908)92:0.091237,((((((Qu
ery0538_KY065486:0.09616,Query0453_KT337340:0.09616)90:0.045005,Query0
271_KT337355:0.141164):0.033879,(Query0273_KY065505:0.120407,Query0047
_KT337339:0.120407)72:0.054636):0.036464,Query0037_KT337354:0.211507):
0.001483,Query0107_KY065456:0.21299):0.01213,Query0268_KY065500:0.2251
2)92:0.065835,(Query0250_KY065475:0.047343,Query0249_KY065474:0.047343
)100:0.243611)88:0.07719)98:0.265024,((((Query0424_KY065466:0.088266,
Query0277_KY065468:0.088266)100:0.233949,Query0279_KY065467:0.322215)6
4:0.021944,Query0023_KY065457:0.344159):0.080191,Query0286_KY065464:0.
42435)90:0.150473,Query0155_KC348598:0.574824)88:0.058345):0.066024,Qu
ery0095_AF195902:0.699192):0.055111,((KC595518_Myoviridae_Abouovirus:0
.053887,KC595517_Myoviridae_Abouovirus:0.053887)100:0.244778,(KT151956
_Myoviridae_Jimmervirus:0.043048,KC595514_Myoviridae_Jimmervirus:0.043
048)100:0.255617)100:0.455639):0.013547,(((Query0002_MG765275:0.243287
,AY236756_Siphoviridae_Phijl1virus:0.243287)98:0.067895,(JN051154_Siph
oviridae_Phijl1virus:0.052204,JX486087_Siphoviridae_Phijl1virus:0.0522
04)100:0.258977)100:0.381138,((GQ478084_Siphoviridae_Phifelvirus:0.085
76,GQ478081_Siphoviridae_Phifelvirus:0.08576)100:0.356097,GQ478086_Sip
hoviridae_Phifelvirus:0.441857)100:0.250463)88:0.075531):0.018113,((((
(((((AP001553_Siphoviridae_Phietavirus:0.180556,AY954963_Siphoviridae_
Phietavirus:0.180556):0.04049,AY954964_Siphoviridae_Phietavirus:0.2210
46):0.011869,AY954962_Siphoviridae_Phietavirus:0.232915):0.038152,((DQ
908929_Siphoviridae_Phietavirus:0.105205,AY954965_Siphoviridae_Phietav
irus:0.105205)100:0.134343,AB370268_Siphoviridae_Phietavirus:0.239548)
:0.031518)70:0.039674,((AY954967_Siphoviridae_Phietavirus:0.148828,AY9
54966_Siphoviridae_Phietavirus:0.148828)100:0.094911,AY954968_Siphovir
idae_Phietavirus:0.243739)94:0.067002):0.027141,((DQ530362_Siphovirida
e_Phietavirus:0.219617,AP008954_Siphoviridae_Phietavirus:0.219617):0.0
19376,AY954960_Siphoviridae_Phietavirus:0.238993)84:0.098889)98:0.0936
08,((((((DQ530360_Siphoviridae_Phietavirus:0.148139,DQ530359_Siphovir
idae_Phietavirus:0.148139):0.024249,(DQ517338_Siphoviridae_Phietavirus
:0.066878,AY954952_Siphoviridae_Phietavirus:0.066878)100:0.10551):0.03
6519,((AY954951_Siphoviridae_Phietavirus:0.147559,AF424781_Siphovirida
e_Phietavirus:0.147559):0.04379,AP008953_Siphoviridae_Phietavirus:0.19
1349):0.017558):0.019905,(AB370205_Siphoviridae_Phietavirus:0.199654,A
Y954953_Siphoviridae_Phietavirus:0.199654):0.029158):0.015784,GU477322
_Siphoviridae_Phietavirus:0.244596)86:0.047274,EU861004_Siphoviridae_P
hietavirus:0.29187)100:0.125632,AY954950_Siphoviridae_Phietavirus:0.41
7502):0.013989)68:0.070819,((((DQ834250_Siphoviridae_Phietavirus:0.18
8092,JN192401_Siphoviridae_Phietavirus:0.188092)80:0.031456,DQ831957_S
iphoviridae_Phietavirus:0.219547)70:0.047284,JN192400_Siphoviridae_Phi

etavirus:0.266831)92:0.091404,AY954959_Siphoviridae_Phietavirus:0.3582
35):0.03197,AY954958_Siphoviridae_Phietavirus:0.390206)98:0.112104)100
:0.205102,Query0399_CP018841:0.707411)74:0.078553):0.051273,(((((((((
((((Query0400_KY705260:0.018259,Query0149_KY705261:0.018259)86:0.0190
22,Query0534_KY705276:0.03728):0.010327,Query0394_KY705266:0.047607):0
.00253,Query0550_KY705282:0.050138)68:0.023513,(((Query0398_KY705262:0
.037562,Query0396_KY705264:0.037562):0.013362,AF085222_Siphoviridae_Sf
i21dt1virus:0.050923):0.002146,Query0144_KY705263:0.053069)60:0.020582
):0.003046,Query0225_KY705257:0.076698):0.010832,Query0559_KY705273:0.
08753):0.015583,(((((Query0551_KY705283:0.000825,Query0395_KY705267:0.
000825)100:0.03142,Query0397_KY705265:0.032245)62:0.006175,Query0528_K
Y705274:0.03842):0.025804,Query0561_KY705275:0.064224)84:0.025094,(Que
ry0560_KY705272:0.067366,Query0226_KY705256:0.067366)62:0.021952):0.01
3794):0.011802,((Query0547_KY705287:0.06557,Query0544_KY705284:0.06557
)60:0.02427,Query0558_KY705270:0.089841):0.025073):0.015812,AF115102_S
iphoviridae_Sfi21dt1virus:0.130726)74:0.029454,Query0078_MG708275:0.16
0181)60:0.013533,AF115103_Siphoviridae_Sfi21dt1virus:0.173714)100:0.06
3299,(((((((Query0554_KY705289:0.060785,Query0131_KY705290:0.060785)7
6:0.031241,Query0553_KY705288:0.092027)78:0.016785,FJ236310_Siphovirid
ae_Sfi21dt1virus:0.108812):0.010406,Query0345_MG708274:0.119218):0.027
221,Query0224_KY705251:0.146439):0.00271,Query0229_KY705259:0.14915):0
.01972,Query0557_KY705271:0.16887)60:0.013459,AF145054_Siphoviridae_Sf
i21dt1virus:0.182329)100:0.054684)100:0.317364,((((((Query0546_KY70528
6:0.051189,EF529515_Siphoviridae_Sfi11virus:0.051189):0.016262,(Query0
545_KY705285:0.035307,AY699705_Siphoviridae_Sfi11virus:0.035307)94:0.0
32144)84:0.029173,((Query0404_KY705269:0.015777,Query0154_KY705268:0.0
15777)100:0.059388,FJ226752_Siphoviridae_Sfi11virus:0.075165)86:0.0214
59)100:0.066918,((Query0555_KY705279:0.033692,Query0548_KY705280:0.033
692)82:0.018266,Query0556_KY705278:0.051958)100:0.111583)60:0.044804,(
AF158600_Siphoviridae_Sfi11virus:0.141267,U88974_Siphoviridae_Sfi11vir
us:0.141267)64:0.067078)78:0.048453,((Query0549_KY705281:0.112665,Quer
y0230_KY705258:0.112665)90:0.062611,Query0238_KY705277:0.175275)96:0.0
81523)100:0.297578):0.144651,((((((((((Query0537_KY065487:0.041418,Qu
ery0372_KY065491:0.041418)84:0.010631,Query0118_KY065455:0.05205)84:0.
033802,Query0370_KY065493:0.085852)62:0.026756,Query0369_KY065492:0.11
2608)62:0.030965,Query0371_KY065490:0.143573):0.031107,Query0511_HF563
658:0.174679):0.008123,Query0269_KY065501:0.182802):0.026737,(Query047
3_KY065479:0.136755,Query0270_KY065502:0.136755)62:0.072784)100:0.2921
33,Query0496_KY349816:0.501671)68:0.129129,Query0425_KY065465:0.630801
):0.03181,(((Query0228_KY705254:0.036531,Query0080_KY705252:0.036531):
0.018024,Query0222_KY705253:0.054555)100:0.154113,Query0227_KY705255:0
.208669)100:0.453942):0.036416):0.13821):0.016577,((((EU874396_Siphovi
ridae_Cecivirus:0.137939,GU229986_Siphoviridae_Cecivirus:0.137939)100:
0.464624,Query0316_MF176161:0.602563):0.071336,Query0243_KX190833:0.67
3898):0.032376,((EU887664_Siphoviridae_Tp21virus:0.065842,JX887877_Sip
hoviridae_Tp21virus:0.065842)100:0.592845,DQ289555_Siphoviridae_Wbetav
irus:0.658688):0.047587):0.14754):0.004073,((((((((((((Query0328_MG72
7700:0.061495,KT361650_Siphoviridae_Sitaravirus:0.061495)78:0.048714,(
Query0384_MG727699:0.017549,Query0234_MG727695:0.017549)100:0.092661)7
4:0.038829,Query0330_MG727702:0.149039):0.002935,(((Query0233_MG727696
:0.001096,KP296793_Siphoviridae_Sitaravirus:0.001096)100:0.053163,KP20
2972_Siphoviridae_Sitaravirus:0.05426)88:0.052892,(Query0327_MG727701:

0.068324,KP296796_Siphoviridae_Sitaravirus:0.068324)80:0.038828)72:0.0
44822):0.023141,((Query0386_MG727698:0.072574,KP296795_Siphoviridae_Si
taravirus:0.072574)62:0.053881,KP296791_Siphoviridae_Sitaravirus:0.126
455):0.048659)100:0.269843,KT361651_Siphoviridae_Harrisonvirus:0.44495
8):0.112011,(Query0232_MG727697:0.236354,KT361654_Siphoviridae_Vegasvi
rus:0.236354)98:0.320615)90:0.072221,(Query0174_DQ453159:0.560333,Quer
y0064_MF595878:0.560333)74:0.068857):0.038832,((((((Query0543_KY05662
0:0.085284,Query0407_MG770897:0.085284)78:0.038735,EU861005_Siphovirid
ae_Triavirus:0.124019):0.012783,(AF424782_Siphoviridae_Triavirus:0.111
06,AY954956_Siphoviridae_Triavirus:0.11106):0.025743)66:0.024794,AY954
957_Siphoviridae_Triavirus:0.161597)100:0.106952,AB045978_Siphoviridae
_Triavirus:0.268548)94:0.047818,AY954955_Siphoviridae_Triavirus:0.3163
67)100:0.327135,((AF424783_Siphoviridae_Biseptimavirus:0.187765,AB2435
56_Siphoviridae_Biseptimavirus:0.187765)100:0.204,AY508486_Siphovirida
e_Biseptimavirus:0.391765)100:0.251737)60:0.024519):0.073621,((DQ34006
4_Myoviridae_Svunavirus:0.114459,DQ840344_Myoviridae_Svunavirus:0.1144
59)100:0.475856,Query0374_KU160495:0.590314)72:0.151328):0.030609,((Qu
ery0504_KX190835:0.664858,Query0502_KX190832:0.664858):0.04518,(AJ3122
40_Siphoviridae_Psavirus:0.063171,JX120799_Siphoviridae_Psavirus:0.063
171)100:0.646867):0.062214):0.037545,((((KF188410_Siphoviridae_C5virus
:0.082497,EU340421_Siphoviridae_C5virus:0.082497)98:0.039096,KJ564037_
Siphoviridae_C5virus:0.121592):0.015724,(AY739900_Siphoviridae_C5virus
:0.127909,KJ564038_Siphoviridae_C5virus:0.127909):0.009407)94:0.030654
,KJ564036_Siphoviridae_C5virus:0.16797)100:0.641826):0.024209,(((((((
((((Query0292_KY624612:0.0,Query0029_KY613597:0.0)100:0.0,Query0311_K
Y566218:0.0)100:0.0,Query0387_KY624615:0.0)100:0.0,Query0388_KY624614:
0.0)100:0.0,Query0389_KY624616:0.0)100:0.0,Query0391_KY624610:0.0)100:
0.0,Query0392_KY624613:0.0)100:2.6e-05,Query0390_KY624611:2.6e-
05)100:0.615528,KU998237_Siphoviridae_Vendettavirus:0.615554):0.060894
,(((Query0368_KY065495:0.172112,Query0042_KY065462:0.172112)100:0.2301
06,Query0365_KY065498:0.402218)98:0.045415,((Query0041_KY065504:0.0287
38,Query0038_KY065503:0.028738)100:0.1245,Query0536_KY065485:0.153239)
100:0.294395)98:0.228816):0.038884,((Query0565_MG711465:0.583938,Query
0171_MF893271:0.583938)70:0.066775,Query0393_KX965989:0.650713)72:0.06
462):0.053796,((((KX620753_Siphoviridae_Doucettevirus:0.16565,KX62075
1_Siphoviridae_Doucettevirus:0.16565)64:0.029524,KX620750_Siphoviridae
_Doucettevirus:0.195174):0.021449,KX620754_Siphoviridae_Doucettevirus:
0.216623)100:0.3257,(KX620749_Siphoviridae_Anatolevirus:0.085152,KX620
748_Siphoviridae_Anatolevirus:0.085152)100:0.457171)94:0.087157,(Query
0523_KU984980:0.003474,KU984979_Siphoviridae_Pfr1virus:0.003474)100:0.
626006)94:0.13965):0.02539,((((KU160643_Siphoviridae_Korravirus:0.0227
95,KU160640_Siphoviridae_Korravirus:0.022795)62:0.002502,KU160659_Siph
oviridae_Korravirus:0.025297)100:0.033958,(KU160661_Siphoviridae_Korra
virus:0.036199,KU160648_Siphoviridae_Korravirus:0.036199)96:0.023056)9
8:0.05041,(((KU160653_Siphoviridae_Korravirus:0.014377,KU160645_Siphov
iridae_Korravirus:0.014377)100:0.024538,KU160672_Siphoviridae_Korravir
us:0.038915)100:0.02088,KU160652_Siphoviridae_Korravirus:0.059795)98:0
.049869)100:0.684855):0.039485):0.023882):0.029606,(((((((((((FJ84888
5_Siphoviridae_Sk1virus:0.00262,FJ848884_Siphoviridae_Sk1virus:0.00262
)100:0.092759,FJ848883_Siphoviridae_Sk1virus:0.095379):0.028639,(Query
0117_KY554760:0.011442,Query0116_KY554761:0.011442)100:0.112576):0.031
755,((Query0200_KX379665:0.086225,AF009630_Siphoviridae_Sk1virus:0.086

225)60:0.039399,(Query0350_KX379670:0.078178,Query0349_KX379671:0.0781
78):0.047447):0.030148):0.012426,((JQ740804_Siphoviridae_Sk1virus:0.04
7235,JQ740813_Siphoviridae_Sk1virus:0.047235)60:0.005887,JQ740789_Siph
oviridae_Sk1virus:0.053122)100:0.115077):0.006581,((DQ054536_Siphoviri
dae_Sk1virus:0.113218,DQ227763_Siphoviridae_Sk1virus:0.113218)64:0.028
806,FJ848881_Siphoviridae_Sk1virus:0.142024):0.032756):0.00942,((JQ740
787_Siphoviridae_Sk1virus:0.099073,JQ740788_Siphoviridae_Sk1virus:0.09
9073)68:0.031766,FJ848882_Siphoviridae_Sk1virus:0.130839):0.053362):0.
043569,(Query0203_KX379668:0.041499,Query0202_KX379667:0.041499)100:0.
18627):0.008371,(((((Query0204_KX379669:0.008317,Query0201_KX379666:0.
008317)100:0.061048,Query0199_KX379664:0.069365)78:0.040728,EU221285_S
iphoviridae_Sk1virus:0.110093)72:0.040071,(Query0348_KX379672:0.007413
,Query0347_KX379673:0.007413)100:0.142751):0.025141,((AF011378_Siphovi
ridae_Sk1virus:0.018606,DQ227764_Siphoviridae_Sk1virus:0.018606)100:0.
075702,GQ979703_Siphoviridae_Sk1virus:0.094308)88:0.080997)60:0.060835
)100:0.556126,(((((((((Query0514_KY554776:0.000418,Query0119_KY554769
:0.000418):8e-
05,Query0516_KY554770:0.000498)74:0.003407,Query0120_KY554768:0.003905
)90:0.010872,(Query0519_KY554773:0.000997,Query0513_KY554775:0.000997)
100:0.013779):0.004757,Query0512_KY554774:0.019533)100:0.162625,Query0
515_KY554777:0.182158)88:0.034344,(Query0518_KY554772:0.031698,Query05
17_KY554771:0.031698)100:0.184805)100:0.480431,(KJ804259_Siphoviridae_
Sextaecvirus:0.184707,KF929199_Siphoviridae_Sextaecvirus:0.184707)100:
0.512227):0.009456,((((Query0205_MG252693:0.103135,Query0004_MG765276:
0.103135)100:0.119057,Query0148_MG744354:0.222192)78:0.030529,Query030
8_KX223815:0.252721)100:0.424542,Query0312_KY363359:0.677263):0.029126
):0.039467,((((((Query0115_KY554762:0.001154,Query0114_KY554763:0.0011
54)100:0.007115,Query0113_KY554764:0.008269)100:0.029307,Query0112_KY5
54765:0.037576)100:0.277159,(Query0111_KY554766:0.000365,Query0110_KY5
54767:0.000365)100:0.314371)100:0.276945,Query0499_MF580956:0.59168)96
:0.107931,Query0474_KY979235:0.699612):0.046245):0.04641):0.030266,(((
((KC013023_Siphoviridae_Lmd1virus:0.033692,JQ659259_Siphoviridae_Lmd1v
irus:0.033692)94:0.023073,KC013024_Siphoviridae_Lmd1virus:0.056765)100
:0.107311,(KC013025_Siphoviridae_Lmd1virus:0.094257,KC013022_Siphoviri
dae_Lmd1virus:0.094257)94:0.069819)86:0.058956,KC013021_Siphoviridae_L
md1virus:0.223032)100:0.143558,(((KM262192_Siphoviridae_Una4virus:0.04
7358,GQ451696_Siphoviridae_Una4virus:0.047358)100:0.08445,(KM262191_Si
phoviridae_Una4virus:0.009904,KC013026_Siphoviridae_Una4virus:0.009904
)100:0.121904):0.02944,(KC013029_Siphoviridae_Una4virus:0.010021,KC013
027_Siphoviridae_Una4virus:0.010021)100:0.151228)100:0.205341)100:0.45
5943):0.039484,((((HE962497_Siphoviridae_Sap6virus:0.071224,KF192053_S
iphoviridae_Sap6virus:0.071224):0.020709,JF731128_Siphoviridae_Sap6vir
us:0.091933)86:0.03204,(KJ094032_Siphoviridae_Sap6virus:0.087568,AB712
291_Siphoviridae_Sap6virus:0.087568)88:0.036405)100:0.466601,(((JX4422
41_Siphoviridae_P70virus:0.060827,KJ094021_Siphoviridae_P70virus:0.060
827)64:0.029335,(JX126920_Siphoviridae_P70virus:0.037531,JX126919_Siph
oviridae_P70virus:0.037531)100:0.052631):0.015741,KJ094020_Siphovirida
e_P70virus:0.105903)100:0.484671)100:0.271442):0.025477):0.034923,((((
((((KU160668_Myoviridae_Sonnyvirus:0.003574,KU160656_Myoviridae_Sonnyv
irus:0.003574)100:0.016702,KU160665_Myoviridae_Sonnyvirus:0.020276)100
:0.038301,KU647629_Myoviridae_Chippewavirus:0.058577)78:0.006719,(KU16
0651_Myoviridae_Jawnskivirus:0.028071,KT365401_Myoviridae_Jawnskivirus

:0.028071)98:0.037225)100:0.501641,((KX522650_Myoviridae_Arvunavirus:0
.015037,KU160660_Myoviridae_Arv1virus:0.015037)100:0.104414,KM879463_M
yoviridae_Arvunavirus:0.119451)100:0.447486)100:0.310372,(((Query0275_
KU886268:0.604086,KU160654_Siphoviridae_Laroyevirus:0.604086)100:0.196
21,Query0097_KY006853:0.800296):0.044902,((Query0468_KT070868:0.487578
,KY565347_Siphoviridae_Tp84virus:0.487578)100:0.251803,Query0220_LN881
738:0.73938):0.105818):0.032112):0.027074,(KU647627_Siphoviridae_Kelle
ziovirus:0.040152,KU647626_Siphoviridae_Kelleziovirus:0.040152)100:0.8
64232):0.006666,(((((KF669651_Siphoviridae_Andromedavirus:0.012757,KC3
30684_Siphoviridae_Andromedavirus:0.012757)64:0.006001,KC330679_Siphov
iridae_Andromedavirus:0.018758)100:0.024964,(KC330682_Siphoviridae_And
romedavirus:0.005024,KC330680_Siphoviridae_Andromedavirus:0.005024)100
:0.038698)78:0.046939,((KF669659_Siphoviridae_Andromedavirus:0.026289,
KC330683_Siphoviridae_Andromedavirus:0.026289)74:0.022448,KF669648_Sip
hoviridae_Andromedavirus:0.048737)78:0.041924)100:0.779402,(((KM236248
_Podoviridae_Pagevirus:0.045355,KP411017_Podoviridae_Pagevirus:0.04535
5)82:0.017879,(KF669660_Podoviridae_Pagevirus:0.032854,KF669655_Podovi
ridae_Pagevirus:0.032854)90:0.030381)100:0.136347,KM236247_Podoviridae
_Pagevirus:0.199581)100:0.670482)60:0.040987):0.011365):0.00665,((((((
((((((((((GQ303263_Siphoviridae_L5virus:0.036391,JF704093_Siphovirid
ae_L5virus:0.036391):0.013377,JX307702_Siphoviridae_L5virus:0.049768)7
6:0.015778,JN699015_Siphoviridae_L5virus:0.065546)100:0.07635,((JX4116
19_Siphoviridae_L5virus:0.018962,JX307704_Siphoviridae_L5virus:0.01896
2)78:0.009045,JQ512844_Siphoviridae_L5virus:0.028007)100:0.113889)100:
0.075,((((JF704101_Siphoviridae_L5virus:0.01591,JF704098_Siphoviridae_
L5virus:0.01591)100:0.021293,HM755814_Siphoviridae_L5virus:0.037203)90
:0.027439,AY129332_Siphoviridae_L5virus:0.064642)90:0.031391,((JF70411
1_Siphoviridae_L5virus:0.032083,JF957058_Siphoviridae_L5virus:0.032083
):0.009882,Query0414_AP018485:0.041966)100:0.054066)100:0.120864)96:0.
052275,(Query0412_AP018486:0.22565,JF957060_Siphoviridae_L5virus:0.225
65)94:0.043521)90:0.029242,((((((JN408461_Siphoviridae_L5virus:0.14280
3,GU339467_Siphoviridae_L5virus:0.142803)80:0.034966,JN408460_Siphovir
idae_L5virus:0.177769)74:0.026455,(JF946695_Siphoviridae_L5virus:0.131
306,DQ398043_Siphoviridae_L5virus:0.131306)98:0.072919):0.019099,EU744
250_Siphoviridae_L5virus:0.223324):0.011813,(JF704110_Siphoviridae_L5v
irus:0.063923,JN699005_Siphoviridae_L5virus:0.063923)100:0.171214)70:0
.047956,((JF937094_Siphoviridae_L5virus:0.045622,JF704097_Siphoviridae
_L5virus:0.045622)66:0.017111,(JN699019_Siphoviridae_L5virus:0.038435,
JN049605_Siphoviridae_L5virus:0.038435)94:0.024297)100:0.220361)74:0.0
15319)86:0.036566,((((JF704107_Siphoviridae_L5virus:0.015616,JN408459_
Siphoviridae_L5virus:0.015616)100:0.02549,JQ684677_Siphoviridae_L5viru
s:0.041106)100:0.043073,JN083852_Siphoviridae_L5virus:0.084179)100:0.1
40274,(JN831654_Siphoviridae_L5virus:0.072525,JX015524_Siphoviridae_L5
virus:0.072525)100:0.151929)100:0.110525):0.014356,KU998249_Siphovirid
ae_Soupsvirus:0.349335)96:0.054995,(JN116826_Siphoviridae_Unassigned:0
.08452,JN116827_Siphoviridae_Rer2virus:0.08452)100:0.31981)86:0.04099,
((((((((((AY500152_Siphoviridae_L5virus:0.038984,JF937108_Siphovirida
e_L5virus:0.038984)92:0.034108,JF937110_Siphoviridae_L5virus:0.073092)
:0.007394,JN020140_Siphoviridae_L5virus:0.080486):0.011772,((AY500153_
Siphoviridae_L5virus:0.063466,JF957057_Siphoviridae_L5virus:0.063466):
0.008471,JN153086_Siphoviridae_L5virus:0.071937):0.020321):0.000811,((
JX307705_Siphoviridae_L5virus:0.06257,EU744249_Siphoviridae_L5virus:0.

06257)60:0.017949,JF937100_Siphoviridae_L5virus:0.080519):0.01255):0.0
03961,(EU744251_Siphoviridae_L5virus:0.085646,JN698998_Siphoviridae_L5
virus:0.085646):0.011384):0.010959,((EU826470_Siphoviridae_L5virus:0.0
95404,GU247132_Siphoviridae_L5virus:0.095404):0.007031,(JN398369_Sipho
viridae_L5virus:0.092296,JF937099_Siphoviridae_L5virus:0.092296):0.010
139):0.005554):0.007062,(JN699016_Siphoviridae_L5virus:0.088448,AF2716
93_Siphoviridae_L5virus:0.088448)72:0.026603):0.017338,(((JN687951_Sip
hoviridae_L5virus:0.090427,JF937103_Siphoviridae_L5virus:0.090427):0.0
07444,(JN572689_Siphoviridae_L5virus:0.046977,JQ698665_Siphoviridae_L5
virus:0.046977)100:0.050894):0.010356,JN153085_Siphoviridae_L5virus:0.
108228)72:0.024162):0.006965,EU744248_Siphoviridae_L5virus:0.139355):0
.010667,JN699000_Siphoviridae_L5virus:0.150022)100:0.295298)100:0.2884
96,(((((KC700557_Siphoviridae_Likavirus:0.031224,KC700556_Siphoviridae
_Likavirus:0.031224):0.008349,KC700558_Siphoviridae_Likavirus:0.039573
)100:0.042603,KT184391_Siphoviridae_Likavirus:0.082176)80:0.04081,(((K
T152029_Siphoviridae_Likavirus:0.017547,KT124227_Siphoviridae_Likaviru
s:0.017547)88:0.021567,KT184390_Siphoviridae_Likavirus:0.039114):0.011
743,KT124229_Siphoviridae_Likavirus:0.050857)100:0.072129)98:0.118873,
(((JX182370_Siphoviridae_R4virus:0.007247,JX262376_Siphoviridae_R4viru
s:0.007247)100:0.188199,JX182369_Siphoviridae_Unassigned:0.195446)70:0
.014645,(JX889246_Siphoviridae_Camvirus:0.056805,KT186228_Siphoviridae
_Camvirus:0.056805)100:0.153286):0.031768)100:0.491957)64:0.085229,(((
((((((((((((((JX570710_Siphoviridae_Pa6virus:0.01697,JX262223_Siphov
iridae_Pa6virus:0.01697):0.000607,KJ578792_Siphoviridae_Pa6virus:0.017
577):0.001798,JX262220_Siphoviridae_Pa6virus:0.019375):0.000601,((KJ57
8759_Siphoviridae_Pa6virus:0.016198,KJ578758_Siphoviridae_Pa6virus:0.0
16198):0.002232,JX262217_Siphoviridae_Pa6virus:0.01843):0.001546):0.00
2419,(KR337644_Siphoviridae_Pa6virus:0.018132,KJ578774_Siphoviridae_Pa
6virus:0.018132):0.004263):0.001482,((JX570706_Siphoviridae_Pa6virus:0
.02018,KR337650_Siphoviridae_Pa6virus:0.02018):0.002895,JX262216_Sipho
viridae_Pa6virus:0.023075):0.000802):0.000744,(KR337653_Siphoviridae_P
a6virus:0.009249,KR337649_Siphoviridae_Pa6virus:0.009249)100:0.015372)
:0.000273,(((KR337647_Siphoviridae_Pa6virus:0.019065,KJ578781_Siphovir
idae_Pa6virus:0.019065):0.002979,(KJ578788_Siphoviridae_Pa6virus:0.016
735,KJ578779_Siphoviridae_Pa6virus:0.016735):0.00531):0.002261,(KJ5787
76_Siphoviridae_Pa6virus:0.023762,KJ578765_Siphoviridae_Pa6virus:0.023
762):0.000544):0.000587):0.002331,JX262221_Siphoviridae_Pa6virus:0.027
224):0.000437,FJ706172_Siphoviridae_Pa6virus:0.027661):0.000411,((((KJ
578787_Siphoviridae_Pa6virus:0.0212,KR337654_Siphoviridae_Pa6virus:0.0
212):0.004053,(KJ578784_Siphoviridae_Pa6virus:6.2e-
05,KJ578783_Siphoviridae_Pa6virus:6.2e-
05)100:0.025191):0.00039,KJ578790_Siphoviridae_Pa6virus:0.025643):0.00
0483,(KJ578791_Siphoviridae_Pa6virus:0.021775,JX570712_Siphoviridae_Pa
6virus:0.021775):0.004351):0.001946):0.001352,(((JX570714_Siphoviridae
_Pa6virus:0.02081,KJ578767_Siphoviridae_Pa6virus:0.02081):0.001759,(JX
262224_Siphoviridae_Pa6virus:0.010346,JX262225_Siphoviridae_Pa6virus:0
.010346)96:0.012223):0.00484,((JX262215_Siphoviridae_Pa6virus:0.020409
,JX262222_Siphoviridae_Pa6virus:0.020409):0.004441,DQ431235_Siphovirid
ae_Pa6virus:0.02485):0.002558):0.002015):0.002092,KJ578785_Siphovirida
e_Pa6virus:0.031516):0.003913,(JX570705_Siphoviridae_Pa6virus:0.023345
,JX262218_Siphoviridae_Pa6virus:0.023345)88:0.012083):0.000811,KR33764
5_Siphoviridae_Pa6virus:0.036239):0.002061,KJ578761_Siphoviridae_Pa6vi

rus:0.0383):0.00133,(((((((KR337648_Siphoviridae_Pa6virus:0.017639,KJ5
78770_Siphoviridae_Pa6virus:0.017639):0.006,KR337643_Siphoviridae_Pa6v
irus:0.023639):0.001872,(JX570713_Siphoviridae_Pa6virus:0.024614,JX570
702_Siphoviridae_Pa6virus:0.024614):0.000897):0.001573,JX262219_Siphov
iridae_Pa6virus:0.027084):0.002811,KJ578780_Siphoviridae_Pa6virus:0.02
9895):0.001614,KJ722067_Siphoviridae_Pa6virus:0.031509):0.001067,(((((
KJ578789_Siphoviridae_Pa6virus:5.3e-
05,KJ578778_Siphoviridae_Pa6virus:5.3e-
05)92:0.000441,KJ578786_Siphoviridae_Pa6virus:0.000494)100:0.019359,KJ
578773_Siphoviridae_Pa6virus:0.019853):0.005972,KR337651_Siphoviridae_
Pa6virus:0.025825):0.002798,KR337652_Siphoviridae_Pa6virus:0.028623):0
.003952):0.007055):0.001378,FJ706171_Siphoviridae_Pa6virus:0.041008):0
.007123,KR337646_Siphoviridae_Pa6virus:0.048131)100:0.770914):0.042097
,((((((((((((((JF937098_Siphoviridae_Che8virus:0.123072,GU060500_Sip
hoviridae_Che8virus:0.123072):0.037607,FJ174690_Siphoviridae_Che8virus
:0.160679):0.015198,(JN398368_Siphoviridae_Che8virus:0.140345,JN859129
_Siphoviridae_Che8virus:0.140345):0.035533):0.016602,(((HQ728524_Sipho
viridae_Che8virus:0.067566,JX121091_Siphoviridae_Che8virus:0.067566)10
0:0.0684,JN698996_Siphoviridae_Che8virus:0.135966)76:0.049624,EF536069
_Siphoviridae_Che8virus:0.185589):0.00689):0.030208,((JN020141_Siphovi
ridae_Che8virus:0.159983,JF937093_Siphoviridae_Che8virus:0.159983):0.0
45812,JN699012_Siphoviridae_Che8virus:0.205795):0.016892):0.019213,((J
Q300538_Siphoviridae_Che8virus:0.160443,JN020142_Siphoviridae_Che8viru
s:0.160443)76:0.054422,AY129330_Siphoviridae_Che8virus:0.214865):0.027
035):0.013662,((JN020143_Siphoviridae_Che8virus:0.16976,JX411620_Sipho
viridae_Che8virus:0.16976)94:0.057186,FJ174692_Siphoviridae_Che8virus:
0.226945):0.028616):0.013148,(((((DQ398050_Siphoviridae_Che8virus:0.13
743,EU816590_Siphoviridae_Che8virus:0.13743):0.03759,JF704117_Siphovir
idae_Che8virus:0.175019):0.027655,(DQ398045_Siphoviridae_Che8virus:0.1
48132,JN542517_Siphoviridae_Che8virus:0.148132):0.054542):0.030429,JF9
37102_Siphoviridae_Che8virus:0.233103):0.010038,FJ174693_Siphoviridae_
Che8virus:0.243141):0.02557)100:0.294327,((AY129336_Siphoviridae_Che8v
irus:0.200185,JQ809702_Siphoviridae_Che8virus:0.200185)98:0.052176,JF7
04115_Siphoviridae_Che8virus:0.252361)100:0.310676)98:0.086537,(JN6989
93_Siphoviridae_Corndogvirus:0.037101,AY129335_Siphoviridae_Corndogvir
us:0.037101)100:0.612473):0.007613,(((((EU816588_Siphoviridae_Cjw1viru
s:0.07562,AY129331_Siphoviridae_Cjw1virus:0.07562):0.009299,JF937106_S
iphoviridae_Cjw1virus:0.084919):0.010261,(JN006061_Siphoviridae_Cjw1vi
rus:0.061124,JN412590_Siphoviridae_Cjw1virus:0.061124)82:0.034055):0.0
11807,(((JF937091_Siphoviridae_Cjw1virus:0.047889,DQ398041_Siphovirida
e_Cjw1virus:0.047889)100:0.035868,GQ303265_Siphoviridae_Cjw1virus:0.08
3758):0.007054,EU816591_Siphoviridae_Cjw1virus:0.090811):0.016175)100:
0.488614,(((((AY129338_Siphoviridae_Omegavirus:0.143603,JF937101_Sipho
viridae_Omegavirus:0.143603)100:0.072759,(JF957059_Siphoviridae_Omegav
irus:0.074968,JF937090_Siphoviridae_Omegavirus:0.074968)100:0.141394)1
00:0.054928,JN698997_Siphoviridae_Omegavirus:0.27129)90:0.022711,JN201
525_Siphoviridae_Omegavirus:0.294002)100:0.283245,KJ567043_Siphovirida
e_Gaiavirus:0.577247)64:0.018354)96:0.061587)76:0.038299,((((((KP02720
0_Siphoviridae_Fishburnevirus:0.03187,JN572061_Siphoviridae_Fishburnev
irus:0.03187)98:0.028098,KT347313_Siphoviridae_Fishburnevirus:0.059967
):0.012088,(KC691256_Siphoviridae_Fishburnevirus:0.026894,KF841477_Sip
hoviridae_Fishburnevirus:0.026894)100:0.045161)100:0.175819,(KR080195_

Siphoviridae_Phayoncevirus:0.199659,JN412591_Siphoviridae_Bignuzvirus:
0.199659):0.048215)100:0.225134,(((Query0413_AP018487:0.121153,JN69900
1_Siphoviridae_Brujitavirus:0.121153)100:0.154886,FJ168659_Siphovirida
e_Brujitavirus:0.276039)100:0.131344,(KP027201_Siphoviridae_Che9cvirus
:0.211901,AY129333_Siphoviridae_Che9cvirus:0.211901)100:0.195481)90:0.
065626)94:0.067183,((((((JN624851_Siphoviridae_Redivirus:0.158671,KU93
5727_Siphoviridae_Redivirus:0.158671):0.013248,KF986246_Siphoviridae_B
uttersvirus:0.171919):0.020385,KU935729_Siphoviridae_Redivirus:0.19230
4):0.012754,KU935728_Siphoviridae_Charlievirus:0.205058):0.020363,(KU9
35731_Siphoviridae_Redivirus:0.162361,KC576783_Siphoviridae_Buttersvir
us:0.162361):0.063061):0.013205,(KM588359_Siphoviridae_Charlievirus:0.
156319,JN256079_Siphoviridae_Charlievirus:0.156319)78:0.082308)100:0.3
01564)100:0.155295)72:0.078123,((((JN680858_Siphoviridae_Bronvirus:0.0
63358,JF744988_Siphoviridae_Bronvirus:0.063358)100:0.117226,(JF704108_
Siphoviridae_Bronvirus:0.056492,HM152763_Siphoviridae_Bronvirus:0.0564
92)100:0.124092)100:0.381038,((JF937105_Siphoviridae_Reyvirus:0.184652
,JN699628_Siphoviridae_Bongovirus:0.184652)100:0.28727,DQ398052_Siphov
iridae_Wildcatvirus:0.471922)100:0.0897)100:0.148414,((KJ538721_Siphov
iridae_Marvinvirus:0.060057,JF704100_Siphoviridae_Marvinvirus:0.060057
)100:0.627239,KJ510413_Siphoviridae_Bernal13virus:0.687296)60:0.02274)
94:0.063574):0.009838,(((((((((Query0185_AP018471:0.000163,Query0043_
AP018469:0.000163)88:0.000177,Query0186_AP018470:0.00034)100:0.011072,
JN831653_Siphoviridae_Tm4virus:0.011412)100:0.226147,(JF937104_Siphovi
ridae_Tm4virus:0.0575,JX042579_Siphoviridae_Tm4virus:0.0575)100:0.1800
59):0.047869,(((JN185608_Siphoviridae_Tm4virus:0.027043,HM152764_Sipho
viridae_Tm4virus:0.027043):0.00206,HM152767_Siphoviridae_Tm4virus:0.02
9103):0.010865,JF704106_Siphoviridae_Tm4virus:0.039968)100:0.24546):0.
011295,AF068845_Siphoviridae_Tm4virus:0.296722)92:0.051969,JN243855_Si
phoviridae_Tm4virus:0.348691)100:0.172678,(JN412593_Siphoviridae_Liefi
evirus:0.030601,DQ398042_Siphoviridae_Liefievirus:0.030601)100:0.49076
8)100:0.144088,(KF024728_Siphoviridae_Ff47virus:0.024157,JX901189_Siph
oviridae_Ff47virus:0.024157)100:0.6413)90:0.036558,(KJ028219_Siphoviri
dae_Trigintaduovirus:0.460862,EU203571_Siphoviridae_Gilesvirus:0.46086
2)100:0.241153)76:0.081432)70:0.029464,((((JN412589_Siphoviridae_Patie
ncevirus:0.261392,AY129339_Siphoviridae_Barnyardvirus:0.261392)100:0.0
59995,(EU770222_Siphoviridae_Barnyardvirus:0.188861,FJ174691_Siphoviri
dae_Barnyardvirus:0.188861)100:0.132526)100:0.145004,((DQ398047_Siphov
iridae_Pbi1virus:0.007548,DQ398051_Siphoviridae_Plotvirus:0.007548)100
:0.1852,KJ194582_Siphoviridae_Hawkeyevirus:0.192748)100:0.273643)100:0
.086029,(JF704112_Siphoviridae_Send513virus:0.037143,KF416342_Siphovir
idae_Send513virus:0.037143)100:0.515277)100:0.260492)64:0.042214,(((((
(KX557287_Siphoviridae_Nymphadoravirus:0.051733,KU963257_Siphoviridae_
Nymphadoravirus:0.051733)100:0.086433,KU963255_Siphoviridae_Nymphadora
virus:0.138166)100:0.205476,(KU963249_Siphoviridae_Baxtervirus:0.18161
6,KU963263_Siphoviridae_Baxtervirus:0.181616)100:0.162026)98:0.183218,
KU963247_Siphoviridae_Attisvirus:0.52686)100:0.239094,(KU998234_Siphov
iridae_Wizardvirus:0.095926,KX557286_Siphoviridae_Wizardvirus:0.095926
)100:0.670029):0.053079,((((KU998244_Siphoviridae_Smoothievirus:0.0486
68,KU998247_Siphoviridae_Smoothievirus:0.048668)80:0.015454,KU998246_S
iphoviridae_Smoothievirus:0.064121)100:0.189814,KU998245_Siphoviridae_
Smoothievirus:0.253936)100:0.547639,(Query0354_KR063281:0.736308,Query
0098_JN116824:0.736308):0.065267):0.017458):0.036093):0.006016):0.0209

74,(((((((((((((JN699011_Siphoviridae_Coopervirus:0.028678,JF704094_S
iphoviridae_Coopervirus:0.028678):0.003366,DQ398044_Siphoviridae_Coope
rvirus:0.032044):0.007513,KF279411_Siphoviridae_Coopervirus:0.039557):
0.006306,EU770221_Siphoviridae_Coopervirus:0.045863)70:0.011584,(((JF7
04104_Siphoviridae_Coopervirus:0.009121,KT270441_Siphoviridae_Coopervi
rus:0.009121)100:0.015925,KF279412_Siphoviridae_Coopervirus:0.025046)9
8:0.02282,KF493881_Siphoviridae_Coopervirus:0.047866)74:0.009581)98:0.
042764,KR080194_Siphoviridae_Coopervirus:0.100211)100:0.101419,((KR080
199_Siphoviridae_Acadianvirus:0.053444,JN699007_Siphoviridae_Acadianvi
rus:0.053444)74:0.01473,KF024727_Siphoviridae_Acadianvirus:0.068173)10
0:0.133457)100:0.064224,((((KF713485_Siphoviridae_Pg1virus:0.024955,KJ
174157_Siphoviridae_Pg1virus:0.024955):0.004178,(AF547430_Siphoviridae
_Pg1virus:0.013898,JN006064_Siphoviridae_Pg1virus:0.013898)86:0.015235
):0.003339,(KJ595576_Siphoviridae_Pg1virus:0.014545,KR781349_Siphoviri
dae_Pg1virus:0.014545)96:0.017927)72:0.016687,JN192463_Siphoviridae_Pg
1virus:0.049159)100:0.216695)98:0.047763,(((JN698992_Siphoviridae_Pipe
fishvirus:0.044484,JN699003_Siphoviridae_Pipefishvirus:0.044484):0.008
263,KF493879_Siphoviridae_Pipefishvirus:0.052747)68:0.014406,DQ398049_
Siphoviridae_Pipefishvirus:0.067153)100:0.246465)72:0.045593,((KF02472
2_Siphoviridae_L5virus:0.006845,AY129334_Siphoviridae_Rosebushvirus:0.
006845)100:0.031485,KR997932_Siphoviridae_Rosebushvirus:0.03833)100:0.
320881)100:0.192175,(Query0490_KR011061:0.205836,Query0188_MF477236:0.
205836)100:0.34555)66:0.011261,(Query0040_AP018463:0.005641,Query0039_
AP018462:0.005641)100:0.557006)100:0.208837,(KP876465_Siphoviridae_Ydn
12virus:0.031281,KP876466_Siphoviridae_Ydn12virus:0.031281)100:0.74020
3)78:0.039176,KU160669_Siphoviridae_Tankvirus:0.81066):0.071456):0.008
844,(JX182372_Siphoviridae_Phic31virus:0.202441,AJ550940_Siphoviridae_
Phic31virus:0.202441)100:0.688519):0.021405,((((JN412588_Myoviridae_Bx
z1virus:0.043134,AY129337_Myoviridae_Bxz1virus:0.043134)64:0.006609,JN
204348_Myoviridae_Bxz1virus:0.049743)74:0.00605,JN699627_Myoviridae_Bx
z1virus:0.055793)92:0.018683,(KT281790_Myoviridae_Bxz1virus:0.055492,J
F704092_Myoviridae_Bxz1virus:0.055492)76:0.018984)100:0.83789):0.00162
2,(((((KU998241_Siphoviridae_Woesvirus:0.036624,KU963245_Siphoviridae_
Woesvirus:0.036624)100:0.159203,KU998240_Siphoviridae_Woesvirus:0.1958
26)72:0.026894,((KR011063_Siphoviridae_Tin2virus:0.001288,KR011064_Sip
hoviridae_Tin2virus:0.001288)100:0.05718,KR011062_Siphoviridae_Tin2vir
us:0.058468)100:0.164252)88:0.054542,((KU998243_Siphoviridae_Demosthen
esvirus:0.003937,KU963258_Siphoviridae_Demosthenesvirus:0.003937)100:0
.01826,KU998242_Siphoviridae_Demosthenesvirus:0.022197)100:0.255065)10
0:0.193788,KP790008_Siphoviridae_Gordtnkvirus:0.47105)100:0.442937):0.
015079):0.011248,((((KU647628_Siphoviridae_Mudcatvirus:0.120983,KU1606
42_Siphoviridae_Mudcatvirus:0.120983)100:0.276106,(KU160646_Siphovirid
ae_Gordonvirus:0.038463,KU160641_Siphoviridae_Gordonvirus:0.038463)100
:0.358626)100:0.092439,(GU580942_Siphoviridae_Pepy6virus:0.12339,GU580
941_Siphoviridae_Pepy6virus:0.12339)100:0.366139)96:0.082137,Query0571
_MG711463:0.571665)100:0.368649):0.011849,(((((Query0459_KU886273:0.55
0535,Query0133_MG835450:0.550535)86:0.217262,KR935217_Siphoviridae_Cro
nusvirus:0.767797):0.087077,(KR136260_Siphoviridae_P12002virus:0.11221
1,KR136259_Siphoviridae_P12002virus:0.112211)100:0.742663):0.060399,((
(Query0462_KU886274:0.352342,Query0458_KU886270:0.352342)100:0.261341,
Query0460_KU886272:0.613682)80:0.097151,(Query0457_KU886271:0.495949,Q
uery0276_KU886269:0.495949)96:0.214884):0.20444):0.030291,((Query0461_

KU886275:0.787698,KT355471_Siphoviridae_Decurrovirus:0.787698)74:0.094
654,Query0187_MG269973:0.882352):0.063213):0.006599):0.001548,(((((((
((((((((((((Query0164_MG065663:0.0,Query0158_MG065665:0.0)100:0.01974
1,Query0160_MG065667:0.019741)76:0.012689,(Query0106_MG065671:0.009014
,Query0020_MG065670:0.009014)90:0.023416)100:0.0762,(((Query0252_MG065
657:0.006984,Query0209_MG065681:0.006984)70:0.011987,Query0427_MG06564
3:0.01897):0.003547,(Query0421_MG065649:0.002496,Query0210_MG065680:0.
002496)98:0.020022)100:0.086113):0.01122,(((Query0429_MG065641:0.01519
,Query0028_MG065679:0.01519)94:0.037876,Query0211_MG065687:0.053066)64
:0.007797,Query0430_MG065640:0.060863)100:0.058987)88:0.031106,((Query
0166_MG065669:0.00518,Query0025_MG065675:0.00518)100:0.079667,Query025
9_MG065636:0.084847)96:0.06611)88:0.054922,((AY308796_Siphoviridae_Tls
virus:0.140842,KM236241_Siphoviridae_Tlsvirus:0.140842)70:0.018129,KC1
39513_Siphoviridae_Tlsvirus:0.158972)82:0.046907)76:0.019936,Query0305
_MF996376:0.225814)70:0.027057,(((KP085586_Siphoviridae_T1virus:0.0566
62,KU194206_Siphoviridae_T1virus:0.056662)70:0.009953,JX912252_Siphovi
ridae_T1virus:0.066614)98:0.064093,KU194205_Siphoviridae_T1virus:0.130
707)100:0.122164):0.005402,((((Query0442_KY780482:0.096463,KR269719_Si
phoviridae_Kp36virus:0.096463):0.038655,KT001920_Siphoviridae_Kp36viru
s:0.135118):0.011151,((Query0563_MF415412:0.096223,JF501022_Siphovirid
ae_Kp36virus:0.096223):0.029451,KP658157_Siphoviridae_Kp36virus:0.1256
74):0.020595):0.020764,KR262148_Siphoviridae_Kp36virus:0.167033)100:0.
09124)62:0.025903,((((((KF771239_Siphoviridae_Rogue1virus:0.026051,KF
771237_Siphoviridae_Rogue1virus:0.026051)98:0.016009,KF771238_Siphovir
idae_Rogue1virus:0.04206)100:0.028056,KT825490_Siphoviridae_Rogue1viru
s:0.070115)84:0.02432,KJ668713_Siphoviridae_Rogue1virus:0.094436)78:0.
023047,(KC333879_Siphoviridae_Rogue1virus:0.084499,KC579452_Siphovirid
ae_Rogue1virus:0.084499)78:0.032983)100:0.108682,(((Query0488_KX130960
:0.079963,AM156909_Siphoviridae_Rtpvirus:0.079963)100:0.097121,Query02
07_MG065688:0.177084):0.008341,(Query0100_KY398841:0.143531,JN986845_S
iphoviridae_Rtpvirus:0.143531)68:0.041894)76:0.040739):0.012404,(Query
0300_MF158042:0.177493,Query0157_MF158039:0.177493)92:0.061076)88:0.04
5608)100:0.153183,Query0019_MG065673:0.437359):0.010525,Query0506_MG06
5637:0.447884)66:0.031833,(Query0510_MG065638:0.217271,Query0213_MG065
685:0.217271)100:0.262447)100:0.296298,((((((((((((((((Query0053_KX
581096:0.0,Query0050_KX581093:0.0)100:0.0,Query0054_KX581097:0.0)100:0
.0,Query0056_KX581095:0.0)100:3e-06,Query0309_KY658680:3e-06):2e-
06,(((Query0051_KX581090:0.0,Query0049_KX581092:0.0):0.0,Query0052_KX5
81091:0.0):3e-06,Query0046_KX581099:3e-06)62:2e-06)82:4.8e-
05,Query0532_KY658675:5.3e-05):1.2e-05,Query0055_KX581094:6.5e-
05)72:5.6e-
05,Query0151_KY658677:0.000121)100:0.002813,((Query0542_KX581100:0.000
338,Query0529_KY658678:0.000338)60:0.001256,Query0530_KY658679:0.00159
3)72:0.001341)94:0.003574,Query0045_KX581098:0.006508)100:0.040673,(Qu
ery0533_KY658676:8e-06,Query0531_KY658674:8e-
06)100:0.047172)100:0.422911,Query0362_KY271402:0.470092):0.008553,Que
ry0016_MF975720:0.478645)90:0.138935,(Query0364_KY271401:0.566654,Quer
y0153_KU927493:0.566654)78:0.050926):0.01572,(((((((JQ182732_Siphoviri
dae_Hk97virus:0.121761,JQ086374_Siphoviridae_Hk97virus:0.121761):0.022
677,JQ086372_Siphoviridae_Hk97virus:0.144438):0.037753,JQ086369_Siphov
iridae_Hk97virus:0.182191):0.01058,AF069529_Siphoviridae_Hk97virus:0.1
92771):0.023766,((HM173637_Siphoviridae_Hk97virus:0.157956,JQ086373_Si

phoviridae_Hk97virus:0.157956):0.022406,JQ086377_Siphoviridae_Hk97viru
s:0.180362):0.036176)76:0.030685,JQ182727_Siphoviridae_Hk97virus:0.247
223)90:0.079303,(JQ182726_Siphoviridae_Hk97virus:0.220515,AF069308_Sip
hoviridae_Hk97virus:0.220515)90:0.106011)100:0.306774):0.006052,((AF44
7491_Siphoviridae_E125virus:0.166812,CP000625_Siphoviridae_E125virus:0
.166812):0.032134,AY453853_Siphoviridae_E125virus:0.198946)100:0.44040
6):0.014365,(Query0310_KR072689:0.541892,Query0246_MF695815:0.541892)8
6:0.111825):0.048739,(Query0168_KT724718:0.609349,Query0101_KX815270:0
.609349):0.093106):0.010947,(HQ711985_Siphoviridae_D3virus:0.146743,AF
165214_Siphoviridae_D3virus:0.146743)100:0.566659):0.062614):0.066314,
((((((Query0353_KY860935:0.182649,Query0108_KX017520:0.182649)100:0.18
5093,Query0403_MF158038:0.367742)88:0.047298,JF974294_Siphoviridae_Pis
4avirus:0.41504)98:0.173106,(Query0357_KY271395:0.287664,Query0355_KY2
71396:0.287664)100:0.300482):0.163875,((Query0491_MG675557:0.654025,Qu
ery0136_KX982260:0.654025):0.047591,Query0017_KF591601:0.701616):0.050
404):0.077939,(Query0541_JF314845:0.72125,Query0416_JN175269:0.72125):
0.108709):0.012371):0.052053,(((((((((((((((Query0423_MG646668:0.053
42,KR270151_Siphoviridae_Jerseyvirus:0.05342):0.009469,JQ007353_Siphov
iridae_Jerseyvirus:0.062889):0.013012,(EF177456_Siphoviridae_Jerseyvir
us:0.055258,HE775250_Siphoviridae_Jerseyvirus:0.055258):0.020643):0.01
1974,(Query0096_KY379853:0.074935,KC139511_Siphoviridae_Jerseyvirus:0.
074935):0.012941):0.01386,AY730274_Siphoviridae_Jerseyvirus:0.101736):
0.003887,JX297445_Siphoviridae_Jerseyvirus:0.105623):0.01676,(JX202565
_Siphoviridae_Jerseyvirus:0.090836,KF562865_Siphoviridae_Jerseyvirus:0
.090836):0.031547):0.028381,KF148055_Siphoviridae_Jerseyvirus:0.150763
):0.001925,(Query0267_KY176369:0.102411,KF562864_Siphoviridae_Jerseyvi
rus:0.102411):0.050277):0.014684,KM272358_Siphoviridae_Jerseyvirus:0.1
67372)98:0.081488,((GU196278_Siphoviridae_K1gvirus:0.101062,GU196277_S
iphoviridae_K1gvirus:0.101062)88:0.056447,(GU196280_Siphoviridae_K1gvi
rus:0.029697,GU196279_Siphoviridae_K1gvirus:0.029697)100:0.127812)100:
0.091352)76:0.01538,(Query0135_MF158037:0.151317,KC139518_Siphoviridae
_Sp31virus:0.151317)98:0.112924)78:0.098992,(Query0134_MF158036:0.2733
96,KC832325_Siphoviridae_Jerseyvirus:0.273396)80:0.089836)100:0.221809
,((((((FJ750948_Siphoviridae_Hk578virus:0.060663,JX865427_Siphovirida
e_Hk578virus:0.060663):0.006101,KM233151_Siphoviridae_Hk578virus:0.066
764):0.013023,JQ086375_Siphoviridae_Hk578virus:0.079786)62:0.027171,(G
Q502199_Siphoviridae_Hk578virus:0.060875,JN984867_Siphoviridae_Hk578vi
rus:0.060875)96:0.046083):0.014034,KM896878_Siphoviridae_Hk578virus:0.
120991)96:0.068173,Query0422_MG065648:0.189164)100:0.128,KF772233_Siph
oviridae_Eiauvirus:0.317164)100:0.267877)90:0.218442,(((((Query0094_KF
302036:0.379946,Query0079_KX365748:0.379946)100:0.201261,Query0231_KY6
53118:0.581207)72:0.081643,Query0258_KU708004:0.66285):0.06486,(Query0
338_MG674163:0.332679,Query0009_MG641885:0.332679)100:0.395031):0.0197
19,Query0359_KY271399:0.74743):0.056054):0.030768,((((((JN871591_Siph
oviridae_Chivirus:0.026472,KC677662_Siphoviridae_Chivirus:0.026472)72:
0.012965,KC139512_Siphoviridae_Chivirus:0.039436)76:0.026957,JX094499_
Siphoviridae_Chivirus:0.066393):0.013318,KC139519_Siphoviridae_Chiviru
s:0.079712)100:0.280949,((((Query0526_MF415410:0.0,Query0410_MF476924:
0.0)100:0.0,Query0564_MF415413:0.0)100:1.1e-
05,Query0562_MF415411:1.1e-
05)100:0.005312,Query0382_MG835858:0.005322)100:0.355339)84:0.20189,(Q
uery0409_MF476925:0.445918,Query0218_MF959998:0.445918)80:0.116633)100

:0.201732,((((Query0128_MF074189:0.557822,Query0092_MF150911:0.557822)
90:0.080487,Query0169_KX879602:0.638309):0.044253,(Query0402_KX196154:
0.456445,Query0090_KY622015:0.456445)100:0.226117):0.040428,((((Query0
147_KY653127:0.476635,Query0104_CP011103:0.476635)64:0.045711,Query031
3_MG784342:0.522346)62:0.046425,Query0331_MF172979:0.568772):0.048731,
Query0360_KY203335:0.617503):0.105487):0.041293):0.069968):0.031978,((
((((((KP719134_Siphoviridae_Nonagvirus:0.076286,KJ419279_Siphoviridae
_Nonagvirus:0.076286)88:0.07067,(KP719133_Siphoviridae_Nonagvirus:0.05
6936,KP719132_Siphoviridae_Nonagvirus:0.056936)96:0.090019)100:0.21950
9,(KM236243_Siphoviridae_Seuratvirus:0.109761,KP064094_Siphoviridae_Se
uratvirus:0.109761)100:0.256703)100:0.182642,(JQ067084_Siphoviridae_Np
1virus:0.03151,KX129925_Siphoviridae_Np1virus:0.03151)100:0.517596)80:
0.10104,(((((JQ307387_Siphoviridae_Septima3virus:0.021314,HG962376_Sip
hoviridae_Septima3virus:0.021314):0.005578,NC_007806_Siphoviridae_Sept
ima3virus:0.026892)100:0.111035,JF939047_Siphoviridae_Septima3virus:0.
137927)100:0.207378,(KR935213_Siphoviridae_Titanvirus:0.020829,KR93521
5_Siphoviridae_Titanvirus:0.020829)100:0.324477)62:0.033473,Query0034_
KU197014:0.378778)100:0.271368)72:0.041408,JX483876_Myoviridae_Rheph4v
irus:0.691554)70:0.105796,(((((LN610584_Siphoviridae_Ab18virus:0.05769
3,LN610577_Siphoviridae_Ab18virus:0.057693)98:0.072284,JQ067087_Siphov
iridae_Ab18virus:0.129977)100:0.339814,((JX131330_Siphoviridae_Yuaviru
s:0.047934,KC758116_Siphoviridae_Yuavirus:0.047934)100:0.023866,KT7348
62_Siphoviridae_Yuavirus:0.0718)100:0.397991)98:0.121455,(JQ067093_Sip
hoviridae_Pamx74virus:0.199394,JQ067089_Siphoviridae_Pamx74virus:0.199
394)100:0.391852)66:0.138526,((KP202969_Siphoviridae_Jwxvirus:0.093135
,KP202970_Siphoviridae_Jwxvirus:0.093135)100:0.550655,(KT266805_Siphov
iridae_Rdjlvirus:0.02389,HM151342_Siphoviridae_Rdjlvirus:0.02389)100:0
.6199):0.085982)80:0.067577):0.03371,(JQ692107_Siphoviridae_Ssp2virus:
0.049624,JX556418_Siphoviridae_Ssp2virus:0.049624)100:0.781435):0.0232
03,(((KC139649_Podoviridae_Nonanavirus:0.233179,KJ802832_Podoviridae_N
onanavirus:0.233179)100:0.202947,Query0498_KY742649:0.436127)100:0.343
477,(AP013057_Myoviridae_Msw3virus:0.129519,AB767244_Myoviridae_Msw3vi
rus:0.129519)100:0.650085):0.074659):0.011967):0.028153):0.029741,((((
((Query0467_KY707339:0.593013,Query0061_KT588075:0.593013)74:0.113939,
(Query0509_KP836356:0.242262,Query0508_KP836355:0.242262)100:0.464689)
:0.101306,((Query0130_MF370965:0.474184,Query0014_KY379511:0.474184)70
:0.069805,Query0077_KX912252:0.543989)88:0.264269):0.014352,((((JX9765
49_Myoviridae_Ap22virus:0.185483,HM368260_Myoviridae_Ap22virus:0.18548
3)86:0.092878,KJ817802_Myoviridae_Ap22virus:0.27836)66:0.029375,HE8062
80_Myoviridae_Ap22virus:0.307736)100:0.366701,(Query0030_KY290953:0.00
1774,Query0006_KY290954:0.001774)100:0.672663)72:0.148173):0.056254,((
Query0336_KY296501:3.1e-05,Query0335_KY296500:3.1e-
05)100:0.632564,Query0156_KX656785:0.632595)96:0.246269):0.03087,((Que
ry0197_MG640035:0.713167,Query0027_MG065678:0.713167):0.151457,(Query0
361_KT716399:0.708738,Query0062_KT588074:0.708738):0.155885):0.04511):
0.014389):0.011026,((((((DQ873690_Siphoviridae_D3112virus:0.029689,JN
808773_Siphoviridae_D3112virus:0.029689)98:0.02718,EU272037_Siphovirid
ae_D3112virus:0.056869)92:0.06866,DQ631426_Siphoviridae_D3112virus:0.1
25529)100:0.120832,(((EU272036_Siphoviridae_D3112virus:0.05027,AY39400
5_Siphoviridae_D3112virus:0.05027)82:0.023958,HE584812_Siphoviridae_D3
112virus:0.074228)78:0.032405,HM624080_Siphoviridae_D3112virus:0.10663
2)100:0.139728)100:0.3691,(Query0125_AB916497:0.49615,Query0015_MF9757

21:0.49615)86:0.11931)70:0.10256,KP010268_Myoviridae_Muvirus:0.71802)6
6:0.083372,(Query0570_MG711460:0.735291,Query0150_KX669658:0.735291):0
.066102):0.133757):0.008581,((((((((Query0408_KY290947:0.083045,Query
0406_KY290949:0.083045)66:0.026707,Query0010_KY290950:0.109752)84:0.04
683,(Query0145_KX455876:0.123153,Query0012_KY290952:0.123153)86:0.0334
29)100:0.485834,(Query0260_KY038360:0.612769,Query0172_KX905163:0.6127
69)60:0.029647):0.043068,(((Query0569_MG711461:0.480803,Query0567_MG71
1467:0.480803):0.048371,Query0572_MG711462:0.529174):0.035107,(Query05
68_MG711466:0.437395,Query0303_KR131710:0.437395)82:0.126886)74:0.1212
02):0.074229,Query0456_MF614628:0.759712):0.0308,(((FN297812_Myovirida
e_Vhmlvirus:0.09287,JX556417_Myoviridae_Vhmlvirus:0.09287)84:0.040481,
AY133112_Myoviridae_Vhmlvirus:0.133351)100:0.597184,(KM360178_Myovirid
ae_Cvm10virus:0.14301,GU903191_Myoviridae_Cvm10virus:0.14301)100:0.587
525):0.059977):0.046919,((((JQ086376_Siphoviridae_Lambdavirus:0.171011
,JQ182735_Siphoviridae_Lambdavirus:0.171011)80:0.090173,EU078592_Sipho
viridae_Lambdavirus:0.261184)100:0.175376,Query0520_CP025712:0.43656)1
00:0.305053,(Query0219_MF959999:0.611019,Query0063_KT898133:0.611019)8
8:0.130595):0.095818):0.053208,((((Query0358_KY271398:0.481342,Query03
56_KY271397:0.481342)64:0.104058,Query0013_HQ110083:0.5854):0.173182,(
Query0479_KY045851:0.474343,Query0441_KX774374:0.474343)100:0.284239):
0.087601,AF155037_Corticoviridae_Corticovirus:0.846183):0.044456):0.05
309):0.007464,((((((KC821619_Siphoviridae_Cba181virus:0.146079,KC82161
7_Siphoviridae_Cba181virus:0.146079):0.024421,KC821613_Siphoviridae_Cb
a181virus:0.1705)100:0.648928,(JQ823122_Siphoviridae_P12024virus:0.071
126,JQ823123_Siphoviridae_P12024virus:0.071126)100:0.748302):0.018631,
(((Query0261_KU234533:0.266896,Query0236_KU234532:0.266896)100:0.47133
6,Query0089_MF663786:0.738232):0.014355,Query0566_MG711464:0.752587):0
.085472):0.035159,((JN939331_Podoviridae_Prtbvirus:0.084353,JN939332_P
odoviridae_Prtbvirus:0.084353)100:0.738046,Query0466_KT820175:0.822398
):0.05082):0.040652,((((((Query0438_KU599883:0.0,Query0437_KU599880:0.
0)100:0.0,Query0463_KT876725:0.0)100:0.000191,Query0436_KU599881:0.000
191)90:0.000186,Query0439_KU599882:0.000376)100:0.006703,(Query0433_KU
599884:0.000256,Query0432_KU599885:0.000256)100:0.006823)100:0.801388,
Query0527_KY421186:0.808467):0.105403):0.037323):0.002518):0.008264,((
(((((((((((Query0378_KX119177:0.022676,Query0325_KX119198:0.022676):
0.00448,Query0178_KX119205:0.027156):0.010593,(Query0326_KX119199:0.02
4266,Query0180_KX119203:0.024266)64:0.013483):0.001489,Query0377_KX119
176:0.039238)92:0.035369,Query0177_KX119204:0.074607):0.026385,((Query
0182_KX119201:3e-06,Query0181_KX119200:3e-
06)100:0.077819,Query0179_KX119202:0.077822):0.02317):0.010895,Query03
76_KX119175:0.111887):0.007259,((AB731695_Podoviridae_Una961virus:0.04
2729,AB647160_Podoviridae_Una961virus:0.042729)82:0.022182,JQ617284_Po
doviridae_Una961virus:0.064911)92:0.054236):0.017746,(Query0375_KX1191
74:0.006838,Query0176_KX119206:0.006838)100:0.130054):0.016669,Query04
93_KX119189:0.153561):0.045732,((((((Query0494_KX119188:0.020265,Query
0317_KX119192:0.020265)92:0.023498,Query0323_KX119194:0.043763):0.0142
22,Query0320_KX119191:0.057985)74:0.042883,Query0319_KX119190:0.100867
)62:0.032553,Query0324_KX119195:0.13342):0.024731,(Query0322_KX119197:
0.11551,Query0321_KX119196:0.11551):0.04264):0.041143):0.047702,Query0
318_KX119193:0.246996)100:0.621403,(((((Query0401_KY653128:0.421288,Qu
ery0379_KY653121:0.421288)98:0.194287,Query0415_L06183:0.615575)98:0.1
7007,Query0363_KY271400:0.785645):0.053654,(Query0434_KU599887:0.07002

1,Query0431_KU599889:0.070021)100:0.769277):0.016285,L13696_Plasmaviri
dae_Plasmavirus:0.855583):0.012815):0.070561,(GQ403789_Sphaerolipoviri
dae_Gammasphaerolipovirus:0.343553,AB063393_Sphaerolipoviridae_Gammasp
haerolipovirus:0.343553)100:0.595406):0.002851,(((((((EU311208_Podovi
ridae_Nona33virus:0.061031,AF125520_Podoviridae_Nona33virus:0.061031)7
2:0.044181,HM208303_Podoviridae_Nona33virus:0.105212):0.022321,AP00515
4_Podoviridae_Nona33virus:0.127533)98:0.059157,KP682381_Podoviridae_No
na33virus:0.18669)94:0.055309,(KJ603229_Podoviridae_Pocjvirus:0.045107
,KF766125_Podoviridae_Pocjvirus:0.045107)100:0.196892)66:0.006967,((((
Query0440_HG803182:0.042213,KR781488_Podoviridae_Tl2011virus:0.042213)
92:0.02403,JQ011318_Podoviridae_Tl2011virus:0.066243)84:0.020791,KF971
864_Podoviridae_Tl2011virus:0.087034)82:0.026633,KP682371_Podoviridae_
Tl2011virus:0.113667)100:0.135299)100:0.581709,((((JX104231_Podovirida
e_Bcep22virus:0.166307,AY349011_Podoviridae_Bcep22virus:0.166307)78:0.
041908,(JN662425_Podoviridae_Bcep22virus:0.069274,FJ937737_Podoviridae
_Bcep22virus:0.069274)100:0.138941)100:0.50547,((Query0385_KY417925:0.
23049,Query0216_MF403009:0.23049)100:0.438445,Query0060_KT898134:0.668
935):0.044751):0.100535,(KC262634_Podoviridae_F116virus:0.13597,AY6258
98_Podoviridae_F116virus:0.13597)100:0.678251):0.016455):0.09329,((((Q
uery0522_CP025713:0.524592,JN391180_Podoviridae_Epsilon15virus:0.52459
2)96:0.252732,(Query0540_DQ785801:0.05591,Query0146_AP009146:0.05591)1
00:0.721414):0.033407,(Query0380_MG197996:0.672564,LT714109_Podovirida
e_P22virus:0.672564)70:0.138166):0.095222,(Query0383_KY653122:0.712206
,Query0257_KU708005:0.712206)64:0.193748):0.018013):0.017844):0.020166
):0.008147,((((((GU815091_Myoviridae_Pbunavirus:0.024985,KR054033_Myov
iridae_Pbunavirus:0.024985)90:0.011302,AB560486_Myoviridae_Pbunavirus:
0.036287)100:0.032684,(KR054030_Myoviridae_Pbunavirus:0.045906,LN61058
9_Myoviridae_Pbunavirus:0.045906)94:0.023065)100:0.46099,Query0284_MG0
18926:0.52996)100:0.360773,(((((Query0341_KY883647:0.008756,AY510084_P
odoviridae_Vp5virus:0.008756)80:0.011611,AY505112_Podoviridae_Vp5virus
:0.020367)80:0.009348,Query0195_KY883656:0.029715)72:0.01449,JF712866_
Podoviridae_Vp5virus:0.044205)68:0.008952,Query0444_KY883642:0.053157)
100:0.837577):0.031166,(LC102729_Podoviridae_Kpp25virus:0.022426,AB910
393_Podoviridae_Kpp25virus:0.022426)100:0.899474):0.048223):0.006253,(
((((((((((((((((((((((((((Query0086_KX828711:0.034527,KM501444_My
oviridae_T4virus:0.034527):0.00444,AP011113_Myoviridae_T4virus:0.03896
7):0.002076,KF925357_Myoviridae_T4virus:0.041043):0.004937,Query0469_M
F001360:0.04598):0.010084,KJ668714_Myoviridae_T4virus:0.056064):0.0057
67,(((HM035025_Myoviridae_T4virus:0.026679,JN202312_Myoviridae_T4virus
:0.026679)94:0.01915,Query0138_MF327007:0.045828):0.005811,HE956711_My
oviridae_T4virus:0.05164):0.010191):0.01341,(((KF208315_Myoviridae_T4v
irus:0.036015,JX128259_Myoviridae_T4virus:0.036015)92:0.016831,JN98684
6_Myoviridae_T4virus:0.052847):0.002197,KM606994_Myoviridae_T4virus:0.
055044)76:0.020196)100:0.031964,((((Query0143_MF327008:0.024109,Query0
142_MF327009:0.024109)100:0.020435,Query0297_MF158045:0.044544)84:0.01
0135,Query0296_MF158046:0.054678)100:0.030906,Query0074_MF001354:0.085
584)100:0.02162)100:0.058672,(((Query0170_MF044457:0.050083,KF582788_M
yoviridae_Rb69virus:0.050083):0.008413,JX536493_Myoviridae_Rb69virus:0
.058496)60:0.020691,KM407600_Myoviridae_Rb69virus:0.079188)100:0.08668
9)96:0.025439,(((((KP007359_Myoviridae_Js98virus:0.047234,EU863409_Myo
viridae_Js98virus:0.047234)72:0.008617,KT176190_Myoviridae_Js98virus:0
.055851)64:0.00697,HM071924_Myoviridae_Js98virus:0.062822)100:0.06269,

HQ829472_Myoviridae_Js98virus:0.125511)60:0.016337,(((GQ981382_Myoviri
dae_Sp18virus:0.056946,KP007361_Myoviridae_Sp18virus:0.056946)64:0.013
845,KP007360_Myoviridae_Sp18virus:0.070791)60:0.028606,(HM563683_Myovi
ridae_Sp18virus:0.076141,KP007362_Myoviridae_Sp18virus:0.076141):0.023
256)96:0.04245)100:0.049467)90:0.013863,(((((KM236240_Myoviridae_Moonv
irus:0.046846,KT001915_Myoviridae_Moonvirus:0.046846)100:0.07736,(GU32
3318_Myoviridae_Cc31virus:0.033948,KJ101592_Myoviridae_Cc31virus:0.033
948)100:0.090258)96:0.017124,((Query0266_MF036690:0.000168,Query0264_M
F036691:0.000168)100:0.023247,Query0420_MF036692:0.023415)100:0.117915
)94:0.023269,(JX181825_Myoviridae_S16virus:0.066199,HQ331142_Myovirida
e_S16virus:0.066199)100:0.0984)74:0.010194,((Query0183_MG751100:0.0199
5,KT239446_Myoviridae_Jd18virus:0.01995)100:0.079236,KR269720_Myovirid
ae_Jd18virus:0.099186)100:0.075607)94:0.030384)98:0.052146,(KP202158_M
yoviridae_Tg1virus:0.078033,HE956709_Myoviridae_Tg1virus:0.078033)100:
0.179292)100:0.03937,(Query0139_MF327006:0.104701,EU863408_Myoviridae_
Rb49virus:0.104701)100:0.191993)100:0.070191,((((KT001919_Myoviridae_K
p15virus:0.011493,KT001918_Myoviridae_Kp15virus:0.011493)100:0.008184,
HQ918180_Myoviridae_Kp15virus:0.019676)68:0.003669,GU295964_Myoviridae
_Kp15virus:0.023346)76:0.012969,KT321315_Myoviridae_Kp15virus:0.036315
)100:0.33057)78:0.013942,((((Query0026_KY290951:0.006584,Query0008_KY2
90956:0.006584):0.002055,(Query0405_KY290948:2e-
06,AY375531_Myoviridae_Biquartavirus:2e-
06)100:0.008637):0.000974,(Query0007_KY290957:0.005494,Query0003_KY290
958:0.005494)70:0.004119)100:0.126373,((((JN377895_Myoviridae_Secunda5
virus:0.06208,DQ529280_Myoviridae_Secunda5virus:0.06208):0.005381,JX30
6041_Myoviridae_Secunda5virus:0.067461):0.004398,HM452125_Myoviridae_S
ecunda5virus:0.07186)92:0.022086,JN377894_Myoviridae_Secunda5virus:0.0
93945)100:0.042041)100:0.244841)100:0.0522,((Query0373_KU160494:0.2747
12,Query0005_KY290955:0.274712)100:0.107409,KP671755_Myoviridae_Schizo
t4virus:0.382121)100:0.050906)100:0.055334,Query0501_KU255730:0.488361
)100:0.176491,((((((((((Query0451_KU686201:0.00137,Query0450_KU68620
0:0.00137)100:0.004858,Query0449_KU686202:0.006229)100:0.08518,((Query
0262_KU686199:0.002816,Query0087_KU686198:0.002816)82:0.001441,Query00
85_KU686197:0.004257)100:0.087152)80:0.015265,((Query0455_KU686209:0.0
00441,Query0454_KU686208:0.000441)62:0.000133,Query0446_KU686207:0.000
574)100:0.1061)82:0.023612,((Query0447_KU686204:9.7e-
05,Query0445_KU686206:9.7e-
05)100:0.000504,Query0448_KU686205:0.0006)100:0.129685)100:0.050271,(Q
uery0411_KU686213:0.038755,Query0088_KU686212:0.038755)100:0.141802)10
0:0.070207,Query0091_MG198570:0.250764)100:0.081491,Query0274_KU682439
:0.332254)96:0.053784,Query0057_KY979132:0.386038)100:0.096888,Query02
17_MF403007:0.482927)76:0.020047,(Query0495_KU873925:0.387157,Query023
7_KY914485:0.387157)100:0.115817)92:0.096914,((Query0198_KY630187:0.46
6049,Query0044_AP018399:0.466049)94:0.101248,Query0215_MF403008:0.5672
98):0.032591)88:0.064965)74:0.016615,((KR052480_Myoviridae_M12virus:0.
0636,KF381361_Myoviridae_M12virus:0.0636)90:0.017576,KR052482_Myovirid
ae_M12virus:0.081175)100:0.600293)76:0.014094,((((((((JN126049_Ackerm
annviridae_Cba120virus:0.045975,KX438380_Ackermannviridae_Cba120virus:
0.045975)88:0.018294,HQ259103_Ackermannviridae_Cba120virus:0.064268)66
:0.016566,KR296692_Ackermannviridae_Cba120virus:0.080834):0.009748,JX1
81828_Ackermannviridae_Vi1virus:0.090582):0.009908,((KF669654_Ackerman
nviridae_Vi1virus:0.024569,KF669653_Ackermannviridae_Vi1virus:0.024569

)100:0.053479,FQ312032_Ackermannviridae_Vi1virus:0.078048)82:0.022441)
:0.005478,(((JN673056_Ackermannviridae_Cba120virus:0.053638,JN593240_A
ckermannviridae_Cba120virus:0.053638)78:0.034801,(Query0280_KY000003:0
.041419,KX245012_Ackermannviridae_Cba120virus:0.041419)98:0.047019):0.
011255,KP797973_Ackermannviridae_Cba120virus:0.099694):0.006274):0.010
509,((JX128257_Ackermannviridae_Vi1virus:0.091545,KJ174318_Ackermannvi
ridae_Cba120virus:0.091545):0.018368,KJ174317_Ackermannviridae_Vi1viru
s:0.109914):0.006563)100:0.084035,(((KX147096_Ackermannviridae_:0.1284
57,AB797215_Ackermannviridae_:0.128457)92:0.033823,(FJ373894_Ackermann
viridae_Ag3virus:0.112077,JX181829_Ackermannviridae_Ag3virus:0.112077)
94:0.050204):0.018506,(KJ716335_Ackermannviridae_Limestonevirus:0.0571
33,HE600015_Ackermannviridae_Limestonevirus:0.057133)100:0.123654)82:0
.019725)100:0.10442,((Query0244_MF285619:0.014193,JX878496_Ackermannvi
ridae_:0.014193)100:0.174169,KP037007_Ackermannviridae_:0.188363)100:0
.116568)100:0.390631)94:0.067322,((((GU296433_Myoviridae_Cp8virus:0.03
2578,JX569801_Myoviridae_Cp8virus:0.032578)100:0.023637,Query0278_KX22
9736:0.056215):0.004914,((JN132397_Myoviridae_Cp8virus:0.013375,FR8234
50_Myoviridae_Cp8virus:0.013375)100:0.023056,Query0329_KX879627:0.0364
32)96:0.024697)100:0.620154,(((FN667789_Myoviridae_Cp220virus:0.069646
,FN667788_Myoviridae_Cp220virus:0.069646)100:0.100865,HE815464_Myoviri
dae_Cp220virus:0.170512)100:0.41669,HM246720_Myoviridae_Cp220virus:0.5
87201)92:0.094081)96:0.081602)90:0.060949,((((((((((((((((Query0165_
MG065662:0.009253,Query0018_MG065672:0.009253)88:0.012327,Query0024_MG
065674:0.02158)72:0.01127,Query0082_MG065690:0.03285):0.013332,(Query0
426_MG065644:0.027163,Query0109_MG065677:0.027163):0.019019)80:0.00762
9,(((Query0247_MG065658:0.026885,DQ832317_Myoviridae_V5virus:0.026885)
96:0.012242,(Query0497_MG065647:0.019217,Query0281_MG065646:0.019217)9
2:0.01991):0.002908,KC690136_Myoviridae_V5virus:0.042035)80:0.011776)6
8:0.019127,(JQ031132_Myoviridae_V5virus:0.037845,KJ190158_Myoviridae_V
5virus:0.037845)94:0.035093):0.005063,Query0162_MG065661:0.078001):0.0
01779,((((Query0478_MG065654:3.8e-05,Query0161_MG065666:3.8e-
05)100:0.035537,Query0507_MG065635:0.035575)78:0.016578,Query0208_MG06
5682:0.052152):0.00636,Query0480_MG065655:0.058512):0.021268)100:0.282
034,Query0248_MG065659:0.361814)66:0.021252,((Query0485_MG065653:0.169
643,Query0484_MG065652:0.169643)98:0.098794,Query0022_MG065676:0.26843
6)100:0.11463)90:0.07454,(((JX181824_Myoviridae_Se1virus:0.041904,GU07
0616_Myoviridae_Se1virus:0.041904)100:0.105937,(KF550303_Myoviridae_Se
1virus:0.041103,JN882284_Myoviridae_Se1virus:0.041103)100:0.106737)100
:0.113414,Query0069_KY652726:0.261254)100:0.196352):0.098712,(((((KC95
4774_Myoviridae_Cr3virus:0.018996,JQ691612_Myoviridae_Cr3virus:0.01899
6)100:0.086219,KT353109_Myoviridae_Cr3virus:0.105215)100:0.022832,JQ69
1611_Myoviridae_Cr3virus:0.128047)100:0.067221,(Query0289_MF979563:0.0
07774,Query0288_MF979560:0.007774)100:0.187493)78:0.058747,JQ015307_My
oviridae_Cr3virus:0.254014)100:0.302305)64:0.045182,Query0123_KR296694
:0.601501)82:0.049505,(((((((Query0304_KY883635:0.020073,Query0070_KY
883636:0.020073)98:0.020513,Query0071_KY883637:0.040585)94:0.032609,((
Query0066_KY883639:0.015225,Query0065_KY883638:0.015225)74:0.004922,Qu
ery0340_KY883640:0.020147)98:0.053048)94:0.030814,Query0072_KY883634:0
.104009)100:0.347352,Query0099_KX507046:0.45136):0.027116,((Query0184_
MF988720:0.324174,Query0152_KX822733:0.324174)100:0.130534,(Query0129_
MF370964:0.263978,Query0093_KF302034:0.263978)100:0.190729):0.023769)1
00:0.065586,(((Query0283_MG065645:0.024565,Query0251_MG065656:0.024565

)100:0.025978,(Query0282_MG065639:0.015048,Query0206_MG065689:0.015048
)100:0.035495)100:0.18349,Query0253_MG065650:0.234033)100:0.31003)90:0
.08244,Query0214_MG065684:0.626503)70:0.024503)62:0.050728,((((((((((
(Query0265_MG646669:0.002411,Query0105_MG646671:0.002411)100:0.021365,
Query0081_MG646670:0.023777)100:0.032254,Query0059_MG646672:0.056031):
0.00069,JN225449_Myoviridae_Felixo1virus:0.05672):0.003751,KF055347_My
oviridae_Felixo1virus:0.060471):0.001374,KP143762_Myoviridae_Felixo1vi
rus:0.061845):0.015047,JX560968_Myoviridae_Felixo1virus:0.076892)64:0.
009904,(((((EU877232_Myoviridae_Felixo1virus:0.035374,KP869100_Myoviri
dae_Felixo1virus:0.035374)68:0.009166,KR014248_Myoviridae_Felixo1virus
:0.04454):0.004662,KM092515_Myoviridae_Felixo1virus:0.049202)60:0.0069
48,KM657822_Myoviridae_Felixo1virus:0.05615)72:0.010988,KP010413_Myovi
ridae_Felixo1virus:0.067138)64:0.019658)84:0.026652,((((((Query0141_M
F327004:0.008507,Query0140_MF327005:0.008507)78:0.00895,Query0301_MF15
8041:0.017456)94:0.020707,(Query0346_MF327003:0.019322,Query0299_MF158
043:0.019322)96:0.018842):0.010623,Query0298_MF158044:0.048787)92:0.01
2853,KM236239_Myoviridae_Mooglevirus:0.061639)70:0.012027,KT363872_Myo
viridae_Mooglevirus:0.073666):0.024318,((Query0302_MF158040:0.062194,K
T454806_Myoviridae_Suspvirus:0.062194):0.00816,KT454805_Myoviridae_Sus
pvirus:0.070355)60:0.027629)78:0.015465)80:0.02386,Query0159_MG065664:
0.137308)100:0.183896,Query0167_MG065668:0.321204)68:0.018665,(HQ72826
3_Myoviridae_Ea214virus:0.044862,EU710883_Myoviridae_Ea214virus:0.0448
62)100:0.295007)100:0.361864)90:0.045619,((((((HE983845_Myoviridae_Pak
punavirus:0.019823,LN610572_Myoviridae_Pakpunavirus:0.019823)100:0.016
477,HQ832595_Myoviridae_Pakpunavirus:0.0363)100:0.026467,(Query0476_KR
052143:0.024923,GU988610_Myoviridae_Pakpunavirus:0.024923)100:0.037844
)86:0.025756,(KC862300_Myoviridae_Pakpunavirus:0.079645,KC862297_Myovi
ridae_Pakpunavirus:0.079645)78:0.008879)100:0.313735,Query0489_MG01893
0:0.402258)100:0.081938,((KC862299_Myoviridae_Kpp10virus:0.023881,AB47
2900_Myoviridae_Kpp10virus:0.023881)100:0.042436,LN610573_Myoviridae_K
pp10virus:0.066317)100:0.417879)100:0.263157)82:0.038728,((((((Query0
428_MG065642:0.044739,KX017521_Siphoviridae_T5virus:0.044739)98:0.0294
46,KM236244_Siphoviridae_T5virus:0.074185)100:0.099241,KM979354_Siphov
iridae_T5virus:0.173426):0.000544,((LN887948_Siphoviridae_T5virus:0.11
4644,KJ190157_Siphoviridae_T5virus:0.114644)68:0.025535,Query0084_MG06
5692:0.140179)90:0.033792)96:0.040261,KP143763_Siphoviridae_T5virus:0.
214231)100:0.116183,Query0285_MF979564:0.330415)92:0.189553,Query0194_
KY883654:0.519968)100:0.266113)70:0.023118,Query0122_KR296695:0.809198
):0.014635):0.027994,(((((Query0223_LC168164:0.569905,Query0103_MG0189
27:0.569905)96:0.078035,HQ634192_Siphoviridae_Cbastvirus:0.64794)88:0.
120331,(Query0521_KX925554:0.702238,Query0132_KU665491:0.702238):0.066
033):0.027756,(((Query0475_MF044458:0.468395,Query0175_KY709296:0.4683
95)96:0.165128,Query0001_KJ936628:0.633523)66:0.04372,Query0127_MF7880
75:0.677243)76:0.118784):0.031849,(((KP696447_Siphoviridae_Slashvirus:
0.05588,KF669661_Siphoviridae_Slashvirus:0.05588)68:0.020579,KF669663_
Siphoviridae_Slashvirus:0.076459)88:0.055211,KP696448_Siphoviridae_Sla
shvirus:0.13167)100:0.696206):0.023952):0.015513,(((((JX100812_Siphovi
ridae_Phicbkvirus:0.017991,JX100811_Siphoviridae_Phicbkvirus:0.017991)
92:0.008323,JX100813_Siphoviridae_Phicbkvirus:0.026314)100:0.028146,JX
100809_Siphoviridae_Phicbkvirus:0.05446)100:0.087595,JX100814_Siphovir
idae_Phicbkvirus:0.142055)100:0.625859,(Query0221_KY940711:0.635392,Qu
ery0048_KY629563:0.635392)82:0.132523)76:0.099426):0.002304,(((((((((

KF208639_Myoviridae_B4virus:0.051199,KF669647_Myoviridae_B4virus:0.051
199)96:0.025429,(KJ489402_Myoviridae_B4virus:0.021039,KT307976_Myoviri
dae_B4virus:0.021039)100:0.05559)86:0.031159,(KF669662_Myoviridae_B4vi
rus:0.080419,JN790865_Myoviridae_B4virus:0.080419)84:0.027369)100:0.12
6313,(KJ489397_Myoviridae_Bastillevirus:0.049409,JF966203_Myoviridae_B
astillevirus:0.049409)100:0.184692)100:0.065636,(((((KT995480_Myovirid
ae_Bc431virus:0.083816,KJ451625_Myoviridae_Bc431virus:0.083816)66:0.01
5108,JX094431_Myoviridae_Bc431virus:0.098925)64:0.014614,KU577463_Myov
iridae_Bc431virus:0.113539)92:0.052024,(KJ676859_Myoviridae_Bc431virus
:0.128038,KJ081346_Myoviridae_Bc431virus:0.128038)70:0.037524)98:0.062
766,(KT224359_Myoviridae_Tsarbombavirus:0.067227,JN797797_Myoviridae_T
sarbombavirus:0.067227)100:0.161101)100:0.071409)100:0.066534,((((AP01
3029_Myoviridae_Nit1virus:0.10478,KF669652_Myoviridae_Nit1virus:0.1047
8)82:0.031524,(Query0483_MF422185:0.053076,AB930182_Myoviridae_Nit1vir
us:0.053076)100:0.083228)94:0.05765,Query0239_KY368640:0.193954)100:0.
089224,((KJ010547_Myoviridae_Agatevirus:0.097183,JX238501_Myoviridae_A
gatevirus:0.097183)74:0.013176,KM051843_Myoviridae_Agatevirus:0.110359
)100:0.172819)100:0.083093):0.021577,((((KJ489401_Myoviridae_Wphvirus:
0.027258,KJ489399_Myoviridae_Wphvirus:0.027258)72:0.007948,Query0477_K
X961632:0.035206)100:0.041408,JN654439_Myoviridae_Wphvirus:0.076614)70
:0.013548,HM144387_Myoviridae_Wphvirus:0.090162)100:0.297687)100:0.136
853,(((((((KM216423_Myoviridae_Kayvirus:0.052338,JQ686190_Myoviridae_
Kayvirus:0.052338)62:0.005799,Query0314_MG721208:0.058137)62:0.023038,
(KP027446_Myoviridae_Kayvirus:0.062386,KJ888149_Myoviridae_Kayvirus:0.
062386):0.018789):0.008562,JX878671_Myoviridae_Kayvirus:0.089737)66:0.
009084,((AB853331_Myoviridae_Kayvirus:0.045445,AB853330_Myoviridae_Kay
virus:0.045445)92:0.02925,AB903967_Myoviridae_Kayvirus:0.074696)86:0.0
24126)100:0.221962,(KF021268_Myoviridae_Sep1virus:0.15281,KP027447_Myo
viridae_Sep1virus:0.15281)100:0.167974)62:0.006121,(((JX194239_Myoviri
dae_Silviavirus:0.103159,JX846613_Myoviridae_Silviavirus:0.103159)76:0
.029136,KP881332_Myoviridae_Silviavirus:0.132295)100:0.149321,(Query03
52_KX171212:0.000247,Query0351_KX171213:0.000247)100:0.281369)94:0.045
289)100:0.14227,(Query0076_KY549443:0.41065,DQ004855_Myoviridae_P100vi
rus:0.41065)96:0.058525)82:0.055526)94:0.065987,((Query0011_MG765279:0
.165095,Query0000_MG765277:0.165095)100:0.276476,FJ822135_Myoviridae_U
nassigned:0.441571)96:0.149116)100:0.118927,(((KC595513_Myoviridae_Cp5
1virus:0.009946,KC595512_Myoviridae_Cp51virus:0.009946)100:0.031564,KF
554508_Myoviridae_Cp51virus:0.04151)100:0.386767,KF669649_Myoviridae_S
po1virus:0.428277)100:0.281338)100:0.160029):0.030164,(KC821632_Podovi
ridae_Cba41virus:0.020668,KC821609_Podoviridae_Cba41virus:0.020668)100
:0.87914):0.004647,(((((DQ657948_Alloherpesviridae_Cyprinivirus:0.2773
97,JQ815364_Alloherpesviridae_Cyprinivirus:0.277397)96:0.049277,JQ8153
63_Alloherpesviridae_Cyprinivirus:0.326673)100:0.344811,FJ940765_Alloh
erpesviridae_Cyprinivirus:0.671484)100:0.187285,KT995472_Nimaviridae_W
hispovirus:0.858769):0.027197,(AY509253_Malacoherpesviridae_Ostreaviru
s:0.676174,JX453331_Malacoherpesviridae_Aurivirus:0.676174)100:0.20979
2):0.01849):0.017725,((((((((Query0291_KY499642:0.330508,Query0245_MF2
85618:0.330508)70:0.026885,(Query0418_AP017925:0.018032,Query0417_AP01
7924:0.018032)100:0.339361)100:0.084733,Query0503_AP017972:0.442126)74
:0.073094,JX233784_Myoviridae_Phikzvirus:0.51522)72:0.069692,(AP014693
_Myoviridae_Rsl2virus:0.071791,AP014927_Myoviridae_Rsl2virus:0.071791)
100:0.513121):0.031425,AJ697969_Myoviridae_Elvirus:0.616337)100:0.0385

62,((((KU886223_Myoviridae_Agrican357virus:0.009405,KU886224_Myovirida
e_Agrican357virus:0.009405)88:0.004377,KU886222_Myoviridae_Agrican357v
irus:0.013782):0.002329,KF806589_Myoviridae_Agrican357virus:0.016111)8
4:0.004909,KU886225_Myoviridae_Agrican357virus:0.021019)100:0.63388)10
0:0.043249,(((JX316028_Myoviridae_Eah2virus:0.036582,KX397364_Myovirid
ae_Eah2virus:0.036582)100:0.075125,JN641803_Myoviridae_Ntreusvirus:0.1
11707)100:0.065936,(Query0481_KX397365:0.126029,KX397370_Myoviridae_Ma
chinavirus:0.126029)100:0.051614)100:0.520505)100:0.224033):0.010221,(
((((((((JX415535_Podoviridae_G7cvirus:0.061302,KC206276_Podoviridae_G
7cvirus:0.061302):0.001518,KJ135004_Podoviridae_G7cvirus:0.06282)78:0.
013087,(KF562340_Podoviridae_G7cvirus:0.050652,KF192075_Podoviridae_G7
cvirus:0.050652)92:0.025254)98:0.042089,HQ259105_Podoviridae_G7cvirus:
0.117996):0.002095,KF620435_Podoviridae_G7cvirus:0.120091)98:0.048774,
JX880034_Podoviridae_G7cvirus:0.168864)100:0.207336,(((KF787095_Podovi
ridae_Jwalphavirus:0.144674,KT321317_Podoviridae_Jwalphavirus:0.144674
)92:0.077231,Query0293_MG018928:0.221905)100:0.062958,(KX098389_Podovi
ridae_Ea92virus:0.006271,KF806588_Podoviridae_Ea92virus:0.006271)100:0
.278591)100:0.091338)100:0.142397,((KC139520_Podoviridae_Sp58virus:0.0
83552,KC139517_Podoviridae_Sp58virus:0.083552)100:0.075556,KM236242_Po
doviridae_Sp58virus:0.159108)100:0.359489)90:0.066709,(((((FN422399_Po
doviridae_Lit1virus:0.021898,HG962375_Podoviridae_Lit1virus:0.021898)8
0:0.008452,JX194238_Podoviridae_Lit1virus:0.030349)100:0.177388,(FN422
398_Podoviridae_Luz7virus:0.072733,LC064302_Podoviridae_Luz7virus:0.07
2733)100:0.135004)100:0.138315,Query0307_MG775260:0.346052)100:0.21321
5,KJ621082_Podoviridae_Dfl12virus:0.559267)60:0.026039)100:0.332131,((
(((Query0465_KT876726:0.000164,Query0435_KU599886:0.000164)100:0.00935
8,(Query0032_KU599878:0.000163,Query0031_KU599877:0.000163)100:0.00936
)100:0.124319,(Query0464_KT876724:0.000545,Query0033_KU599879:0.000545
)100:0.133296)100:0.675476,Query0443_MF497422:0.809317):0.055408,(Quer
y0075_KX365747:0.417,AB757800_Podoviridae_Kf1virus:0.417)100:0.447724)
:0.052713):0.014964):0.007936,((((((((((((((AY077832_Poxviridae_Cap
ripoxvirus:0.01467,AY077835_Poxviridae_Capripoxvirus:0.01467)100:0.014
537,AF325528_Poxviridae_Capripoxvirus:0.029207)100:0.115983,AF410153_P
oxviridae_Suipoxvirus:0.14519):0.008031,(AY689437_Poxviridae_Cervidpox
virus:0.014892,AY689436_Poxviridae_Cervidpoxvirus:0.014892)100:0.13832
9):0.008165,(AY386371_Poxviridae_Yatapoxvirus:0.073329,AJ293568_Poxvir
idae_Yatapoxvirus:0.073329)100:0.088057)96:0.042478,(AF170722_Poxvirid
ae_Leporipoxvirus:0.049928,AF170726_Poxviridae_Leporipoxvirus:0.049928
)100:0.153936)100:0.076715,(HQ647181_Poxviridae_Unassigned:0.112433,KY
094066_Poxviridae_Unassigned:0.112433)100:0.168146)100:0.073595,((((((
(DQ437594_Poxviridae_Orthopoxvirus:0.072546,AF438165_Poxviridae_Orthop
oxvirus:0.072546)62:0.011597,AF012825_Poxviridae_Orthopoxvirus:0.08414
3):0.007479,AF482758_Poxviridae_Orthopoxvirus:0.091622):0.009427,(X691
98_Poxviridae_Orthopoxvirus:0.087319,AF380138_Poxviridae_Orthopoxvirus
:0.087319):0.01373):0.007871,AY243312_Poxviridae_Orthopoxvirus:0.10892
):0.009752,((KU749311_Poxviridae_Orthopoxvirus:0.029153,KU749310_Poxvi
ridae_Orthopoxvirus:0.029153)100:0.041373,KP143769_Poxviridae_Orthopox
virus:0.070526)96:0.048146)100:0.123358,HQ849551_Poxviridae_Orthopoxvi
rus:0.24203)100:0.112143)100:0.083248,((((KM502564_Poxviridae_Parapoxv
irus:0.061786,AY386265_Poxviridae_Parapoxvirus:0.061786)82:0.010888,AY
386264_Poxviridae_Parapoxvirus:0.072674)100:0.022457,GQ329670_Poxvirid
ae_Parapoxvirus:0.095131)100:0.248173,((HE601899_Poxviridae_Unassigned

:0.268496,U60315_Poxviridae_Molluscipoxvirus:0.268496)78:0.022251,KU98
0965_Poxviridae_Unassigned:0.290747)88:0.052558)100:0.094117)84:0.0813
12,DQ356948_Poxviridae_Crocodylidpoxvirus:0.518733)96:0.031165,((((KJ8
01920_Poxviridae_Avipoxvirus:0.086696,AF198100_Poxviridae_Avipoxvirus:
0.086696)78:0.02026,KJ859677_Poxviridae_Avipoxvirus:0.106956)100:0.077
901,AY318871_Poxviridae_Avipoxvirus:0.184857)100:0.135831,KP728110_Pox
viridae_Avipoxvirus:0.320688)100:0.22921)100:0.134851,KT159937_Poxviri
dae_Unassigned:0.684749)100:0.085728,(((((HF679134_Poxviridae_Betaento
mopoxvirus:0.231244,HF679131_Poxviridae_Betaentomopoxvirus:0.231244)92
:0.030438,AF250284_Poxviridae_Betaentomopoxvirus:0.261682)72:0.020671,
(HF679133_Poxviridae_Betaentomopoxvirus:0.094871,HF679132_Poxviridae_B
etaentomopoxvirus:0.094871)100:0.187482)100:0.124921,AP013055_Poxvirid
ae_Alphaentomopoxvirus:0.407274)94:0.064633,AF063866_Poxviridae_Unassi
gned:0.471907)100:0.29857)100:0.073388,Query0471_KX522565:0.843865)66:
0.02568,(((((((((((KT989884_Iridoviridae_Ranavirus:0.003613,JQ724856
_Iridoviridae_Ranavirus:0.003613)100:0.040167,FJ433873_Iridoviridae_Ra
navirus:0.04378)96:0.025317,(KX574343_Iridoviridae_Unassigned:0.055416
,KX353311_Iridoviridae_Ranavirus:0.055416)78:0.013682)84:0.033767,((AY
548484_Iridoviridae_Ranavirus:0.042972,KX185156_Iridoviridae_Ranavirus
:0.042972)100:0.035509,AY150217_Iridoviridae_Ranavirus:0.078481):0.024
383)100:0.217109,AY521625_Iridoviridae_Ranavirus:0.319973)100:0.171347
,(L63545_Iridoviridae_Lymphocystivirus:0.25867,AY380826_Iridoviridae_L
ymphocystivirus:0.25867)100:0.232649)78:0.060507,AF371960_Iridoviridae
_Megalocytivirus:0.551827)100:0.114585,((((((HF920636_Iridoviridae_Iri
dovirus:0.046091,HF920634_Iridoviridae_Iridovirus:0.046091)86:0.012331
,HF920633_Iridoviridae_Iridovirus:0.058422)100:0.041139,(GQ918152_Irid
oviridae_Iridovirus:0.076419,HF920635_Iridoviridae_Iridovirus:0.076419
)100:0.023141)100:0.200261,(KF938901_Iridoviridae_Unassigned:0.263133,
DQ643392_Iridoviridae_Chloriridovirus:0.263133)72:0.036688)100:0.14479
7,(AF303741_Iridoviridae_Iridovirus:0.247604,HF920637_Iridoviridae_Iri
dovirus:0.247604)100:0.197014)100:0.153271,CU469068_Ascoviridae_Toursv
irus:0.597889)100:0.068522)100:0.087498,((((JX491653_Ascoviridae_Ascov
irus:0.068619,EF133465_Ascoviridae_Ascovirus:0.068619):0.023333,KJ7551
91_Ascoviridae_Ascovirus:0.091952)100:0.269324,AM398843_Ascoviridae_As
covirus:0.361275)100:0.145175,DQ517337_Ascoviridae_Ascovirus:0.506451)
100:0.247459)100:0.04096,(((KF483846_Marseilleviridae_Unassigned:0.139
258,HQ113105_Marseilleviridae_Unassigned:0.139258)100:0.060194,GU07108
6_Marseilleviridae_Marseillevirus:0.199452)100:0.063612,KT835053_Marse
illeviridae_Unassigned:0.263064)100:0.531805)96:0.021869,U18466_Asfarv
iridae_Asfivirus:0.816739)62:0.009619,((((EU304328_Phycodnaviridae_Pra
sinovirus:0.640382,AJ890364_Phycodnaviridae_Coccolithovirus:0.640382)8
2:0.06221,KX008963_Phycodnaviridae_Raphidovirus:0.702591)64:0.027732,(
EU916176_Phycodnaviridae_Phaeovirus:0.457379,AF204951_Phycodnaviridae_
Phaeovirus:0.457379)100:0.272944)82:0.038538,((DQ491002_Phycodnavirida
e_Chlorovirus:0.237924,JF411744_Phycodnaviridae_Chlorovirus:0.237924)1
00:0.164497,EF101928_Phycodnaviridae_Chlorovirus:0.402422)100:0.36644)
88:0.057496)86:0.024815,(HQ336222_Mimiviridae_Mimivirus:0.751075,GU244
497_Mimiviridae_Cafeteriavirus:0.751075)100:0.100097)88:0.018373):0.03
9992,(((((((((((((KM102981_Baculoviridae_Alphabaculovirus:0.092119,E
U839994_Baculoviridae_Alphabaculovirus:0.092119)94:0.029194,DQ123841_B
aculoviridae_Alphabaculovirus:0.121313)100:0.049175,KM009991_Baculovir
idae_Alphabaculovirus:0.170488)70:0.017873,((EU780426_Baculoviridae_Al

phabaculovirus:0.098327,AF169823_Baculoviridae_Alphabaculovirus:0.0983
27)100:0.053924,EF035042_Baculoviridae_Alphabaculovirus:0.152251)88:0.
036109):0.026082,((AY126275_Baculoviridae_Alphabaculovirus:0.044723,JQ
798165_Baculoviridae_Alphabaculovirus:0.044723)90:0.016454,U59461_Bacu
loviridae_Alphabaculovirus:0.061176)100:0.153266)100:0.057898,((DQ0173
80_Baculoviridae_Alphabaculovirus:0.098884,KJ631622_Baculoviridae_Alph
abaculovirus:0.098884)72:0.018347,AY864330_Baculoviridae_Alphabaculovi
rus:0.117231)100:0.15511)96:0.044386,(((((KF158713_Baculoviridae_Alpha
baculovirus:0.189526,FJ227128_Baculoviridae_Alphabaculovirus:0.189526)
72:0.015144,EU309041_Baculoviridae_Alphabaculovirus:0.20467)76:0.02267
8,DQ837165_Baculoviridae_Alphabaculovirus:0.227348):0.007261,KM596836_
Baculoviridae_Unassigned:0.234609)94:0.038991,(((KJ676450_Baculovirida
e_Alphabaculovirus:0.175763,KF611977_Baculoviridae_Alphabaculovirus:0.
175763)74:0.031926,FJ914221_Baculoviridae_Alphabaculovirus:0.207689)98
:0.036498,DQ504428_Baculoviridae_Alphabaculovirus:0.244187)94:0.029412
)88:0.043127)64:0.037026,KP752043_Baculoviridae_Alphabaculovirus:0.353
752):0.011149,((((EU591746_Baculoviridae_Alphabaculovirus:0.027863,AP0
06270_Baculoviridae_Alphabaculovirus:0.027863)100:0.291333,(AF303045_B
aculoviridae_Alphabaculovirus:0.014713,AF271059_Baculoviridae_Alphabac
ulovirus:0.014713)100:0.304483):0.01618,KR011717_Baculoviridae_Unassig
ned:0.335376):0.008514,AY394490_Baculoviridae_Alphabaculovirus:0.34389
):0.021011):0.028831,(GQ202541_Baculoviridae_Alphabaculovirus:0.128165
,AF081810_Baculoviridae_Alphabaculovirus:0.128165)100:0.265567):0.0058
58,(AF325155_Baculoviridae_Alphabaculovirus:0.071221,JX454574_Baculovi
ridae_Alphabaculovirus:0.071221)100:0.328369)100:0.04749,(((((((KC961
304_Baculoviridae_Alphabaculovirus:0.072522,AF512031_Baculoviridae_Alp
habaculovirus:0.072522)66:0.021725,KF894742_Baculoviridae_Alphabaculov
irus:0.094246)94:0.028256,AY043265_Baculoviridae_Alphabaculovirus:0.12
2502)62:0.010645,U75930_Baculoviridae_Alphabaculovirus:0.133148)64:0.0
04427,AP009046_Baculoviridae_Alphabaculovirus:0.137574):0.019111,DQ486
030_Baculoviridae_Alphabaculovirus:0.156686)74:0.031182,(((KR815466_Ba
culoviridae_Alphabaculovirus:0.020208,DQ813662_Baculoviridae_Alphabacu
lovirus:0.020208)100:0.070785,AY327402_Baculoviridae_Alphabaculovirus:
0.090993)84:0.016385,KJ631623_Baculoviridae_Alphabaculovirus:0.107378)
92:0.08049)100:0.058493,(((JX467702_Baculoviridae_Alphabaculovirus:0.0
80259,L22858_Baculoviridae_Alphabaculovirus:0.080259)100:0.03297,L3318
0_Baculoviridae_Alphabaculovirus:0.113229)98:0.06976,(EF125867_Baculov
iridae_Alphabaculovirus:0.145976,KU565883_Baculoviridae_Alphabaculovir
us:0.145976)78:0.037014)100:0.063372)100:0.200718)100:0.120505,((((((
(((((KJ406702_Baculoviridae_Betabaculovirus:0.090676,KR091910_Baculovi
ridae_Betabaculovirus:0.090676)100:0.08398,DQ333351_Baculoviridae_Beta
baculovirus:0.174656)62:0.016413,GQ884143_Baculoviridae_Betabaculoviru
s:0.191069)68:0.032501,(U53466_Baculoviridae_Betabaculovirus:0.166656,
AY229987_Baculoviridae_Betabaculovirus:0.166656)100:0.056914):0.014006
,(KC179784_Baculoviridae_Betabaculovirus:0.02341,HQ116624_Baculovirida
e_Betabaculovirus:0.02341)100:0.214166):0.016748,((KX151395_Baculoviri
dae_Betabaculovirus:0.212217,KP296186_Baculoviridae_Betabaculovirus:0.
212217):0.013493,AF499596_Baculoviridae_Betabaculovirus:0.22571)60:0.0
28614):0.002003,AF547984_Baculoviridae_Betabaculovirus:0.256327):0.031
143,JN408834_Baculoviridae_Betabaculovirus:0.28747)74:0.026332,AF27093
7_Baculoviridae_Betabaculovirus:0.313802)70:0.01382,KU593505_Baculovir
idae_Betabaculovirus:0.327622)82:0.032156,(KR584663_Baculoviridae_Beta

baculovirus:0.002399,AY522332_Baculoviridae_Betabaculovirus:0.002399)1
00:0.35738)98:0.074814,(((AF162221_Baculoviridae_Betabaculovirus:0.07
3969,EU255577_Baculoviridae_Betabaculovirus:0.073969)100:0.048718,EU67
8671_Baculoviridae_Betabaculovirus:0.122687)100:0.126621,(KM371112_Bac
uloviridae_Betabaculovirus:0.217939,KR011718_Baculoviridae_Betabaculov
irus:0.217939)86:0.03137)100:0.095657,DQ288858_Baculoviridae_Betabacul
ovirus:0.344965)96:0.089628)100:0.132992)100:0.172048,(((AY349019_Bacu
loviridae_Gammabaculovirus:0.073201,DQ317692_Baculoviridae_Gammabaculo
virus:0.073201)100:0.164441,AY430810_Baculoviridae_Gammabaculovirus:0.
237641)100:0.470611,AF403738_Baculoviridae_Deltabaculovirus:0.708252)8
4:0.03138)100:0.146268,(((KM610234_Nudiviridae_Unassigned:0.606902,KJ1
84318_Nudiviridae_Unassigned:0.606902)100:0.113063,(EU747721_Nudivirid
ae_Alphanudivirus:0.52934,EF203088_Nudiviridae_Alphanudivirus:0.52934)
100:0.190625)100:0.069632,(AF451898_Nudiviridae_Betanudivirus:0.027459
,JN418988_Nudiviridae_Betanudivirus:0.027459)100:0.762138)86:0.096304)
86:0.023635):0.005977,((((((((((KR351281_Herpesviridae_Cytomegaloviru
s:0.087677,FJ483968_Herpesviridae_Cytomegalovirus:0.087677)100:0.03579
7,AY186194_Herpesviridae_Cytomegalovirus:0.123475)100:0.133571,(AF4808
84_Herpesviridae_Cytomegalovirus:0.094132,AY446894_Herpesviridae_Cytom
egalovirus:0.094132)100:0.162914)100:0.056838,(FJ483967_Herpesviridae_
Cytomegalovirus:0.156423,FJ483970_Herpesviridae_Cytomegalovirus:0.1564
23)100:0.157461)100:0.07774,(AF281817_Herpesviridae_Unassigned:0.31961
5,KC503762_Herpesviridae_Unassigned:0.319615)100:0.07201)100:0.072718,
((JX867617_Herpesviridae_Muromegalovirus:0.237579,AF232689_Herpesvirid
ae_Muromegalovirus:0.237579)100:0.058689,U68299_Herpesviridae_Muromega
lovirus:0.296268)100:0.168075)100:0.061407,(((KU351741_Herpesviridae_R
oseolovirus:0.077169,AF037218_Herpesviridae_Roseolovirus:0.077169)100:
0.105204,(AF157706_Herpesviridae_Roseolovirus:0.039843,X83413_Herpesvi
ridae_Roseolovirus:0.039843)100:0.14253)100:0.148152,KF017583_Herpesvi
ridae_Unassigned:0.330525)100:0.195225)100:0.102916,((KC462165_Herpesv
iridae_Proboscivirus:0.055754,KF921519_Herpesviridae_Proboscivirus:0.0
55754)100:0.198706,KT832477_Herpesviridae_Proboscivirus:0.25446)100:0.
374205)76:0.054941,(((((((X64346_Herpesviridae_Rhadinovirus:0.066954,
AF083424_Herpesviridae_Rhadinovirus:0.066954)100:0.122419,AF318573_Her
pesviridae_Rhadinovirus:0.189372)60:0.012948,KT595939_Herpesviridae_Un
assigned:0.20232)78:0.031066,(AF083501_Herpesviridae_Rhadinovirus:0.15
0399,AF148805_Herpesviridae_Rhadinovirus:0.150399)100:0.082987)90:0.05
5336,(KM924295_Herpesviridae_Percavirus:0.084238,U20824_Herpesviridae_
Percavirus:0.084238)100:0.204484)84:0.034572,(((KF274499_Herpesviridae
_Macavirus:0.06434,AF005370_Herpesviridae_Macavirus:0.06434)100:0.0397
1,AY839756_Herpesviridae_Macavirus:0.10405)80:0.032841,KJ705001_Herpes
viridae_Macavirus:0.136891)100:0.186403)94:0.042375,(U97553_Herpesviri
dae_Rhadinovirus:0.204639,HQ221963_Herpesviridae_Rhadinovirus:0.204639
)100:0.16103)98:0.045687,(((DQ279927_Herpesviridae_Lymphocryptovirus:0
.025337,AJ507799_Herpesviridae_Lymphocryptovirus:0.025337)100:0.120189
,AY037858_Herpesviridae_Lymphocryptovirus:0.145527)100:0.080868,AF3197
82_Herpesviridae_Lymphocryptovirus:0.226395)100:0.184961)100:0.27225)8
8:0.040264,(((((((((((AP010838_Herpesviridae_Varicellovirus:0.021268,
AY665713_Herpesviridae_Varicellovirus:0.021268)84:0.007962,JQ343919_He
rpesviridae_Varicellovirus:0.02923)100:0.015937,AF030027_Herpesviridae
_Varicellovirus:0.045167)100:0.033941,KM051845_Herpesviridae_Varicello
virus:0.079108)100:0.067981,(FJ478159_Herpesviridae_Varicellovirus:0.1

03239,KT819633_Herpesviridae_Varicellovirus:0.103239)100:0.04385)100:0
.077256,(AY261359_Herpesviridae_Varicellovirus:0.050209,AJ004801_Herpe
sviridae_Varicellovirus:0.050209)100:0.174137):0.013537,(X04370_Herpes
viridae_Varicellovirus:0.068809,AF275348_Herpesviridae_Varicellovirus:
0.068809)100:0.169074)64:0.032401,BK001744_Herpesviridae_Varicelloviru
s:0.270284)70:0.008987,EU082088_Herpesviridae_Mardivirus:0.27927)74:0.
04745,((((((JQ360576_Herpesviridae_Simplexvirus:0.024434,JN561323_Her
pesviridae_Simplexvirus:0.024434)100:0.030716,JN555585_Herpesviridae_S
implexvirus:0.05515)100:0.027198,AB825953_Herpesviridae_Simplexvirus:0
.082348)72:0.009921,((DQ149153_Herpesviridae_Simplexvirus:0.043079,AF5
33768_Herpesviridae_Simplexvirus:0.043079):0.000295,AY714813_Herpesvir
idae_Simplexvirus:0.043374)100:0.048895)100:0.050461,HM625781_Herpesvi
ridae_Simplexvirus:0.142729)84:0.019568,KT594769_Herpesviridae_Simplex
virus:0.162298)96:0.034365,JQ596859_Herpesviridae_Simplexvirus:0.19666
3)100:0.130057):0.03103,(((AB049735_Herpesviridae_Mardivirus:0.117737,
AF243438_Herpesviridae_Mardivirus:0.117737):0.015056,AF291866_Herpesvi
ridae_Mardivirus:0.132793)100:0.184357,(KJ668231_Herpesviridae_Mardivi
rus:0.004157,KX589235_Herpesviridae_Mardivirus:0.004157)100:0.312993):
0.0406)100:0.184878,((AY372243_Herpesviridae_Iltovirus:0.305228,NC_006
623_Herpesviridae_Iltovirus:0.305228)100:0.198345,KM924292_Herpesvirid
ae_Scutavirus:0.503573)90:0.039055)100:0.181242)100:0.169508,(EU522111
_Hytrosaviridae_Muscavirus:0.739328,EF568108_Hytrosaviridae_Glossinavi
rus:0.739328)80:0.154049):0.022136):0.024824):0.024337,((((Query0212_M
G065686:0.00037,Query0083_MG065691:0.00037)100:0.689425,Query0126_AP01
7903:0.689795)92:0.171334,(Query0486_KY984068:0.741889,AB366653_Myovir
idae_Rslunavirus:0.741889)72:0.119241):0.091802,((DQ665652_Alloherpesv
iridae_Batrachovirus:0.58748,DQ665917_Alloherpesviridae_Batrachovirus:
0.58748)100:0.167588,M75136_Alloherpesviridae_Ictalurivirus:0.755068)1
00:0.197864):0.011743):0.005216,((((((((((((((Query0192_KY883652:0.0
,Query0191_KY883651:0.0)100:0.0,Query0193_KY883653:0.0)100:0.012233,(Q
uery0334_KY883648:0.005097,Query0190_KY883650:0.005097)80:0.007136)72:
0.008816,((Query0342_KY883646:0.004395,Query0339_KY883641:0.004395)68:
0.00067,Query0333_KY883649:0.005065)100:0.015984):0.017913,(Query0343_
KY883645:0.024345,Query0337_KY883643:0.024345):0.014617)78:0.027795,Qu
ery0344_KY883644:0.066757)100:0.203194,((((Query0242_MF285616:0.002517
,Query0241_MF285617:0.002517)100:0.072126,Query0332_MF285620:0.074643)
100:0.048276,Query0287_MF979561:0.122919)74:0.042467,Query0505_KY25003
5:0.165386)92:0.104565):0.109577,((((((Query0068_KY652725:0.063422,Que
ry0067_KY652724:0.063422):0.016137,(Query0240_MF285615:0.057964,Query0
073_KY652723:0.057964):0.021595):0.035428,KT367885_Podoviridae_Kp32vir
us:0.114988):0.011626,((LN866626_Podoviridae_Kp32virus:0.023563,HM4808
46_Podoviridae_Kp32virus:0.023563)100:0.092569,EU734173_Podoviridae_Kp
32virus:0.116132):0.010481):0.024465,(GQ413937_Podoviridae_Kp32virus:0
.106261,KR149291_Podoviridae_Kp32virus:0.106261)88:0.044817)94:0.06585
7,Query0295_MG835568:0.216935)100:0.162592)62:0.082427,Query0381_MF979
559:0.461954)82:0.147406,((Query0294_MG018929:0.307988,Query0121_MG545
917:0.307988):0.034637,Query0419_KY250034:0.342625)100:0.266736):0.153
096,((((((((KX211991_Podoviridae_Kp34virus:0.055239,KT964103_Podoviri
dae_Kp34virus:0.055239)76:0.042839,KT367886_Podoviridae_Kp34virus:0.09
8078):0.023715,(KU666550_Podoviridae_Kp34virus:0.059632,AB716666_Podov
iridae_Kp34virus:0.059632)86:0.06216):0.010818,((KP708985_Podoviridae_
Kp34virus:0.091171,KF765493_Podoviridae_Kp34virus:0.091171):0.014025,G

Q413938_Podoviridae_Kp34virus:0.105195):0.027415):0.014586,KP708986_Po
doviridae_Kp34virus:0.147196)100:0.287038,Query0492_JX290549:0.434235)
98:0.101674,((((KT388103_Podoviridae_Fri1virus:0.074675,KT388102_Podov
iridae_Fri1virus:0.074675):0.01533,(HQ186308_Podoviridae_Fri1virus:0.0
29055,JX658790_Podoviridae_Fri1virus:0.029055)100:0.06095):0.0235,(KT8
04908_Podoviridae_Fri1virus:0.097478,KR149290_Podoviridae_Fri1virus:0.
097478):0.016027)100:0.130016,KC311669_Podoviridae_Fri1virus:0.243521)
100:0.292388)98:0.114677,((((KF626667_Podoviridae_Pradovirus:0.111959,
KU595433_Podoviridae_Pradovirus:0.111959)82:0.021423,Query0036_KU19701
3:0.133382)88:0.036364,KU595432_Podoviridae_Pradovirus:0.169746)100:0.
343321,((Query0306_MG775261:0.258419,Query0235_KY316062:0.258419):0.04
7187,Query0524_KX756572:0.305606)100:0.207461)68:0.137519):0.089267,((
(AY299121_Siphoviridae_Xp10virus:0.069217,DQ777876_Siphoviridae_Xp10vi
rus:0.069217)98:0.039732,AP008979_Siphoviridae_Xp10virus:0.108949)100:
0.151159,(EU717894_Siphoviridae_Xp10virus:0.166377,AB720063_Siphovirid
ae_Xp10virus:0.166377)100:0.093731)100:0.479744):0.022604):0.070545,(Q
uery0196_KY883657:0.185558,Query0189_KY883658:0.185558)100:0.647444):0
.057096,(((((KC294142_Podoviridae_Luz24virus:0.051393,KF856712_Podovir
idae_Luz24virus:0.051393)84:0.031627,KP233880_Podoviridae_Luz24virus:0
.083021)72:0.019002,(HG518155_Podoviridae_Luz24virus:0.045306,LN610578
_Podoviridae_Luz24virus:0.045306)100:0.056717)100:0.240555,(Query0552_
MG845684:0.198345,Query0487_MG775259:0.198345)100:0.144233)100:0.37636
3,((((JX867715_Podoviridae_Phieco32virus:0.018843,KP308307_Podoviridae
_Phieco32virus:0.018843)100:0.057119,KM044272_Podoviridae_Phieco32viru
s:0.075962)80:0.02109,JX415536_Podoviridae_Phieco32virus:0.097052):0.0
0925,KF981730_Podoviridae_Phieco32virus:0.106302)100:0.612638)90:0.171
158):0.040374,(Query0525_KJ410740:0.83664,Query0500_MF580957:0.83664):
0.093832):0.010897,(((EU100884_Siphoviridae_P23virus:0.012291,EU100883
_Siphoviridae_P23virus:0.012291)100:0.877936,KT151955_Siphoviridae_Jen
stvirus:0.890227):0.016576,KU160638_Siphoviridae_Amigovirus:0.906803):
0.034566):0.016368,Query0124_KR296693:0.957738):0.012153):0.006485):0.
015682,(((HG531932_Lavidaviridae_Sputnikvirus:0.147222,EU606015_Lavida
viridae_Sputnikvirus:0.147222)100:0.704516,HQ712116_Lavidaviridae_Mavi
rus:0.851738)74:0.127465,(Query0576_GU722197:0.931466,AJ888457_Bicauda
viridae_Bicaudavirus:0.931466)74:0.047737):0.012855):0.001142,Query025
4_MG065651:0.9932):0.000505,((((((((((AM087121_Lipothrixviridae_Betal
ipothrixvirus:0.026687,AM087120_Lipothrixviridae_Betalipothrixvirus:0.
026687)82:0.019806,AM087123_Lipothrixviridae_Betalipothrixvirus:0.0464
93)100:0.144111,EU545650_Lipothrixviridae_Betalipothrixvirus:0.190605)
100:0.157312,AM087122_Lipothrixviridae_Betalipothrixvirus:0.347916)62:
0.028737,AF440571_Lipothrixviridae_Betalipothrixvirus:0.376653)90:0.32
858,AJ854042_Lipothrixviridae_Deltalipothrixvirus:0.705234)90:0.148649
,(((AJ344259_Rudiviridae_Rudivirus:0.085873,AJ414696_Rudiviridae_Rudiv
irus:0.085873)100:0.248543,AJ875026_Rudiviridae_Rudivirus:0.334416)100
:0.442716,AJ567472_Lipothrixviridae_Gammalipothrixvirus:0.777132):0.07
6751):0.020496,KY780159_Portogloboviridae_Alphaportoglobovirus:0.87437
9)96:0.062204,EF432053_Ampullaviridae_Ampullavirus:0.936583)82:0.01037
1,(GU080336_Turriviridae_Alphaturrivirus:0.263182,AY569307_Turrivirida
e_Alphaturrivirus:0.263182)100:0.683772)78:0.040618,((((((AY388628_Fus
elloviridae_Alphafusellovirus:0.203844,AY370762_Fuselloviridae_Alphafu
sellovirus:0.203844):0.020621,(EU030939_Fuselloviridae_Alphafusellovir
us:0.01084,EU030938_Fuselloviridae_Alphafusellovirus:0.01084)100:0.213

625)62:0.079354,X07234_Fuselloviridae_Alphafusellovirus:0.303818):0.06
7242,(AY423772_Fuselloviridae_Alphafusellovirus:0.314772,FJ870916_Fuse
lloviridae_Alphafusellovirus:0.314772):0.056288)72:0.171223,(FJ870915_
Fuselloviridae_Betafusellovirus:0.373791,FJ870917_Fuselloviridae_Betaf
usellovirus:0.373791)66:0.168492)100:0.364509,(((Query0577_JQ010983:1e
-06,Query0575_JF917095:1e-
06)94:0.727314,Query0578_LC208019:0.727315):0.148749,HE580237_Guttavir
idae_Betaguttavirus:0.876064):0.030728)84:0.080779):0.006133):0.001633
,((((((AY701338_Tectiviridae_Betatectivirus:0.125514,AY257527_Tectivir
idae_Betatectivirus:0.125514)96:0.137046,EU408779_Tectiviridae_Betatec
tivirus:0.26256)62:0.137881,KF188458_Tectiviridae_Betatectivirus:0.400
441)100:0.542688,Query0579_MF186604:0.943129)60:0.017481,(AY848689_Tec
tiviridae_Alphatectivirus:0.011393,AY848686_Tectiviridae_Alphatectivir
us:0.011393)100:0.949217):0.02334,(((Query0470_MF001367:0.749173,Query
0173_CP002495:0.749173)94:0.135543,(LK392619_Podoviridae_Cp1virus:0.08
1936,Z47794_Podoviridae_Cp1virus:0.081936)100:0.80278)92:0.094966,Quer
y0290_MF979562:0.979682):0.004267):0.011389):0.001176,(((((((((((((K
C693023_Adenoviridae_Mastadenovirus:0.019628,KC693022_Adenoviridae_Mas
tadenovirus:0.019628):0.004722,KP329565_Adenoviridae_Mastadenovirus:0.
02435)100:0.014091,AY771780_Adenoviridae_Mastadenovirus:0.038442)86:0.
01828,L19443_Adenoviridae_Mastadenovirus:0.056722)84:0.02477,(((KC6930
21_Adenoviridae_Mastadenovirus:0.013048,HQ241819_Adenoviridae_Mastaden
ovirus:0.013048)100:0.017847,KP329561_Adenoviridae_Mastadenovirus:0.03
0894)84:0.018988,KP329564_Adenoviridae_Mastadenovirus:0.049882)90:0.03
161)60:0.015892,X73487_Adenoviridae_Mastadenovirus:0.097384):0.008741,
(FJ025931_Adenoviridae_Mastadenovirus:0.078951,J01917_Adenoviridae_Mas
tadenovirus:0.078951)72:0.027174):0.013323,(((HQ605912_Adenoviridae_Ma
stadenovirus:0.027068,AY598782_Adenoviridae_Mastadenovirus:0.027068)10
0:0.055638,KX505867_Adenoviridae_Mastadenovirus:0.082706)86:0.030815,(
KM190146_Adenoviridae_Mastadenovirus:0.026132,KP329563_Adenoviridae_Ma
stadenovirus:0.026132)100:0.087389):0.005928):0.01792,(((AY163756_Aden
oviridae_Mastadenovirus:0.034525,DQ086466_Adenoviridae_Mastadenovirus:
0.034525)100:0.034484,(AY487947_Adenoviridae_Mastadenovirus:0.021777,J
N254802_Adenoviridae_Mastadenovirus:0.021777)100:0.047232)70:0.023368,
(AJ854486_Adenoviridae_Mastadenovirus:0.022803,AB333801_Adenoviridae_M
astadenovirus:0.022803)100:0.069574)92:0.044991):0.01378,HQ913600_Aden
oviridae_Mastadenovirus:0.151148)98:0.090939,(((((((KT698852_Adenovir
idae_Mastadenovirus:0.034715,KT698856_Adenoviridae_Mastadenovirus:0.03
4715)100:0.111598,KJ563221_Adenoviridae_Mastadenovirus:0.146313):0.011
694,KT160425_Adenoviridae_Mastadenovirus:0.158006):0.013377,AF030154_A
denoviridae_Mastadenovirus:0.171384):0.017017,((AF289262_Adenoviridae_
Mastadenovirus:0.128599,AC_000191_Adenoviridae_Mastadenovirus:0.128599
)72:0.01277,(AC_000001_Adenoviridae_Mastadenovirus:0.0,AF252854_Adenov
iridae_Mastadenovirus:0.0)100:0.141369)78:0.047032):0.012004,AC_000190
_Adenoviridae_Mastadenovirus:0.200405):0.006701,AC_000189_Adenoviridae
_Mastadenovirus:0.207106):0.020484,(((((GU226970_Adenoviridae_Mastaden
ovirus:0.034847,KX871230_Adenoviridae_Mastadenovirus:0.034847)72:0.013
163,JN252129_Adenoviridae_Mastadenovirus:0.048009):0.001713,KP238322_A
denoviridae_Mastadenovirus:0.049722)72:0.015492,Y07760_Adenoviridae_Ma
stadenovirus:0.065214)98:0.050478,JN418926_Adenoviridae_Mastadenovirus
:0.115692)100:0.111898):0.014497)78:0.039642,((KT698855_Adenoviridae_M
astadenovirus:0.008762,KT698854_Adenoviridae_Mastadenovirus:0.008762)7

4:0.006986,KT698853_Adenoviridae_Mastadenovirus:0.015747)100:0.265981)
80:0.047717,(EU835513_Adenoviridae_Mastadenovirus:0.241964,HM049560_Ad
enoviridae_Mastadenovirus:0.241964)100:0.087481)100:0.15471,(((((AF03
6092_Adenoviridae_Atadenovirus:0.047463,JQ345700_Adenoviridae_Atadenov
irus:0.047463)82:0.015669,U40839_Adenoviridae_Atadenovirus:0.063132)10
0:0.159691,Y09598_Adenoviridae_Atadenovirus:0.222823):0.013288,(DQ1064
14_Adenoviridae_Atadenovirus:0.134337,KJ156523_Adenoviridae_Atadenovir
us:0.134337)94:0.101774):0.035444,KJ675568_Adenoviridae_Atadenovirus:0
.271555)100:0.14802,((((HM585353_Adenoviridae_Siadenovirus:0.034297,EU
715130_Adenoviridae_Siadenovirus:0.034297)72:0.029026,KP144329_Adenovi
ridae_Siadenovirus:0.063323):0.007833,(AC_000016_Adenoviridae_Siadenov
irus:0.0,AF074946_Adenoviridae_Siadenovirus:0.0)100:0.071156)88:0.0611
86,AF224336_Adenoviridae_Siadenovirus:0.132342)100:0.287233)92:0.06458
)96:0.06589,((((((GU734104_Adenoviridae_Aviadenovirus:0.100281,AF08397
5_Adenoviridae_Aviadenovirus:0.100281)100:0.093222,GU936707_Adenovirid
ae_Aviadenovirus:0.193502):0.018967,((KF477312_Adenoviridae_Aviadenovi
rus:0.042412,KC493646_Adenoviridae_Aviadenovirus:0.042412)100:0.119907
,GU188428_Adenoviridae_Aviadenovirus:0.162318)68:0.050151):0.013073,(K
F477313_Adenoviridae_Aviadenovirus:0.121578,U46933_Adenoviridae_Aviade
novirus:0.121578)98:0.103964):0.032616,(FN824512_Adenoviridae_Aviadeno
virus:0.14316,KX121164_Adenoviridae_Aviadenovirus:0.14316)96:0.114998)
72:0.030392,(JF510462_Adenoviridae_Aviadenovirus:0.095786,KJ469653_Ade
noviridae_Aviadenovirus:0.095786)100:0.192765)100:0.261494)100:0.44646
9):0.001776,(((AB290007/AB290006/AB290005/..._Polydnaviridae_Ichnoviru
s:0.585324,AJ632330/AJ632320/AJ632317/..._Polydnaviridae_Bracovirus:0.
585324)100:0.293818,(AB291209/AB291208/AB291207/..._Polydnaviridae_Ich
novirus:0.327954,AY029400/AF004378/U41655/..._Polydnaviridae_Ichnoviru
s:0.327954)100:0.551188)80:0.117951,(X14855_Tristromaviridae_Alphatris
tromavirus:0.786397,KU307456_Tristromaviridae_Alphatristromavirus:0.78
6397)100:0.210695):0.001198):0.000693,((((AY950802_Sphaerolipoviridae_
Alphasphaerolipovirus:0.135759,KC252997_Sphaerolipoviridae_Alphasphaer
olipovirus:0.135759)100:0.192837,KT809302_Sphaerolipoviridae_Alphaspha
erolipovirus:0.328596)66:0.047369,JN968479_Sphaerolipoviridae_Alphasph
aerolipovirus:0.375964)100:0.552479,AY048850_Sphaerolipoviridae_Betasp
haerolipovirus:0.928444)100:0.070539):0.000605,Query0137_FR725450:0.99
9588):1.1e-05,Query0263_MF361639:0.999599):8.6e-
05,Query0163_MG065660:0.999686):0.000141,(AY722806_Globuloviridae_Glob
ulovirus:0.719259,AJ635161_Globuloviridae_Globulovirus:0.719259)100:0.
280567):0.000145,(Query0574_MF443783:0.000369,Query0573_MF443784:0.000
369)100:0.999602):2.8e-05,AB537968_Clavaviridae_Clavavirus:1.0)100;

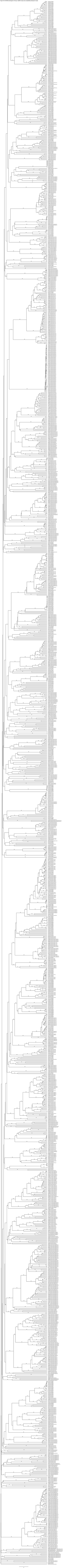

Figure S10 UPGMA dendrogram of Group I dsDNA viruses and unclassified prokaryotic viruses
